# Systematic Study of the Behavior of Different Metal and Metal-Containing Particles under the Microwave Irradiation and Transformation of Nanoscale and Microscale Morphology

**DOI:** 10.3390/nano9010019

**Published:** 2018-12-24

**Authors:** Evgeniy O. Pentsak, Vera A. Cherepanova, Mikhail A. Sinayskiy, Andrey V. Samokhin, Valentine P. Ananikov

**Affiliations:** 1 Zelinsky Institute of Organic Chemistry, Russian Academy of Sciences, Moscow 119991, Russia; p_eugene@ioc.ac.ru (E.O.P.); cherepanova.vera.a@gmail.com (V.A.C.); 2 Baikov Institute of Metallurgy and Material Science (IMET), Russian Academy of Sciences, Moscow 119334, Russia; sinaisky@imet.ac.ru (M.A.S.); samokhin@imet.ac.ru (A.V.S.)

**Keywords:** microwave irradiation, metal nanoparticles, metal composite nanoparticles, carbide nanoparticles, metal oxide nanoparticles, M/C catalyst, graphite support, carbon pitting, carbon etching

## Abstract

In recent years, the application of microwave (MW) irradiation has played an increasingly important role in the synthesis and development of high performance nanoscale catalytic systems. However, the interaction of microwave irradiation with solid catalytic materials and nanosized structures remains a poorly studied topic. In this paper we carried out a systematic study of changes in morphology under the influence of microwave irradiation on nanoscale particles of various metals and composite particles, including oxides, carbides, and neat metal systems. All systems were studied in the native solid form without a solvent added. Intensive absorption of microwave radiation was observed for many samples, which in turn resulted in strong heating of the samples and changes in their chemical structure and morphology. A comparison of two very popular catalytic materials—metal particles (M) and supported metal on carbon (M/C) systems—revealed a principal difference in their behavior under microwave irradiation. The presence of carbon support influences the heating mechanism; the interaction of substances with the support during the heating is largely determined by heat transfer from the carbon. Etching of the carbon surface, involving the formation of trenches and pits on the surface of the carbon support, were observed for various types of the investigated nanoparticles.

## 1. Introduction

Microwave irradiation is actively used in different fields of chemistry [1] including organic synthesis [2,3,4], catalyst preparation [5], carbon material synthesis [6,7], nanoparticle preparation [8,9], biomass conversion [10], hydrocarbon conversion [11], and many others. Microwave irradiation is successfully used for the heating of reaction medium and for the preparation and activation of catalysts. Rapid heating to high temperatures is more effectively achieved with microwave irradiation than with conventional heaters.

Usually, the microwave-assisted heterogeneous reactions, as well as preparation of catalysts and nanoparticles involve some liquid phase (solvents). For this reason, the average temperature of the system should not exceed 300 °C [12,13]; moreover, most of the commercial laboratory microwave systems have operating limitations for higher temperatures [14]. Under these conditions, microwave heating may only result in a reduction of the catalyst precursor and immobilization of the catalytic particles on the support, but not in the modification of the support. On the other hand, the problem of “hot spots” (high temperature microdomains) is one of the challenges of using microwave heating for using of heterogeneous catalysts [12,15]. The temperature within hot spots is reported at least 200 °C higher than in the rest of the sample. Nevertheless, localized overheating up to 900 °C is mentioned in some works [16]. This overheating may result in the interaction of metal particles with the support. Also, microwave irradiation can be used to prepare [17] calcine [18] and activate [19] catalysts at higher temperatures. In addition, microwave radiation is used in the conversion of hydrocarbons, which also proceeds at high temperatures [20,21,22]. In some studies, microwave irradiation is used to reduce the effect of the deactivation of the catalyst by coke formation during hydrocarbon processing, or to regenerate such catalysts [23,24,25]. The catalysts used for hydrocarbon processing are usually transparent for microwaves and therefore do not heat up under the action of microwaves. It is more likely that the carbon formed by coking can start to absorb the microwaves, which may result in specific interactions between the carbon and the catalyst. Thus, a whole range of important processes involving the interaction of inorganic components can occur at higher temperatures (above the common 300 °C). These processes may lead to significant changes in the morphology of the catalytic system.

Different classes of inorganic particles (metals, carbides, oxides, composite particles) can play diverse roles in catalysis as precursors of catalysts, supports, or active catalytic centers [26]. Interactions of various substances that can be used in heterogeneous catalysis with microwave irradiation have been studied extensively [1,5,8,15,19]. At the same time, the effects of microwave irradiation on the morphology of solid catalyst precursors, supports and catalytic particles, described in a limited number of works, have not yet been fully understood. The character of morphological changes during microwave processing is of fundamental interest. The investigation of these changes may help to determine the possible roles of various classes of particles in the formation of hot spots under the microwaving conditions.

Also, an impressive amount of research has been done on the microwave-mediated sintering of metals, alloys, and ceramics [27,28,29,30]. It shows that microwaves can be effectively used for sintering various metal-containing particles. However, as these studies were predominantly focused on obtaining structural materials, the microwave processing was usually carried out under specific conditions; i.e. inert or reducing atmosphere, the use of microwave-adsorbing “susceptor”, prolonged reaction times, high irradiation power, etc. Such conditions have no connection with studying morphological changes of catalytic systems or their components. As the majority of organic transformations are carried out in open air, it would be appropriate to investigate the changes in nanoscale morphology under aerated conditions, notably to assess the impact of sample oxidation during treatment and what morphological changes are caused by it.

Another important challenge concerns the interactions of supported metal-containing particles with carbon materials. Carbon supports, including graphitic structures, are used for many types of catalysts for a wide range of reactions [31,32,33,34,35,36,37,38]; they play a special role in the catalytic processes under microwave irradiation. Carbon supports work very well as a microwave radiation sensitizer: they are rapidly heated to high temperatures in a microwave oven. Besides, as noted above, interactions of heterogeneous catalysts with carbon particles may improve the efficiency of hydrocarbon conversion processes.

The effect of microwave irradiation has been studied for some systems, such as Pd/C, Pt/C, Ni/C. A number of metals and metal compounds have been shown to promote surface modifications of the support (in the form of trenches and pits) [6,39,40]. The effects of microwave irradiation on mixtures of graphite and carbon black with magnetite or silicon carbide have also been studied [41,42], but the concomitant morphological changes have not been examined. Another possibility is the cutting of carbon materials with metal particles [43,44]. Such modifications of the support morphology are of considerable interest for the development of heterogeneous catalytic systems. However, no systematic study on compound specificity of such interactions has been carried out previously. Possible correlations between the microwave absorbance by deposited particles and their interference with the morphology of carbon support remain to be explored. Dedicated studies are necessary to determine the relationship between the type of interactions of deposited substances with microwaves and their ability to promote morphological changes at the support surface.

## 2. Materials and Methods 

For the broadest coverage of various types of interactions of materials with microwaves, nano- and micron-sized powders of 41 substances obtained by plasma-chemical synthesis were selected. The list included 14 oxides, 4 carbides, 6 metals, 3 metal compositions, 8 metal compositions with carbon, 4 nitrogen compounds, 1 metal composition with boron and 1 sulfide (Table S1). The substances consisted of individual particles of a near-spherical or faceted shape. The average particle size varied in a wide range of 8–210 nm; the particle size distributions were close to log-normal. Metal powders and oxygen-free metal compounds are capable of interactions with atmospheric oxygen [45,46].

It was important to compare changes in morphology for substances with different types of interaction with microwaves. The conventional classification of substances by the type of interaction with microwaves is as follows: materials transparent to microwaves; bulk metals/conductive materials reflecting microwave radiation; materials heated by dielectric losses or/and magnetic losses or/and conductive losses. At the same time, changes in the nature of materials during the heating process infer concomitant changes in the mechanisms of their interaction with microwaves. Moreover, mechanisms of interaction of substances with microwaves depend not only on the chemical nature of the substance but also on the dispersity, shape and size of the particles, as well as the properties of radiation.

### 2.1. Initial Samples Preparation

Initial samples of metal-containing powders were received from IMET. These substances were prepared by the method of plasma-chemical synthesis in the interaction of various types of metal-containing raw materials (pure metals, oxides, chlorides, hydroxides; see Table S1 for details) with: oxygen for the preparation of oxides, hydrogen for the production of metals, hydrogen and carbon-containing gases for the preparation of carbides and metal-carbon systems, nitrogen included in the plasma stream for the preparation of nitrides. A direct current plasma torch with a power up to 30 kW was used as a thermal plasma generator. The raw materials in the form of dispersed materials with a particle size of less than 50 µm or vapors (in the case of titanium and silicon chlorides) were injected into the plasma jet using a carrier gas. The design of the reactor provides complete evaporation of the feedstock, the target chemical interaction in the gas phase with the formation of vapors of the target product and the formation of nanoparticles by condensation and coagulation mechanisms [46,47]. The resulting nanoparticles were deposited on the water-cooled walls of the reactor with the formation of a loose, easily removable layer and partially carried with the exhaust gases into the filtration apparatus.

### 2.2. General Procedure

The samples of powders (~20 mg) were placed into quartz vials with a layer height of not more than 5 mm. A quartz vial with a sample was mounted on a thermally insulating disk of zirconium oxide, which was rotated during experiment to provide the uniform treatment of the sample. The vials remained open during the experiments performed in air. The microwave treatment was carried out using a microwave system equipped with a Panasonic 2M210-M1 magnetron operating at a frequency of 2450 MHz and a power of 900 W. The samples were treated by microwave irradiation for one and five minutes in a continuous mode. The experiments were carried out at least three times for every sample that demonstrated interactions with microwaves. The experiments were conducted several times for nanoparticles of metals and some other samples to make observations of the sporadic effects of the occurrence of a spark discharge. A load of 20 mg was chosen due to the fact that a smaller amount of substances did not efficiently absorb the microwaves (for example, for several samples studied, phenomena did not occur if only 1–10 mg of compounds were used in the experiment).

To study the interactions with graphite, the samples of metal-containing powders (15 mg) were mixed with graphite (100 mg) and grinded in a mortar. Graphite (high-purity synthetic graphite powder with particle diameters of 10−140 μm) was obtained from a commercial source. Graphite was chosen as a model material for the combination of its rich morphology with the presence of defect-free surface areas that would allow detection of even subtle interactions [48,49,50]. Microwave treatment of the samples was carried out in the same way as described above.

Considering the amounts of sample, it was important to carry out the experiments using a practically convenient ratio for preparation and characterization of new materials. Another important issue is to maintain an optimal amount of sample for efficient and uniform microwave heating in the desired temperature range. Smaller amounts of sample may not efficiently absorb microwaves, while larger amounts of sample may be heated non-uniformly.

The above temperature measurements were made using the infrared thermometers “Kelvin CB Dipol” and Mastech MS6530. But in view of the known difficulties with the implementation of accurate measurements under microwave radiation conditions, the registration was carried out at the time when the magnetron was off, which can result in appreciable errors.

### 2.3. Scanning Electron Microscope (SEM) and Energy Dispersive X-Ray Spectroscopy (EDX) Studies

A target-oriented approach was utilized for the optimization of the analytic measurements [51]. Before measurements, the samples were placed on a 25 mm aluminum specimen stub and fixed by conductive graphite adhesive tape. Sample morphology was studied under native conditions to exclude the metal coating surface effects [52]. The observations were carried out using a Hitachi SU8000 (Hitachi High-Technologies Corporation, Hitachinaka-shi, Japan) field-emission scanning electron microscope (FE-SEM). The images were acquired in a secondary electron mode at a 2–30 kV accelerating voltage and at working distances of 8–10 mm.

For every compound that demonstrated interactions with microwaves, microscopic control of morphology changes was carried out by the investigation of samples obtained in at least two independent experiments. A surface of at least three independent regions was investigated for each sample. The analysis of nanoparticle sizes was performed using a Digimizer v. 4.3.0 software package (MedCalc Software, Ostend, Belgium). Value of d_av_ for Table S2 was calculated as a mean for one hundred particle diameter measurements from several areas of sample.

EDX studies were carried out using an Oxford Instruments X-max EDX system. The spectra and elemental distribution maps were recorded at a 30 kV accelerating voltage.

### 2.4. Surface Area Measurements and X-Ray Phase Analysis (XRD) Study

The samples were measured for the total specific external and internal surface area of dispersed or porous solid particles by measuring the amount of physically adsorbed gas in accordance with the Brunauer, Emmett and Teller method (BET method) according to ISO 9277:1995. A TriStar 3000 (Micromeritics, Norcross, GA, USA) specific surface and porosity analyzer was used for the measurements. Average diameter of initial compound particles (D_BET_) for Table S1 was calculated as D_BET_ = 6000/(q·S), where S is the measured surface area of the powder in m^2^/g and q is the theoretical density in g/cm^3^.

XRD was performed on an Ultima-4 diffractometer (Rigaku Corporation, Akishima-shi, Tokyo, Japan) in filtered Cu Kα radiation, with a high-speed D/teX detector, PDXL software package (Rigaku Corporation, Akishima-shi, Tokyo, Japan), and PDF-2 database.

## 3. Results and Discussion

**Figure 1:**
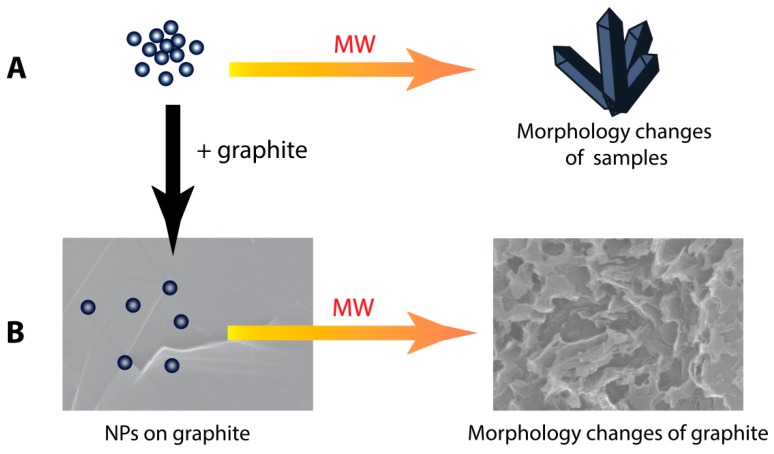
Possible effect of MW-treatment on metal-containing substances (**A**); and MW-treatment effect on graphite support in presence of metal-containing substances (**B**).

In the first part of the work we studied the interaction of nano- and micrometer particles of various metals and their compounds with microwave irradiation under model conditions (Figure 1A). The morphologies were investigated by SEM; additionally, structural study of the samples by XRD was carried out. Detailed descriptions of morphological changes for each sample are given in Table S2. In the second part, we studied interactions of the samples with graphite under similar conditions (Figure 1B), and a relationship between the ability of the initial samples to absorb microwave irradiation and their ability to etch graphite.

### 3.1. Investigation of Morphological Changes in Metal-Containing Substances under Microwave Treatment Conditions

**Figure 2:**
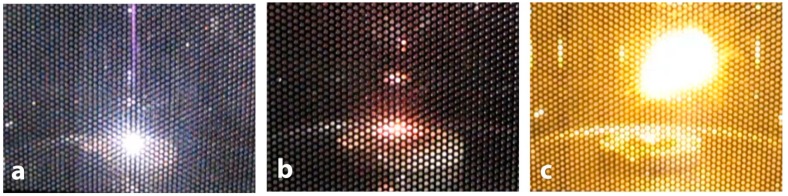
Examples of microwave process types. Type 2: a single spark discharge of Ag powder (**a**); type 3: red heat of W-C powder (**b**); type 4: glow of plasma observed for Mo-Fe-C powder (**c**).

To study structural changes in the initial powders under microwaving, the treatment was carried out for five minutes at power of 900 W. By their behavior under the microwave irradiation conditions, the samples could be conventionally divided into four types: (type 1) the absence of any manifestations during microwave processing or low heat; (type 2) weak MW-absorption or reflection of microwaves: a single spark discharge; (type 3) middle MW-absorption: red heat and red sparks; (type 4) intensive MW-absorption: spark discharges, glow of plasma, flame appearance with red heat (Figure 2).

As expected, the oxides were transparent to microwaves; accordingly, no external signs of microwave absorption and interaction with the microwaves were observed, and no increases in temperature above 200 °C were detected. At such temperatures, morphological changes in the samples are unlikely. A similar result was obtained for compounds with nitrogen and boron: AlN, TiN, TiCN, AlON, and Al-B. No changes in the composition of the material under the influence of microwaves were observed.

By contrast, other samples actively interacted with microwaves, that was manifested by the appearance of spark discharges, heating up to the temperature of red heat, and plasma flashes. 

**Figure 3:**
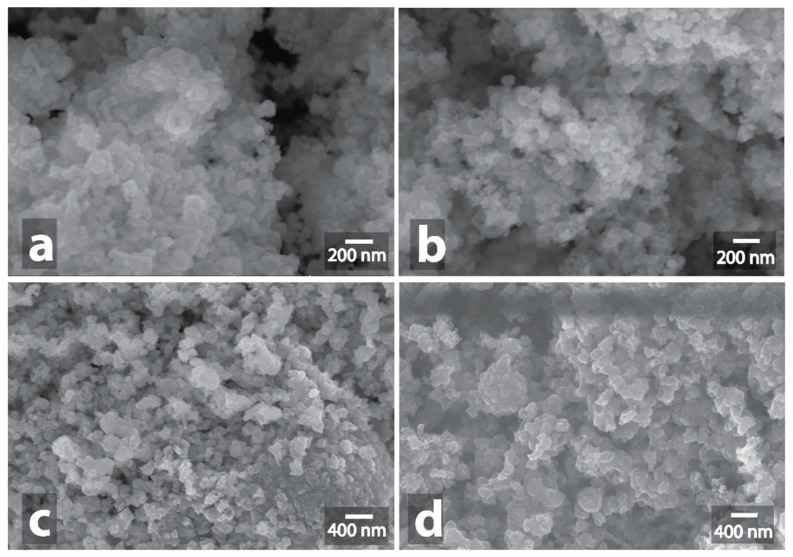
Scanning electron microscope (SEM) image of initial Cu powder (**a**) and SEM image of Cu powder after MW-treatment (**b**), initial Cu/C powder (**c**) and SEM image of Cu/C powder after MW-treatment (**d**).

It is generally known [28] that metals reflect microwaves and, as a result, the heating does not occur. However, a spark discharge may occur in the case of a rough surface. A single spark discharge was observed during the 5 min microwave treatment of powders of silver, platinum, copper, and copper with carbon. In the cases of copper and copper carbon composition, the observed spark discharge did not result in significant changes in morphology (Figure 3, Figures S1–S8, Figure S201). Noteworthy, the observed spark discharge was a stochastic event, occasionally reproducible for the Cu and Cu/C samples.

**Figure 4:**
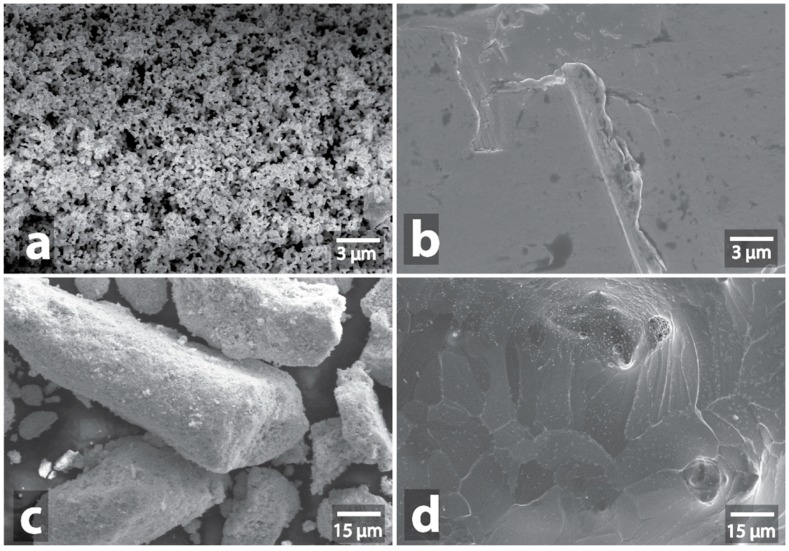
SEM image of initial Ag powder (**a**) and SEM image of Ag powder after MW-treatment (**b**), initial Pt powder (**c**) and SEM image of Pt powder after MW-treatment (**d**).

At the same time, the spark discharge in the case of silver (Figure 4a,b, Figures S17–S24, Figure S203) and platinum (Figure 4c,d, Figures S9–S16, Figure S202) resulted in local fusion, which involved an appreciable part of the silver and platinum powders. The resulting fused aggregate of platinum had a metallic surface and was clearly visible against the background of a black powder that had not been fused. Microscopic examination showed a pronounced domain structure of the fused platinum aggregate, the domain diameter was in the range from 5 μm to 50 μm (Figures S13, S15, S16). Also, an interesting wavy morphology with a period of about 20 nm was noticeable at higher magnifications in some areas of the surface of the fused aggregate, which could be due to the high rate of fusion during the spark discharge (Figure S14). In microscopic images of fused silver aggregates, the domain structure was less noticeable; the surface was hilly, with numerous impregnations of original nanoparticles and polyhedral silver crystallites (Figures S22, S23). XRD data show that the metal phase of silver remains unchanged with no traces of oxidation (Figures S215).

**Figure 5:**
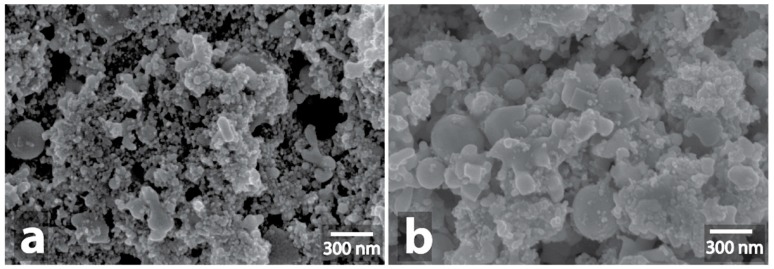
SEM image of initial Re powder (**a**) and SEM image of Re powder after MW-treatment (**b**).

Among other metals, rhenium powders (Re) and the iron carbon composition (Fe/C) demonstrated interaction with microwaves. In the case of rhenium, weak orange sparking was observed. Among the samples with distinctly observable signs of interaction with microwaves, rhenium showed the weakest signs of interaction. In the course of microwave treatment, rhenium powder only slightly changed its color from black to brown. XRD data showed that the main phase in the initial sample was metallic Re, and a small signal of ReO_3_ oxide was also present. After microwaving, the ReO_3_ signal grew much stronger, although the signal corresponding to the unoxidized metal also remained (Figures S216, S217). Subsequent microscopic examination showed no fusion of nanoparticles into larger entities, but the change in the shape of the particles was noticeable: the appearance of polyhedral and cubic particles and an increase in the number of large elongated particles (Figure 5, Figures S25–S30, Figure S204).

**Figure 6:**
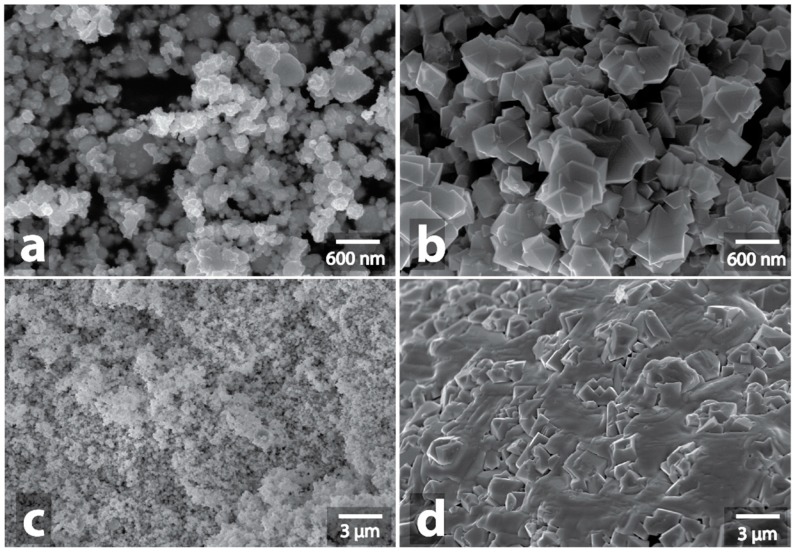
SEM image of initial Fe/C powder (**a**) and SEM image of Fe/C powder after MW-treatment (**b**), initial Mo-Fe-C powder (**c**) and SEM image of Mo-Fe-C powder after MW-treatment (**d**).

Among the metal samples, the most active interaction was observed in the case of iron carbon composition Fe/C (composition contented only 0.37% of carbon). The microwave irradiation resulted in the heating of the sample to a red heat temperature (about 800 °C, according to the pyrometer data); the periodic flashes of plasma and spark discharges were also observed. Significant changes of the Fe/C powder sample morphology were noticeable visually after the treatment: partial melting of the powder produced discrete metal bunches with layers of red iron oxide located between them. In this case, rounded nanoparticles, of which the initial Fe/C powder consisted, were converted into polyhedral particles (Figure 6a,b, Figures S31–S38, Figure S205). Larger particles that appeared as a result of fusion of iron nanoparticles were also found in the form of aggregated polycrystalline formations (Figures S34–S36).

Among the other metal samples, Mo-Fe-C composition powder also exhibited noticeable interactions with microwaves. The heating process of this sample was rather characteristic: no visible manifestations of interactions were detected during the first 30–50 sec of heating. Then, a rapid heating to red heat occurred and lasted until the end of the microwave treatment; as a rule, the heating was preceded by a plasma outburst or a spark discharge. Changes in the Mo-Fe-C sample morphology were similar to those in the Fe/C sample: formation of a metal bunch that separated sections of the orange powder of iron and molybdenum oxides was observed. The formation of particles of a polyhedral shape, and also of large fused irregularly shaped particles, was observed for Mo-Fe-C similarly to the Fe/C sample (Figure 6c,d, Figures S39–S46, Figure S206). As for their specific morphological features, it is worth noting that the large micrometer-sized particles consisted of elongated creases (Figure S44).

**Figure 7:**
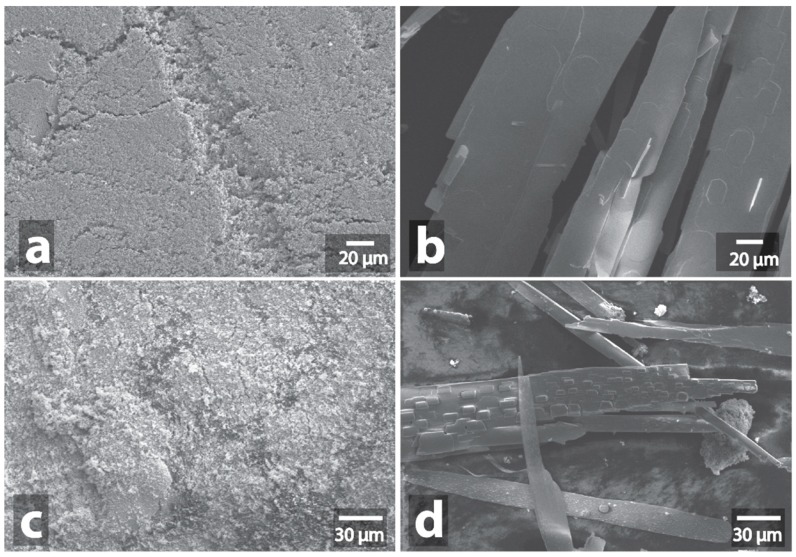
SEM image of initial Mo/C powder (**a**) and SEM image of Mo/C powder after MW-treatment (**b**), initial MoS_2_ powder (**c**) and SEM image of MoS_2_ powder after MW-treatment (**d**).

Molybdenum carbon composition (Mo/C) demonstrated a similar violent reaction under the influence of microwaves (Figure 7a,b, Figures S47–S54, Figure S207). In addition to heating to the temperature of red heat, bright spark discharges, plasma flashes and smoke emissions appeared during the processing. The intensity of heating subsided after 1–2 min of heating, which is probably due to the formation of oxide, which does not absorb microwaves. White oxides crystals, several millimeters long and a few hundred micrometers wide, grew as a result. The images of crystals obtained by SEM showed the presence of small (up to 5 μm) lamellar nuclei on the surface of some of the crystals (Figures S51–S53). Similar crystals grew when MoS_2_ powder was treated with microwaves (Figure 7c,d, Figures S55–S62, Figure S208). The observed crystals represented a mixture of three molybdenum oxides, MoO_2_, MoO_3_, and Mo_4_O_11_, as indicated by the XDR data (Figure S220 The heating process was accompanied by spark discharges, heating of the powder to red heat, and emission of smoke with strong odor.

For other metals and compositions, no strong absorption of microwave radiation accompanied by external manifestations was detected. Perhaps the spark discharge is a probabilistic event for some metal samples, similarly with the copper samples, and in some of these cases a spark discharge may also result in fusion; in any case, it has not been observed under experimental conditions. Interestingly, Fe/C and Mo-Fe-C samples, which have demonstrated the most intense interaction with microwaves, are ferromagnetic materials. At the same time, the samples of Co, Ni, W-Ni-Fe are strong ferromagnets as well; however, no appreciable interaction with the microwaves has been observed for these samples. Alloying of Co, Ni, W-Ni-Fe samples is possible under different conditions (compactified samples, long-term experiment, high microwave power) [28,53], whereas the fusion of other metal samples (W, Mo, Re, W-Cu, inert under the experimental conditions used in this study) can be reportedly achieved by prolonged microwave treatment [54].

Microwave treatment of carbides (WC, TiC) and carbon compositions (W-C, V-C, Cr-C, W-V-C) led to intense heating of the powders to red heat. For WC, TiC, W-V-C and, occasionally, for W-C and V-C, microwave heating was accompanied by periodic spark discharges and plasma flashes. Similar to the case of Mo/C and MoS_2_, a decrease in the intensity of heating was observed for these samples for a few minutes until a complete cessation of heating after 2–3 min of microwave processing, which was associated with the formation of oxides. The heating of silicon carbides (SiC №1 и SiC №2) was much less intense. No external manifestations of interaction with microwaves were observed for these samples, and the temperature increased only to 100 °C within a minute. The microwave-mediated heating of silicon carbide had been investigated in detail previously [55,56].

**Figure 8:**
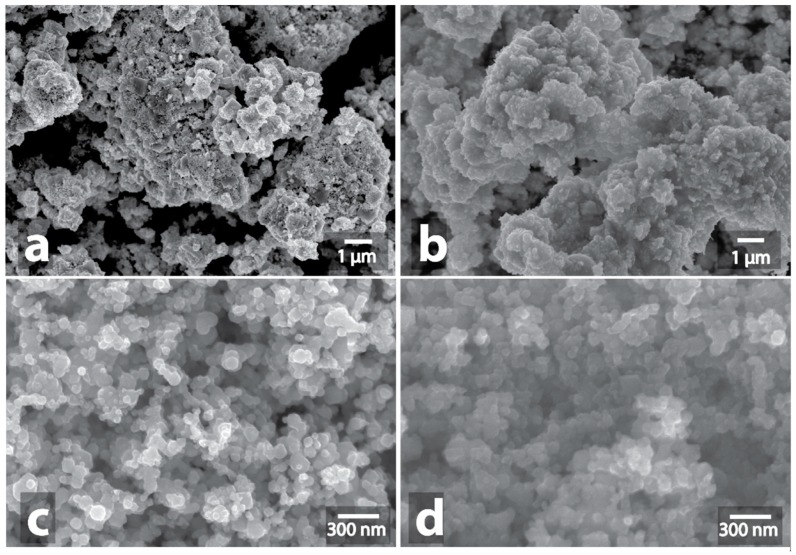
SEM image of initial WC powder (**a**) and SEM image of WC powder after MW-treatment (**b**), initial TiC powder (**c**) and SEM image of TiC powder after MW-treatment (**d**).

The change in color of the powders occurred during the microwave treatment of tungsten carbide and titanium carbide; these changes resulted from rapid oxidation. In the case of WC, the yellow oxide (WO_3_) was formed (Figure S209), and in the case of TiC, the white TiO_2_ was formed (Figure S210). EDX studies have confirmed the formation of oxides (Figures S65, S68, S71, S74). Also, XRD analysis confirmed that the main phase was WO_3_ with an admixture of WC and a slight admixture of metallic tungsten (Figures S218, S219). Microscopic studies showed that large polyhedral particles (up to 1 μm) were present in the initial WC powder; after microwave treatment, almost all of these large particles disappeared, and only the irregularly shaped particles remained (Figure 8a,b, Figures S63–S68). It is interesting that, in the case of TiC, despite the violent reaction during microwave treatment, no morphological changes were detected: the powder consisted of irregular nanoparticles both before and after the microwaving (Figure 8c,d, Figures S69–S74).

**Figure 9:**
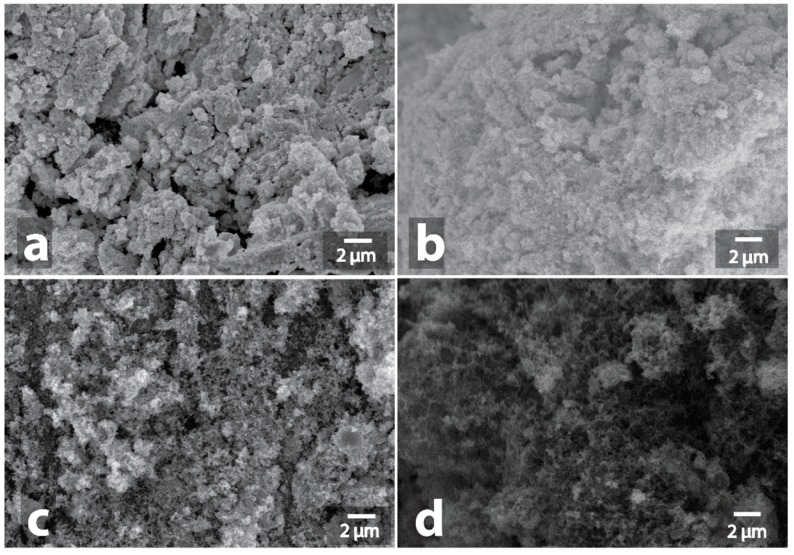
SEM image of initial W-C powder (**a**) and SEM image of W-C powder after MW-treatment (**b**), initial V-C powder (**c**) and SEM image of V-C powder after MW-treatment (**d**).

A similar result was observed in the case of tungsten carbon composition (W-C). Despite the fact that the color was changed from black to yellow during the treatment, as tungsten oxide was formed, no changes in the sample morphology were revealed by SEM examination (Figure 9a,b, Figures S75–S80, Figure S211). At the same time, XRD confirmed the formation of tungsten oxide WO_3_ as the main phase (Figures S221, S222). In the case of vanadium carbon composition (V-C), the color remained black, but a reduction in volume of the powder due to its compaction was substantial. At the same time, no changes in the V-C sample morphology were observed in SEM images (Figure 9c,d, Figures S81–S84, Figure S212).

**Figure 10:**
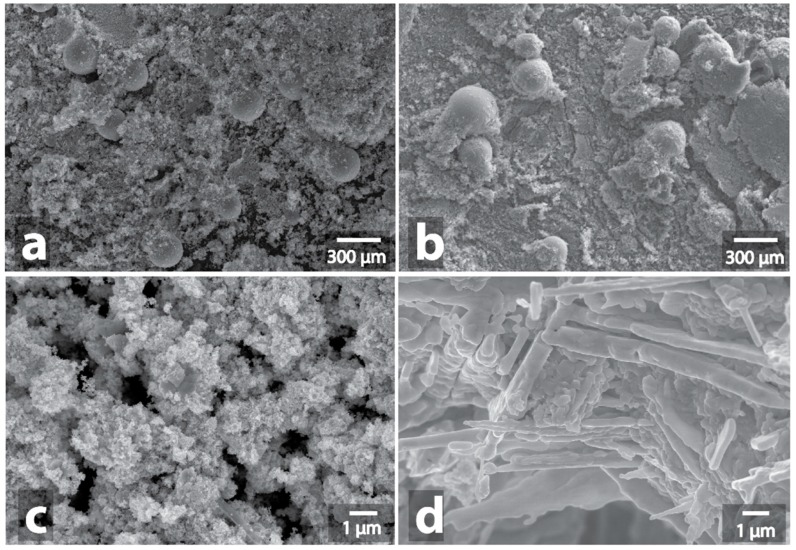
SEM image of initial Cr-C powder (**a**) and SEM image of Cr-C powder after MW-treatment (**b**), initial W-V-C powder (**c**) and SEM image of W-V-C powder after MW-treatment (**d**).

Of all the carbon-containing samples, the reaction of Cr-C was the least violent: only weak red heating was observed. EDX study indicates the formation of oxide (Figure S87, S90). Despite the formation of green chromium oxide Cr_2_O_3_, no morphological changes were detected (Figure 10a,b, Figures S85–S90, Figure S213).

Significant changes in morphology after microwave treatment were detected only in the case of W-V-C sample (Figure 10c,d, Figures S91–S102, Figure S214). Initially, the powder consisted of nanosized irregularly shaped units with an average diameter of about 17 nm. After processing, we observed large micrometer aggregated particles of various shape: needles, rods, globes. At the same time, their distribution was not uniform: the particles of similar shapes often were grouped in segregated bunches. The spherical particles could form clusters like bunches of grapes (Figures S96, S97); in some areas the rods were unidirectional, in other disordered (Figures S95, S99, S100); the needle-shaped particles also could form clusters, in which their growth from one center was observed (Figure S98). During processing, the color of the powder changed from black to orange, apparently as a result of oxidation (Figures S93, S102). XRD showed that in this case, two different phases of WO_3_ oxide arose, and a noticeable signal of metallic tungsten was present after the MW treatment (Figure S223).

In addition to direct microwave heating, an exothermic effect caused by intense oxidation of the powders with atmospheric oxygen contributes to an increase in the temperature for samples that we attributed to type 3 and type 4. Also, EDX and XRD data show a significant increase in oxygen content for this type of sample.

Investigation of the specific surface area using the BET method showed that the specific surface area decreased to a value of a few m^2^/g for every type of sample (except for oxides and weakly absorbing microwave compositions) that was heated in the microwave (Table S4). This indicates the disappearance of the nanoscale structuring of the samples and an increase in the average size of the subunits. The decreasing of the specific surface area for the Re sample and W-C composition was of particular interest; for these samples no significant changes in morphology were observed, but the specific surface area decreased from 6.1 m^2^/g to 0.19 m^2^/g for Re and from 46 m^2^/g to 4.77 m^2^/g in the case of W-C composition.

### 3.2. Changes in the Morphology of Graphite in the Presence of Metal-Containing Substances under the Microwave Treatment Conditions

To study the interaction of powders of metal compounds, model mixtures of graphite with test samples in a ratio of 20:3 were prepared by grinding and mixing in a mortar. The samples prepared in this way were irradiated for 1 minute under the same conditions as the corresponding samples without graphite.

When processing pure graphite, it rapidly heats up to red heat. In the images obtained by SEM, traces of etching on the surface of graphite without metal are almost absent or insignificant (Figures S103 and S104). When processing the model mixtures, for a half of them the treatment is accompanied by heating of the mixture to the red heat temperature, as in the case of pure graphite. But for several model mixtures (Pt/graphite, W-Cu/graphite, W-Ni-Fe/graphite, W-C/graphite, V-C/graphite, Cr-C/graphite, W-V-C/graphite, MoS_2_/graphite), formation of a flame over the irradiated mixture is observed. Spark discharges are additionally observed for Fe_2_O_3_/graphite and WC/graphite samples. As in the case of the initial samples, the process of oxidation of metal-containing particles and graphite with the oxygen of air can give an additional thermal effect. At high temperatures, the oxidation of the components of the mixture occurs fairly quickly and a strong exothermic effect may occur. In addition, such external manifestations of the reaction as the appearance of a flame can obviously be generated by the gaseous products of the interaction with the oxygen: CO_2_, SO_2_ (in the case of MoS_2_).

It is interesting that flame appearance during the microwave treatment of samples with graphite did not depend on the microwave heating intensity of pure powders without graphite. For instance, pure Fe_2_O_3_ was only moderately heated when exposed to microwaves (40 °C in 1 min, 117 °C in 5 min), whereas the same treatment of its mixture with graphite resulted in spark discharges. Conversely, Fe/C, Mo/C, Mo-Fe-C, TiC, which reacted violently to microwave irradiation of their pure powders, produced no similar specific effects when treated in a mixture with graphite.

All samples that underwent microwave treatment were subsequently examined by SEM. Microwave irradiation effects on mixtures of graphite powder with metals or metal compounds are presented in Table S3. The etching of the graphite particles surface, the formation of trenches and pits on graphite sheets were observed for almost all of the samples (Figure 11 and Figures S105–S166). The mixtures of graphite with Fe/C (Figures S113, S114), Mo/C (Figures S125, S126), V-C (Figures S158, S159), W-C (Figures S156, S157), MoS_2_ (Figures S151–S153) where traces of etching were present either in small amounts or absent, were an interesting exception. Another interesting finding was that the intensive surface etching of the graphite particles was also observed for Cu (Figures S117–S120), Cu/C (Figures S121, S122), Ni (Figures S115, S116), Co (Figures S111, S112), W-Cu (Figures S131, S132), Cu-W (Figures S129, S130), W-Ni-Fe (Figures S123, S124), TiN (Figures S140, S141) and even for some oxides such as WO_3_ (Figures S173–S176), ZnO (Figures S177–S180), TiO_2_ (Figures S183, S184), CuO (Figures S191, S192), Fe_2_O_3_ (Figures S187, S188), Y_2_O_3_ (Figures S193, S194), despite that their microwaving without graphite resulted in neither observable heating, nor the intrinsic morphology modification. Only the aluminum, silicon and nitrogen compounds, such as AlN (Figures S136, S137), AlON (Figures S138, S139), Al-B (Figures S154, S155), TiCN (Figures S142, S143), SiC (Figures S147–S150) as well as oxides Al_2_O_3_ (Figures S167–S170), SiO_2_ (Figures S171, S172), ZrO_2_ (Figures S181, S182), CoO (Figures S185, S186), SnO_2_ (Figures S189, S190), MgO (Figures S195, S196), Cr_2_O_3_ (Figures S197, S198), ZrO_2_-SiO_2_ (Figures S199, S200) were inert. No external signs of the reaction, except the red heating of graphite, were observed during their treatment.

Apparently, the heating mechanism in the presence of a carbon support is different: heat transfer from the carbon plays a key role in heating the system. The metal-containing components are likely not to absorb microwaves because of a high degree of dispersion on a graphite surface. This is confirmed by the fact that etching of the carbon surface was observed even at a content of 1% of the metal-containing component, while this amount of sample without graphite did not absorb microwaves. Etching of the carbon surface is specifically determined by chemical and physical properties of the metal species (melting points, solubility of carbon in the melt, the ability to catalyze the interaction of carbon with oxygen in the air, etc.) and not by their belonging to a particular class of compounds (oxides, sulfides, etc.).

## 4. Conclusions

Nanoscale powders of metals and their compounds exhibit variable behaviors under the microwave treatment conditions. Given the apparent complexity and diversity of the mechanisms of interaction of substances with microwaves, the data obtained for various samples can be summarized and divided into 4 different types (Table 1):

- type 1: very weak MW-absorption with a maximum heating up to 200 °C (Al_2_O_3_, SiO_2_, WO_3_, ZnO, ZrO_2_, TiO_2_, CoO, Fe_2_O_3_, SnO_2_, CuO, Y_2_O_3_, MgO, Cr_2_O_3_, ZrO_2_-SiO_2_, Al-B, SiC, AlN, AlON, TiN, TiCN, Cu-W, W-Cu, W-Ni-Fe, Ni, Co);
- type 2: weak MW-absorption or reflection of microwaves, a single spark discharge (Ag, Pt, Cu, Cu/C);
- type 3: moderate MW-absorption, red-colored heat and/or red sparks (Re, W-C, V-C, Cr-C,);
- type 4: intensive MW-absorption, spark discharges, glow of plasma, flame appearance with red-colored heat (Fe/C, Mo/C, Mo-Fe-C, WC, TiC, MoS_2_, W-V-C).

What is interesting to note is that the studied samples of AlN, AlON, TiCN, SiC, Al-B, Al_2_O_3_, SiO_2_, ZrO_2_, CoO, SnO_2_, MgO, Cr_2_O_3_, and ZrO_2_-SiO_2_ showed no observable activity: no significant heating, no morphology changes of initial samples, and no morphology changes of graphite after MW treatment with the samples were observed (these sample were not present in Table 1).

The behavior of the composite particles deserves a few more detailed notes. On one hand, carbon-containing powders (TiC, WC) exhibited good microwave absorption, while silicon carbide showed only weak heating. On the other hand, molybdenum-containing samples demonstrated strong heating during microwave treatment irrespectively of their nature (MoS_2_ and Mo/C). Most metals either did not heat up under the microwave treatment conditions or demonstrate fusion ability with single spark discharges; remarkably, but the microwave treatment of rhenium resulted in continuous heating and orange sparks without the formation of large fused particles.

The responses of a sample to microwaving, e.g. behavior under intensive heating or oxidation of the sample, are not necessarily associated with significant changes in morphology and size of the material particles. Different compounds of the same element, e.g. MoS_2_ and Mo/C, may undergo very similar changes in morphology. In general, the response of a sample to microwaving depends on a number of factors and it is hard to predict based on a single parameter. For instance, the absence of significant changes in W-C and V-C sample morphologies contrasts with dramatic changes and *de novo* formation of a wide variety of particles when the same elements are present in a single nanocomposite.

We have found that the intensity of heating upon irradiation of a pure powder does not always affect the intensity of heating of the powder in the presence of graphite, as well as its ability to interact with graphite particles. Heat transfer from the carbon plays a crucial role in the interaction of compounds with graphite. Etching of carbon materials can be initiated by various classes of metal compounds including oxides. Metal-containing powders that are stable under microwave irradiation in the absence of graphite (Cu, Ni, Co, W-Cu, Cu-W, W-Ni-Fe, TiN, ZnO, WO_3_, TiO_2_, CuO, Fe_2_O_3_, Y_2_O_3_) interact with graphite supports. The interaction results in the formation of trenches and/or pits on the graphite surface. At the same time, intense heating of initial Fe/C, Mo/C, MoS_2_, W-C, V-C samples may cause only a weak interaction with graphite, if any at all.

In each particular case, the sample behavior under the microwave treatment conditions is determined by a combination of different features including chemical nature, particle size, shape, dispersion, electrical, and magnetic properties. All these factors should be taken into account when using microwave irradiation for the preparation of catalysts and when carrying out reactions with microwave heating of the reaction mixture. The present study (see also Supporting Information) provides a systematic analysis of morphological changes of various metal-containing samples under microwave irradiation and serves as a guide for rational assessing of behavior of different materials.

## Figures and Tables

**Figure 11:**
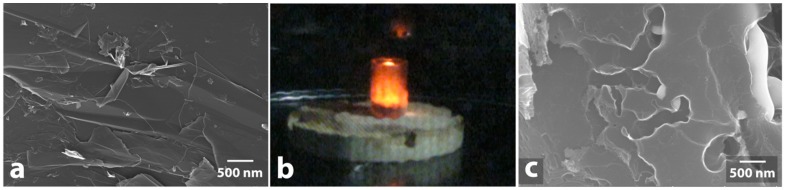
Effect of microwave irradiation on mixture of graphite and metal-containing compounds: initial graphite surface (**a**); microwave heating of mixture of graphite and metal compound (**b**); graphite surface with traces of etching by particles of silver (**c**).

**Table 1:** Effect of microwave irradiation on morphology of metal-containing samples and morphology of graphite after MW-treatment in the presence of metal-containing samples.

Sample	Heating ^a^	Morphology Changes of Initial Samples after MW ^b^	Morphology Changes of Graphite after MW Treatment with the Samples ^c^
Pt	type 2	+	+
Re	type 3	+/-	+
Ag	type 2	+	+
Co	type 1	-	+
Fe/C	type 4	+	-
Ni	type 1	-	+
Cu	type 2	-	+
Cu/C	type 2	-	+
W-Ni-Fe (90-7-3%)	type 1	-	+
Mo/C	type 4	+	-
Mo-Fe-C	type 4	+	+
Cu-W (9:1)	type 1	-	+
W-Cu (1:1)	type 1	-	+
WC	type 4	+/-	+
TiN	type 1	-	+
TiC	type 4	-	+
MoS_2_	type 4	+	-
W-C	type 3	-	+/-
V-C	type 3	-	+/-
Cr-C	type 3	-	+
W-V-C	type 4	+	+
WO_3_	type 1	-	+
ZnO	type 1	-	+/-
TiO_2_	type 1	-	+
Fe_2_O_3_	type 1	-	+/-
CuO	type 1	-	+
Y_2_O_3_	type 1	-	+

^a^ Types of process: type 1 - very weak MW-absorption, heating up to 200 °C; type 2 - weak MW-absorption or reflection of microwaves, a single spark discharge; type 3 - middle MW-absorption, red heat and/or red sparks; type 4 - intensive MW-absorption, spark discharges, glow of plasma, flame appearance with red heat. ^b^ Sign “-” corresponds to the absence of morphology changes; sign “+/-”corresponds to minor changes in particle size or morphology; sign “+”corresponds to significant morphology changes. ^c^ Sign “-” corresponds to the absence of etching traces; sign “+/-”corresponds to slight etching in rare areas; sign “+”corresponds to significant etching, formation of trenches and pits on the surface of graphite.

## Supplementary Materials

The following are available online at http://www.mdpi.com/2079-4991/9/1/19/s1.
