# Supplementary material for: Systematic Study of the Behavior of Different Metal and Metal-Containing Particles under the Microwave Irradiation and Transformation of Nanoscale and Microscale Morphology
Source: Nanomaterials (Basel). 2018 Dec 24;9(1):19. doi: 10.3390/nano9010019 (PMC6359375; doi:10.3390/nano9010019)
Supplement: Supplementary file 1 [file nanomaterials-09-00019-s001.pdf]

Supplementary Materials
for the article

Systematic study of the behavior of different metal and metal-containing particles under the microwave irradiation and transformation of nanoscale and microscale morphology

Evgeniy O. Pentsak[a], Vera A. Cherepanova[a], Mikhail A. Sinayskiy[b], Andrey V. Samokhin[b] and Valentine P. Ananikov[a]*

[a] Zelinsky Institute of Organic Chemistry, Russian Academy of Sciences, Leninsky prospekt 47, Moscow, 119991, Russia;
[b] Baikov Institute of Metallurgy and Material Science (IMET), Russian Academy of Sciences, Leninsky prospekt 49, Moscow 119334, Russia.
E-mail: *val@ioc.ac.ru; http://AnanikovLab.ru*

Table of content

Table S1. Initial samples and their characteristics 5
Table S2. Effect of microwave irradiation on powders of carbides, metals and their compounds 7
Table S3. Effect of microwave irradiation on mixture of graphite powder with metal-contained samples ... 10
Table S4. Measurement of the specific surface of powders by the BET method 13
SEM and EDX investigation of changes in metal compounds under microwave treatment conditions..... 14
SEM images of initial sample of Cu powder 15
SEM images of Cu powder sample after MW treatment 16
SEM images of initial sample of Cu/C powder 17
SEM images of Cu/C powder sample after MW treatment 18
SEM images of initial sample of Pt powder 19
EDX data of initial sample of Pt powder 20
SEM images of Pt powder sample after MW treatment 21
EDX data of Pt powder sample after MW treatment 23
SEM images of initial sample of Ag powder 24
EDX data of initial sample of Ag powder 25
SEM images of Ag powder sample after MW treatment 26
EDX data of Ag powder sample after MW treatment 28
SEM images of initial sample of Re powder 29
EDX data of initial sample of Re powder 30
SEM images of Re powder sample after MW treatment 31
EDX data of Re powder sample after MW treatment 32
SEM images of initial sample of Fe/C powder 33

EDX data of initial sample of Fe/C powder 34
SEM images of Fe/C powder sample after MW treatment..........35
EDX data of Fe/C powder sample after MW treatment 37
SEM images of initial sample of Mo-Fe-C powder 38
EDX data of initial sample of Mo-Fe-C powder 39
SEM images of Mo-Fe-C powder sample after MW treatment 40
EDX data of Mo-Fe-C powder sample after MW treatment 42
SEM images of initial sample of Mo/C powder..........43
EDX data of initial sample of Mo/C powder..........44
SEM images of Mo/C powder sample after MW treatment..........45
EDX data of Mo/C powder sample after MW treatment..........47
SEM images of initial sample of MoS_2 powder 48
EDX data of initial sample of MoS_2 powder 49
SEM images of MoS_2 powder sample after MW treatment 50
EDX data of MoS_2 powder sample after MW treatment 52
SEM images of initial sample of WC powder 53
EDX data of initial sample of WC powder 54
SEM images of WC powder sample after MW treatment 55
EDX data of WC powder sample after MW treatment 56
SEM images of initial sample of TiC powder 57
EDX data of initial sample of TiC powder..........58
SEM images of TiC powder sample after MW treatment 59
EDX data of TiC powder sample after MW treatment..........60
SEM images of initial sample of W-C powder 61
EDX data of initial sample of W-C powder..........62
SEM images of W-C powder sample after MW treatment 63
EDX data of W-C powder sample after MW treatment..........64
SEM images of initial sample of V-C powder 65
SEM images of V-C powder sample after MW treatment 66
SEM images of initial sample of Cr-C powder 67
EDX data of initial sample of Cr-C powder..........68
SEM images of Cr-C powder sample after MW treatment 69
EDX data of Cr-C powder sample after MW treatment..........70
SEM images of initial sample of W-V-C powder..........71
EDX data of initial sample of W-V-C powder..........72
SEM images of W-V-C powder sample after MW treatment..........73

EDX data of W-V-C powder sample after MW treatment........77
Images of changes in the morphology of graphite in the presence of metal compounds under microwave treatment conditions........78
SEM images of morphology of pure graphite after MW treatment........78
SEM images of changes in graphite morphology in the presence of Pt after MW treatment........79
SEM images of changes in graphite morphology in the presence of Re after MW treatment........80
SEM images of changes in graphite morphology in the presence of Ag after MW treatment........81
SEM images of changes in graphite morphology in the presence of Co after MW treatment........82
SEM images of changes in graphite morphology in the presence of Fe/C after MW treatment........83
SEM images of changes in graphite morphology in the presence of Ni after MW treatment........84
SEM images of changes in graphite morphology in the presence of Cu after MW treatment........85
SEM images of changes in graphite morphology in the presence of Cu/C after MW treatment........87
SEM images of changes in graphite morphology in the presence of W-Ni-Fe after MW treatment........88
SEM images of changes in graphite morphology in the presence of Mo/C after MW treatment........89
SEM images of changes in graphite morphology in the presence of Mo-Fe-C after MW treatment........90
SEM images of changes in graphite morphology in the presence of Cu-W after MW treatment........91
SEM images of changes in graphite morphology in the presence of W-Cu after MW treatment........92
SEM images of changes in graphite morphology in the presence of WC after MW treatment........93
EDX data of graphite powder with WC sample after MW treatment........94
SEM images of changes in graphite morphology in the presence of AlN after MW treatment........95
SEM images of changes in graphite morphology in the presence of AlON after MW treatment........96
SEM images of changes in graphite morphology in the presence of TiN after MW treatment........97
SEM images of changes in graphite morphology in the presence of TiCN after MW treatment........98
SEM images of changes in graphite morphology in the presence of TiC after MW treatment........99
EDX data of graphite powder with TiC sample after MW treatment........100
SEM images of changes in graphite morphology in the presence of SiC №1 after MW treatment........101
SEM images of changes in graphite morphology in the presence of SiC №2 after MW treatment........102
SEM images of changes in graphite morphology in the presence of MoS_2 after MW treatment........103
EDX data of graphite powder with MoS_2 sample after MW treatment........104
SEM images of changes in graphite morphology in the presence of Al-B after MW treatment........105
SEM images of changes in graphite morphology in the presence of W-C after MW treatment........106
SEM images of changes in graphite morphology in the presence of V-C after MW treatment........107
SEM images of changes in graphite morphology in the presence of Cr-C after MW treatment........108
EDX data of graphite powder with Cr-C sample after MW treatment........110
SEM images of changes in graphite morphology in the presence of W-V-C after MW treatment........111
SEM images of changes in graphite morphology in the presence of Al_2O_3 after MW treatment........112

SEM images of changes in graphite morphology in the presence of SiO_2 after MW treatment 114
SEM images of changes in graphite morphology in the presence of WO_3 after MW treatment 115
SEM images of changes in graphite morphology in the presence of ZnO after MW treatment 117
SEM images of changes in graphite morphology in the presence of ZrO_2 after MW treatment..............119
SEM images of changes in graphite morphology in the presence of TiO_2 after MW treatment 120
SEM images of changes in graphite morphology in the presence of CoO after MW treatment 121
SEM images of changes in graphite morphology in the presence of Fe_2O_3 after MW treatment............122
SEM images of changes in graphite morphology in the presence of SnO_2 after MW treatment.............123
SEM images of changes in graphite morphology in the presence of CuO after MW treatment 124
SEM images of changes in graphite morphology in the presence of Y_2O_3 after MW treatment..............125
SEM images of changes in graphite morphology in the presence of MgO after MW treatment 126
SEM images of changes in graphite morphology in the presence of Cr_2O_3 after MW treatment 127
SEM images of changes in graphite morphology in the presence of ZrO_2-SiO_2 after MW treatment.......128
SEM images and the macro photographs of samples before and after MW treatment 129
XRD data of samples before and after MW processing 133
XRD diffraction pattern of Ag powder sample after MW treatment 133
XRD diffraction pattern of initial Re powder..134
XRD diffraction pattern of Re powder sample after MW treatment 135
XRD diffraction pattern of initial WC powder 136
XRD diffraction pattern of WC powder sample after MW treatment 137
XRD diffraction pattern of MoS_2 powder sample after MW treatment 138
XRD diffraction pattern of initial W-C powder..139
XRD diffraction pattern of W-C powder sample after MW treatment 140
XRD diffraction pattern of W-V-C powder sample after MW treatment..141

Table S1. Initial samples and their characteristics

Sample	S_{sp}, m^2/g	$D_{BET,}$ nm	Raw material	Element contains, w%		
				[C]	[O]	[N]
Pt	5	56	$Pt(NH_3)_2(NO_2)_2$			
Re	6.1	47	NH_4ReO_4			
Ag	3.8	150	Ag			
Co	12.9	52	$Co(OH)_2$			
Fe/C	5.5	139	$Fe_2O_3 + C_3H_8$	0.37		
Ni	3.8	177	NiO			
Cu	15.4	50	Cu			
Cu/C	17.8	43	$Cu(CH_3COO)_2$	1.74		
W-Ni-Fe (90-7-3%)	4.1	80	WO_3, NiO, Fe_2O_3			
Mo/C	18.3	32	MoO_3	0.22		
Mo-Fe-C	12.9	52	MoO_3:Fe_2O_3 = 1:1	2.7		
Cu-W (9:1)	6.5	98	CuO:WO_3=9:1		2.63	
W-Cu (1:1)	6.5	67	CuO:WO_3=1:1		3.44	
WC	7.5	51	WO_3			
AlN	66.5	28	Al		14.2	25.6
AlON	30	56	Al		37	7.9
TiN	24.7	45	TiH_2		3.4	19.6
TiCN	21.5	-	$TiCl_4$	9.4	5.5	9
TiC	20	61	$TiCl_4$	15.2	1.7	0.13
SiC (1)	25.6	73	$SiCl_4$	27.8		
SiC (2)	82.6	23	$SiCl_4$	34.3		
MoS_2	45.1	27	MoO_3, S	1.42		
Al-B	19.3	109	Al:B=1:2			
W-C	46	8	WO_3	7		
V-C	89	12	V_2O_5	16		
Cr-C	21.8	50	Cr	20.2		
W-V-C	21.4	19	WO_3:V_2O_5=10:1	5.7		
Al_2O_3	69.4	22	Al			
SiO_2	282	8	Schist			
WO_3	4	205	WO_3			
ZnO	20	53	ZnO			
ZrO_2	14.9	68	Zr			
TiO_2	81	18	Ti			
CoO	7.5	150	$Co(OH)_2$			
Fe_2O_3	-	-	Fe			
SnO_2	61.1	14	SnO_2			
CuO	-	-	Cu			
Y_2O_3	8	150	$Y(CH_3COO)_3$			
MgO	60.5	28	Mg			

Cr_2O_3	15.5	74	Cr	
ZrO_2-SiO_2	67	19	$ZrSiO_4$	

Table S2. Effect of microwave irradiation on powders of carbides, metals and their compounds

№	Particles	Type of process	Visual changes	Microscopy study
**1.**	**Pt**<br><br>Micrometer particles of irregular shape (1-100 microns) consisting of spherical particles with a diameter of 10-500 nm (d_{av}=35 nm).	**Type 2**	Fusion	Part of the particles fused into a teardrop particle with a diameter of 500 μm. The domain structure is visible on the particle surface, in some places there is a wavy morphology with a period of about 20 nm.
**2.**	**Ag**<br><br>Micrometer particles of irregular shape (1-100 microns) consisting of rounded particles and irregular shape particles (100-500 nm, d_{av}=157 nm) connected in short chains.	**Type 2**	Fusion	Fusion of the powder with formation a particle of 1-2 mm in size with the domain structure occurred locally. The surface is relatively smooth, covered with nanoparticles (d_{av}=167 nm) and with traces of crystallization of silver.
**3.**	**Cu**<br><br>Rough micrometer particles, consisting of nano-sized subunits (d_{av}=54 nm) of irregular shape.	**Type 2**	No changes	No morphological changes were observed, d_{av}=55 nm.
**4.**	**Re**<br><br>A granular mass consisting of spherical particles with a diameter of 2-600 nm (d_{av}=43 nm).	**Type 3**	Color change	There are no significant changes in morphology. Traces of fusion are noticeable, oblong particles, irregularly shaped particles, polyhedral particles have appeared. At the same time, the particle diameter remained the same (d_{av}=43 nm).
**5.**	**Fe/C**<br><br>A granular mass consisting of spherical particles with a diameter of 5-300 nm (d_{av}=127 nm).	**Type 4**	Color change and fusion	Polycrystalline structures consisting of angular polyhedral particles (d_{av}=427 nm). Also rounded large particles (ca. 500 μm) with a rough surface, formed as a result of fusion and with traces of polyhedral structures, are present.

**6.**	**Cu/C**<br><br>Rough micrometer particles consisting of irregularly shaped subunits with diameters up to 300 nm (d_{av}=60 nm).	**Type 2**	No changes	No morphological changes were observed (d_{av}=71 nm).
**7.**	**WC**<br><br>Rough micrometer particles consisting of angular polyhedral particles with a diameter of 0.2-1 µm, between which irregular nanoparticles are located (d_{av}=28 nm).	**Type 4**	Color change	Micrometer particles with a rough surface. Average diameter of subunits is 58 nm. The polyhedral particles disappeared.
**8.**	**TiC**<br><br>A granular mass consisting of nano-sized irregular shaped subunits (d_{av}=63 nm).	**Type 4**	Color change	No morphological changes were observed. Average diameter of subunits is 61 nm.
**9.**	**W-C**<br><br>Granular mass, consisting of nanoscale subunits (up to 50 nm, d_{av}=9 nm).	**Type 3**	Color change	No morphological changes were observed. Average diameter of subunits is 13 nm.
**10.**	**V-C**<br><br>A granular mass consisting of nano-sized irregular shaped subunits (up to 50 nm, d_{av}=25 nm).	**Type 3**	Compacti on	No morphological changes were observed. Average diameter of subunits is 29 nm.
**11.**	**Cr-C**<br><br>A granular mass consisting of nano-sized irregular shaped subunits (d_{av}=42 nm). Also, spherical particles of regular shape with a diameter of 20-30 µm are observed.	**Type 3**	Color change	No morphological changes were observed. Average diameter of subunits is 40 nm.

**12.**	**W-V-C**<br><br>Micrometer granular particles consisting of an irregular shaped nanoparticle (up to 20 nm, d_{av}=17 nm). Layered inclusions up to 1 micrometer in diameter and needle particles (rarely) were observed.	**Type 4**	Color change	Micrometer particles with a diverse structure: needle crystals (width ca. 1 µm), oblong microparticles (width ca. 300 nm), spherical microparticles (d_{av}=2.5 µm), consisting of rounded particles up to 400 nm in diameter (d_{av}=214 nm), polyhedral particles.
**13.**	**Mo-Fe-C**<br><br>Granular mass consisting of rounded irregularly shaped subunits (d_{av}=50 nm).	**Type 4**	Color change and fusion	Hilly irregularly shaped particles up to 100 microns in diameter with melting traces. Also large millimeter particles with traces of crystallization, polyhedral structures embedded in a smooth surface are present. Agglomerates of nanoparticles are present on the surface of large particles.
**14.**	**Mo/C**<br><br>Granular mass consisting of rounded particles and particles of irregular shape (d_{av}=36 nm) connected in short chains.	**Type4**	Crystals growth	The crystals with several millimeters long and width 300 microns. The surface of the crystals is plate-like. Also, lamellar microcrystals are present. .
**15.**	**MoS_2**<br><br>A granular mass consisting of nano-sized irregular formed subunits (d_{av}=18 nm).	**Type 4**	Crystals growth	The crystals with several millimeters long and 300 microns wide. The surface of the crystals is plate-like. Also, lamellar microcrystals are present.

Types of process.

Type 1) very weak MW-absorption, heating up to 200 °C for Al_2O_3, SiO_2, WO_3, ZnO, ZrO_2, TiO_2, CoO, Fe_2O_3, SnO_2, CuO, Y_2O_3, MgO, Cr_2O_3, ZrO_2-SiO_2, Al-B, SiC, AlN, AlON, TiN, TiCN, Cu-W (9:1), W-Cu (1:1), W-Ni-Fe (90-7-3%), Ni, Co.

Type 2) weak MW-absorption or reflection of microwaves: single spark discharge.

Type 3) middle MW-absorption: red heat and/or red sparks

Type 4) intensive MW-absorption: spark discharges, glow of plasma, flame appearance with red heat.

Table S3. Effect of microwave irradiation on mixture of graphite powder with metal-contained samples

№	Particles	Type of process	Microscopy study
1.	Pt	**The appearance of a flame**	Etching of the graphite surface. The surface of graphite is unevenly covered with rounded particles.
2.	Re	**Red heat of graphite**	Etching of the graphite surface. The surface of graphite is unevenly covered with rounded particles.
3.	Ag	**Red heat of graphite**	Etching of the graphite surface. The surface of graphite is unevenly covered with rounded particles.
4.	Co	**Red heat of graphite**	Etching of the graphite surface. The surface of graphite is unevenly covered with rounded particles.
5.	Fe/C	**Red heat of graphite**	A slight etching of the surface of graphite. Single particles of compound on the surface of graphite.
6.	Ni	**Red heat of graphite**	Etching of the graphite surface. The surface of graphite is unevenly covered with rounded particles.
7.	Cu	**Red heat of graphite**	Etching of the graphite surface. The surface of graphite is unevenly covered with rounded particles.
8.	Cu/C	**The appearance of a flame**	Etching of the graphite surface. The surface of graphite is unevenly covered with rounded particles.
9.	W-Ni-Fe	**The appearance of a flame**	Etching of the graphite surface. The surface of graphite is unevenly covered with rounded and polyhedral particles.
10.	Mo/C	**Red heat of graphite**	Single particles of the compound and agglomerates on the graphite surface.
11.	Mo-Fe-C	**Red heat of graphite**	Etching of the graphite surface. The surface of graphite is unevenly covered with rounded particles.
12.	Cu-W	**Red heat of graphite**	Etching of the graphite surface. The surface of graphite is unevenly covered with rounded particles.
13.	W-Cu	**The appearance of a flame**	Etching of the graphite surface. The surface of graphite is unevenly covered with rounded particles.
14.	WC	**Red heat of graphite, rare spark discharges**	Etching of the graphite surface. The graphite surface is covered with rounded and needle particles of

			nanometer and micrometer size.
15.	AlN	**Red heat of graphite**	Single particles of compound on the surface of graphite.
16.	AlON	**Red heat of graphite**	Single particles of compound on the surface of graphite.
17.	TiN	**Red heat of graphite**	Etching of the graphite surface. Single particles of compound on the surface of graphite.
18.	TiCN	**Red heat of graphite**	Single particles of compound on the surface of graphite.
19.	TiC	**Red heat of graphite**	Etching of the graphite surface. The surface of graphite is unevenly covered with rounded particles and their agglomerates.
20.	SiC (1)	**Red heat of graphite**	The surface of graphite is unevenly covered agglomerates.
21.	SiC (2)	**Red heat of graphite**	The surface of graphite is unevenly covered agglomerates.
22.	MoS_2	**The appearance of a flame**	Plate crystals on the surface of graphite.
23.	Al-B	**Red heat of graphite**	Single particles of compound on the surface of graphite.
24.	W-C	**The appearance of a flame**	Slight etching in rare areas. The surface of graphite is unevenly covered with rounded particles and their agglomerates.
25.	V-C	**The appearance of a flame**	Slight etching in rare areas. The surface of graphite is unevenly covered with rounded particles and their agglomerates.
26.	Cr-C	**The appearance of a flame**	Etching of the graphite surface. The surface of graphite is coated with particles of 10-200 nm in size.
27.	W-V-C	**The appearance of a flame**	Etching of the graphite surface. The graphite surface is covered with rounded and needle particles of nanometer and micrometer size.
28.	Al_2O_3	**Red heat of graphite**	Single particles of compound on the surface of graphite.

29.	SiO_2	**Red heat of graphite**	Single particles of the compound and agglomerates on the graphite surface. Part of substance is sublimated on quartz vial.
30.	WO_3	**Red heat of graphite**	Etching of the graphite surface. The surface of graphite is unevenly covered with rounded particles.
31.	ZnO	**Red heat of graphite**	Etching of the graphite surface. The graphite surface is covered with rounded and plate crystals.
32.	ZrO_2	**Red heat of graphite**	Single particles of compound on the surface of graphite.
33.	TiO_2	**Red heat of graphite**	The surface of graphite is unevenly covered with rounded particles and their agglomerates.
34.	CoO	**Red heat of graphite**	Single particles of compound on the surface of graphite.
35.	Fe_2O_3	**The appearance of a flame, spark discharges**	Etching on individual areas of the graphite surface. Single particles of compound on the surface of graphite.
36.	SnO_2	**Red heat of graphite**	The surface of graphite is unevenly covered with rounded particles.
37.	CuO	**Red heat of graphite**	Etching of the graphite surface. The surface of graphite is unevenly covered with polyhedral particles.
38.	Y_2O_3	**Red heat of graphite**	Etching of the graphite surface. The surface of graphite in rare areas is unevenly covered with filament particles.
39.	MgO	**Red heat of graphite**	Single particles of compound on the surface of graphite.
40.	Cr_2O_3	**Red heat of graphite**	Single particles of compound and agglomerates on the surface of graphite.
41.	ZrO_2-SiO_2	**Red heat of graphite**	The surface of graphite is covered with rounded particles and their agglomerates.

Table S4. Measurement of the specific surface of powders by the BET method

Particles	Specific surface area, S_{sp}, m^2/g	
	Initial sample	Sample after MW treatment
**Ag**	3.8	~0
**Re**	6.1	0.19
**W-V-C**	21.4	1.74
**MoS_2**	45.1	0.44
**W-C**	46	4.77
**WC**	7.5	2.31

SEM and EDX investigation of changes in metal compounds under microwave treatment conditions

A target-oriented approach was utilized for optimization of the analytic measurements. Before measurements, the samples were placed on a 25 mm aluminum specimen stub and fixed by conductive graphite adhesive tape. Sample morphology was studied under native conditions to exclude the metal coating surface effects. The observations were carried out using a Hitachi SU8000 field-emission scanning electron microscope (FE-SEM). The images were acquired in a secondary electron mode at a 2-30 kV accelerating voltage and at working distances of 8-10 mm.

EDX studies were carried out using an Oxford Instruments X-max EDX system. The increased content of carbon and oxygen in the EDX analysis can be resulted from their presence in the carbon adhesive tape and specimen stub which used to fix samples for SEM study.

SEM images of initial sample of Cu powder

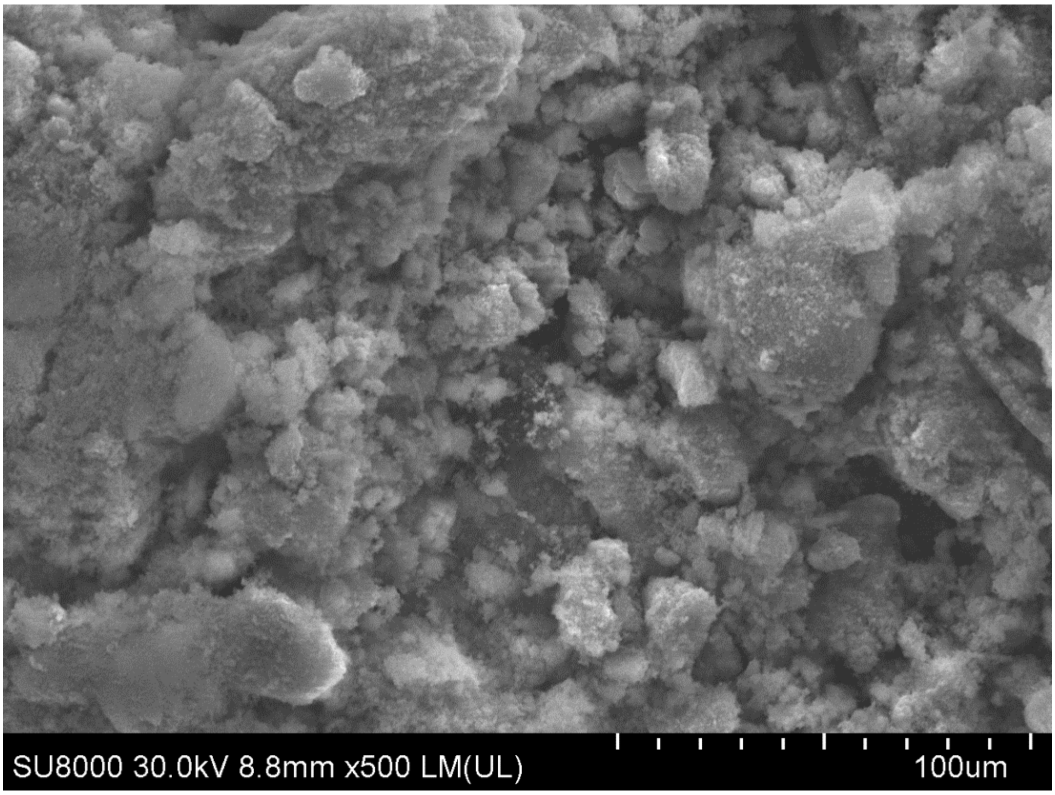

Figure S1. SEM image of initial sample of Cu powder.

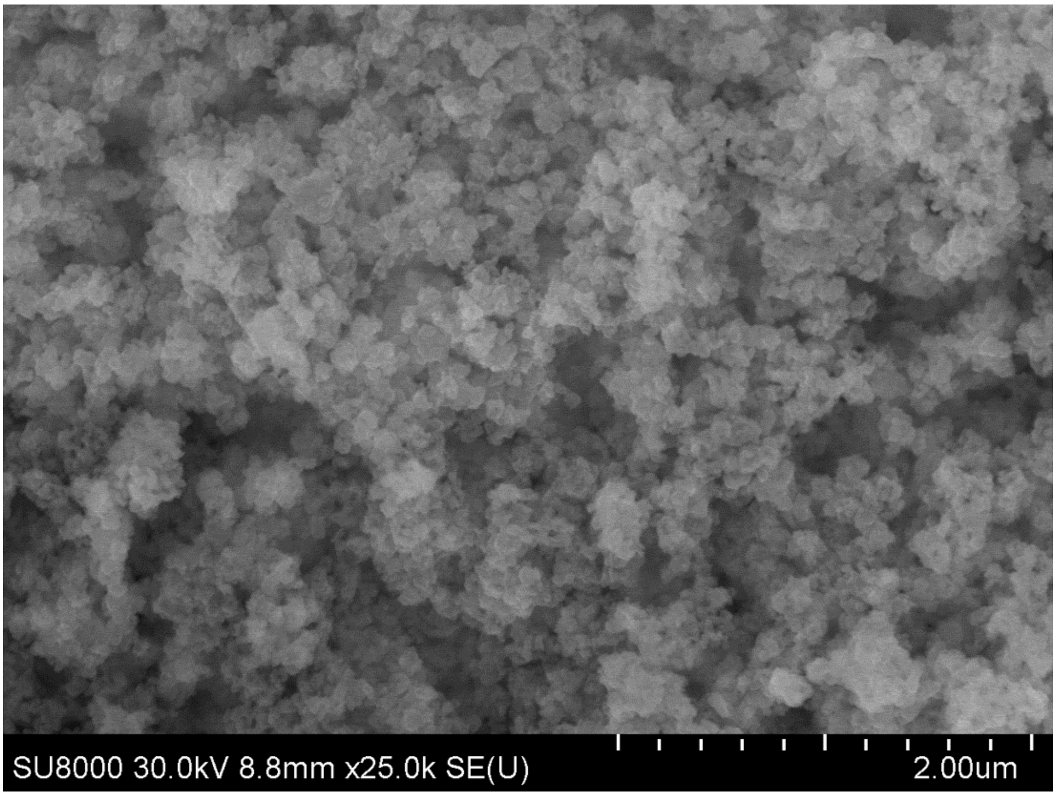

Figure S2. SEM image of initial sample of Cu powder.

SEM images of Cu powder sample after MW treatment

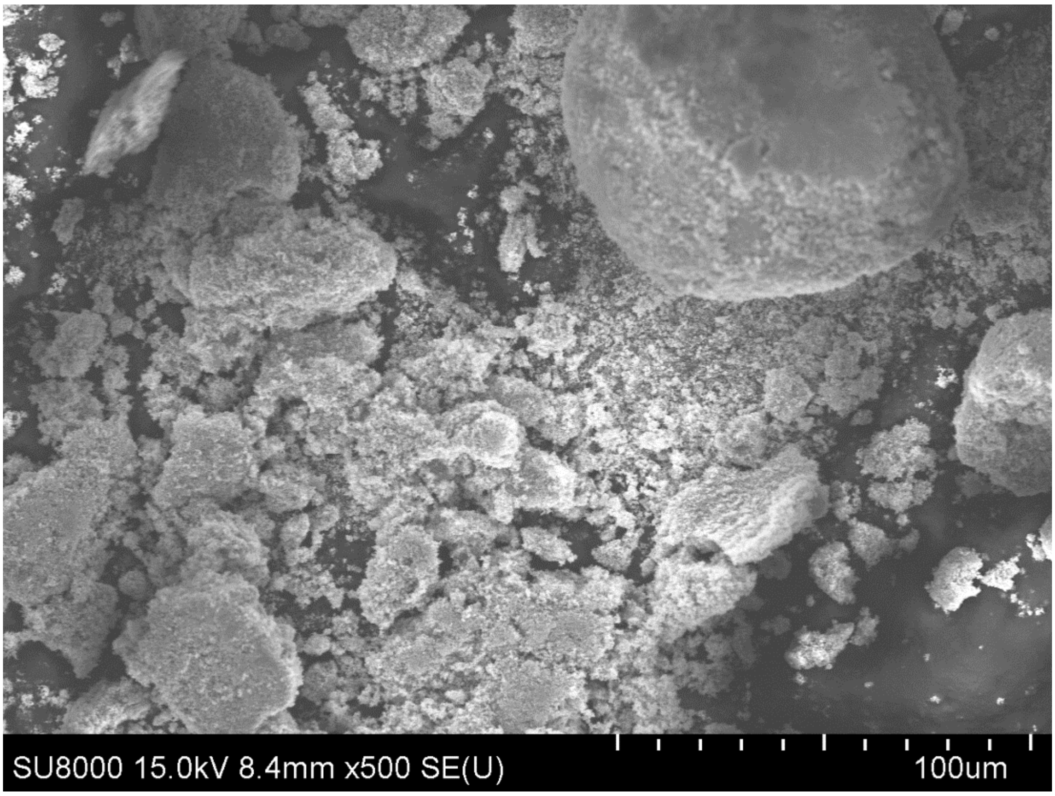

Figure S3. SEM image of Cu powder sample after MW treatment.

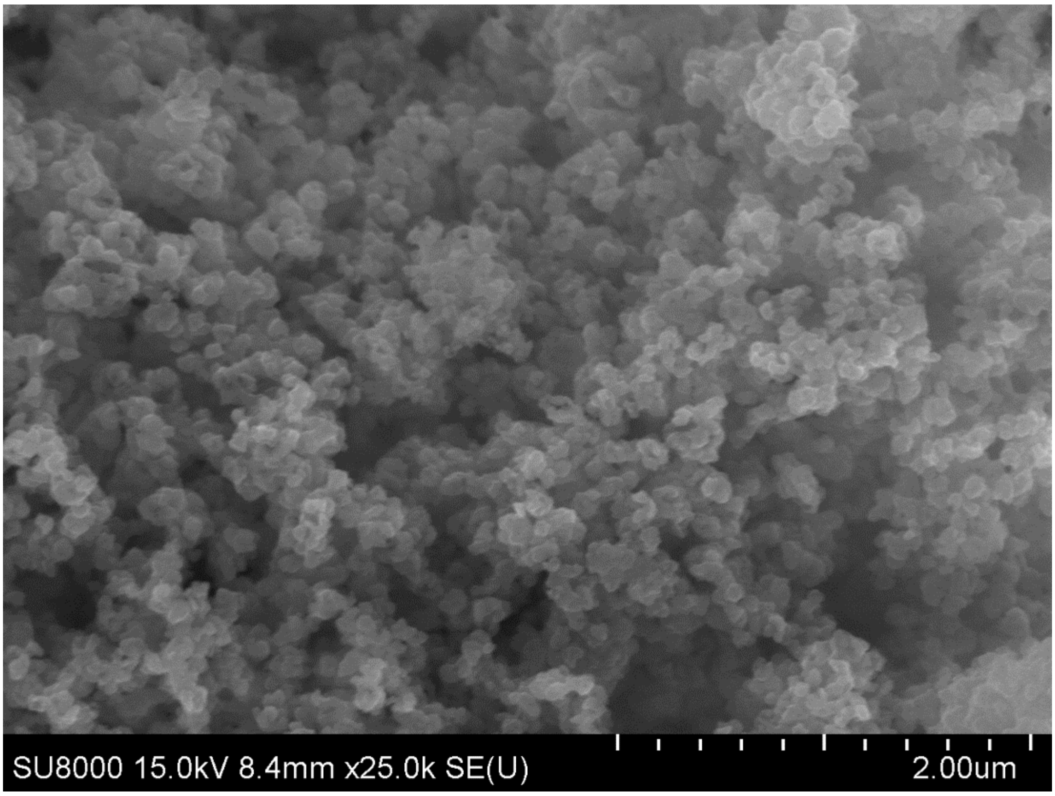

Figure S4. SEM image of Cu powder sample after MW treatment.

SEM images of initial sample of Cu/C powder

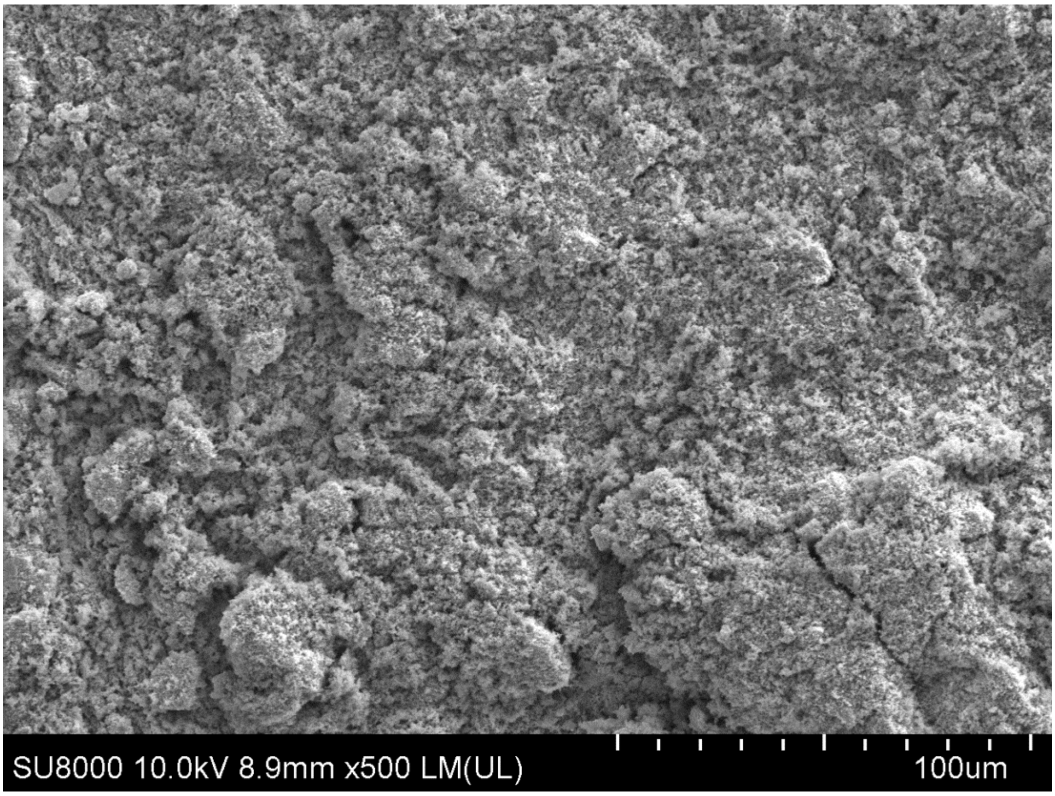

Figure S5. SEM image of initial sample of Cu/C powder.

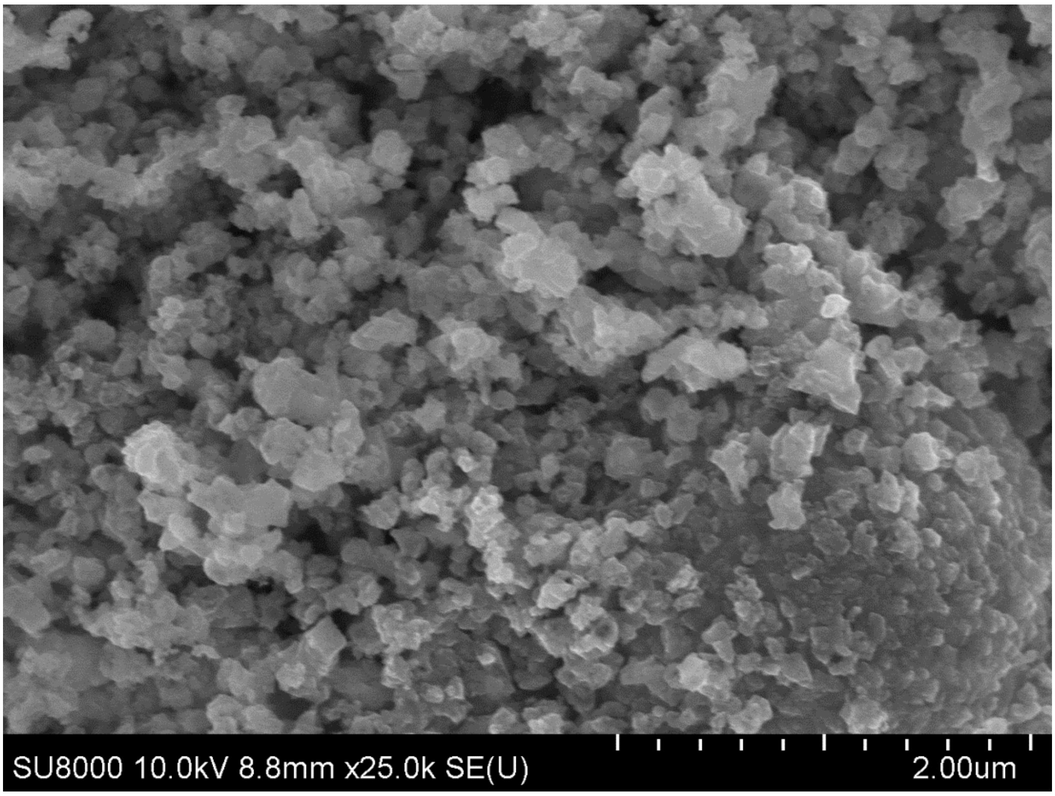

Figure S6. SEM image of initial sample of Cu/C powder.

SEM images of Cu/C powder sample after MW treatment

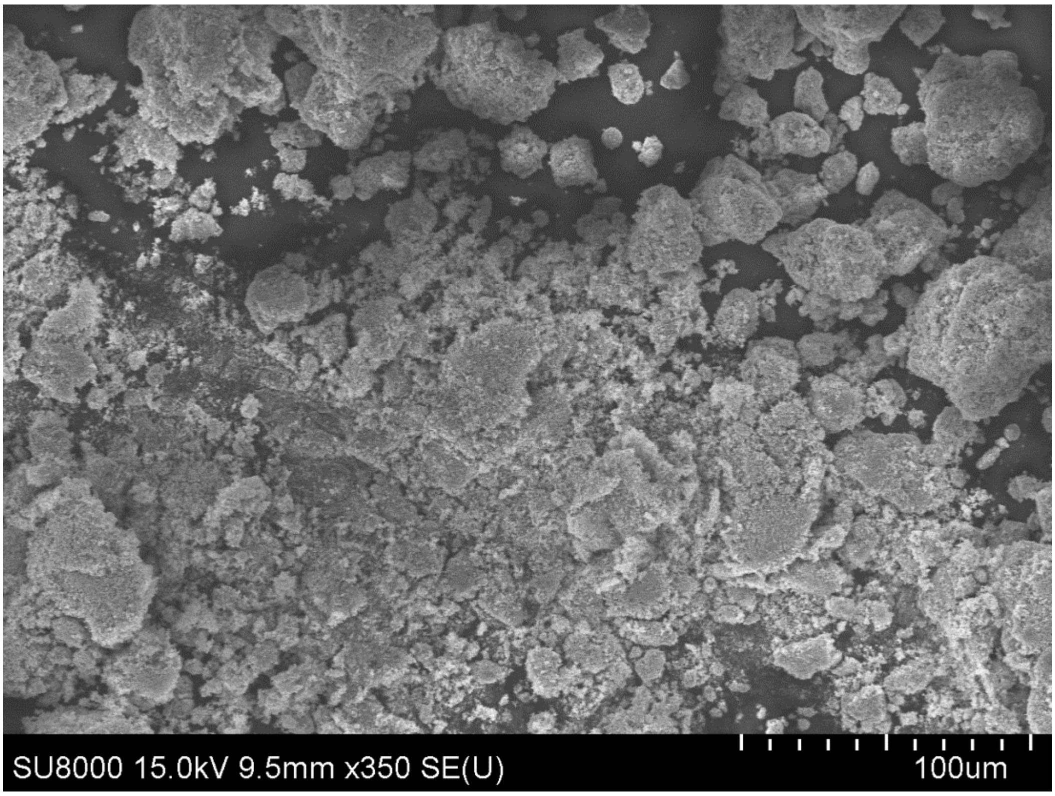

Figure S7. SEM image of Cu/C powder sample after MW treatment.

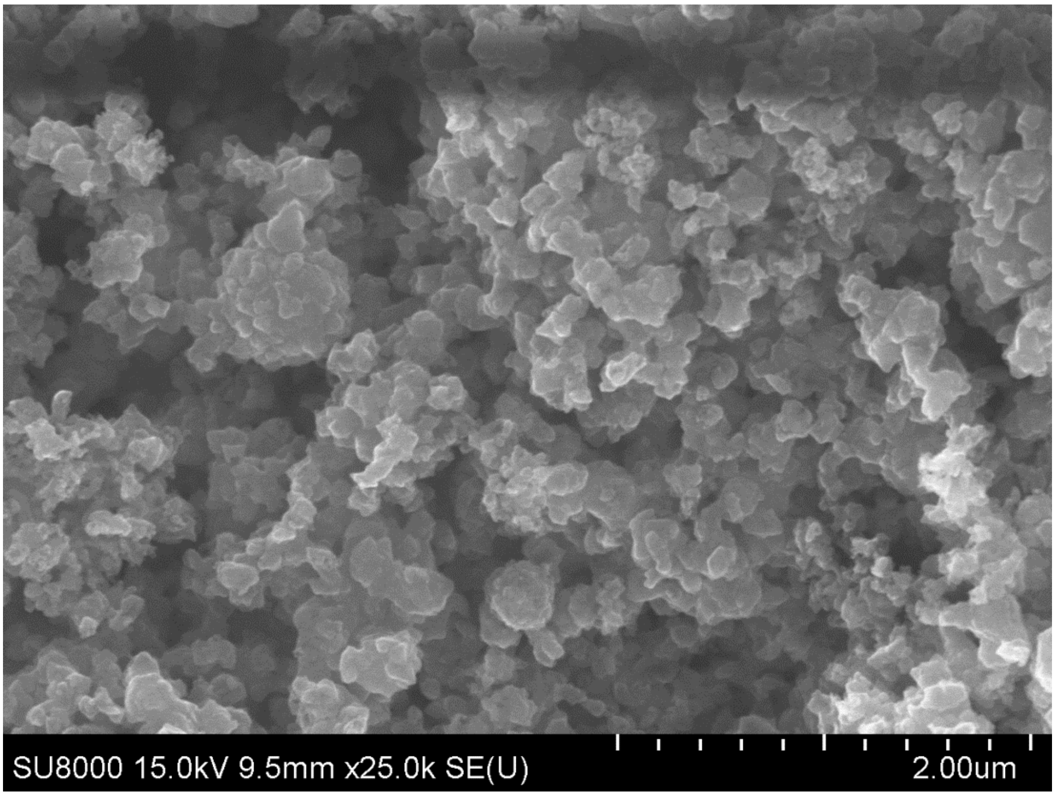

Figure S8. SEM image of Cu/C powder sample after MW treatment.

SEM images of initial sample of Pt powder

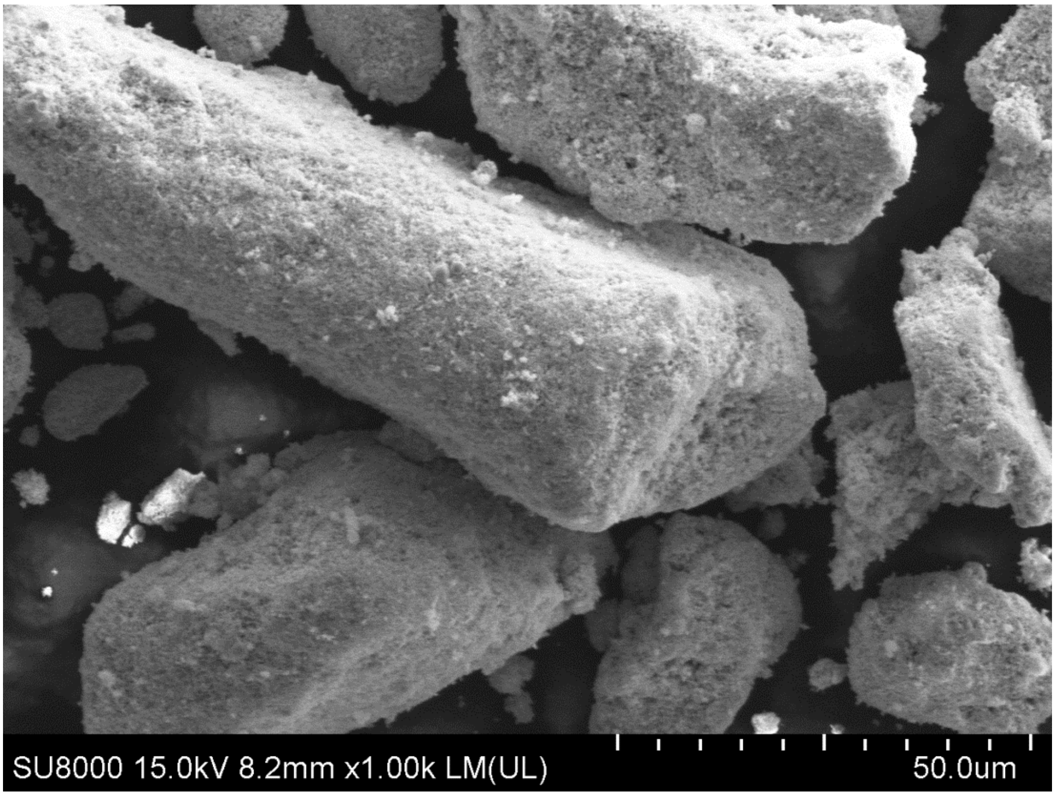

Figure S9. SEM image of initial sample of Pt powder.

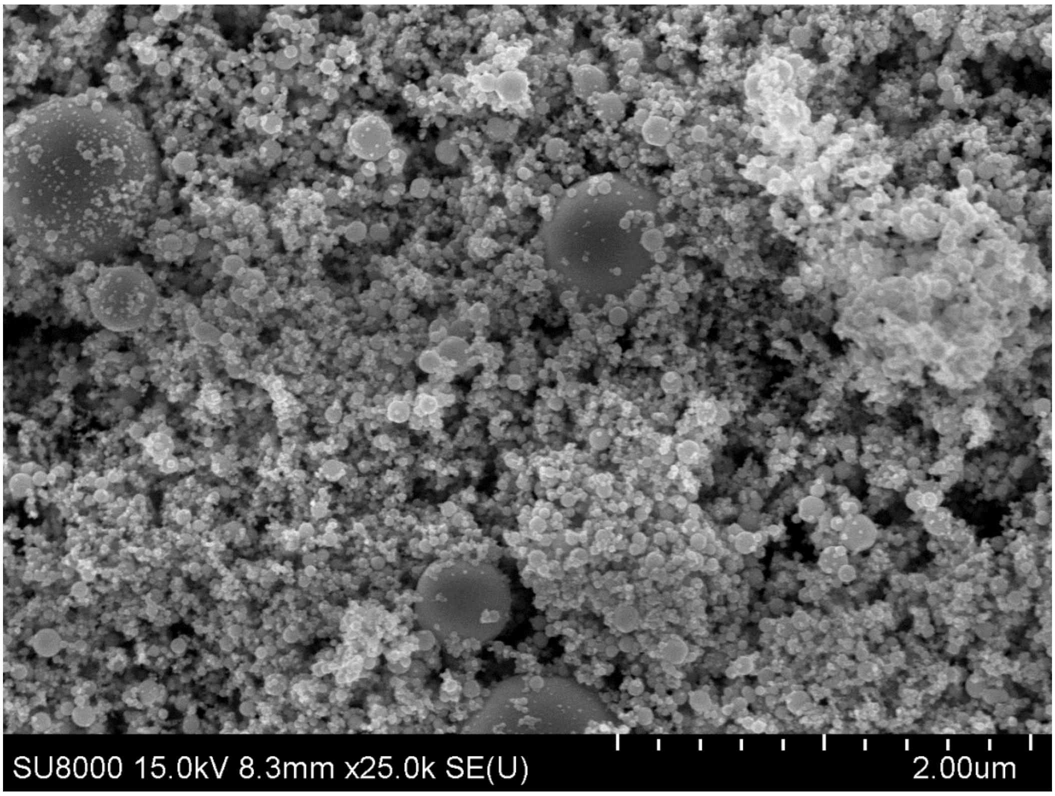

Figure S10. SEM image of initial sample of Pt powder.

EDX data of initial sample of Pt powder

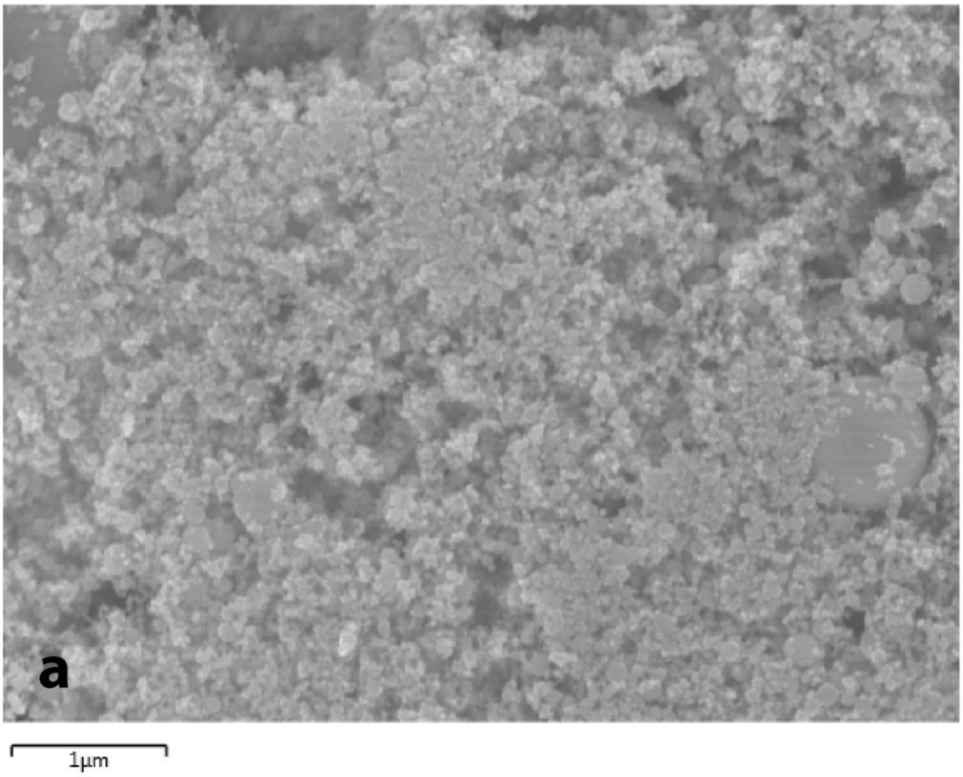

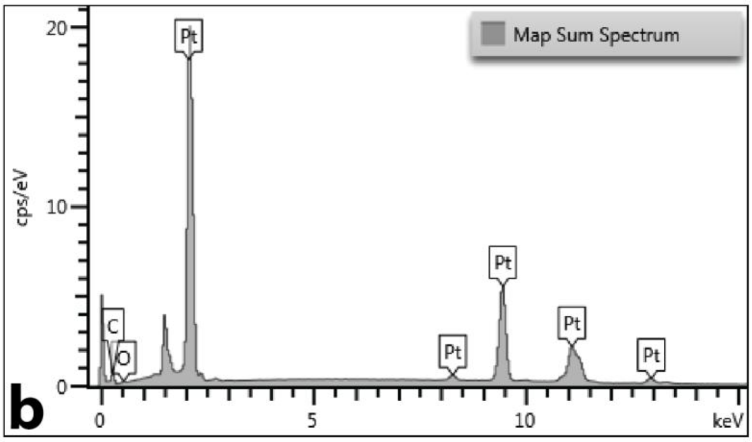

c

Element	Wt%	At%
Pt	86.1	27.9
C	12.9	68.1
O	1.0	4.0
Total:	100	100

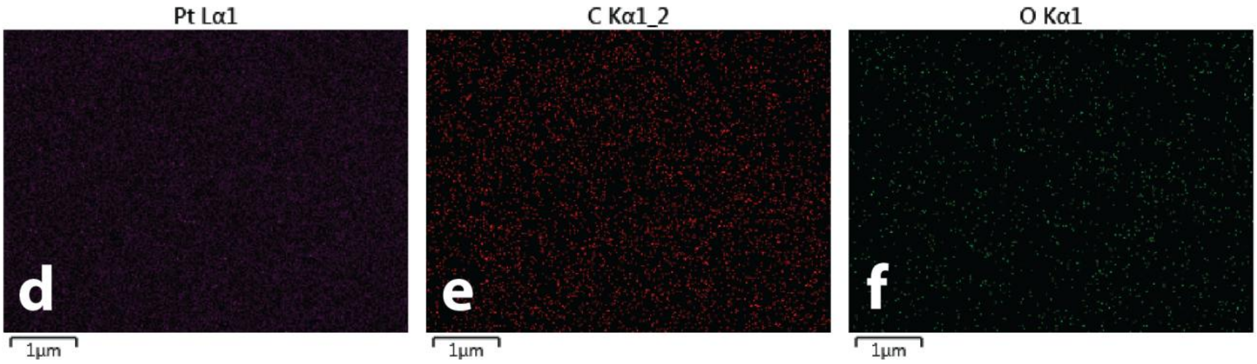

Figure S11. EDX study of initial Pt sample: SEM image (a); EDX spectrum of this area (b); element composition (c) and maps of platinum (d), carbon (e), and oxygen (f) distributions. The original nanopowder does not contain carbon. The presence of carbon and the increased oxygen content in the EDX analysis result by their presence in the carbon tape and stub which fixed samples for SEM.

SEM images of Pt powder sample after MW treatment

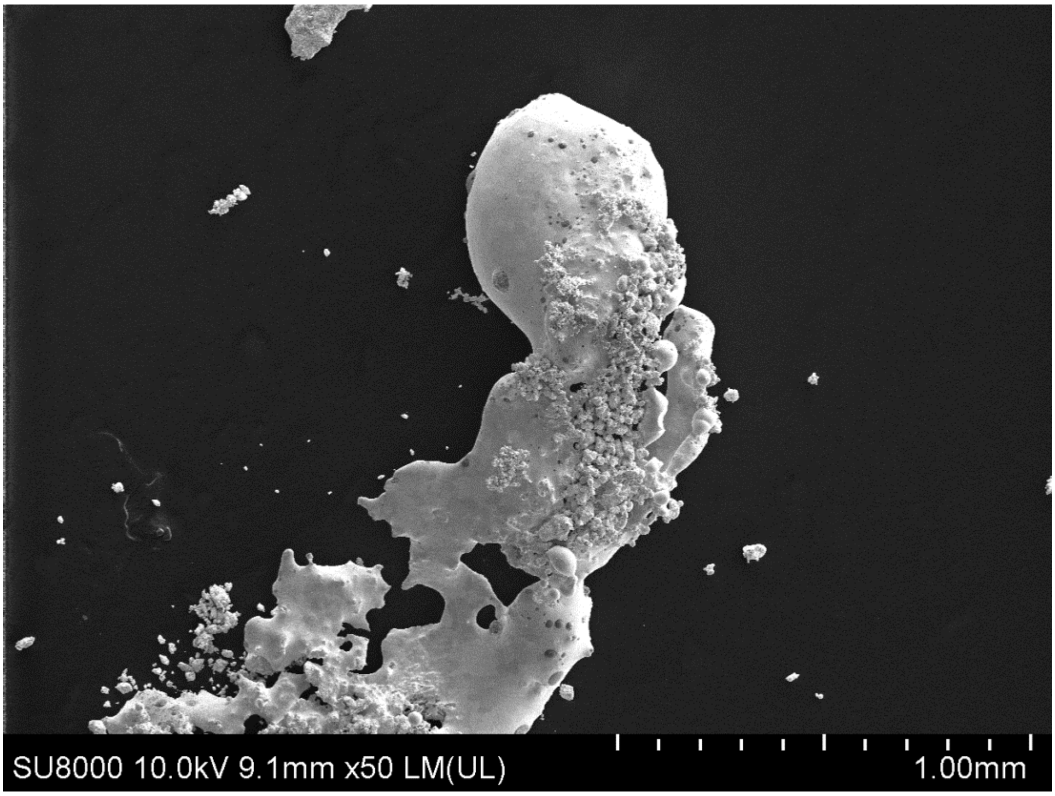

Figure S12. SEM image of Pt powder sample after MW treatment.

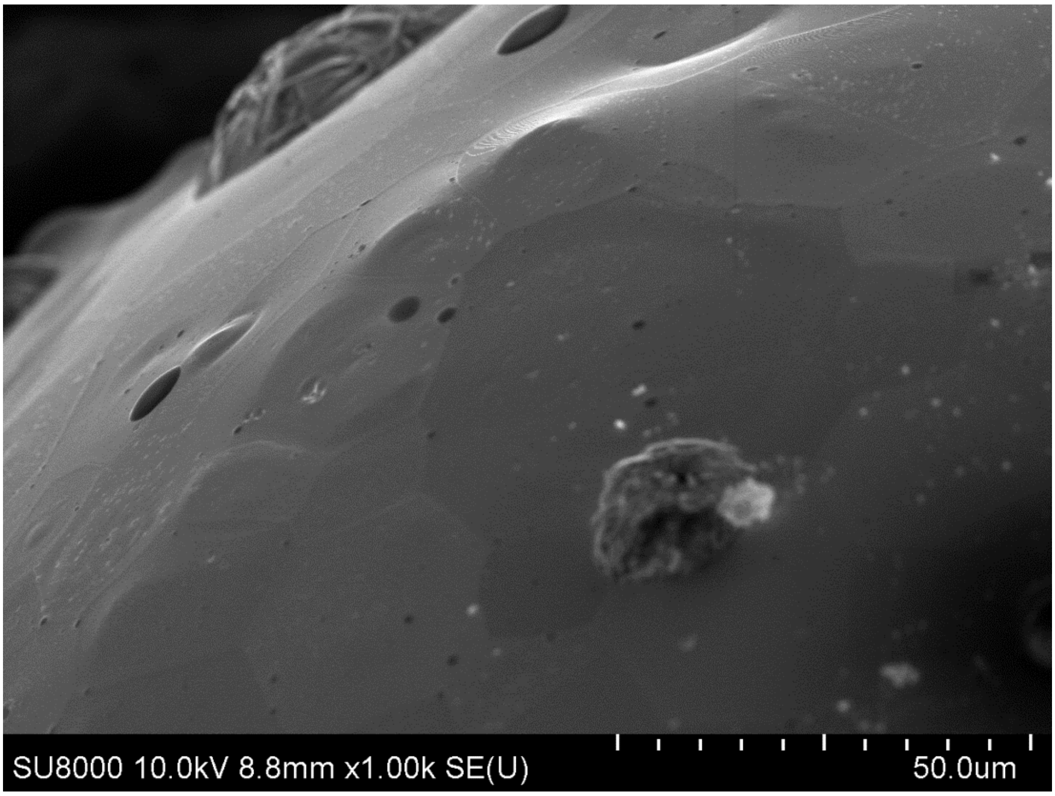

Figure S13. SEM image of Pt powder sample after MW treatment.

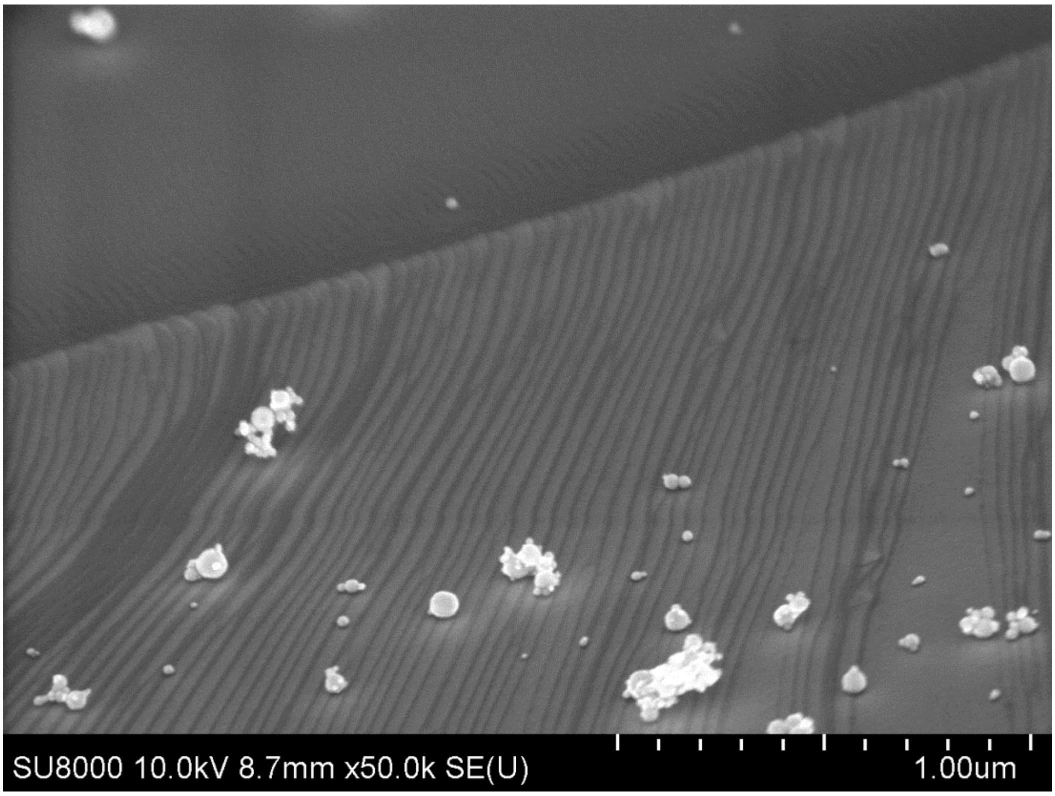

Figure S14. SEM image of Pt powder sample after MW treatment.

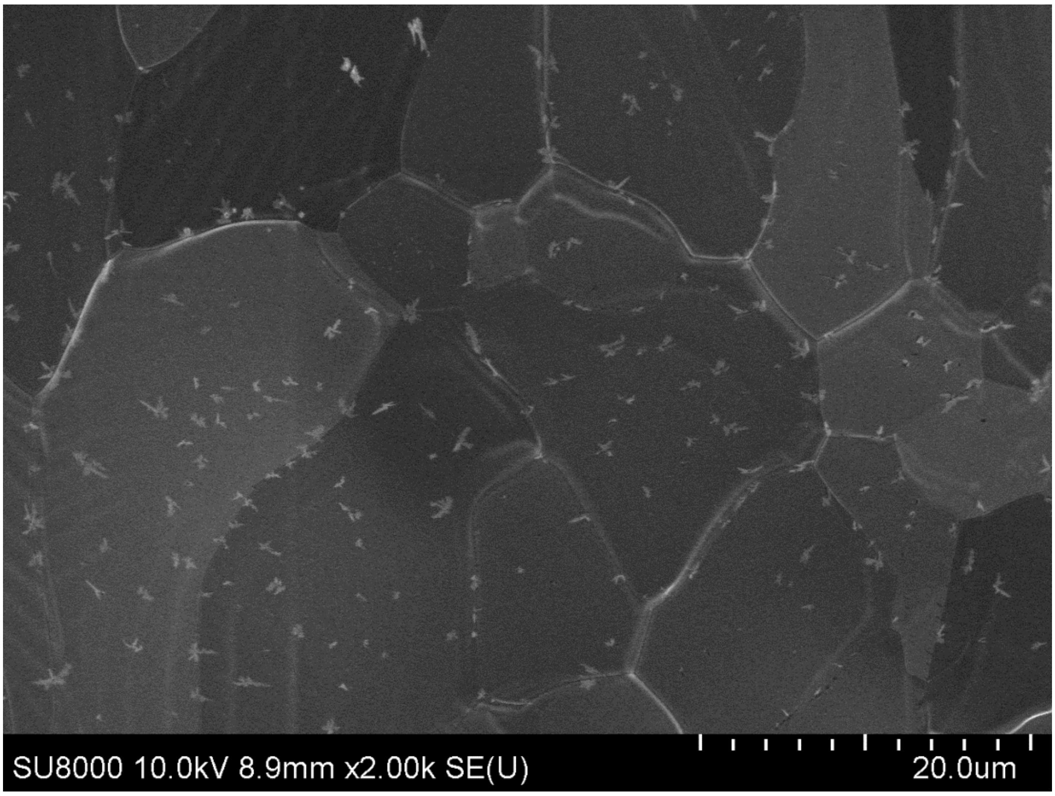

Figure S15. SEM image of Pt powder sample after MW treatment.

EDX data of Pt powder sample after MW treatment

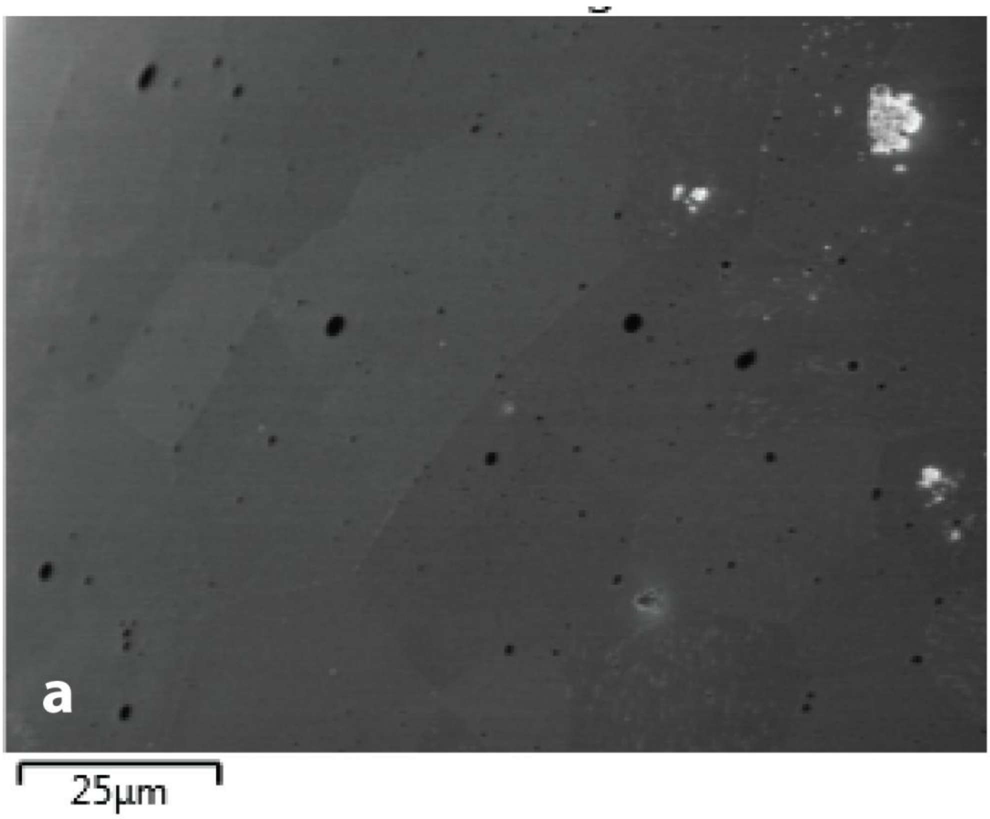

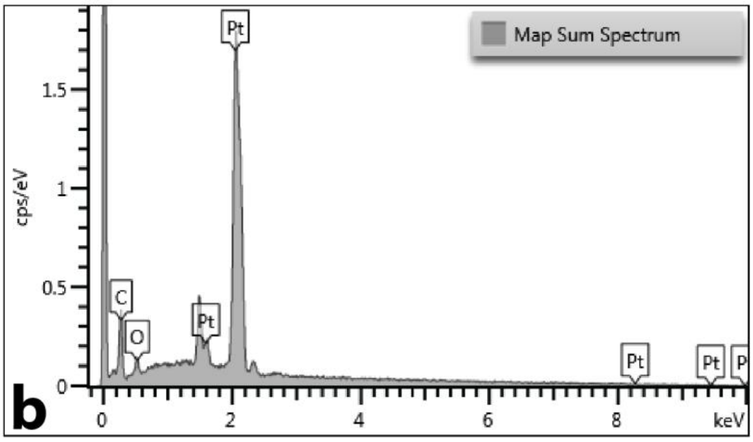

c

Element	Wt%	At%
Pt	86.5	29.0
C	11.7	63.8
O	1.8	7.2
Total:	100	100

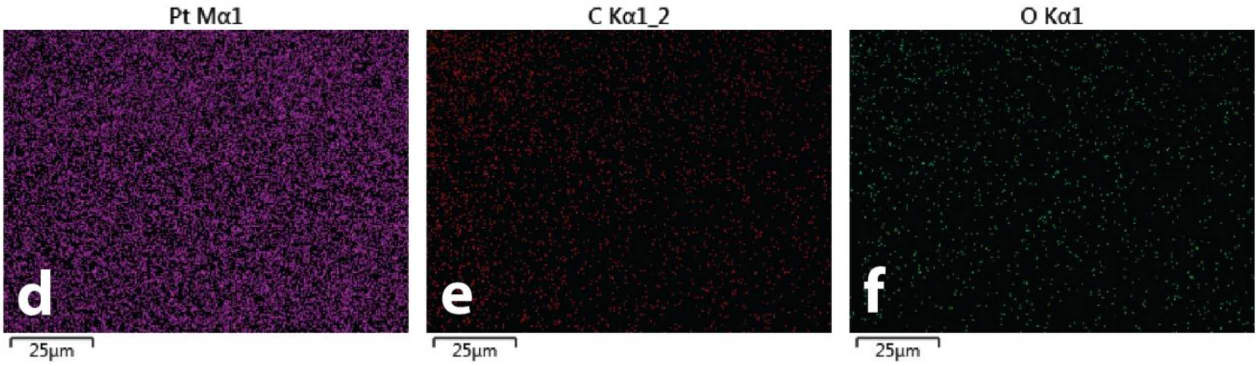

Figure S16. EDX study of Pt powder after MW treatment: SEM image (a); EDX spectrum of this area (b); element composition (c) and maps of platinum (d), carbon (e), and oxygen (f) distributions.

SEM images of initial sample of Ag powder

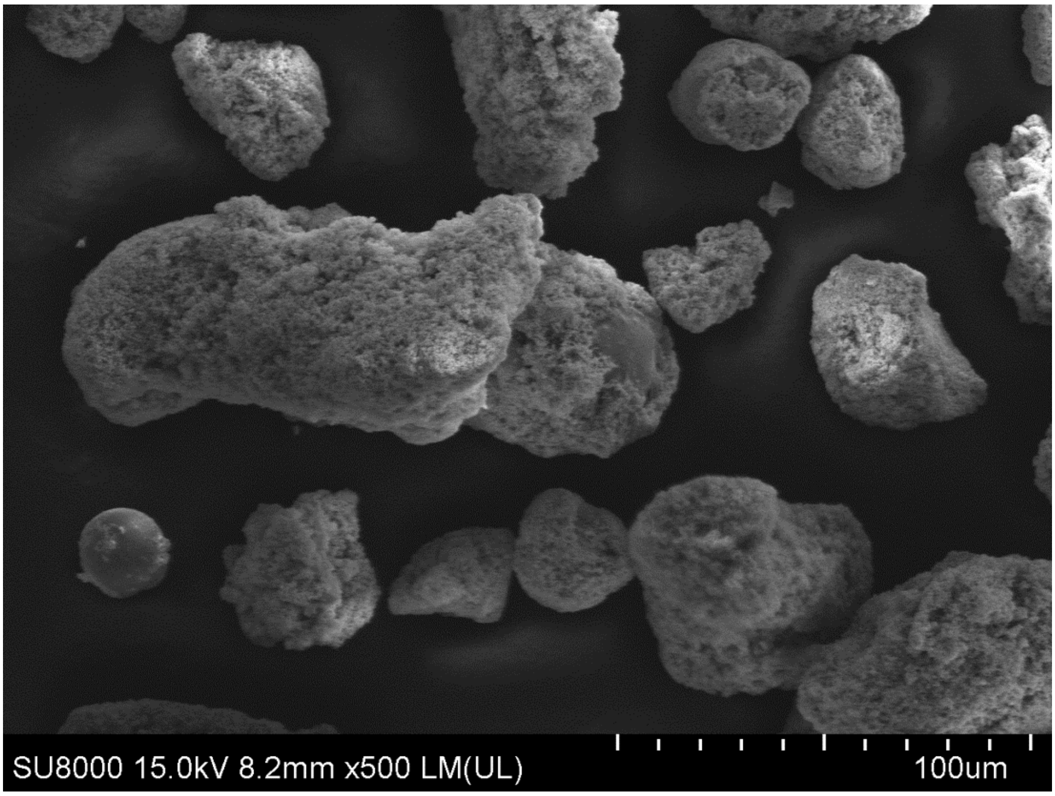

Figure S17. SEM image of initial sample of Ag powder.

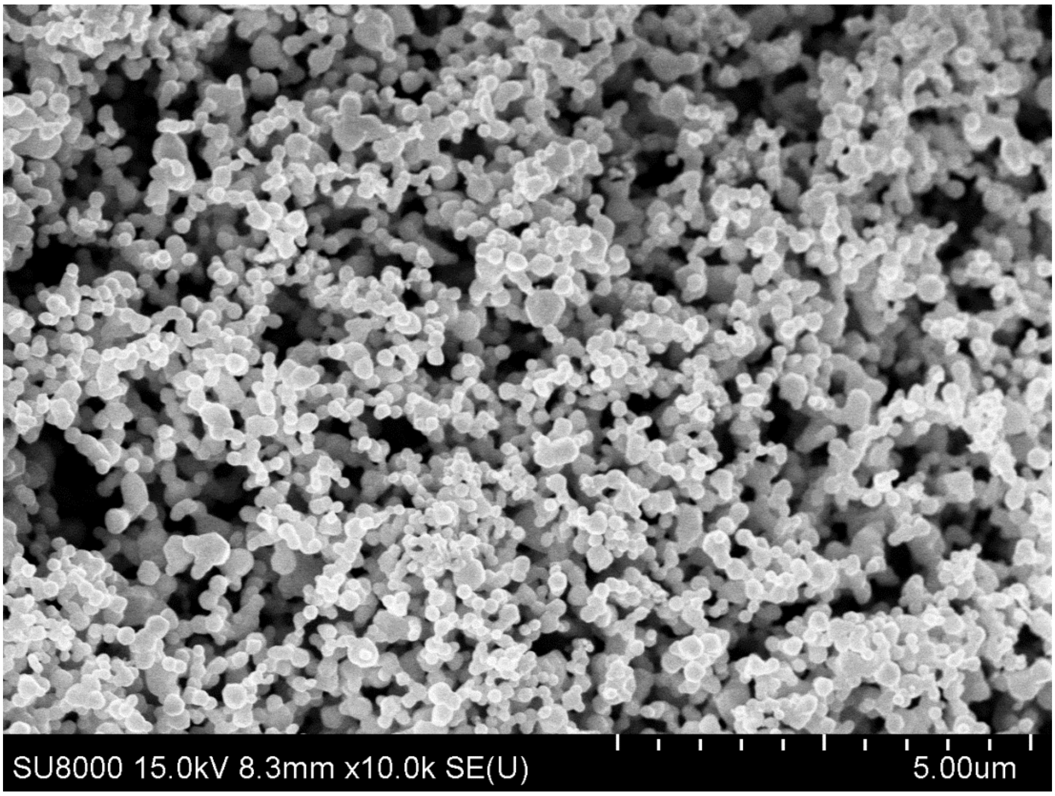

Figure S18. SEM image of initial sample of Ag powder.

EDX data of initial sample of Ag powder

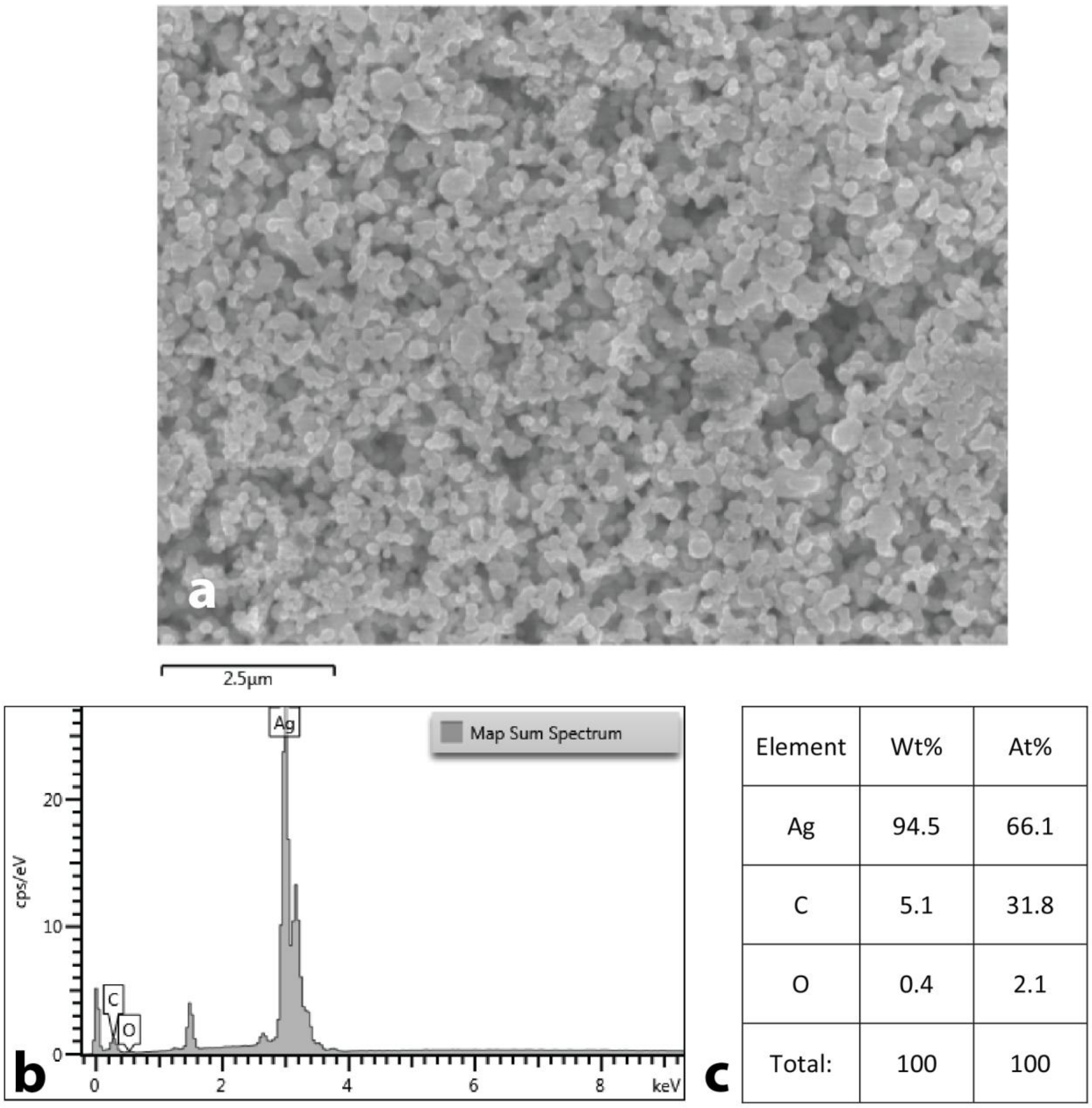

c

Element	Wt%	At%
Ag	94.5	66.1
C	5.1	31.8
O	0.4	2.1
Total:	100	100

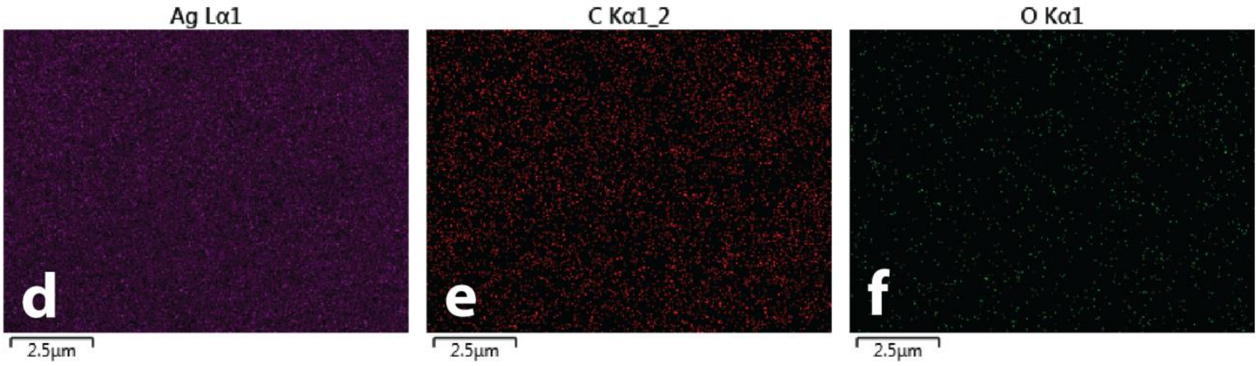

Figure S19. EDX study of initial Ag sample: SEM image (a); EDX spectrum of this area (b); element composition (c) and maps of silver (d), carbon (e), and oxygen (f) distributions. The original nanopowder does not contain carbon. The presence of carbon and the increased oxygen content in the EDX analysis result by their presence in the carbon tape and stub which fixed samples for SEM.

SEM images of Ag powder sample after MW treatment

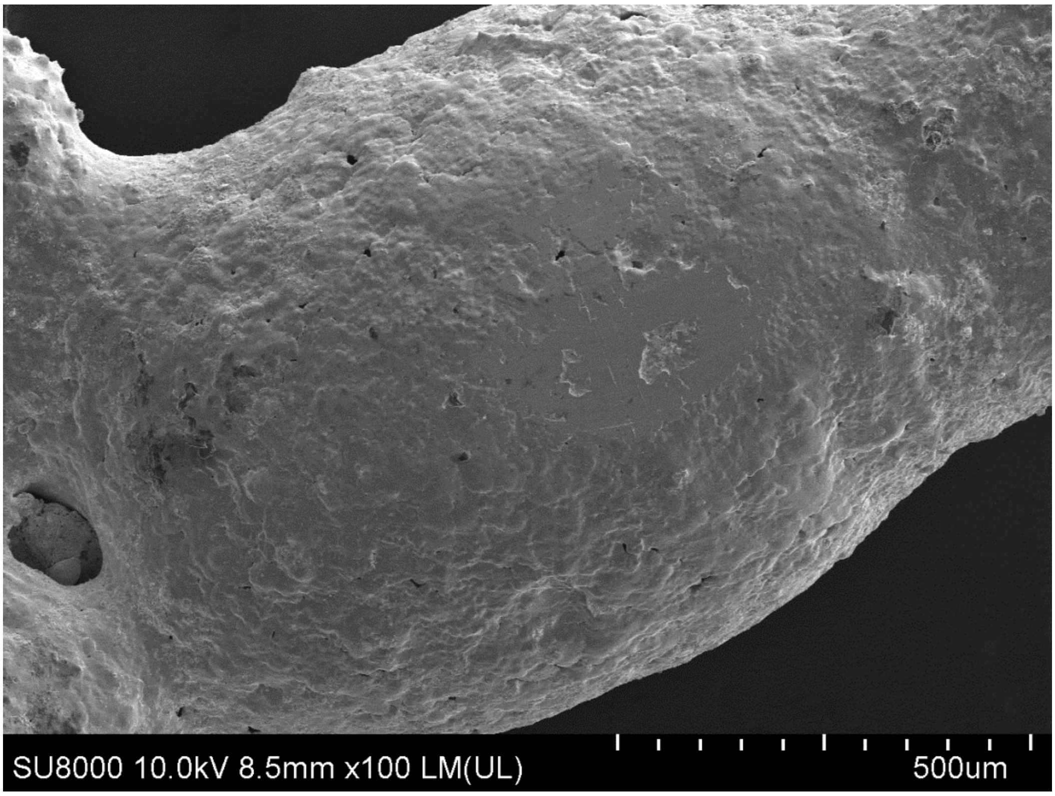

Figure S20. SEM image of Ag powder sample after MW treatment.

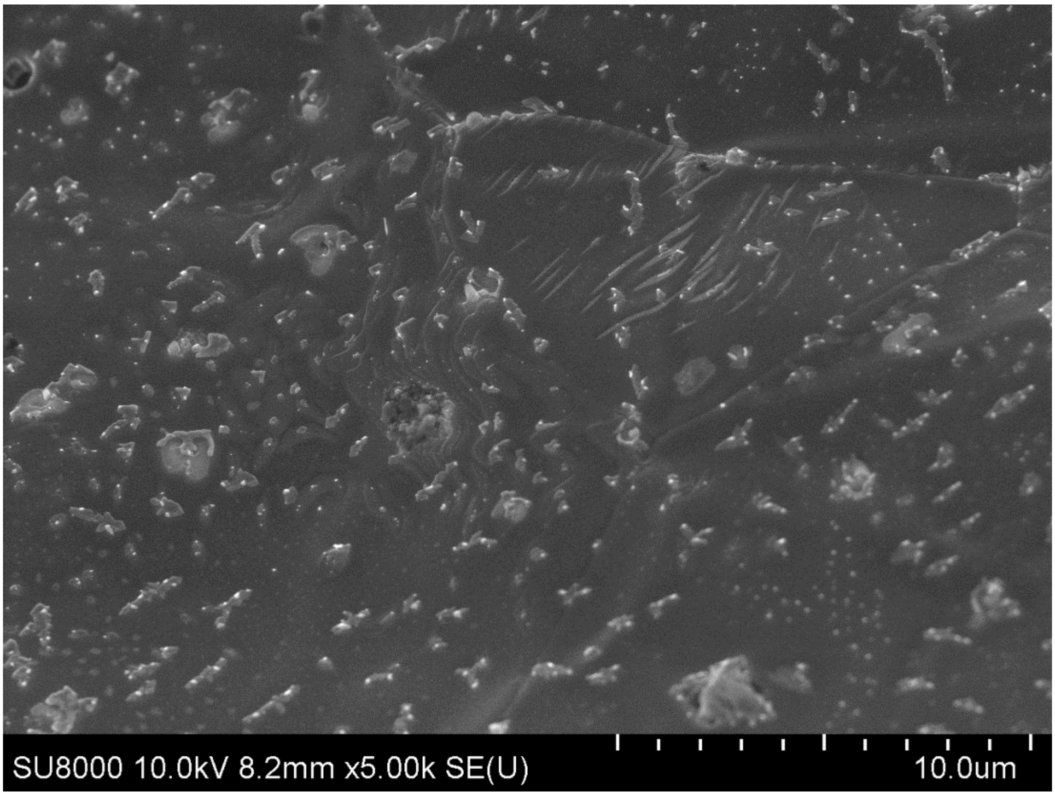

Figure S21. SEM image of Ag powder sample after MW treatment.

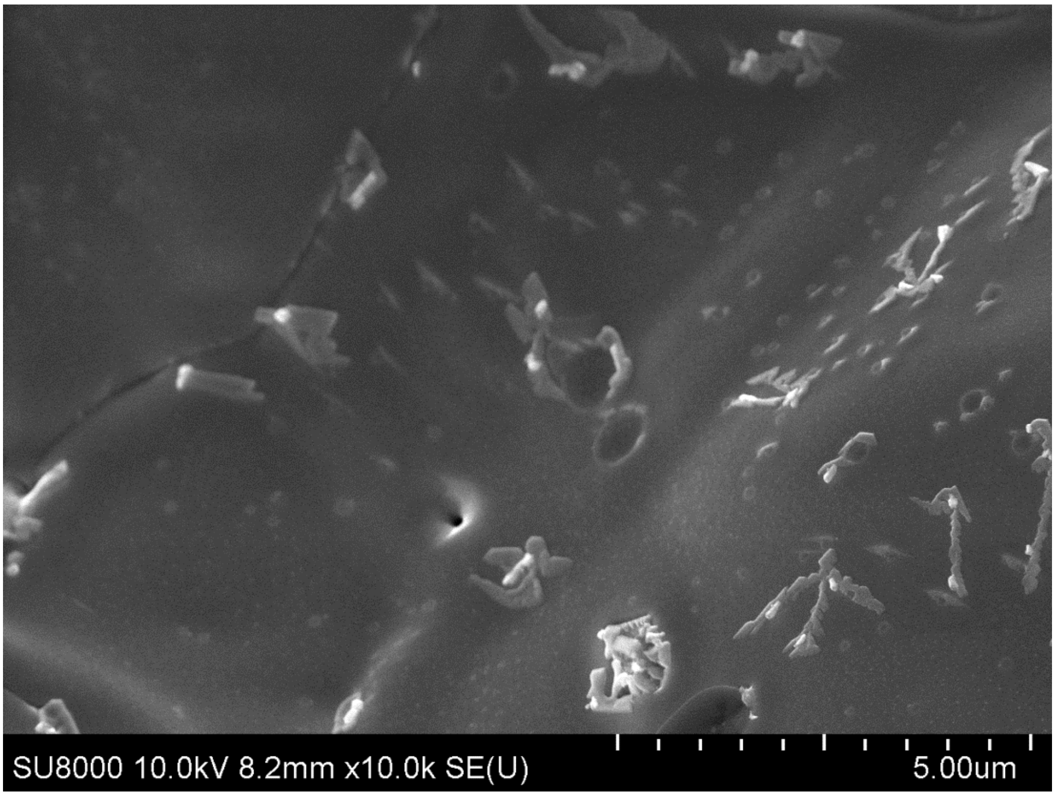

Figure S22. SEM image of Ag powder sample after MW treatment.

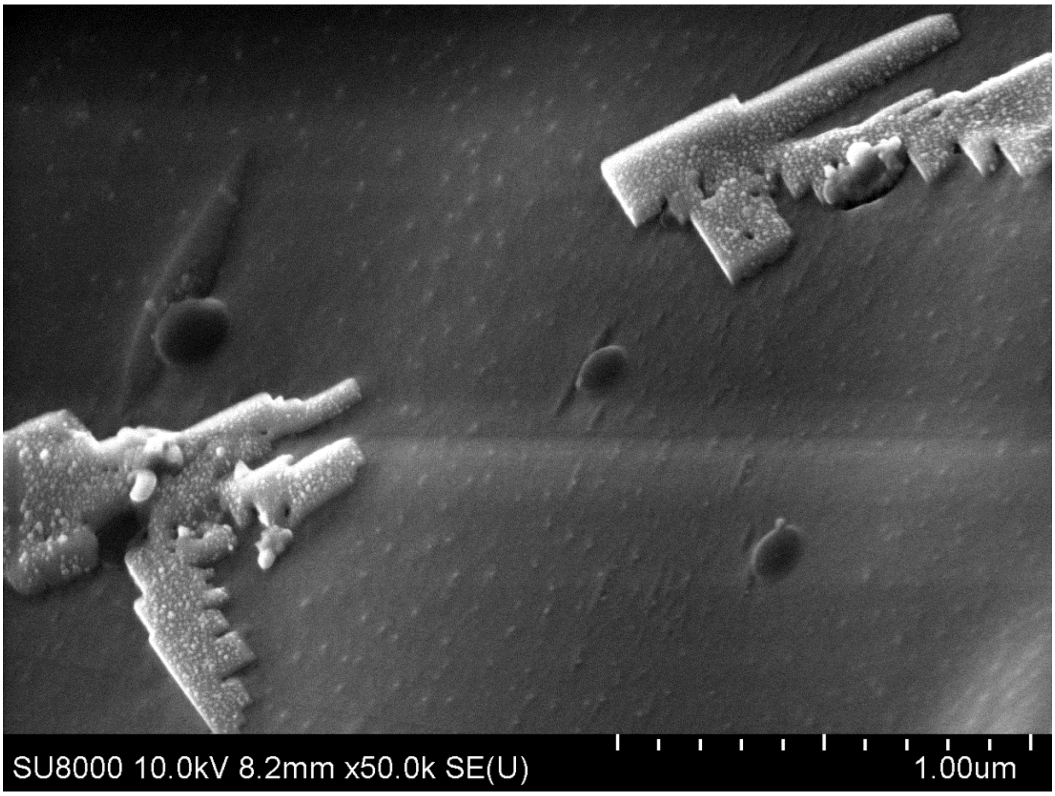

Figure S23. SEM image of Ag powder sample after MW treatment.

EDX data of Ag powder sample after MW treatment

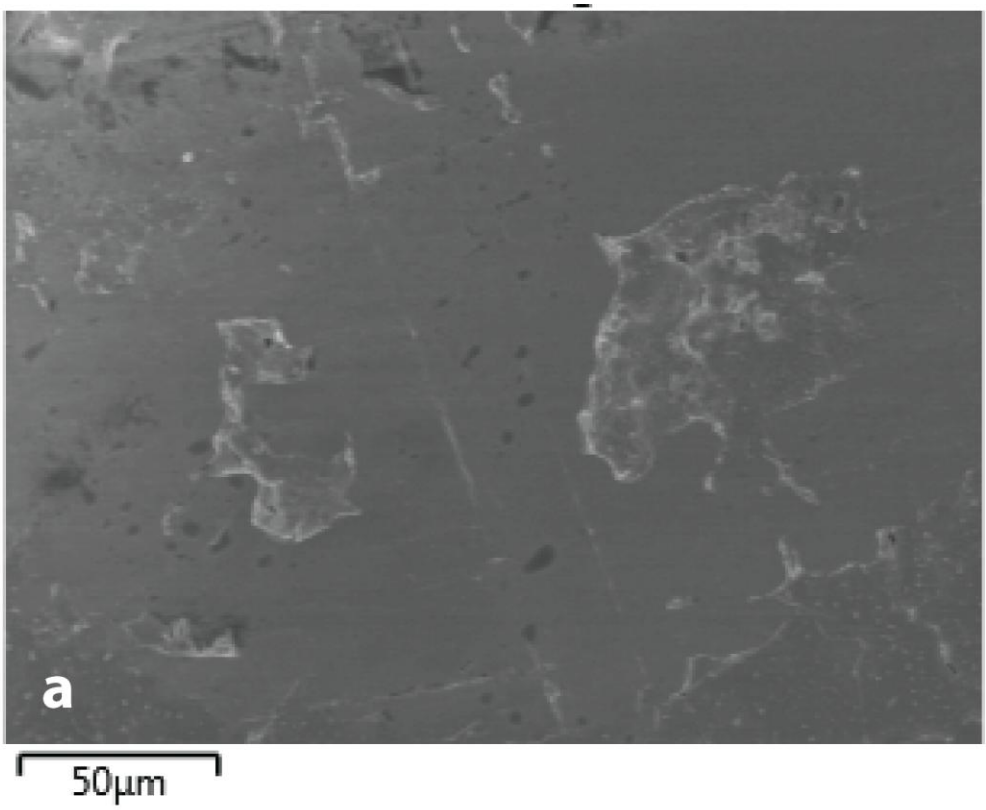

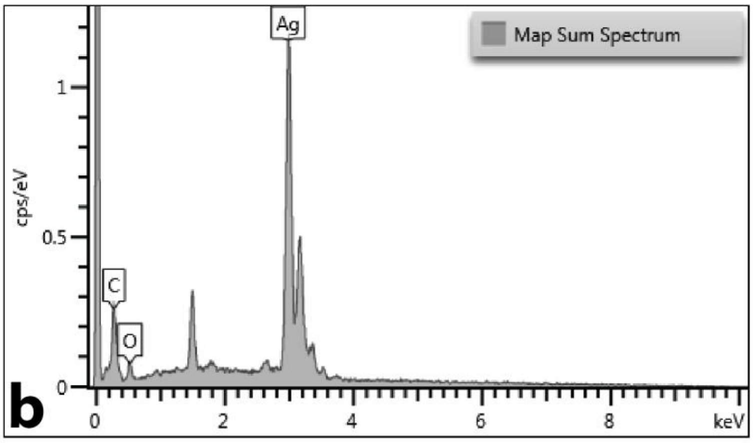

c

Element	Wt%	At%
Ag	94.9	69.6
C	3.0	19.8
O	2.1	10.6
Total:	100	100

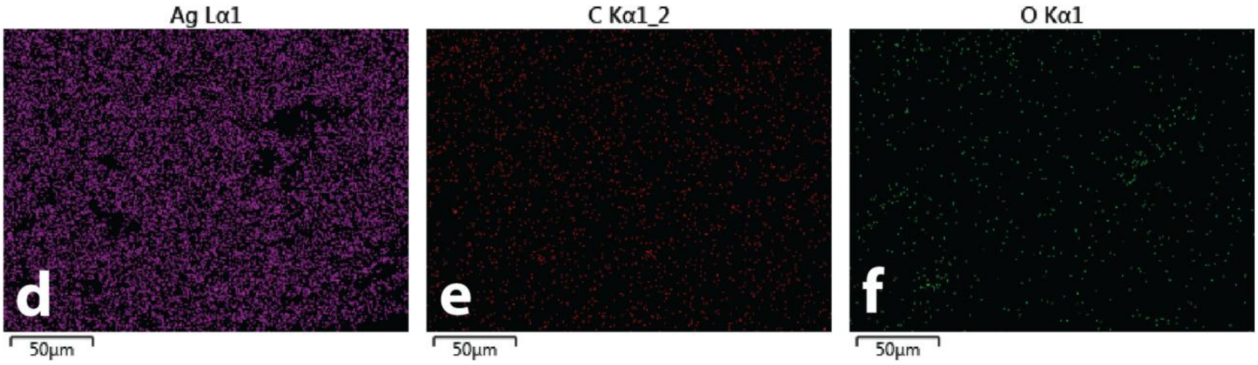

Figure S24. EDX study of Ag powder after MW treatment: SEM image (a); EDX spectrum of this area (b); element composition (c) and maps of silver (d), carbon (e), and oxygen (f) distributions. The presence of carbon and oxygen in the EDX analysis result by their presence in the carbon tape and stub which fixed samples for SEM.

SEM images of initial sample of Re powder

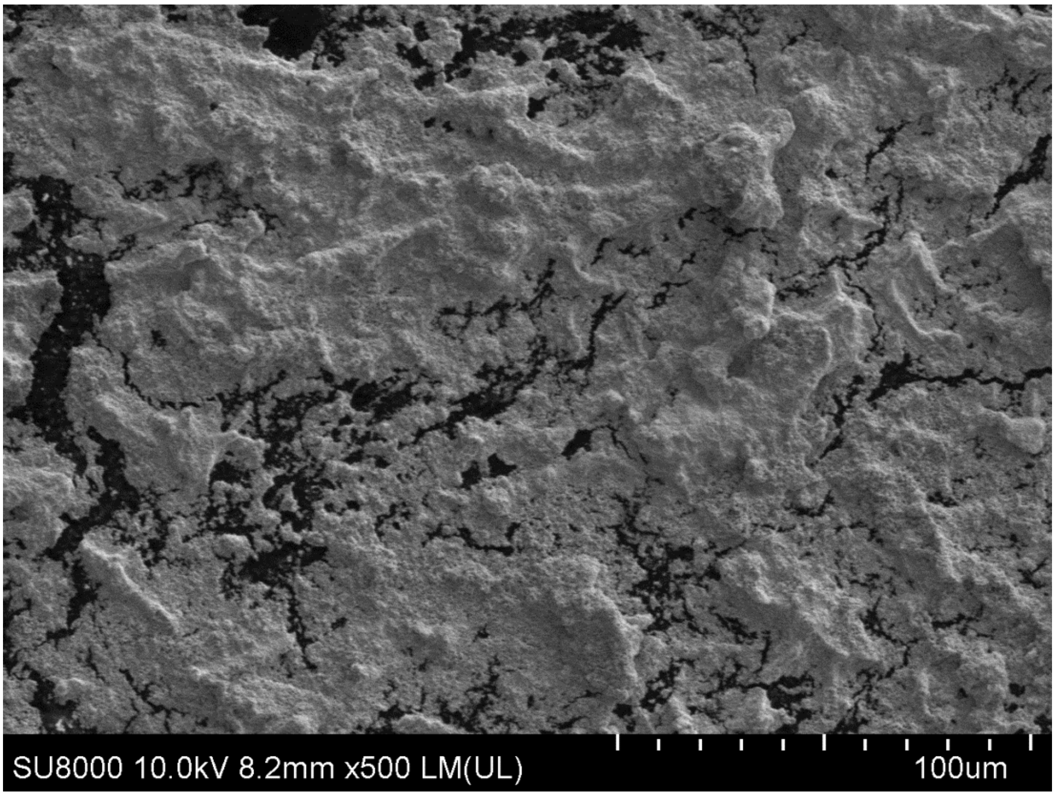

Figure S25. SEM image of initial sample of Re powder.

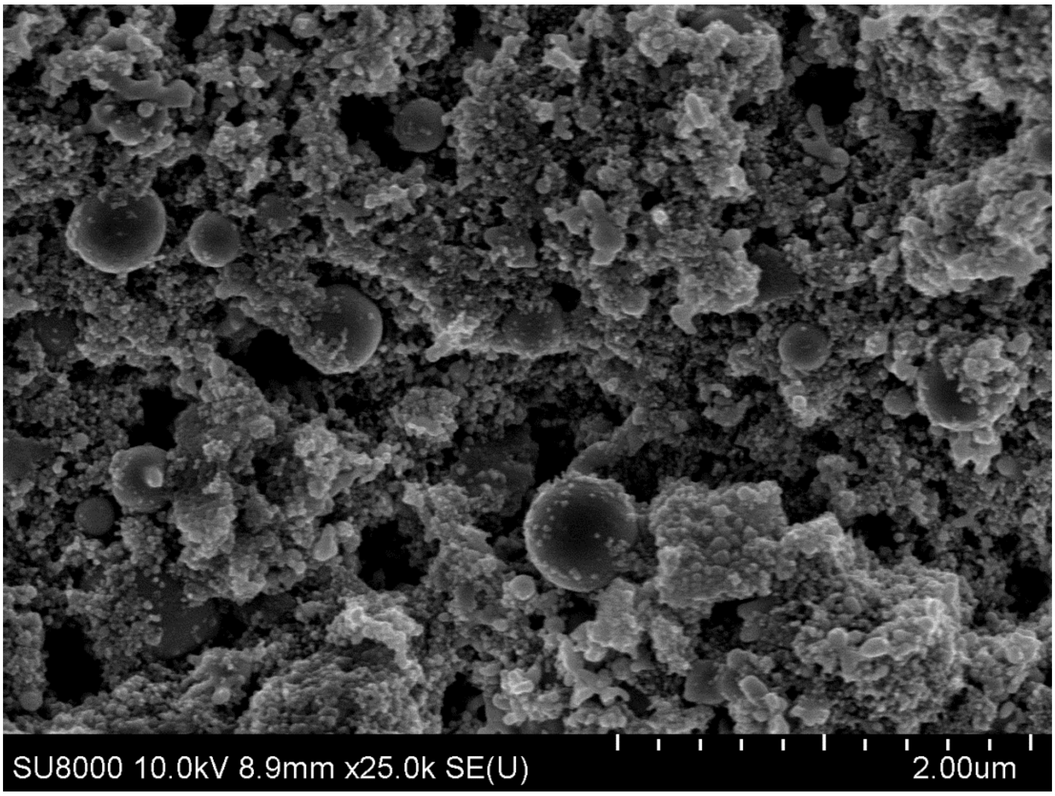

Figure S26. SEM image of initial sample of Re powder.

EDX data of initial sample of Re powder

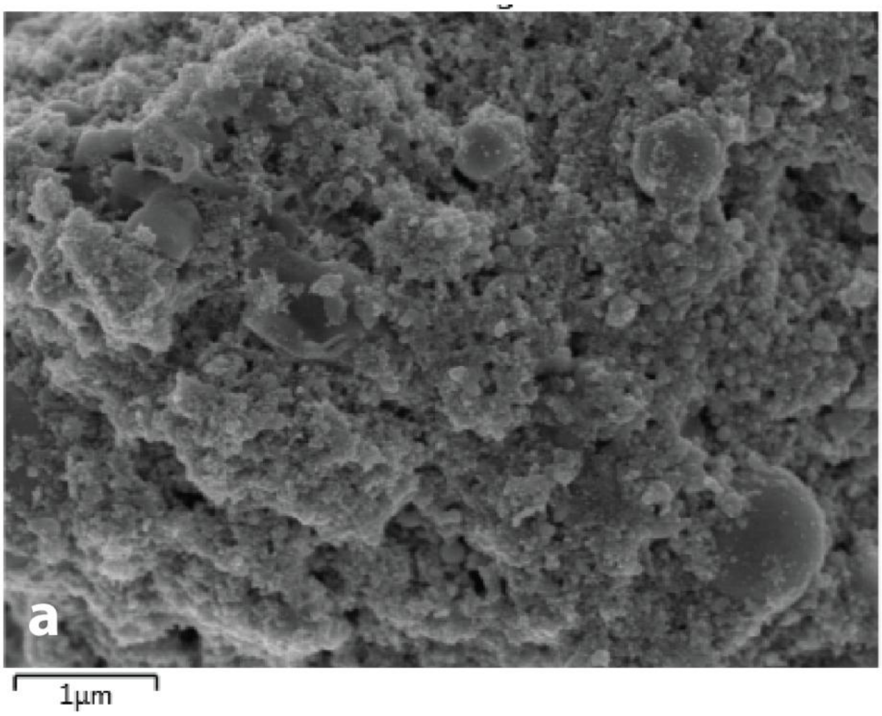

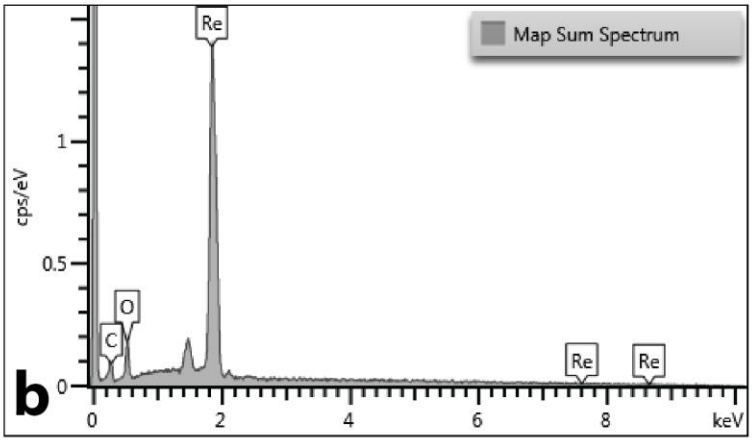

c

Element	Wt%	At%
Re	90.7	42.0
C	4.5	32.3
O	4.8	25.7
Total:	100	100

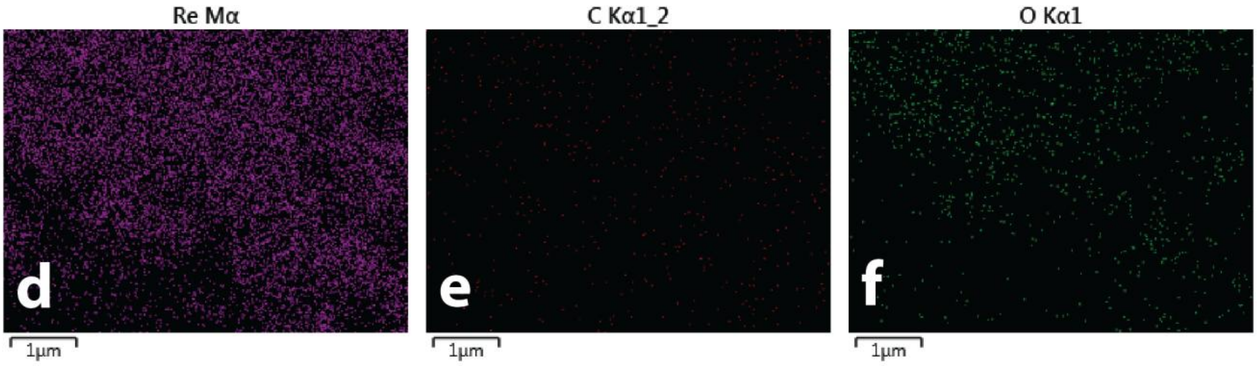

Figure S27. EDX study of initial Re sample: SEM image (a); EDX spectrum of this area (b); element composition (c) and maps of rhenium (d), carbon (e), and oxygen (f) distributions. The original nanopowder does not contain carbon. The presence of carbon and the increased oxygen content in the EDX analysis result by their presence in the carbon tape and stub which fixed samples for SEM. In addition, an oxide film on the surface of the sample can be a source of the oxygen signal.

SEM images of Re powder sample after MW treatment

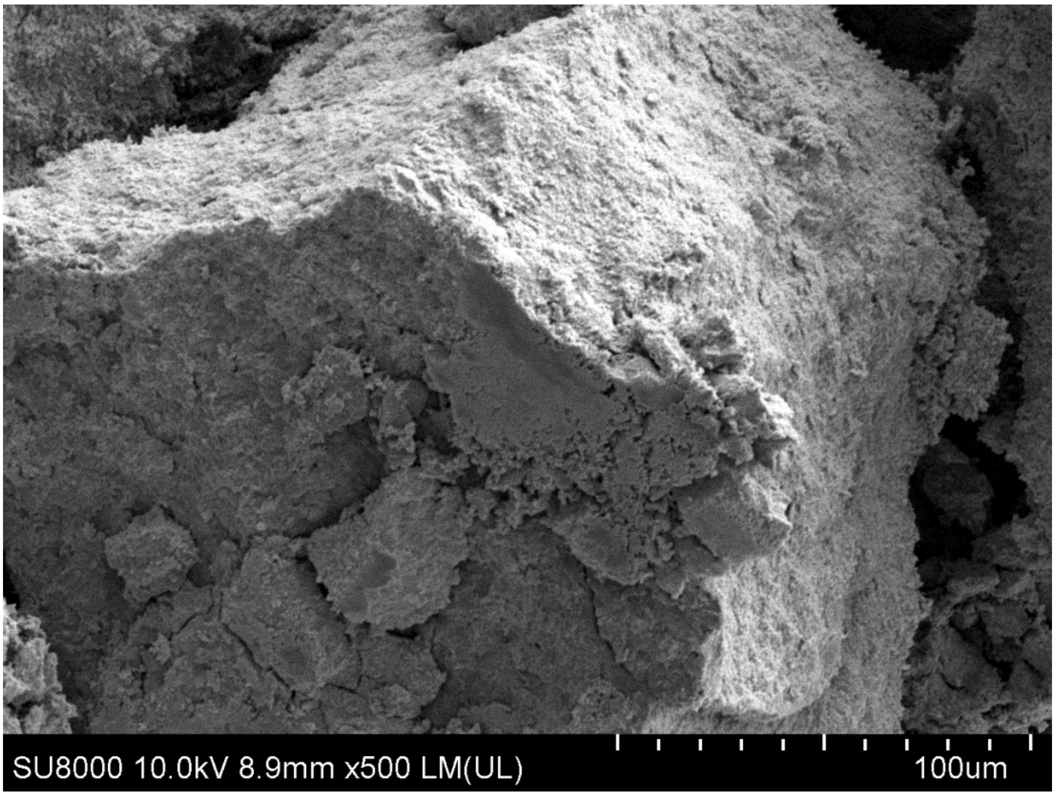

Figure S28. SEM image of Re powder sample after MW treatment.

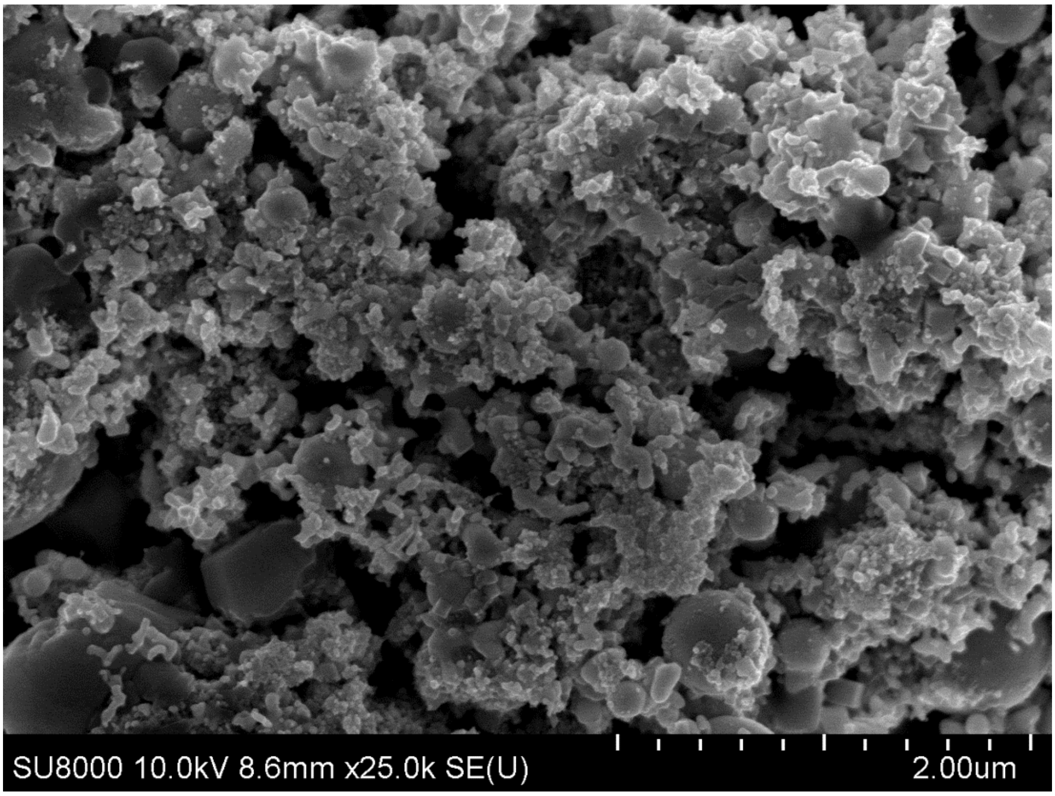

Figure S29. SEM image of Re powder sample after MW treatment.

EDX data of Re powder sample after MW treatment

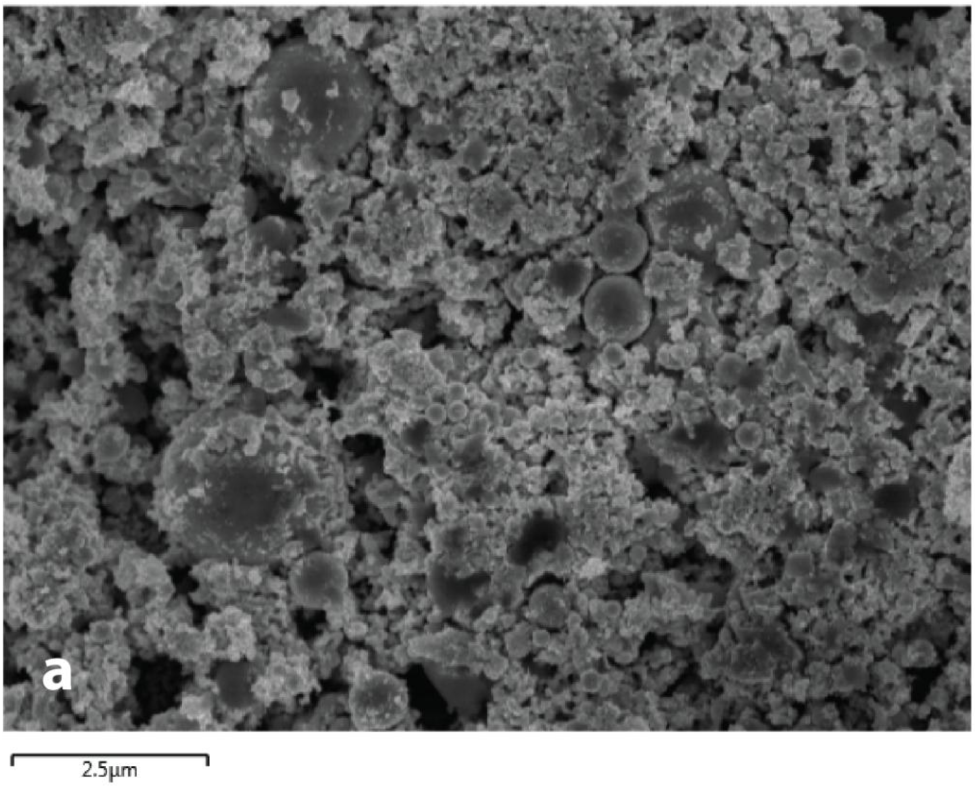

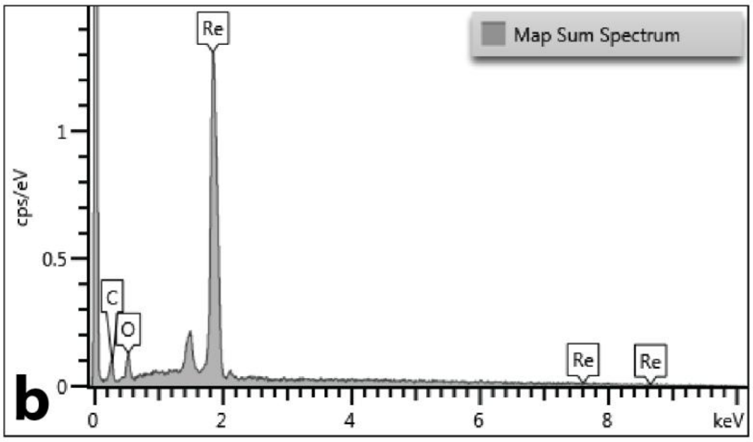

c

Element	Wt%	At%
Re	87.0	33.7
C	4.9	29.7
O	8.1	36.6
Total:	100	100

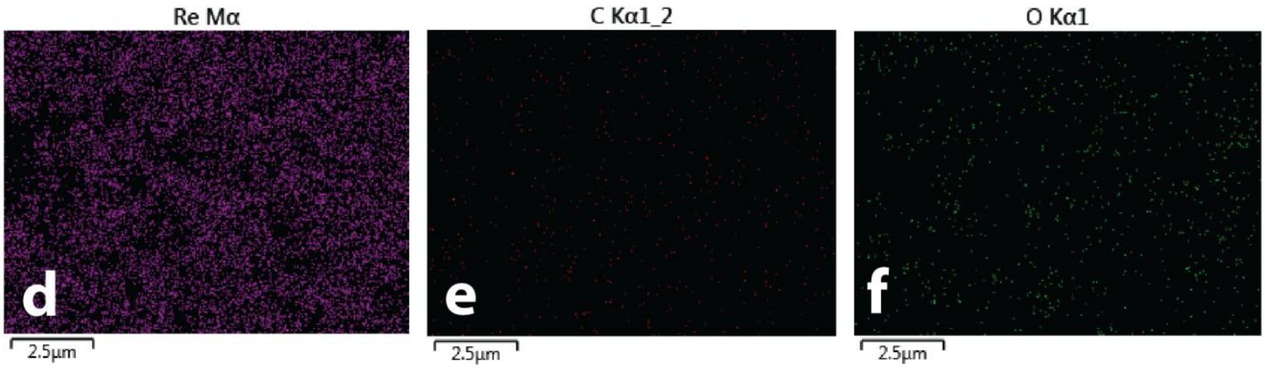

Figure S30. EDX study of Re powder sample after MW treatment: SEM image (a); EDX spectrum of this area (b); element composition (c) and maps of rhenium (d), carbon (e), and oxygen (f) distributions.

SEM images of initial sample of Fe/C powder

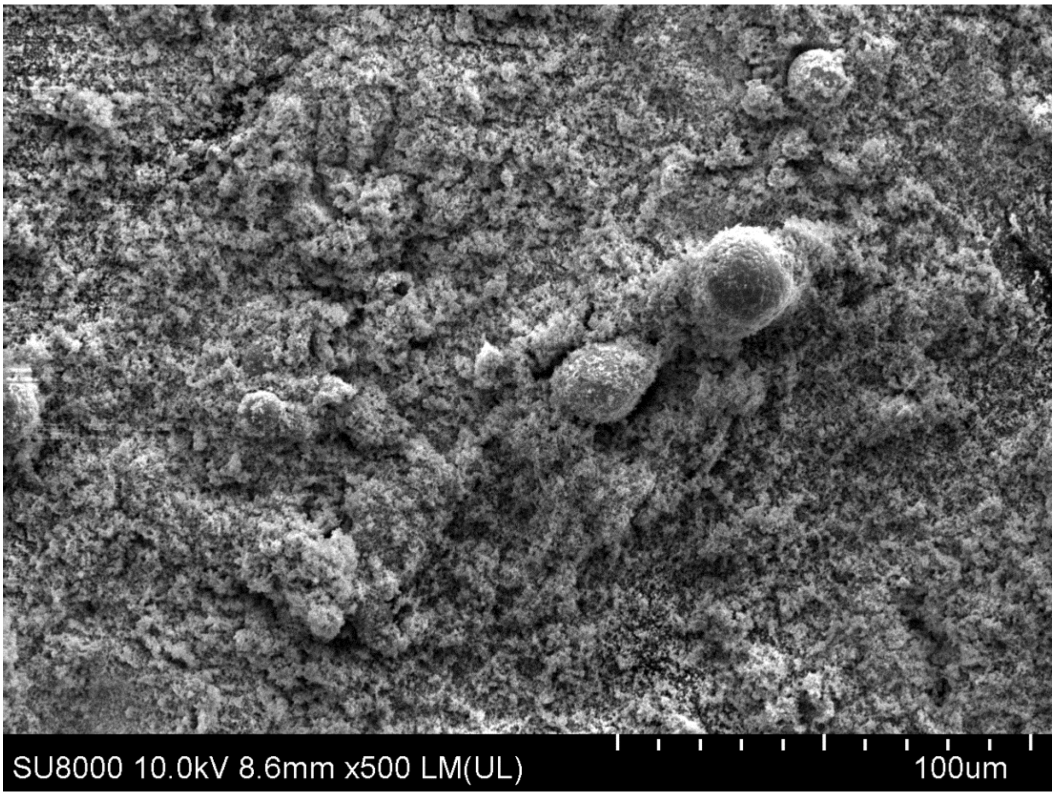

Figure S31. SEM image of initial sample of Fe/C powder.

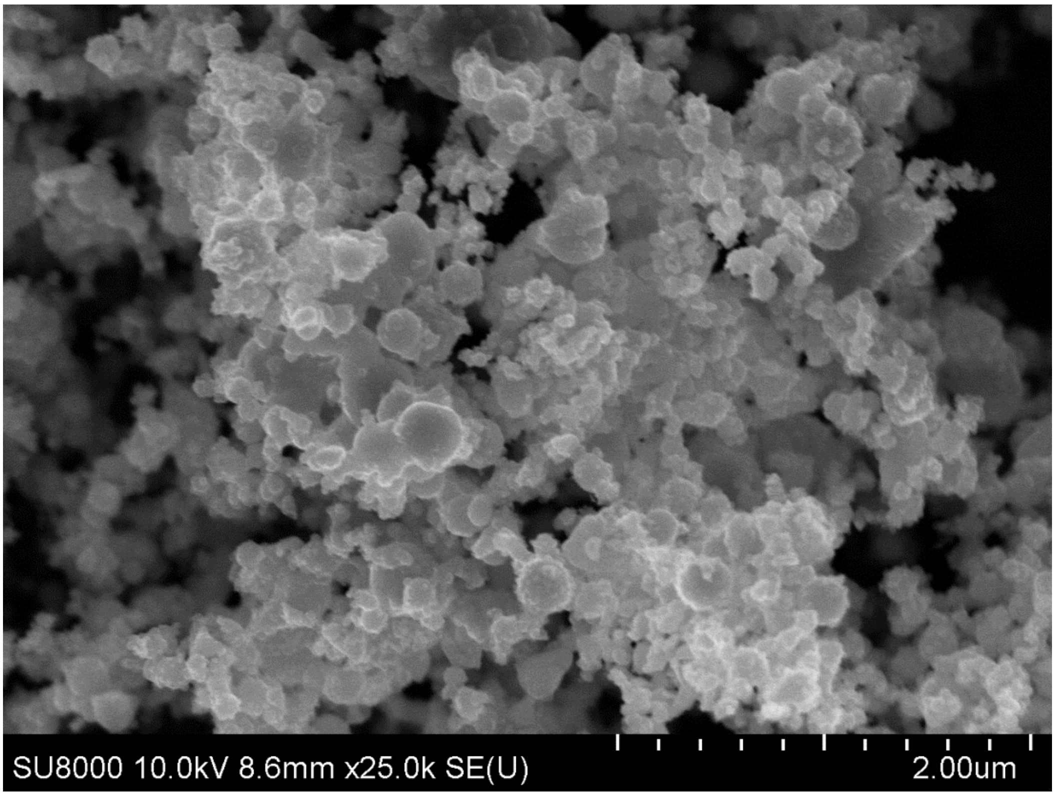

Figure S32. SEM image of initial sample of Fe/C powder.

EDX data of initial sample of Fe/C powder

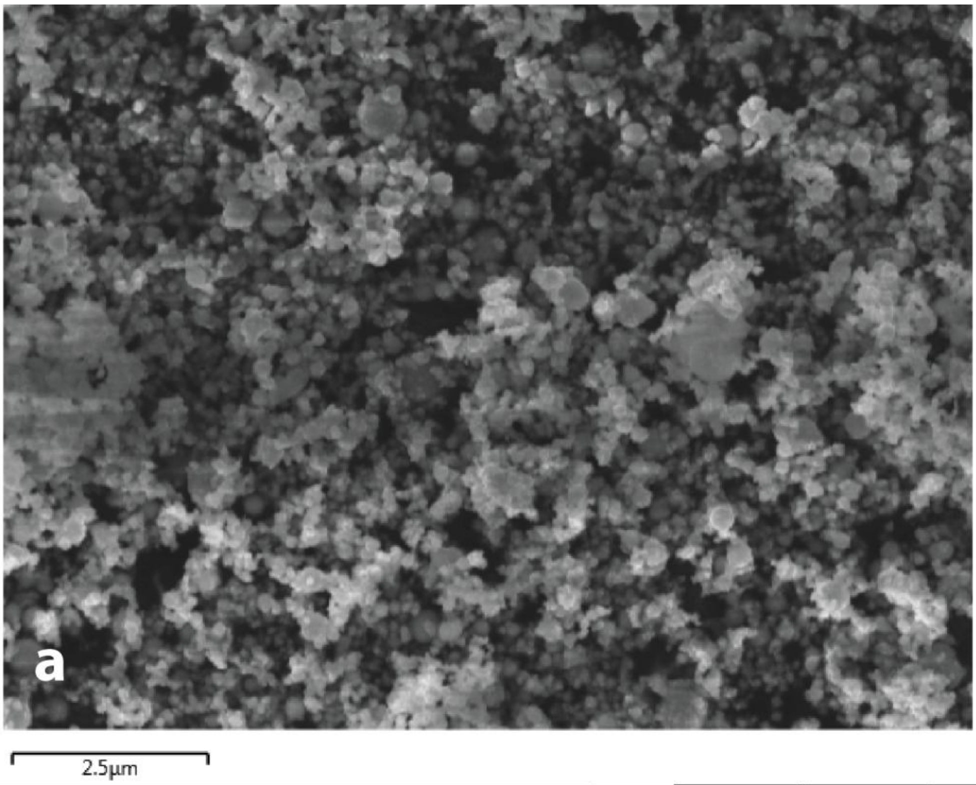

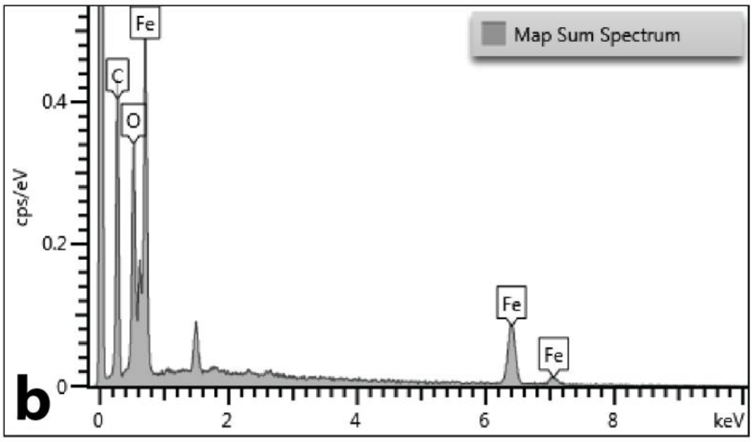

c

Element	Wt%	At%
Fe	68.1	33.0
C	23.1	52.0
O	8.8	15.0
Total:	100	100

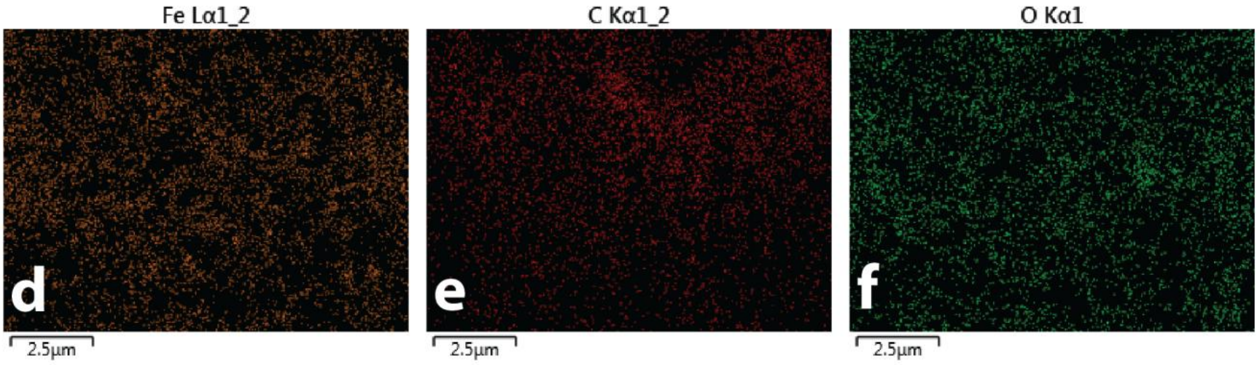

Figure S33. EDX study of initial Fe/C sample: SEM image (a); EDX spectrum of this area (b); element composition (c) and maps of iron (d), carbon (e), and oxygen (f) distributions. The increased oxygen content in the EDX analysis result by its presence in aluminum stub which fixed samples for SEM. In addition, an oxide film on the surface of the sample can be a source of the oxygen signal.

SEM images of Fe/C powder sample after MW treatment

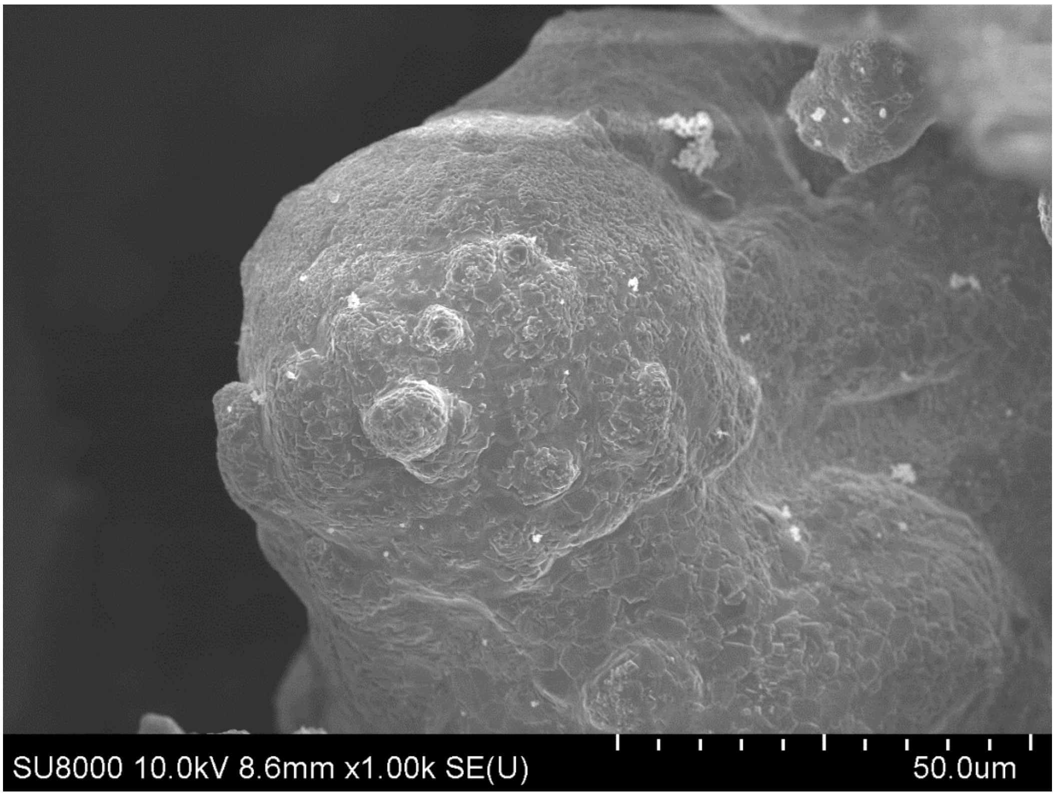

Figure S34. SEM image of Fe/C powder sample after MW treatment (fusion area).

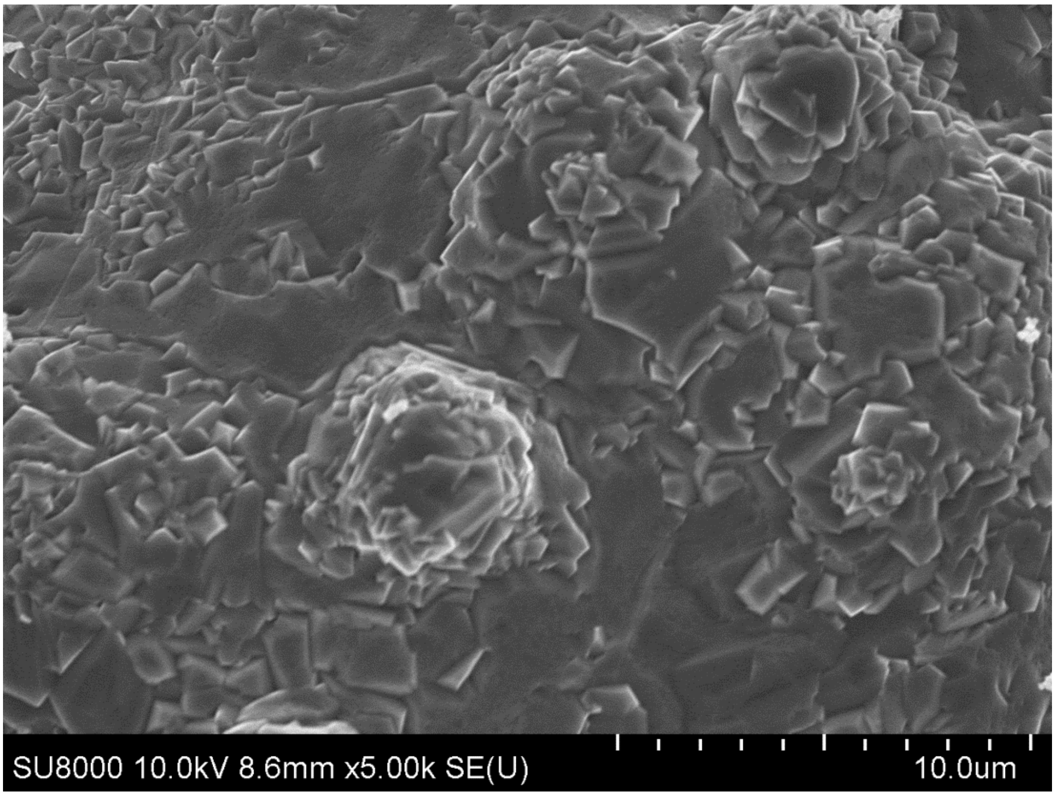

Figure S35. SEM image of Fe/C powder sample after MW treatment (fusion area).

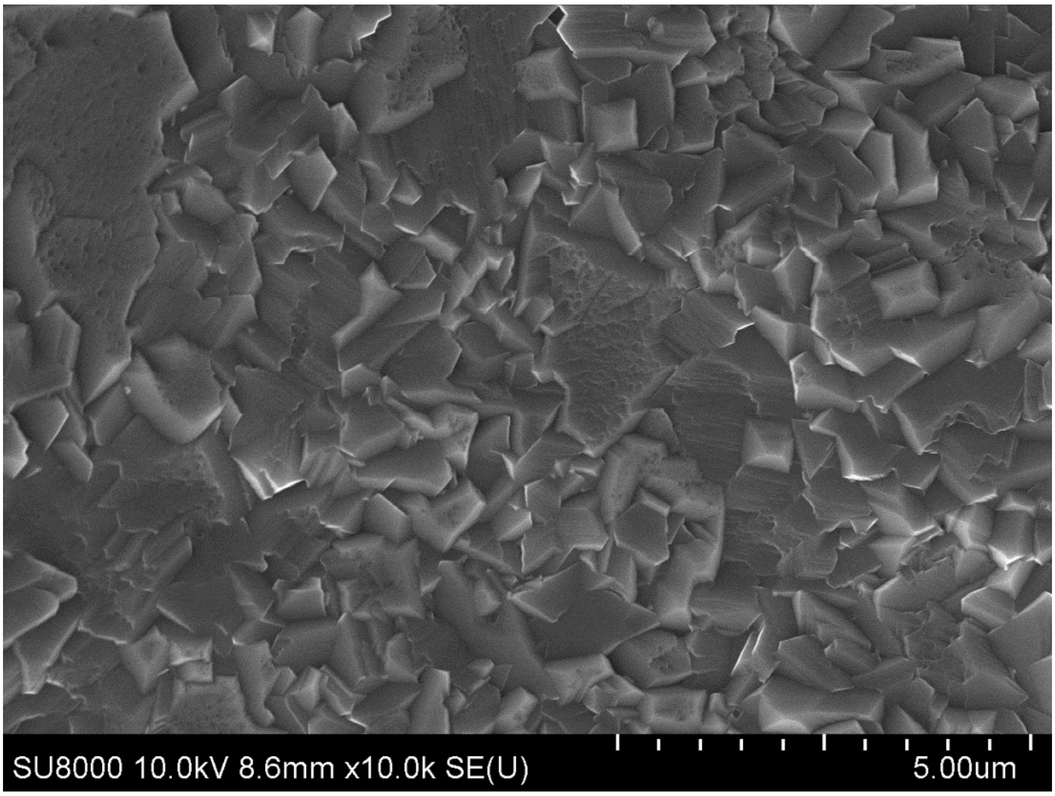

Figure S36. SEM image of Fe/C powder sample after MW treatment (fusion area).

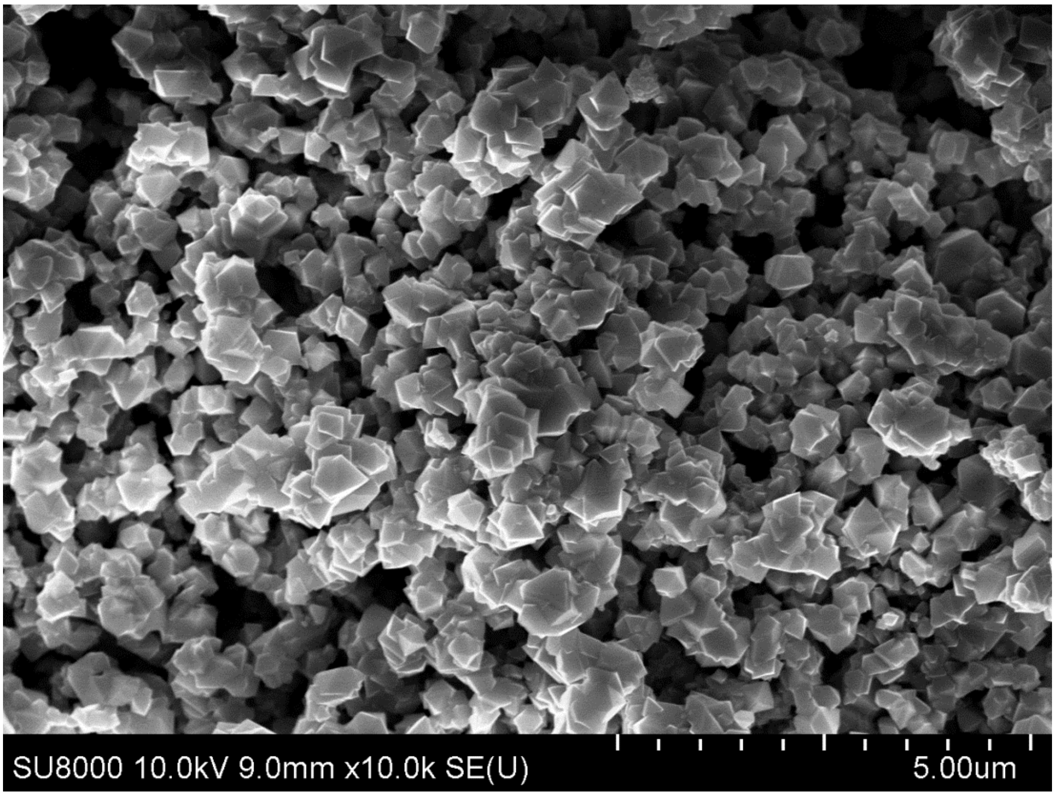

Figure S37. SEM image of Fe/C powder sample after MW treatment (powdered area).

EDX data of Fe/C powder sample after MW treatment

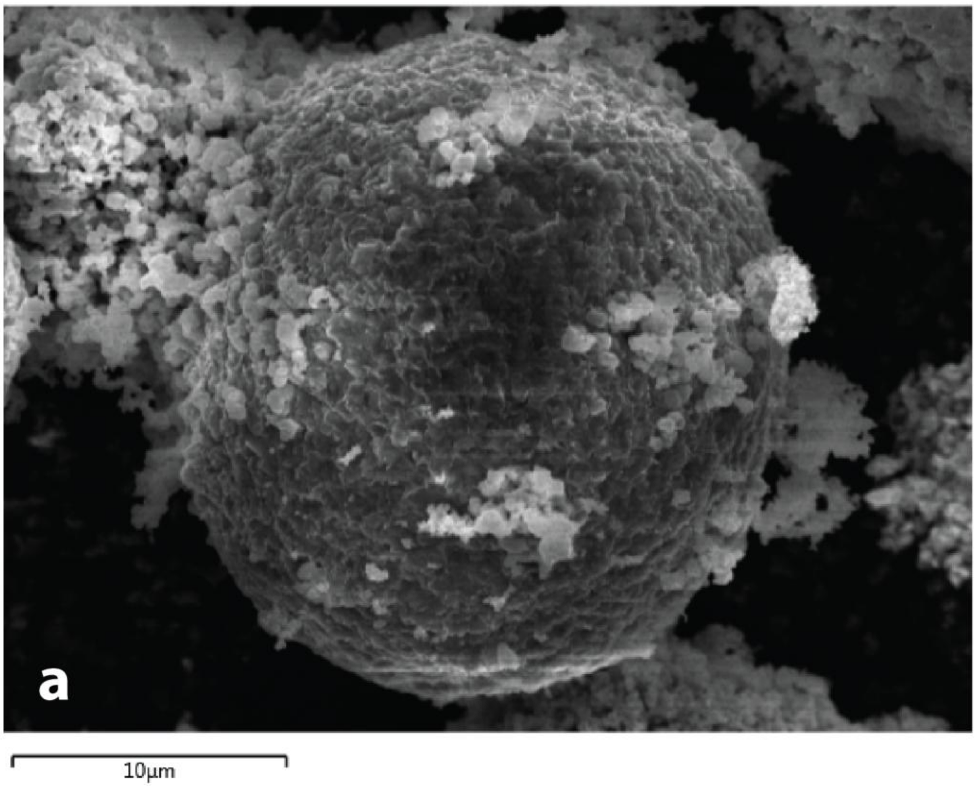

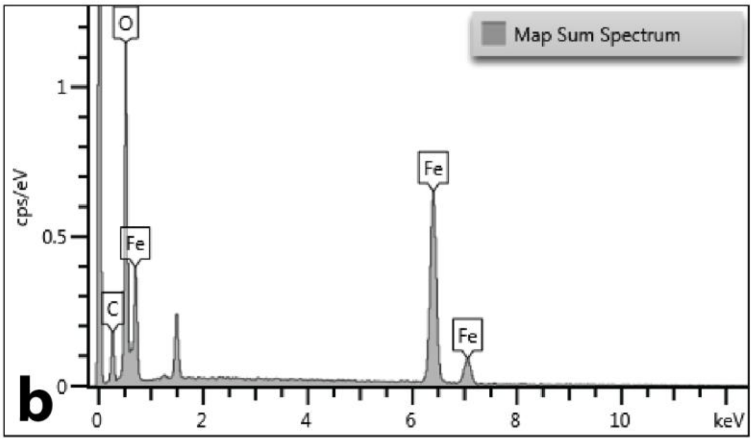

c

Element	Wt%	At%
Fe	67.9	35.2
C	11.1	26.8
O	21.0	38.0
Total:	100	100

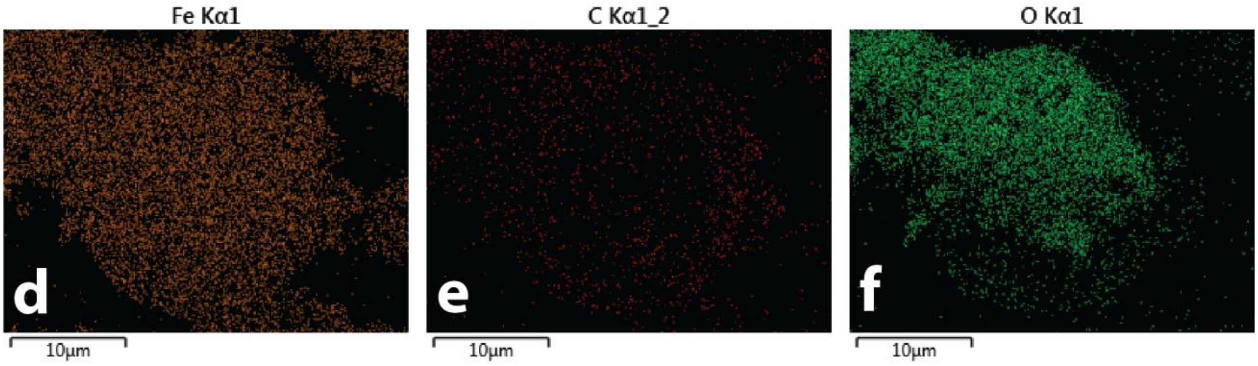

Figure S38. EDX study of Fe/C powder after MW treatment: SEM image (a); EDX spectrum of this area (b); element composition (c) and maps of iron (d), carbon (e), and oxygen (f) distributions.

SEM images of initial sample of Mo-Fe-C powder

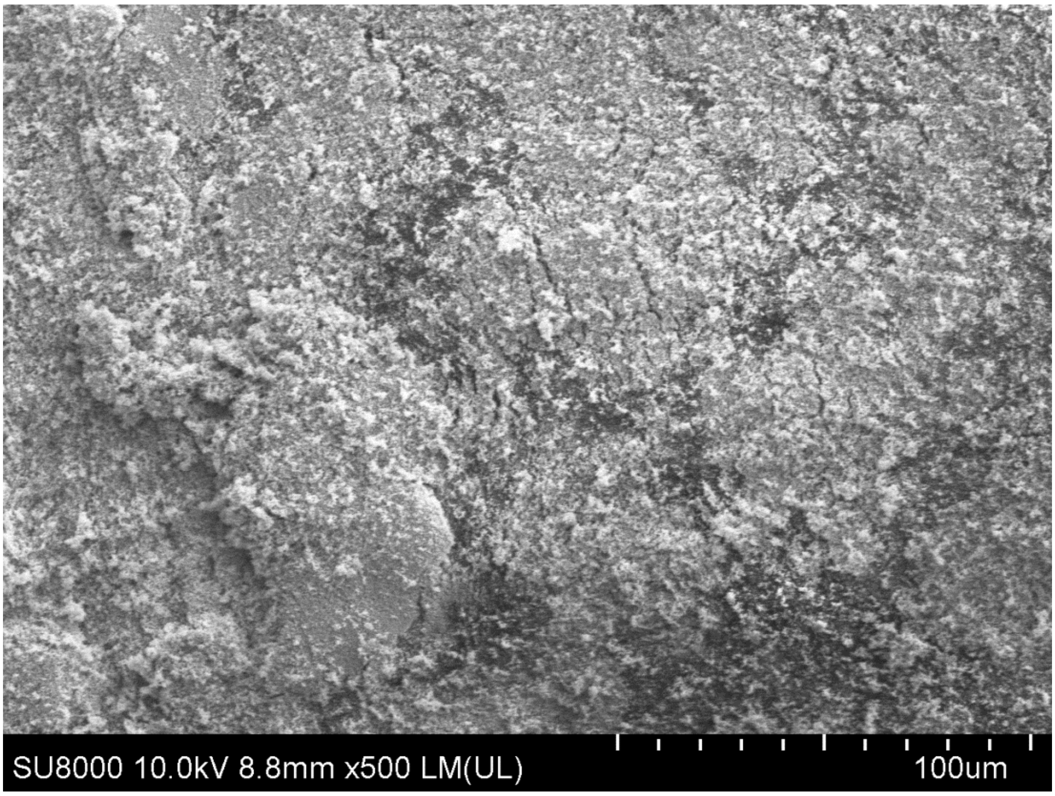

Figure S39. SEM image of initial sample of Mo-Fe-C powder.

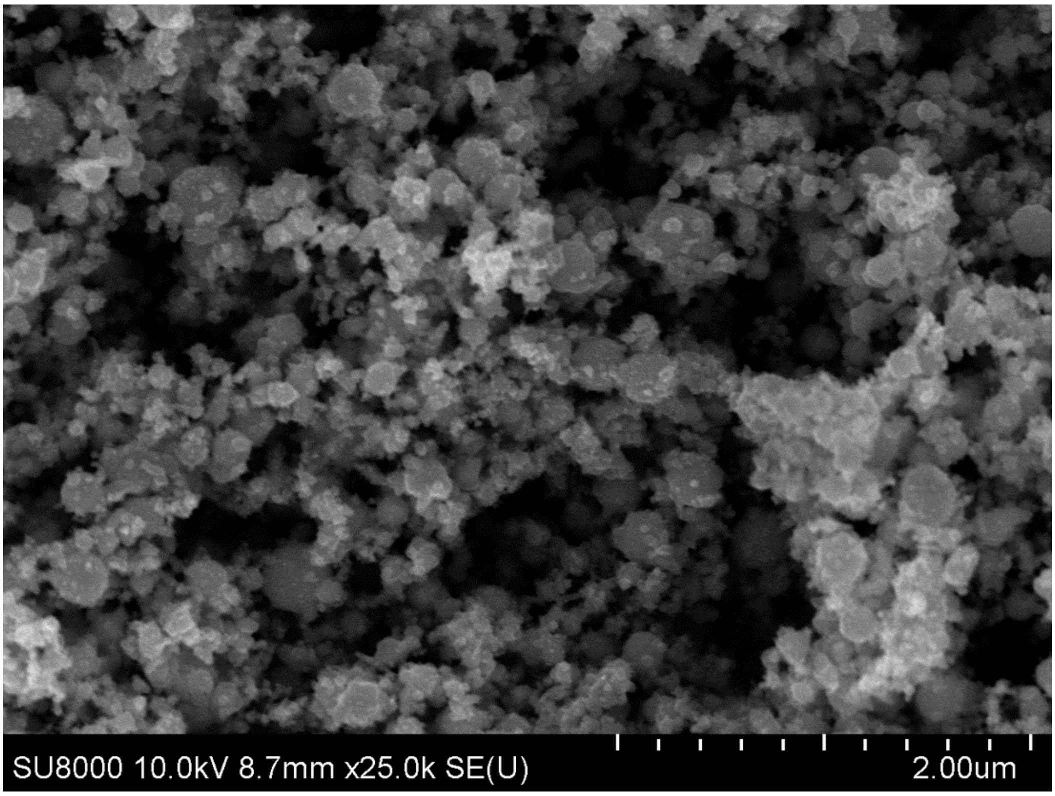

Figure S40. SEM image of initial sample of Mo-Fe-C powder.

EDX data of initial sample of Mo-Fe-C powder

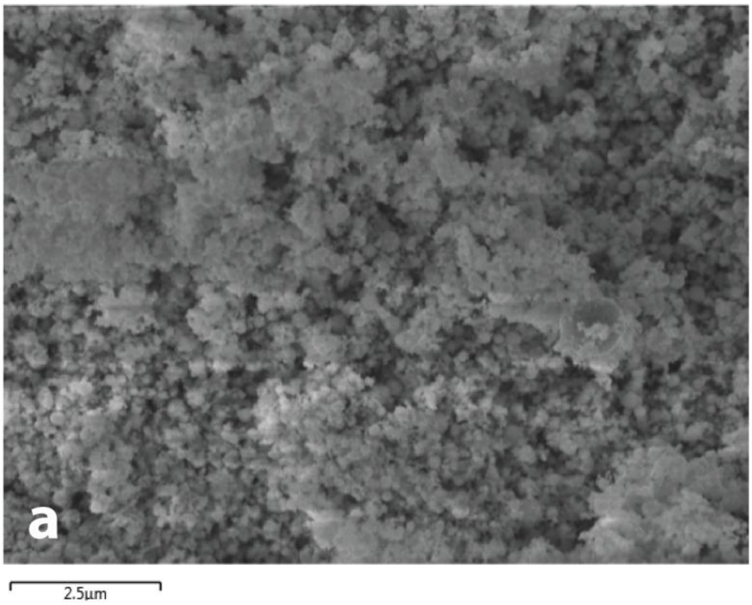

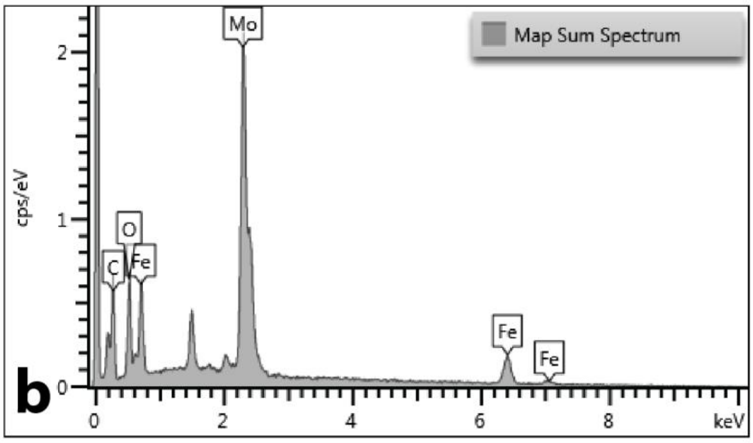

c

Element	Wt%	At%
Mo	47.9	18.7
Fe	31.0	20.8
C	13.8	43.2
O	7.3	17.2
Total:	100	100

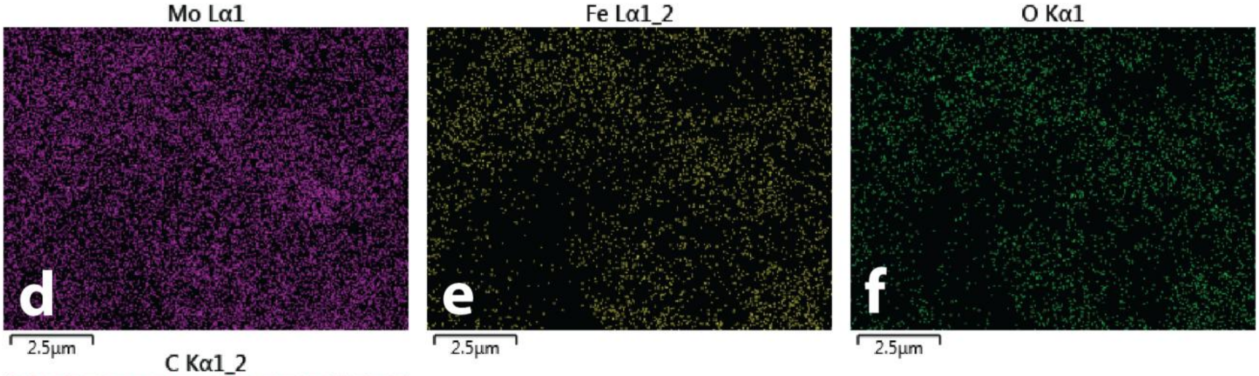

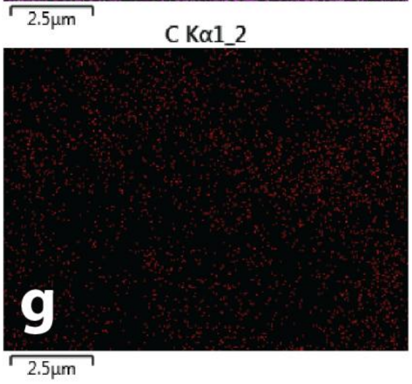

Figure S41. EDX study of initial Mo-Fe-C sample: SEM image (a); EDX spectrum of this area (b); element composition (c) and maps of molybdenum (d), iron (e), oxygen (f), and carbon (g) distributions. The increased oxygen content in the EDX analysis result by its presence in aluminum stub which fixed samples for SEM. In addition, an oxide film on the surface of the sample can be a source of the oxygen signal.

SEM images of Mo-Fe-C powder sample after MW treatment

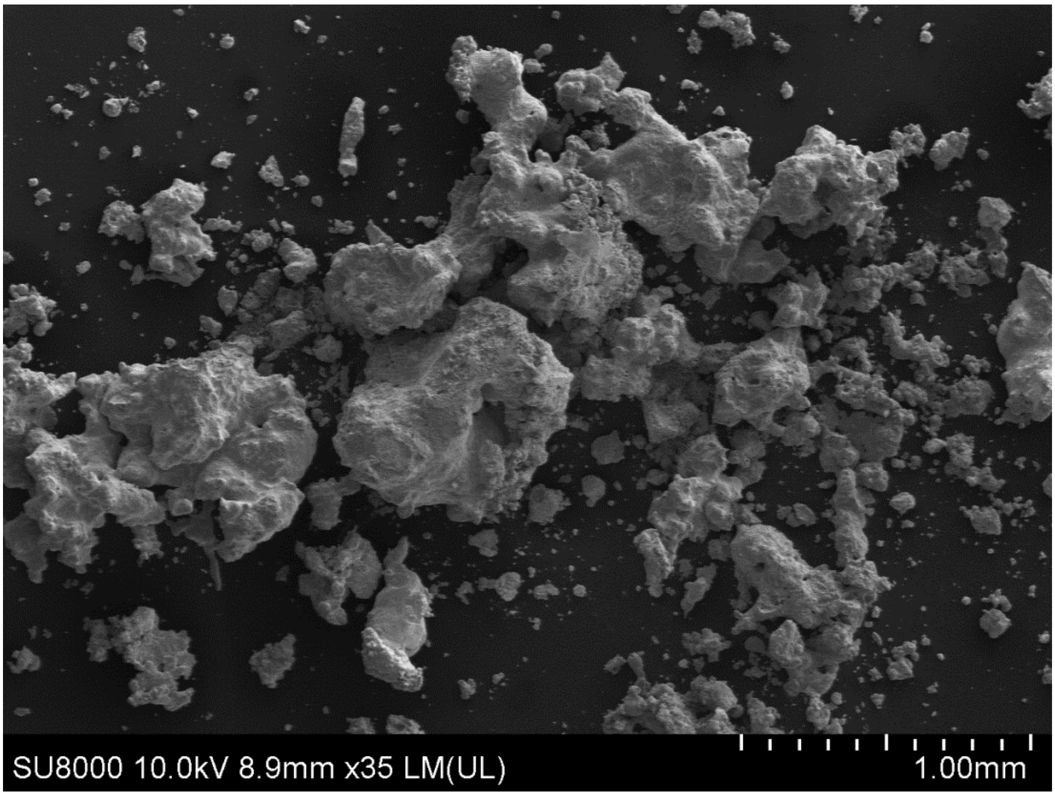

Figure S42. SEM image of Mo-Fe-C powder sample after MW treatment (fusion area).

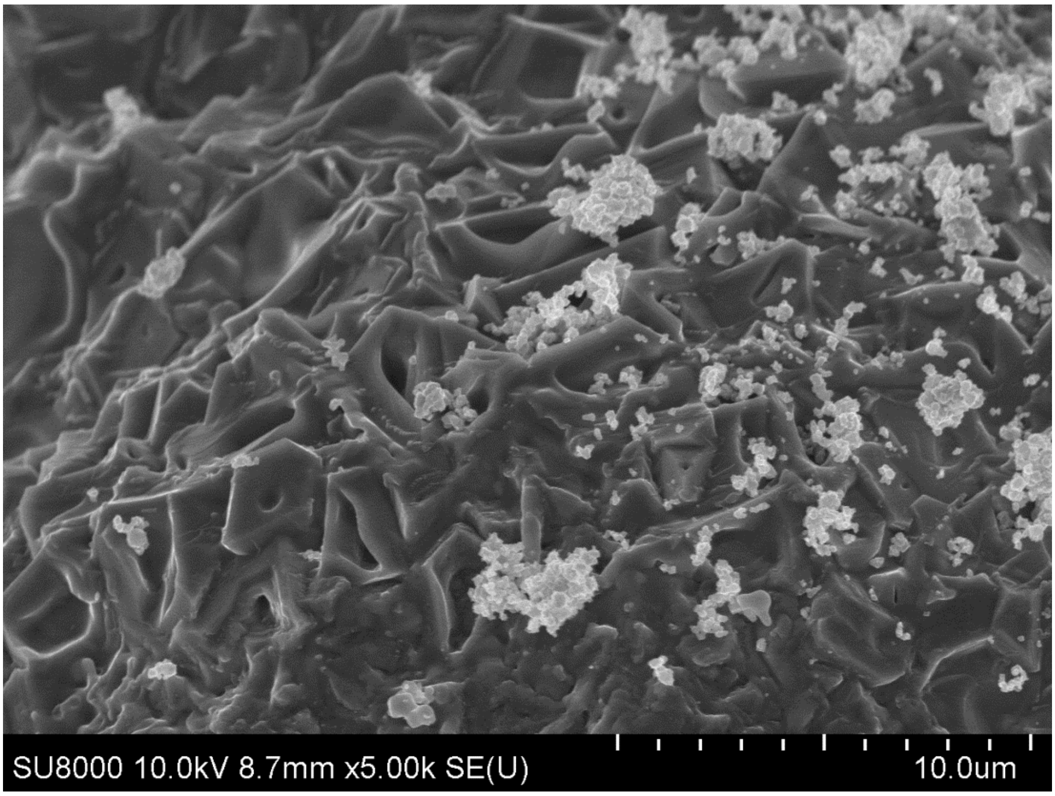

Figure S43. SEM image of Mo-Fe-C powder sample after MW treatment (fusion area).

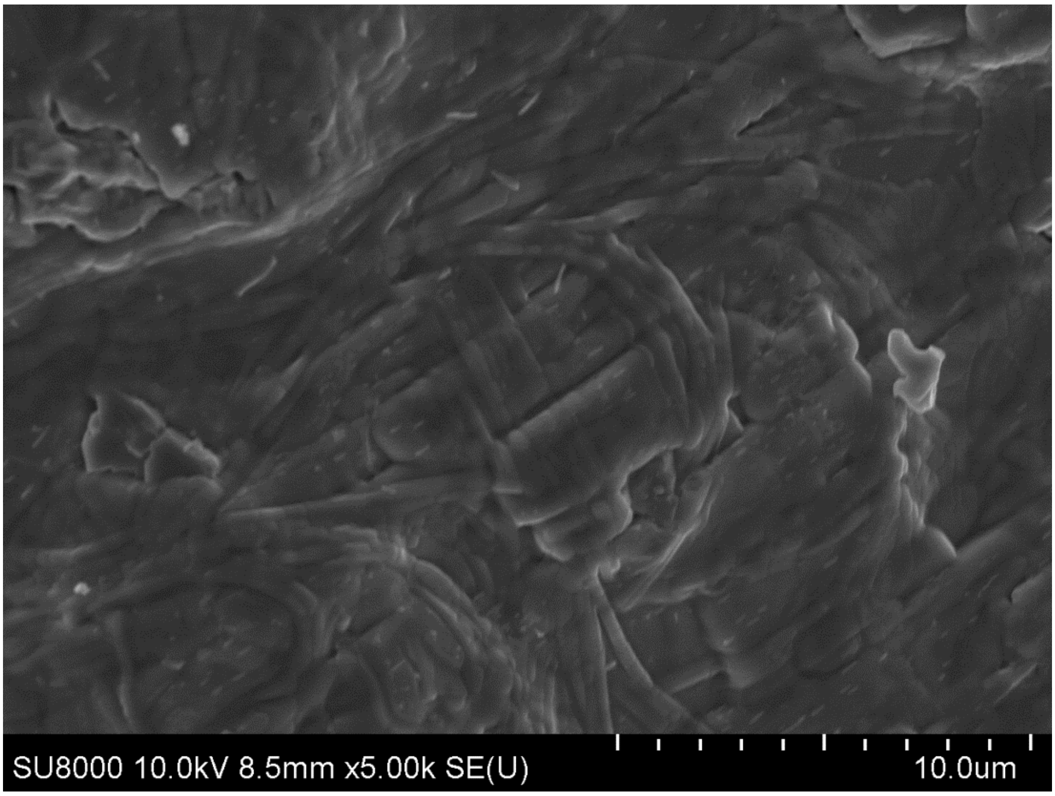

Figure S44. SEM image of Mo-Fe-C powder sample after MW treatment (fusion area).

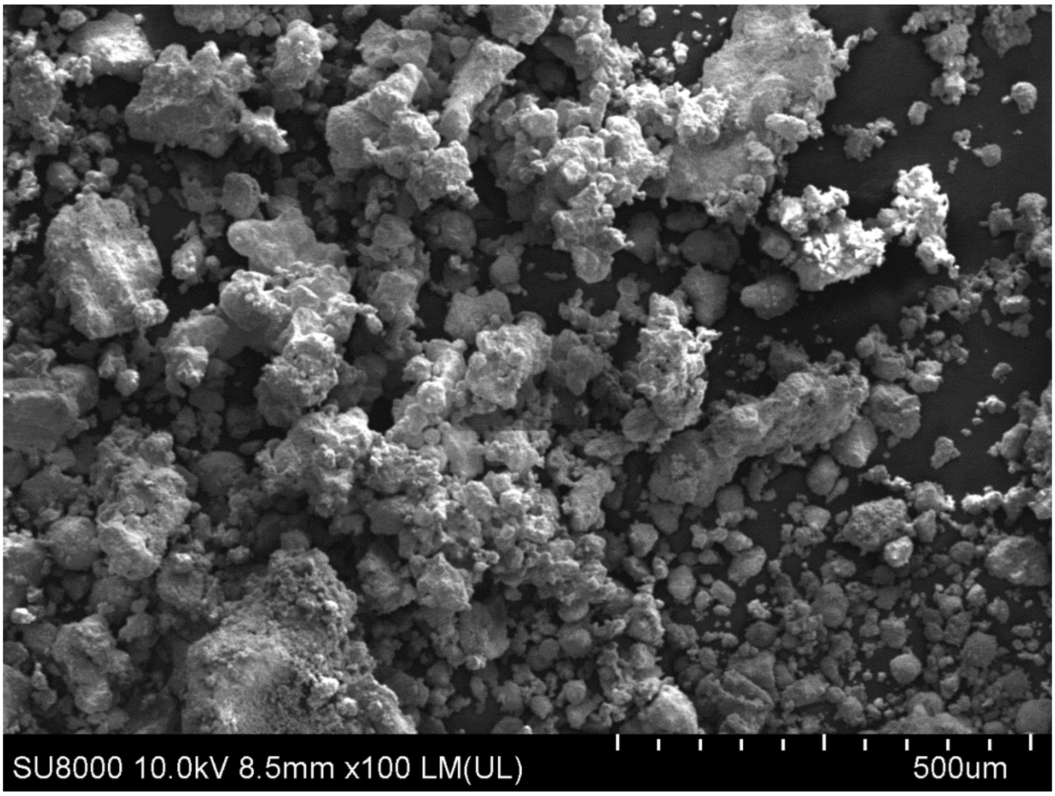

Figure S45. SEM image of Mo-Fe-C powder sample after MW treatment (powdered area).

EDX data of Mo-Fe-C powder sample after MW treatment

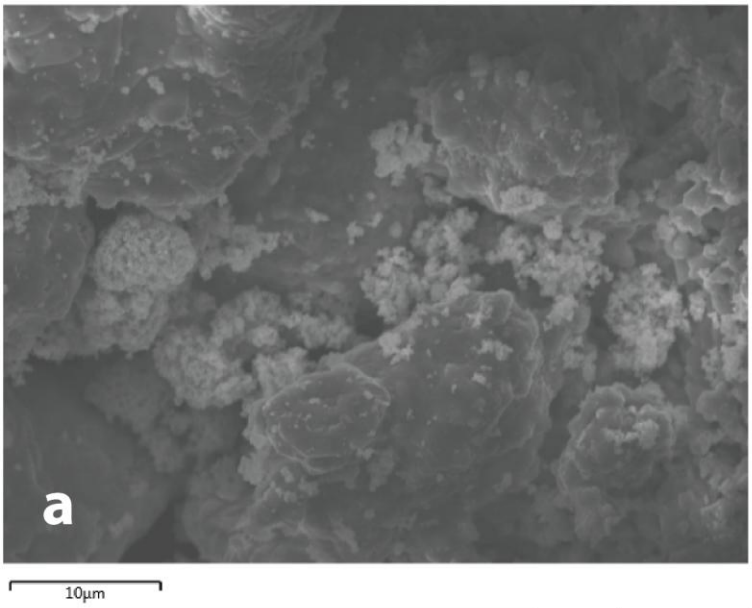

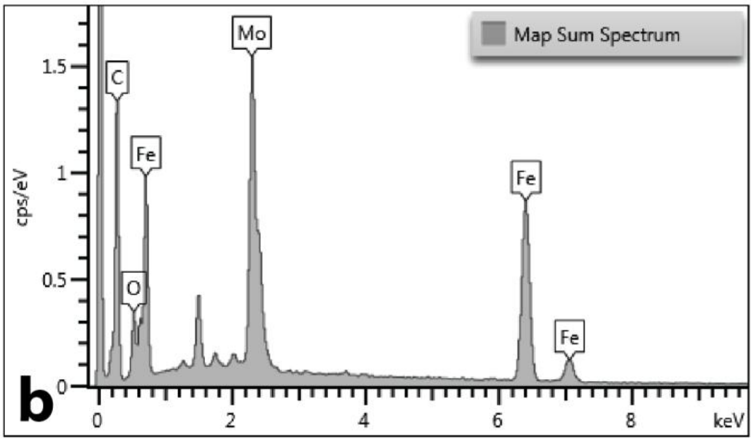

c

Element	Wt%	At%
Mo	33.3	9.8
Fe	28.7	14.6
C	14.1	33.4
O	23.8	42.2
Total:	100	100

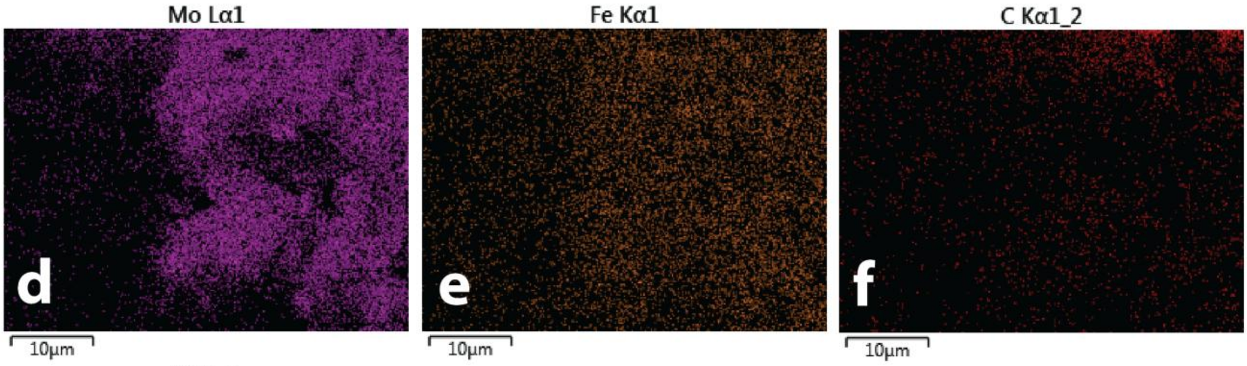

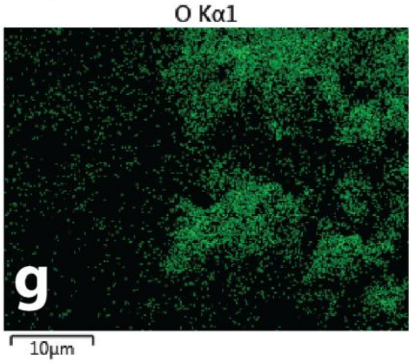

Figure S46. EDX study of Mo-Fe-C powder after MW treatment: SEM image (a); EDX spectrum of this area (b); element composition (c) and maps of molybdenum (d), iron (e), carbon (f), and oxygen (g) distributions.

SEM images of initial sample of Mo/C powder

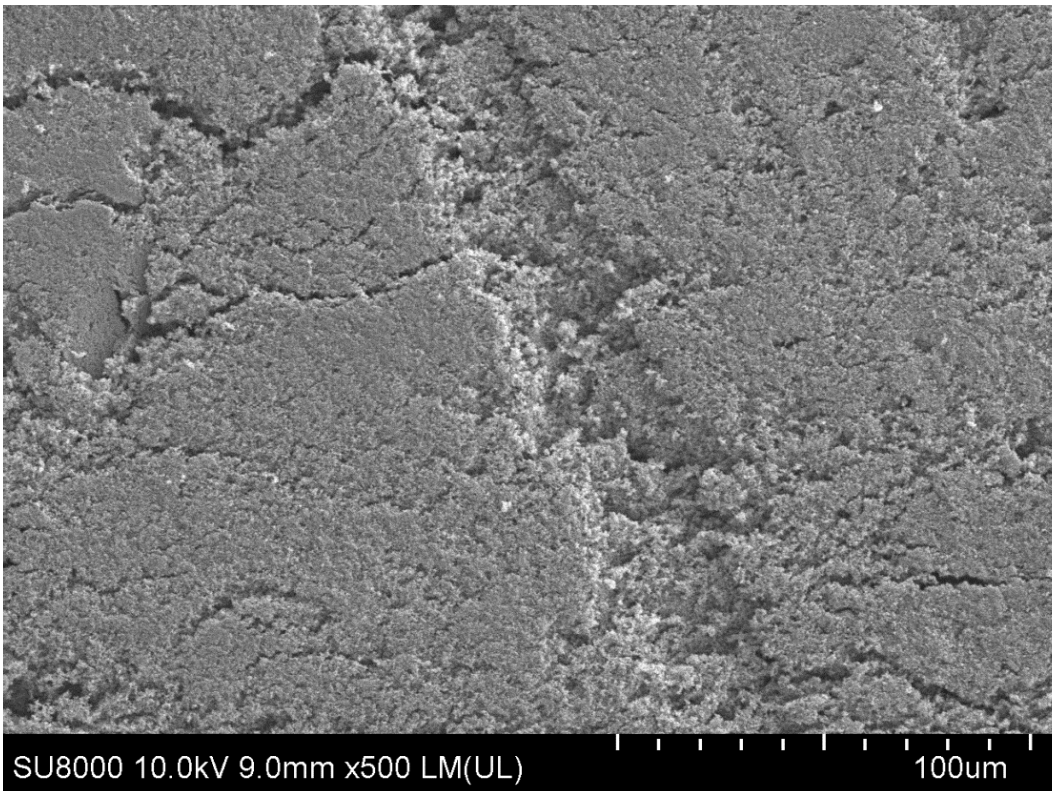

Figure S47. SEM image of initial sample of Mo/C powder.

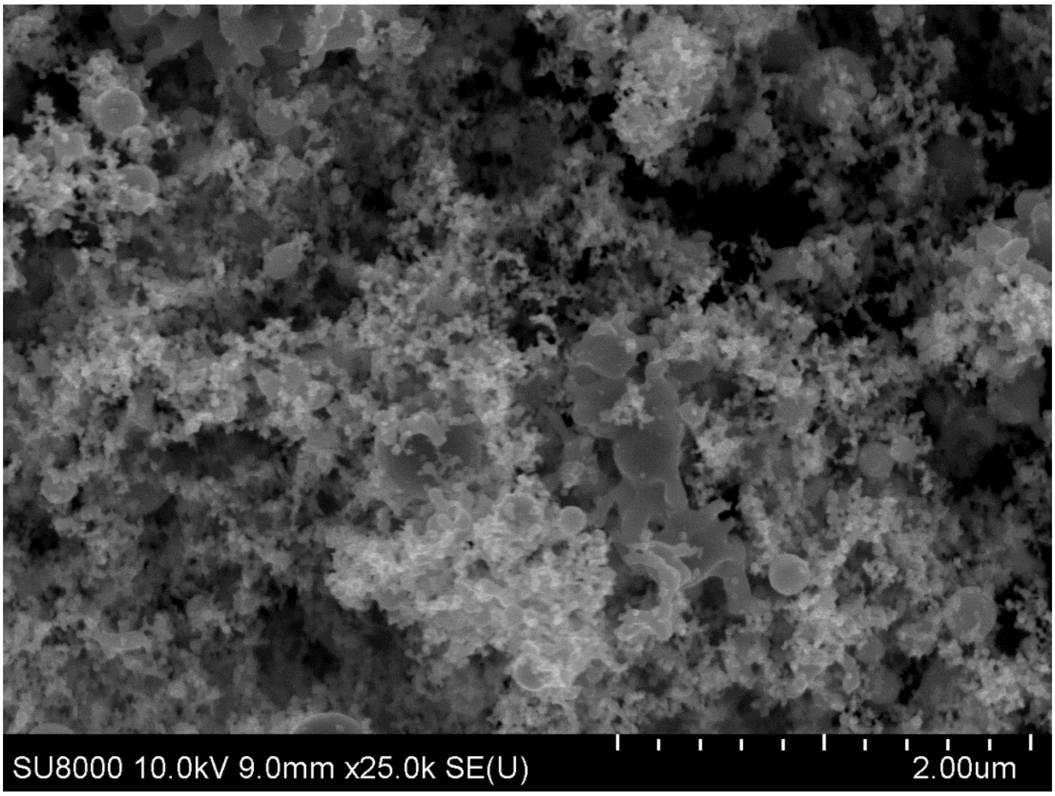

Figure S48. SEM image of initial sample of Mo/C powder.

EDX data of initial sample of Mo/C powder

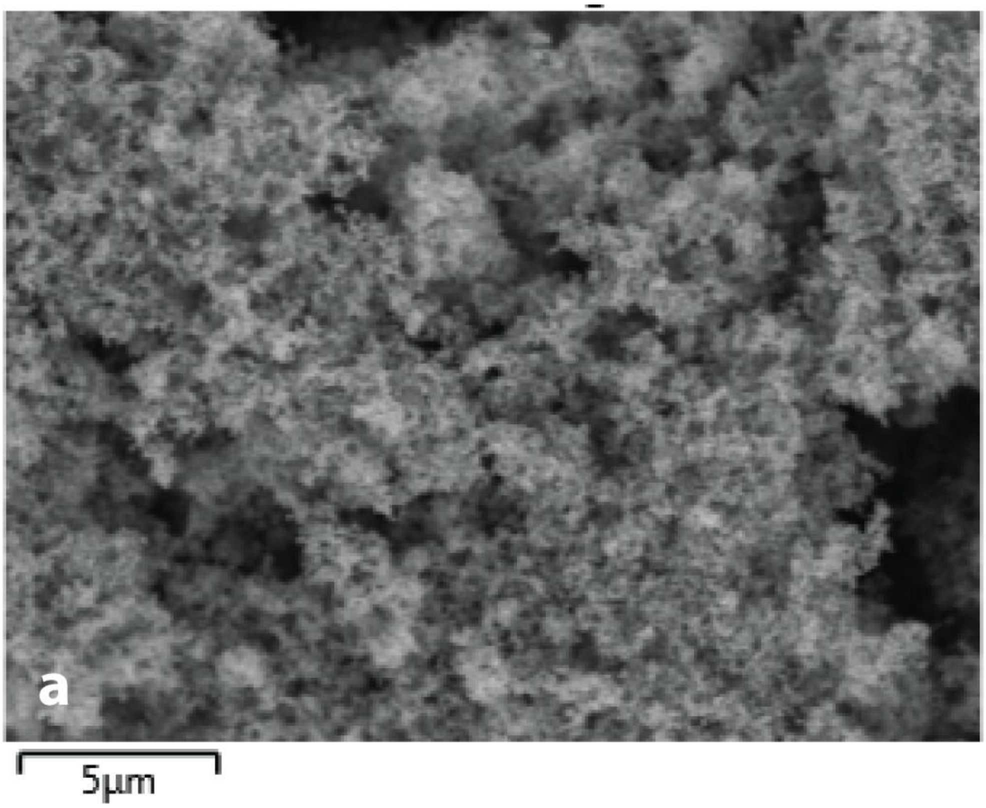

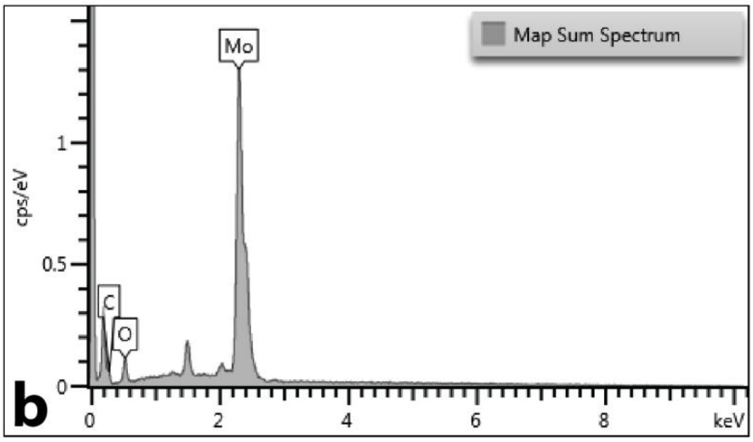

c

Element	Wt%	At%
Mo	90.4	57.7
C	4.3	21.8
O	5.3	20.5
Total:	100	100

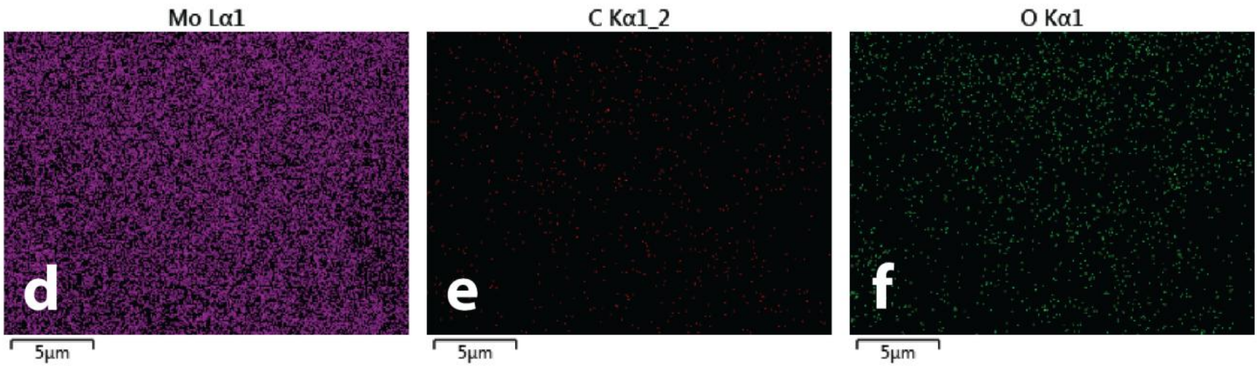

Figure S49. EDX study of initial Mo/C sample: SEM image (a); EDX spectrum of this area (b); element composition (c) and maps of molybdenum (d), carbon (e), oxygen (f) distributions. The increased oxygen content in the EDX analysis result by its presence in aluminum stub which fixed samples for SEM. In addition, an oxide film on the surface of the sample can be a source of the oxygen signal.

SEM images of Mo/C powder sample after MW treatment

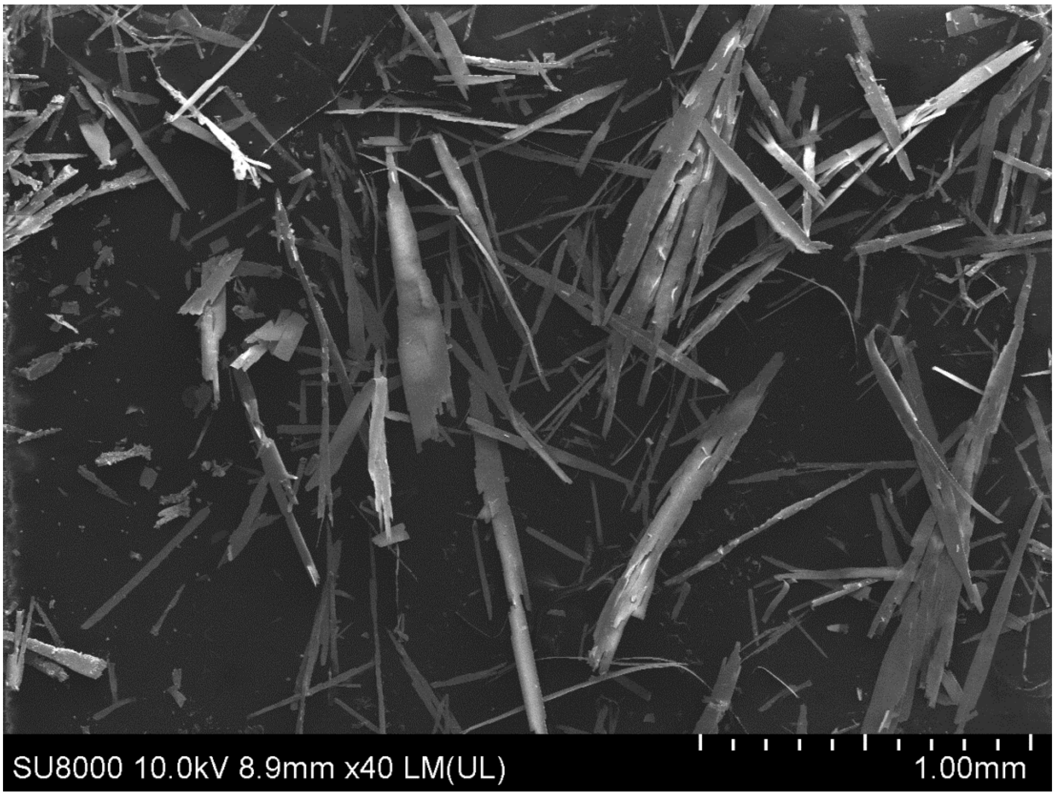

Figure S50. SEM image of Mo/C powder sample after MW treatment.

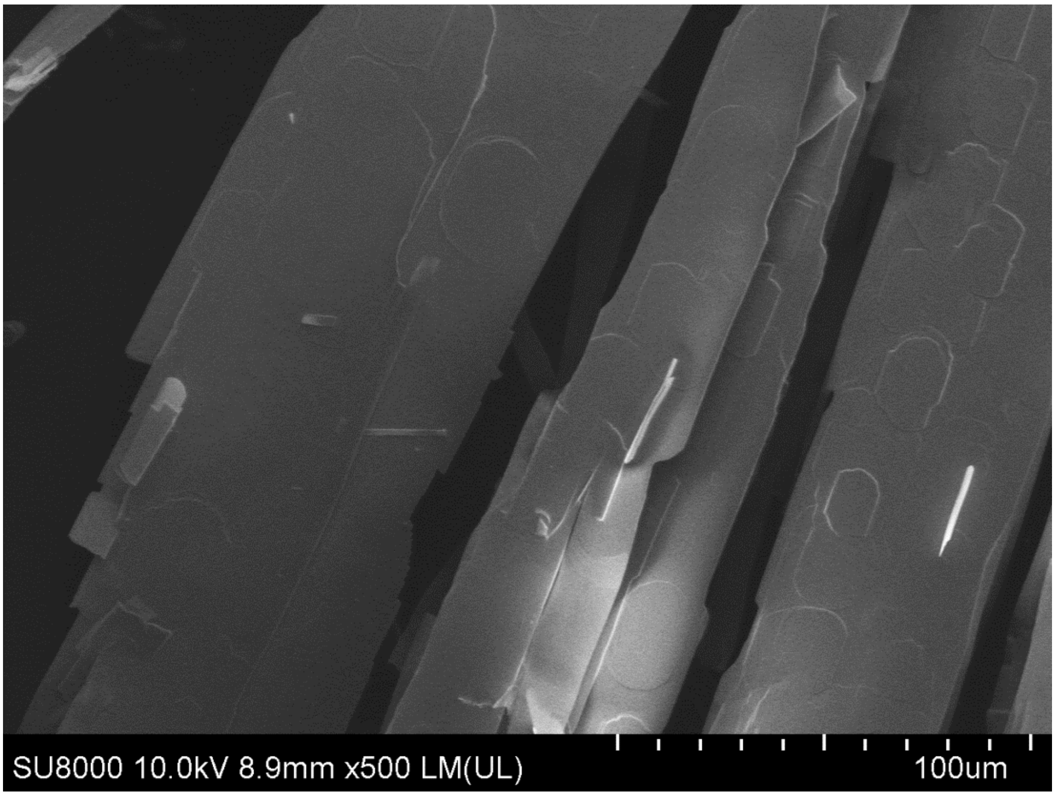

Figure S51. SEM image of Mo/C powder sample after MW treatment.

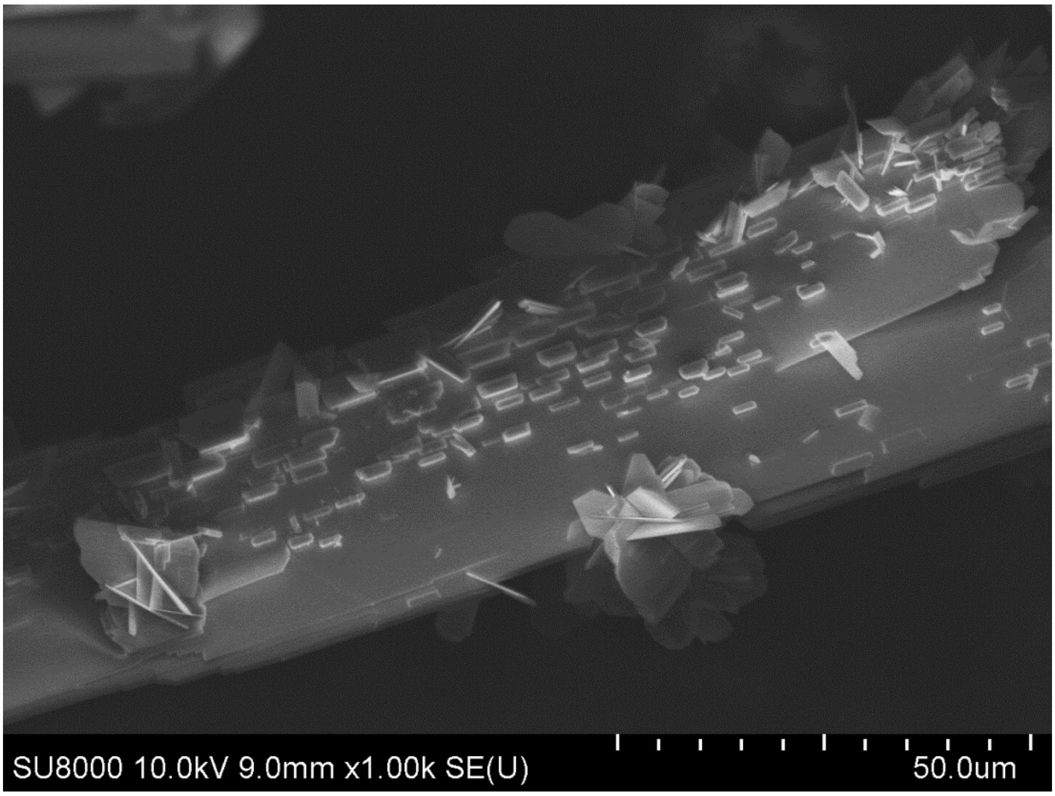

Figure S52. SEM image of Mo/C powder sample after MW treatment.

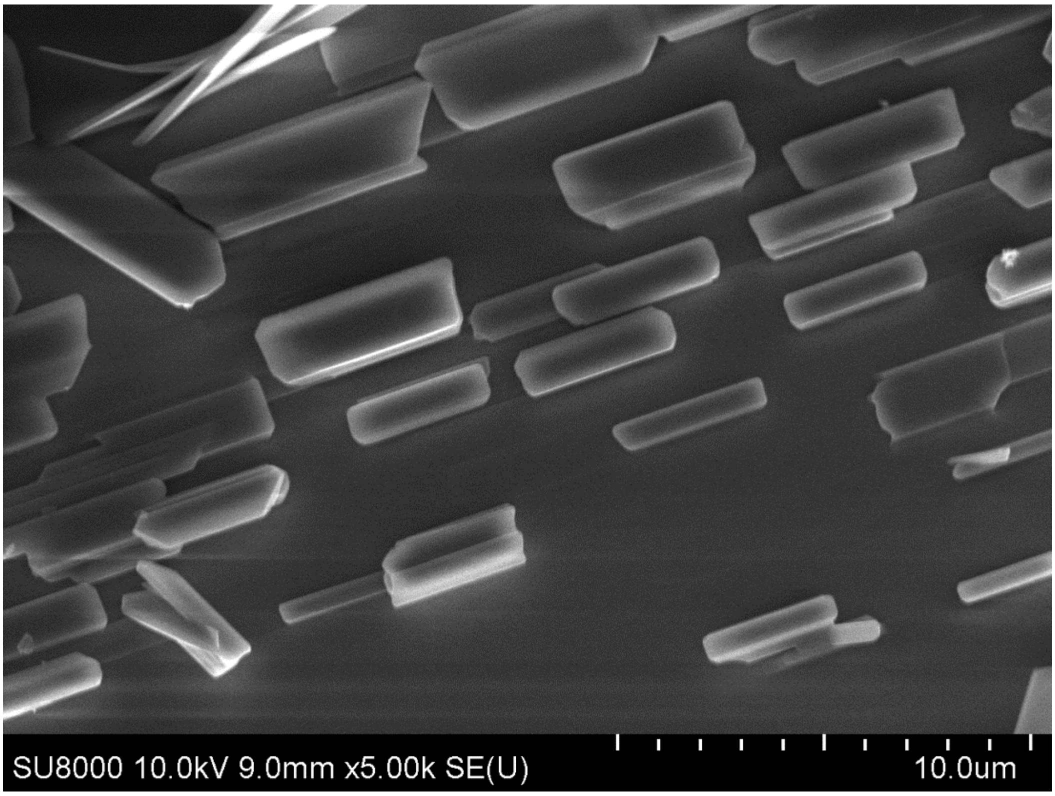

Figure S53. SEM image of Mo/C powder sample after MW treatment.

EDX data of Mo/C powder sample after MW treatment

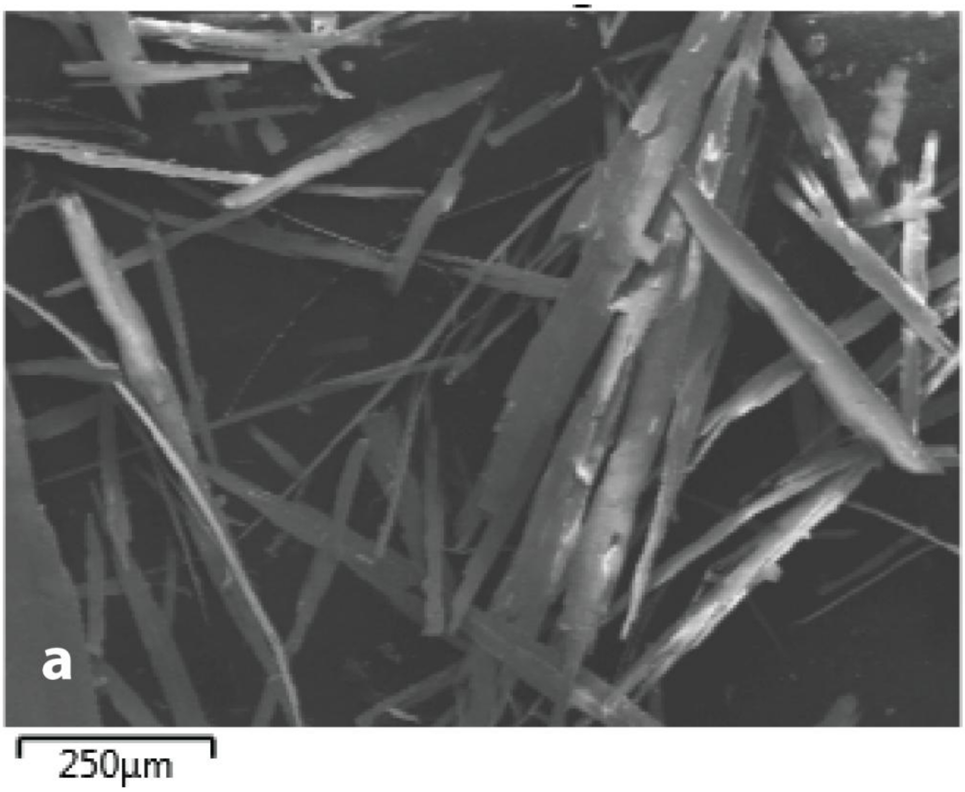

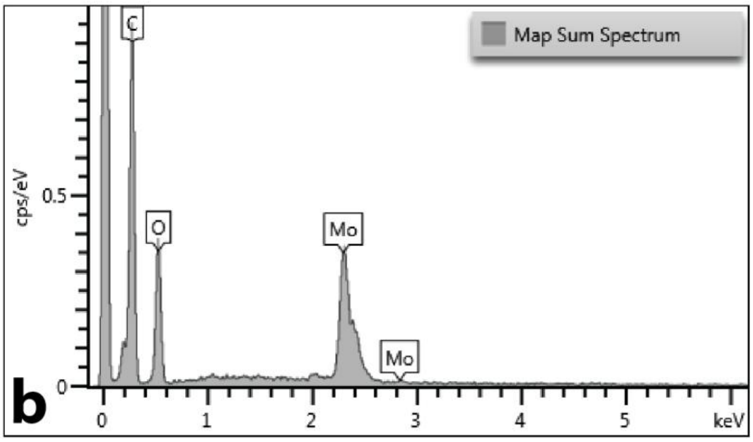

c

Element	Wt%	At%
Mo	62	22
O	38	78
Total:	100	100

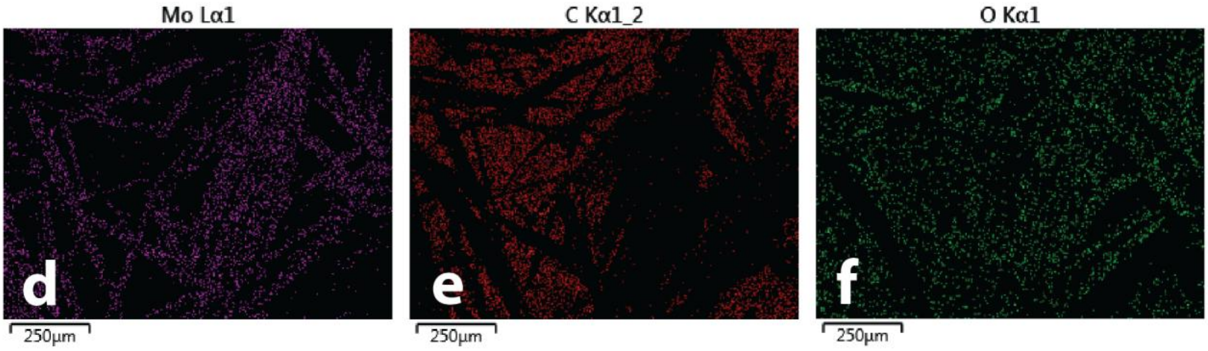

Figure S54. EDX study of Mo/C powder sample after MW treatment: SEM image (a); EDX spectrum of this area (b); element composition (c) and maps of molybdenum (d), carbon (e), oxygen (f) distributions.

SEM images of initial sample of MoS_2 powder

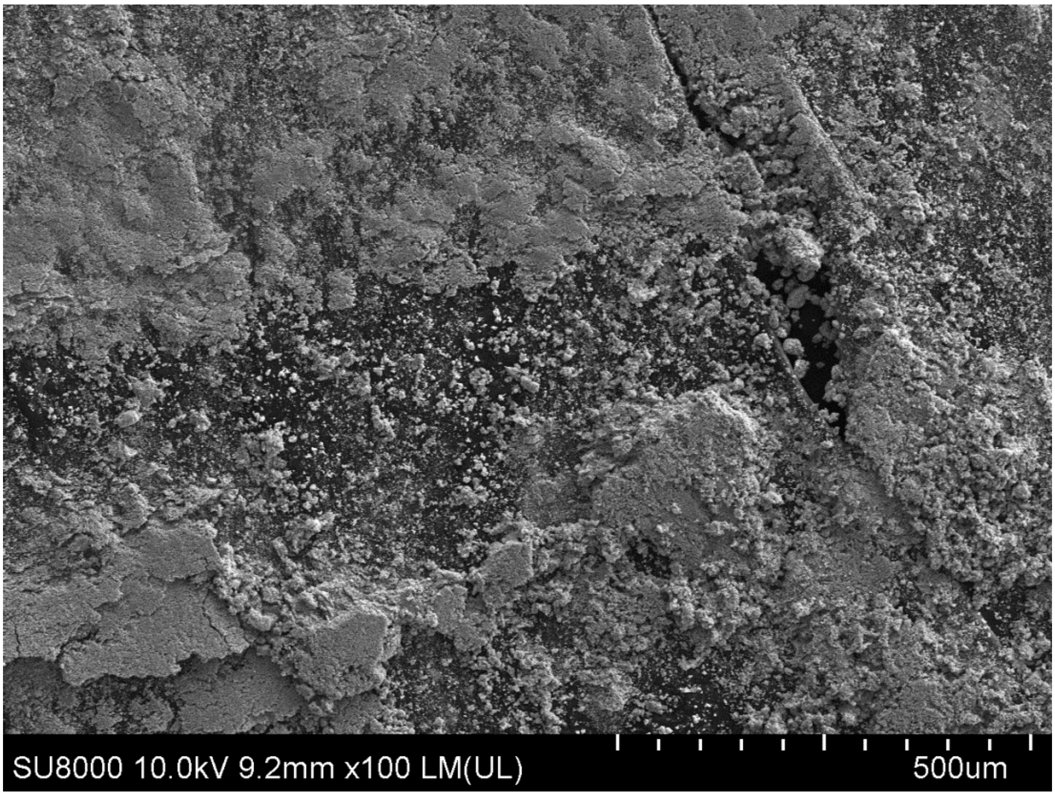

Figure S55. SEM image of initial sample of MoS_2 powder.

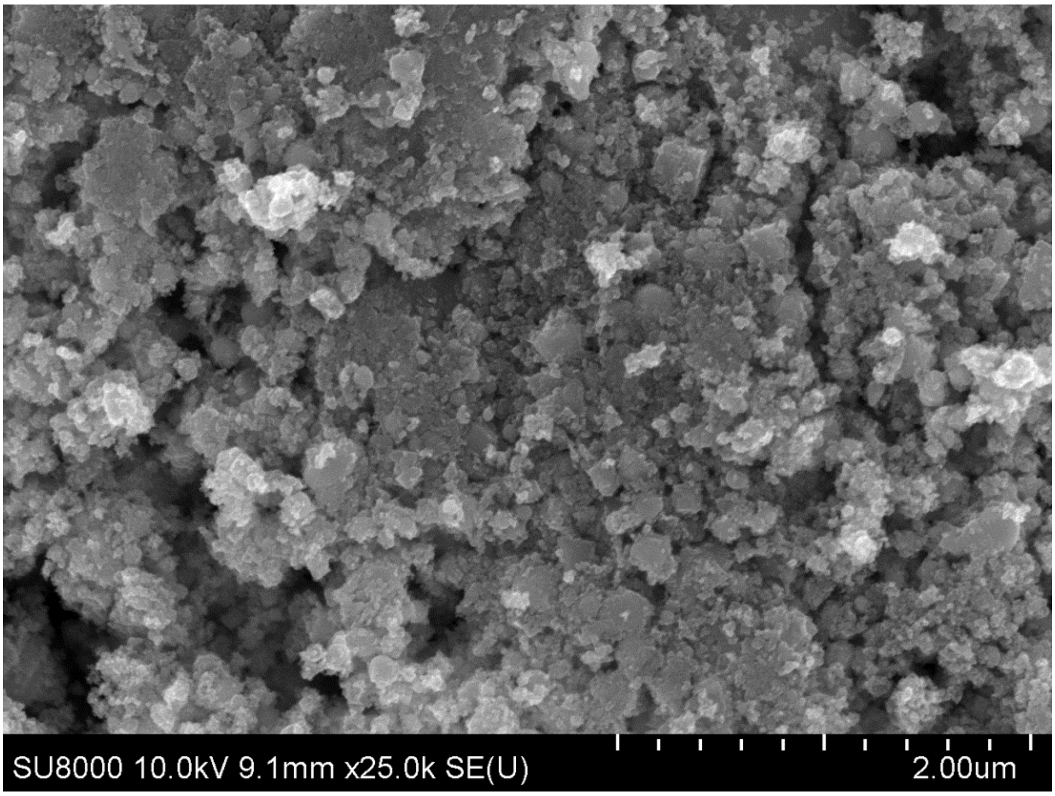

Figure S56. SEM image of initial sample of MoS_2 powder.

EDX data of initial sample of MoS_2 powder

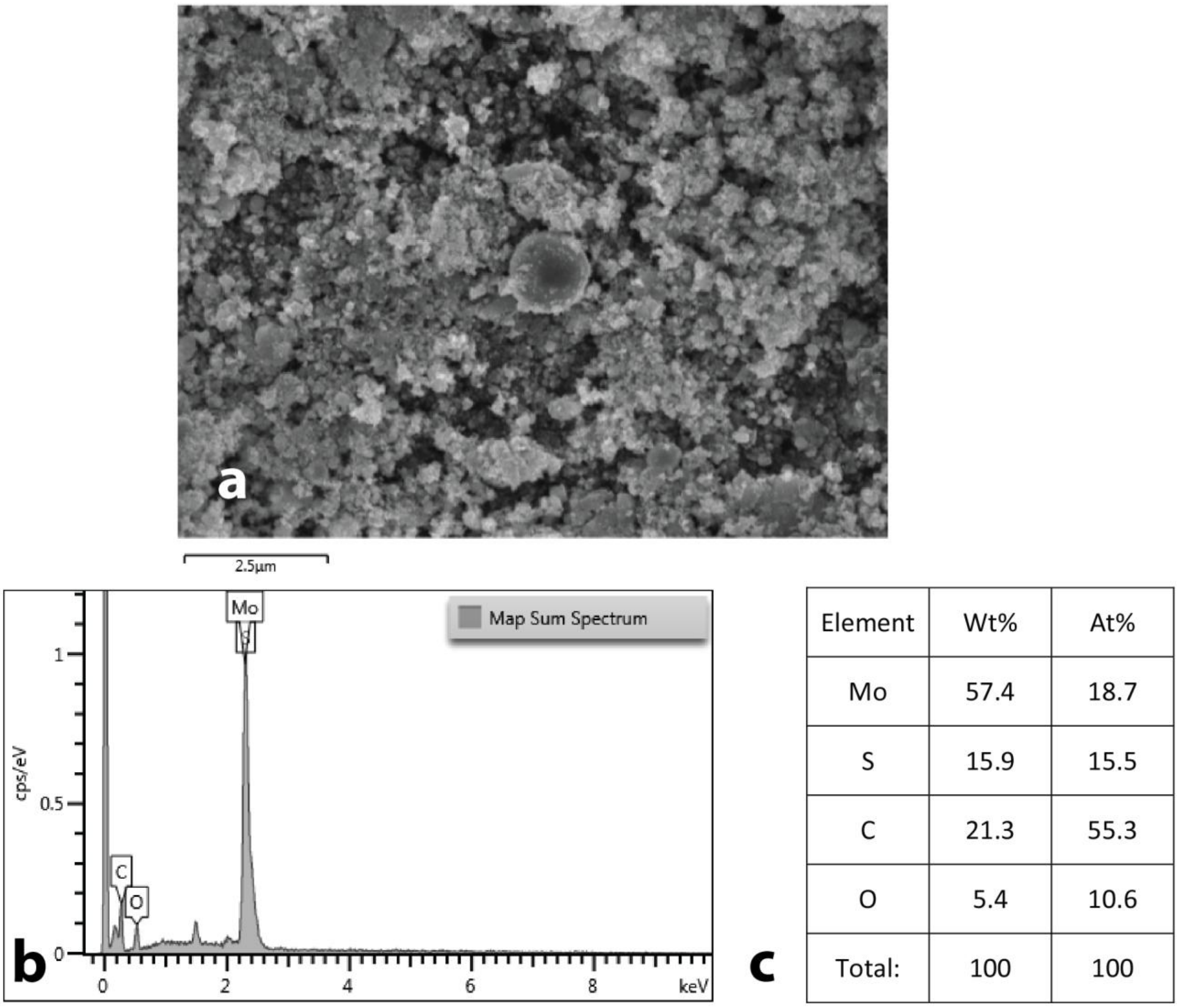

c

Element	Wt%	At%
Mo	57.4	18.7
S	15.9	15.5
C	21.3	55.3
O	5.4	10.6
Total:	100	100

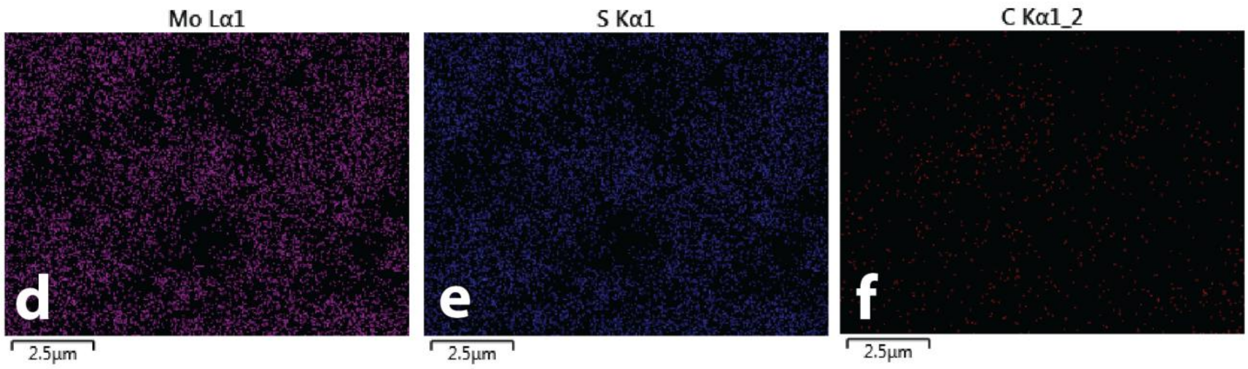

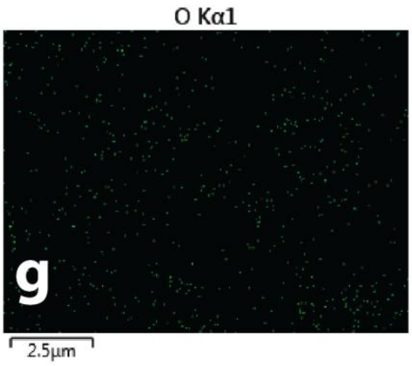

Figure S57. EDX study of initial MoS_2 sample: SEM image (a); EDX spectrum of this area (b); element composition (c) and maps of molybdenum (d), sulfur (e), carbon (f), and oxygen (g) distributions. The original nanopowder does not contain carbon. The presence of carbon and the increased oxygen content in the EDX analysis result by their presence in the carbon tape and stub which fixed samples for SEM.

SEM images of MoS_2 powder sample after MW treatment

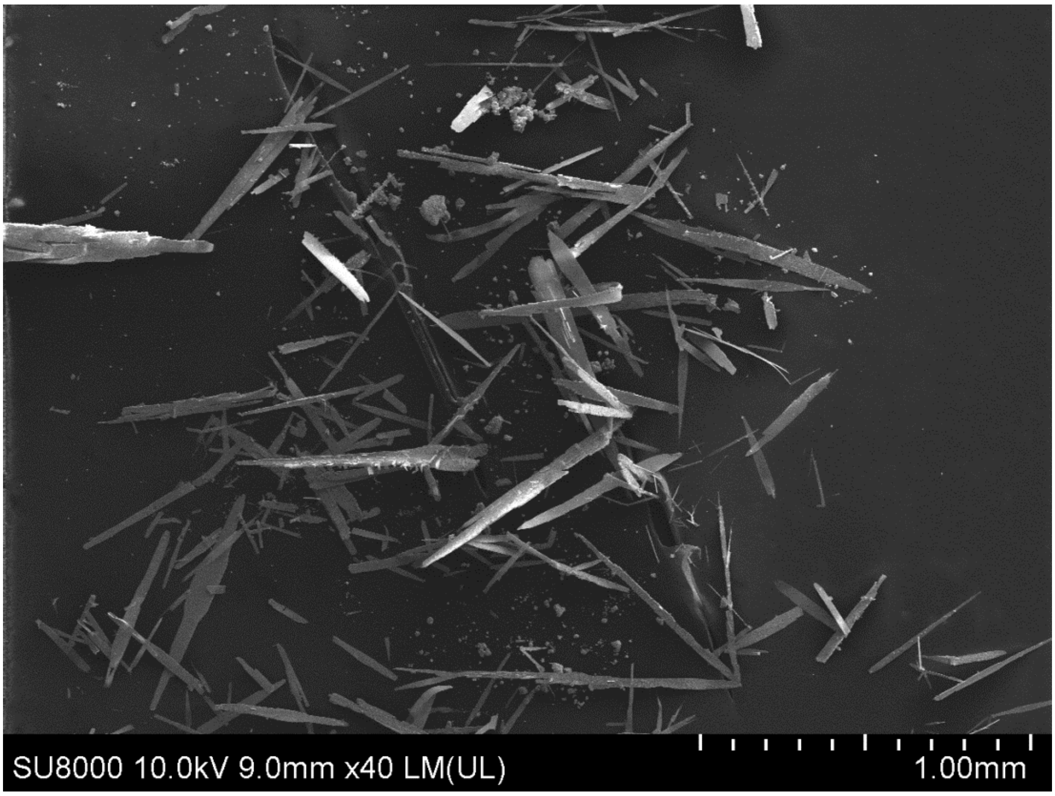

Figure S58. SEM image of MoS_2 powder sample after MW treatment.

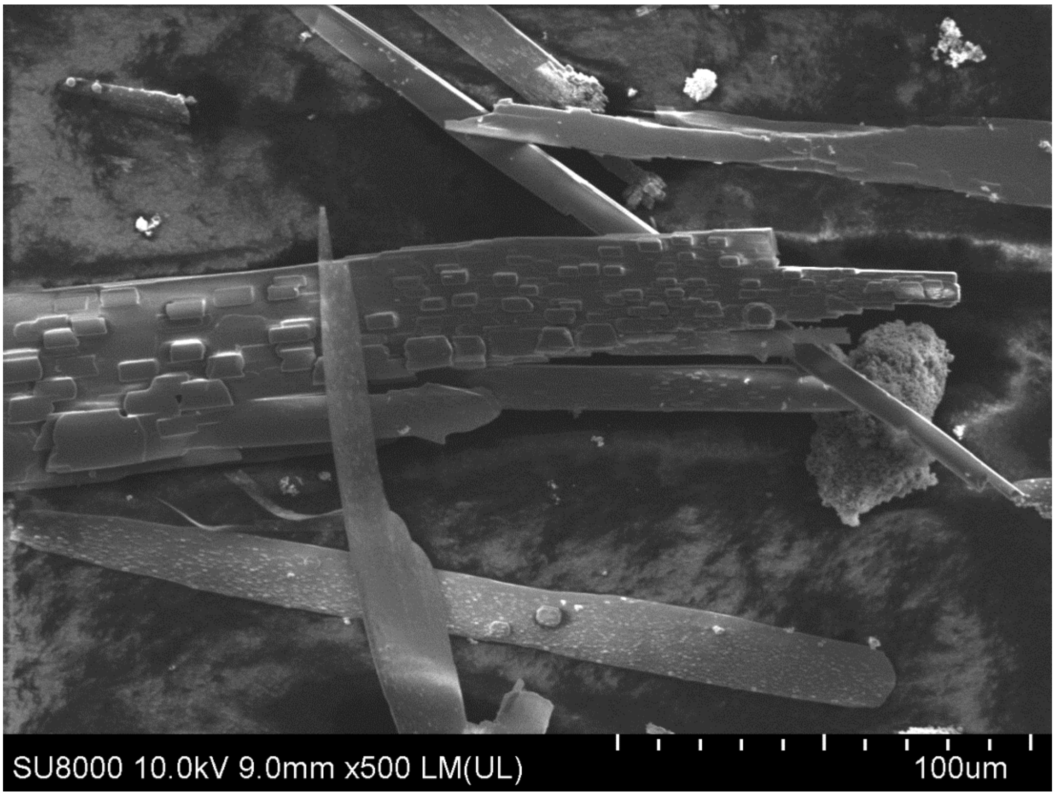

Figure S59. SEM image of MoS_2 powder sample after MW treatment.

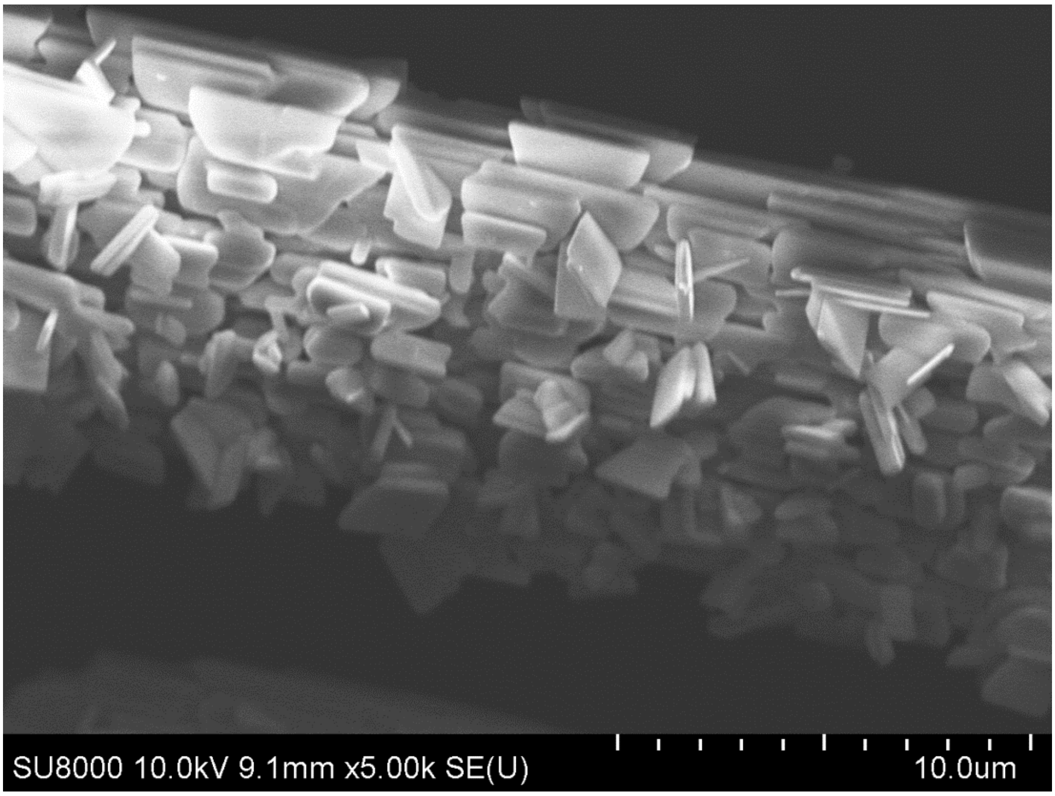

Figure S60. SEM image of MoS_2 powder sample after MW treatment.

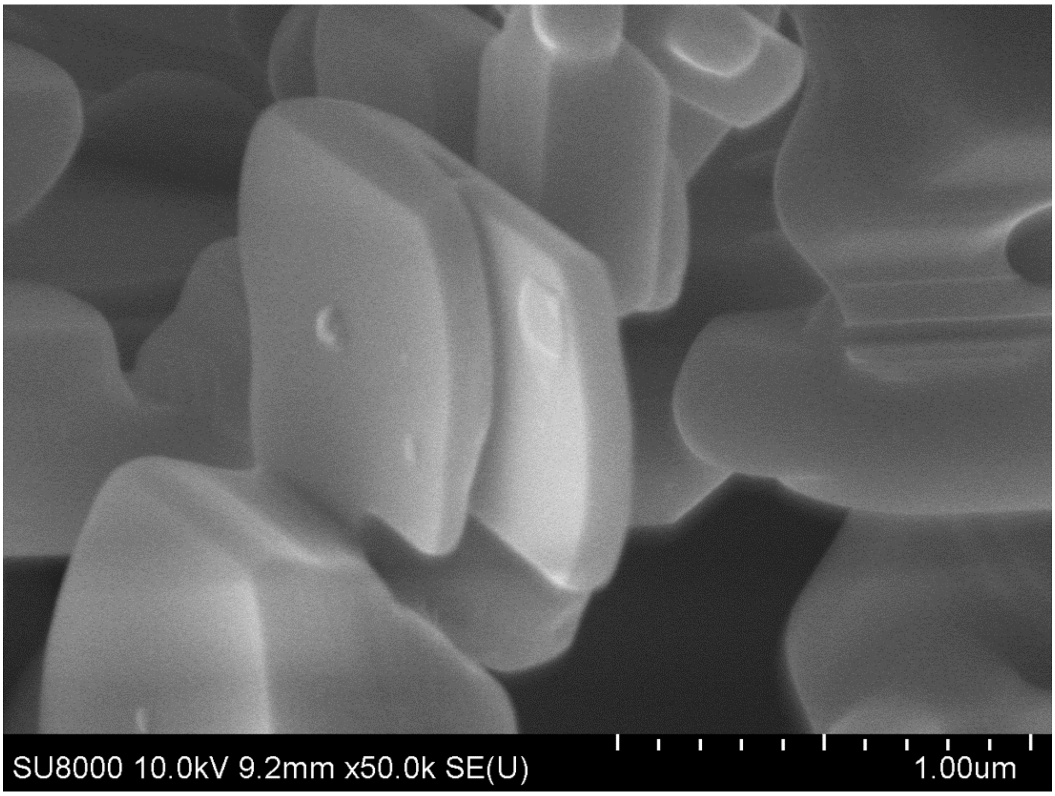

Figure S61. SEM image of MoS_2 powder sample after MW treatment.

EDX data of MoS_2 powder sample after MW treatment

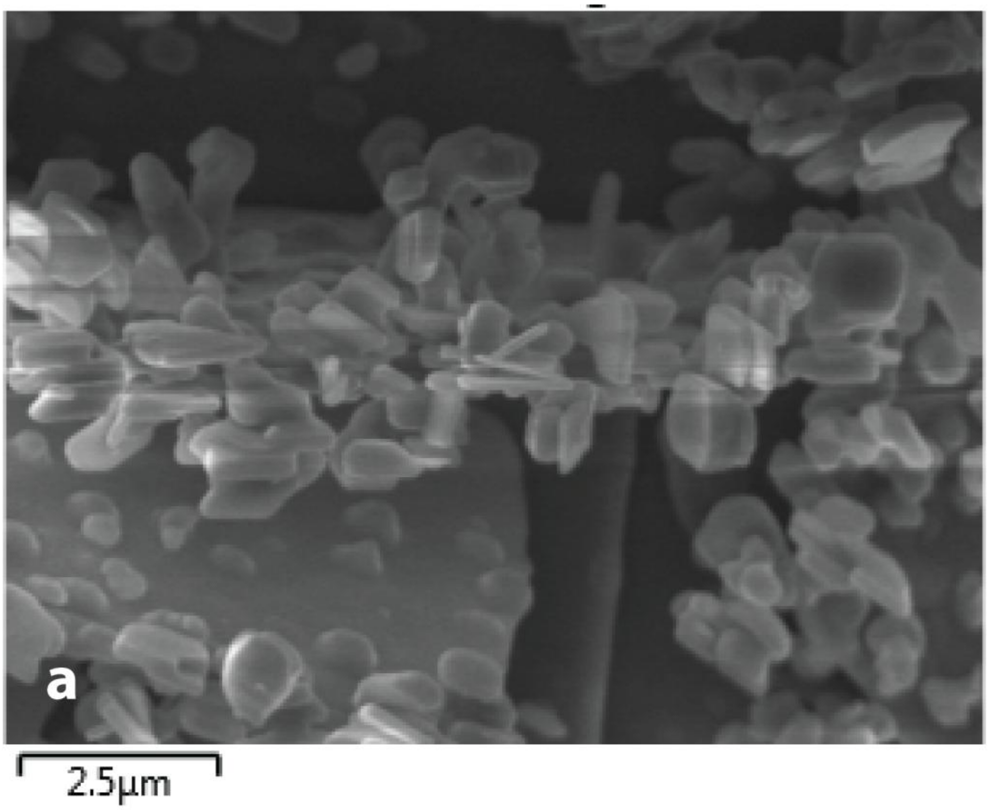

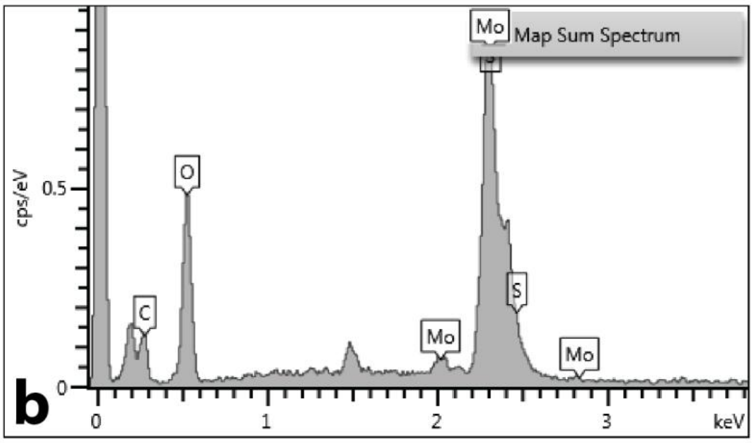

c

Element	Wt%	At%
Mo	67.7	24.1
S	0	0
C	9.5	27.2
O	22.8	48.7
Total:	100	100

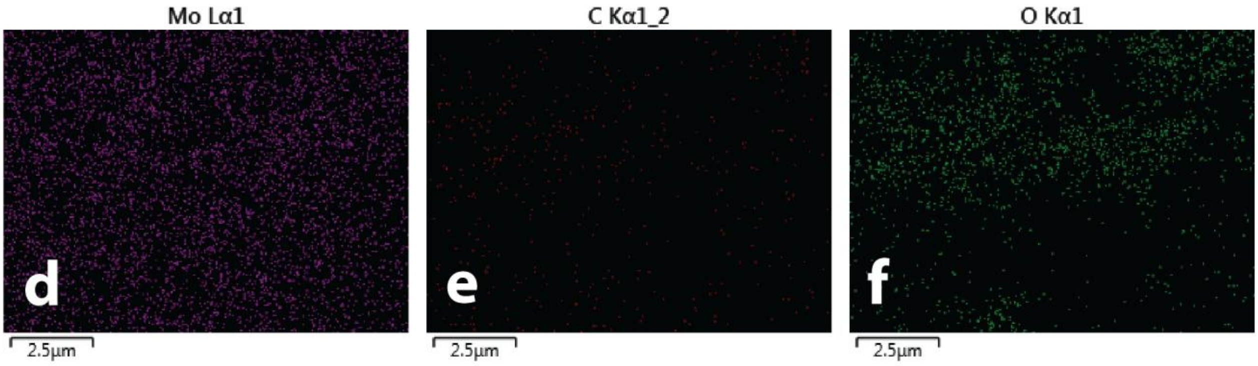

Figure S62. EDX study of MoS_2 powder after MW treatment: SEM image (a); EDX spectrum of this area (b); element composition (c) and maps of molybdenum (d), carbon (e) and oxygen (f) distributions.

SEM images of initial sample of WC powder

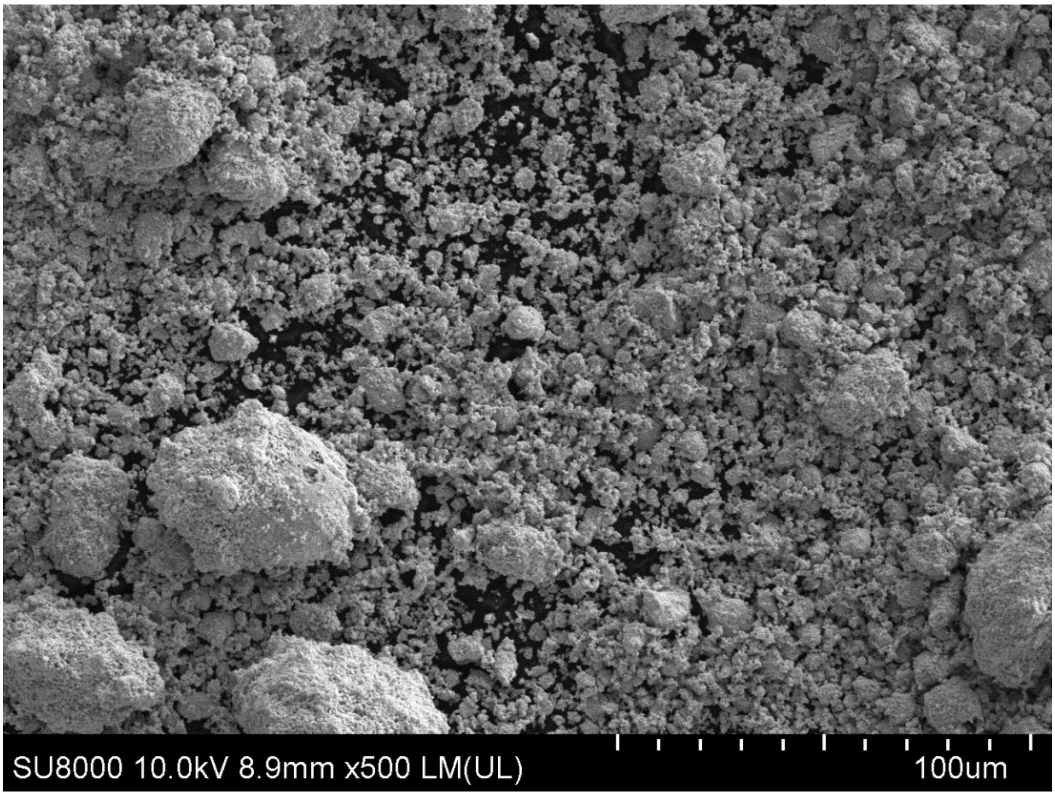

Figure S63. SEM image of initial sample of WC powder.

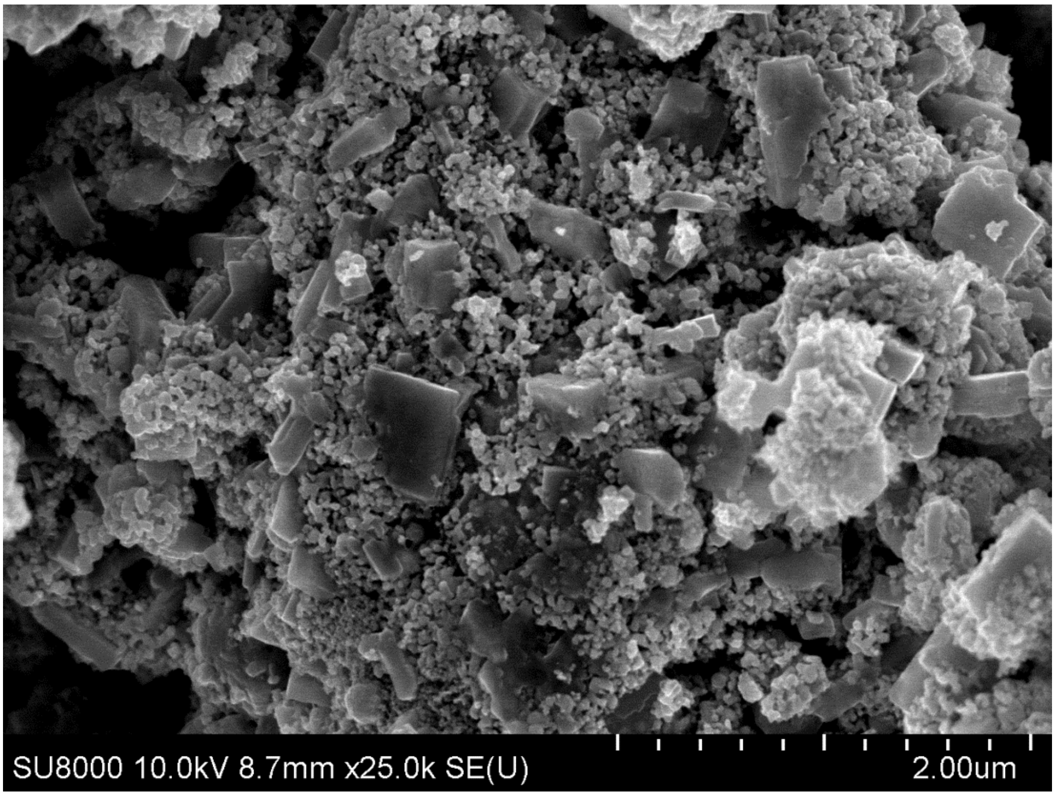

Figure S64. SEM image of initial sample of WC powder.

EDX data of initial sample of WC powder

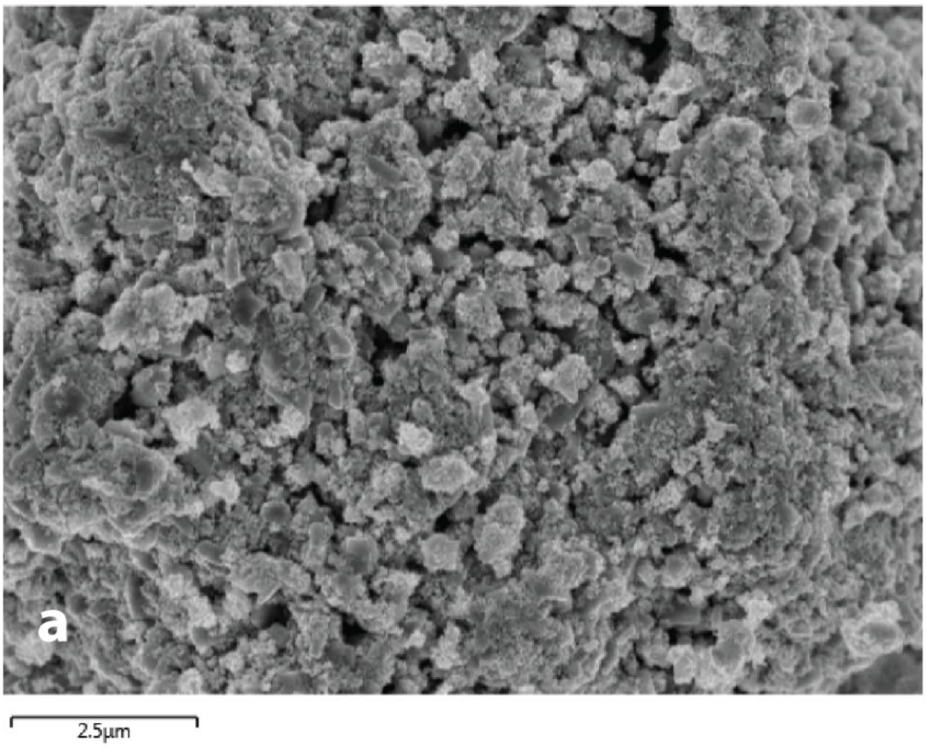

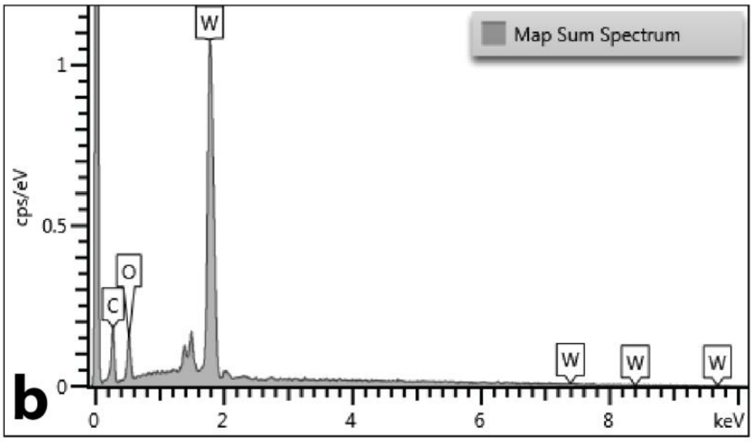

c

Element	Wt%	At%
W	83.0	25.8
C	11.5	54.6
O	9.5	19.6
Total:	100	100

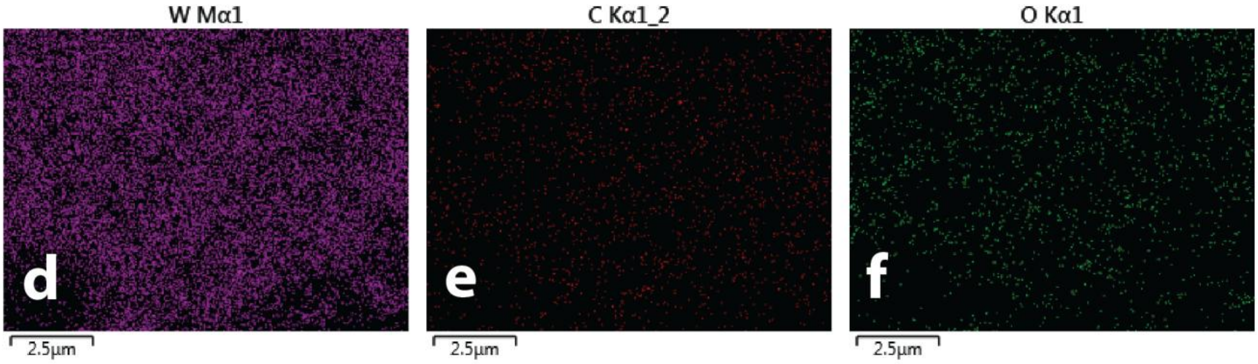

Figure S65. EDX study of initial WC sample: SEM image (a); EDX spectrum of this area (b); element composition (c) and maps of tungsten (d), carbon (e), and oxygen (f) distributions. The increased oxygen content in the EDX analysis result by its presence in aluminum stub which fixed samples for SEM. In addition, an oxide film on the surface of the sample can be a source of the oxygen signal.

SEM images of WC powder sample after MW treatment

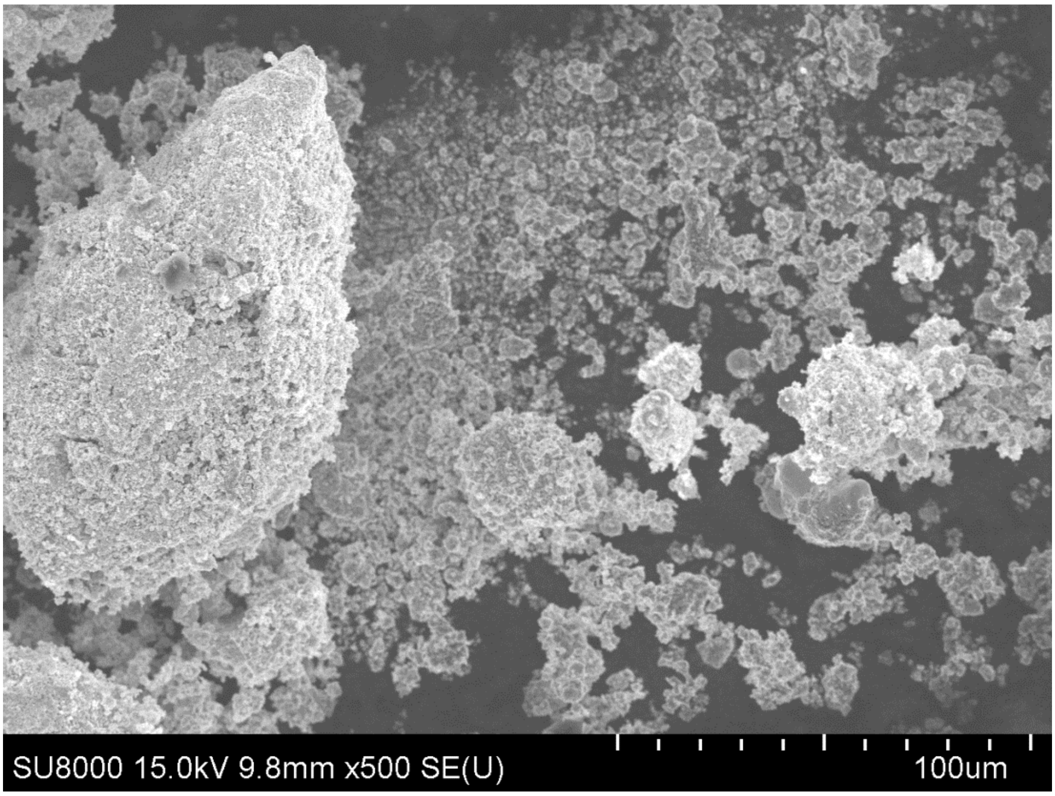

Figure S66. SEM image of WC powder sample after MW treatment.

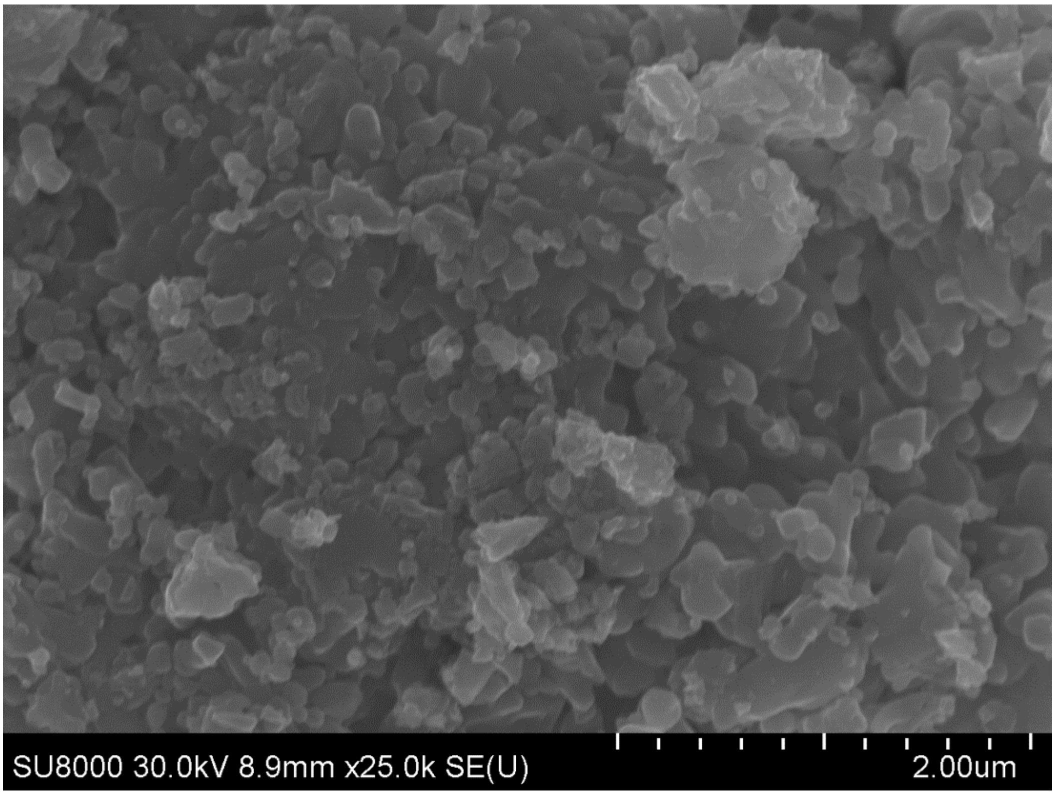

Figure S67. SEM image of WC powder sample after MW treatment.

EDX data of WC powder sample after MW treatment

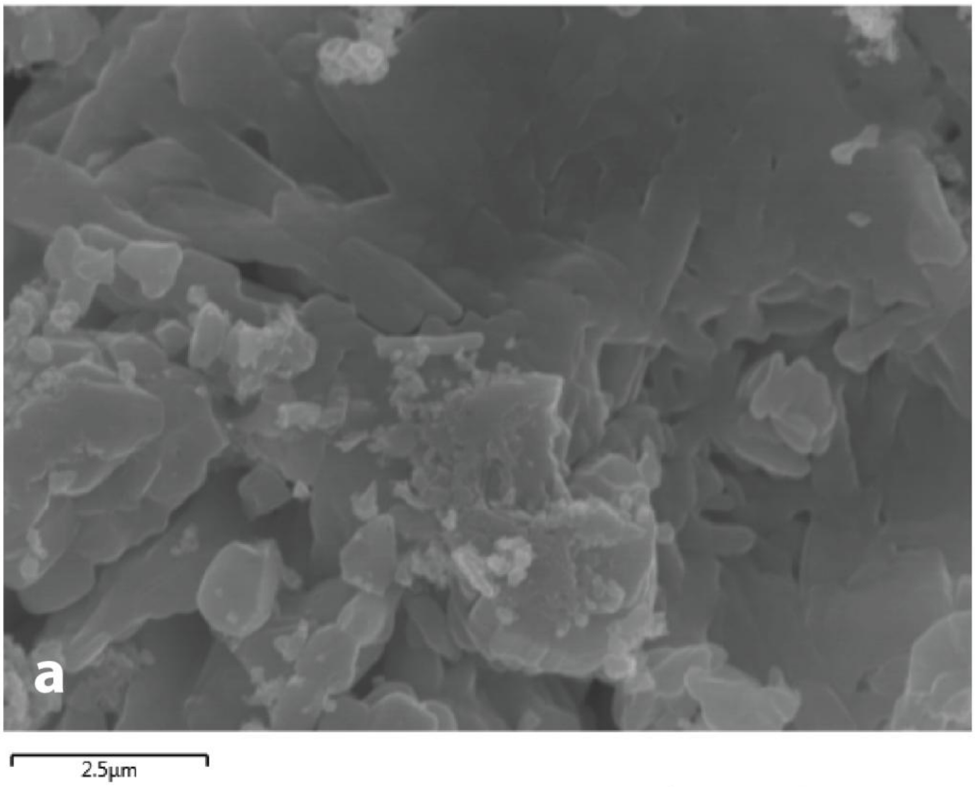

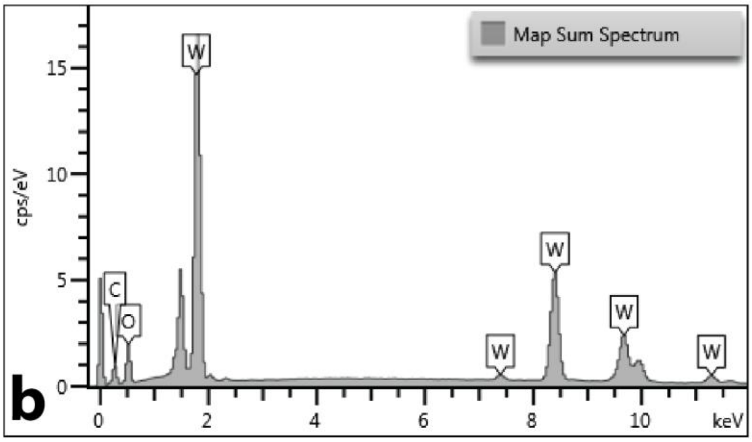

c

Element	Wt%	At%
W	64.1	11.8
C	19.2	47.4
O	16.8	40.7
Total:	100	100

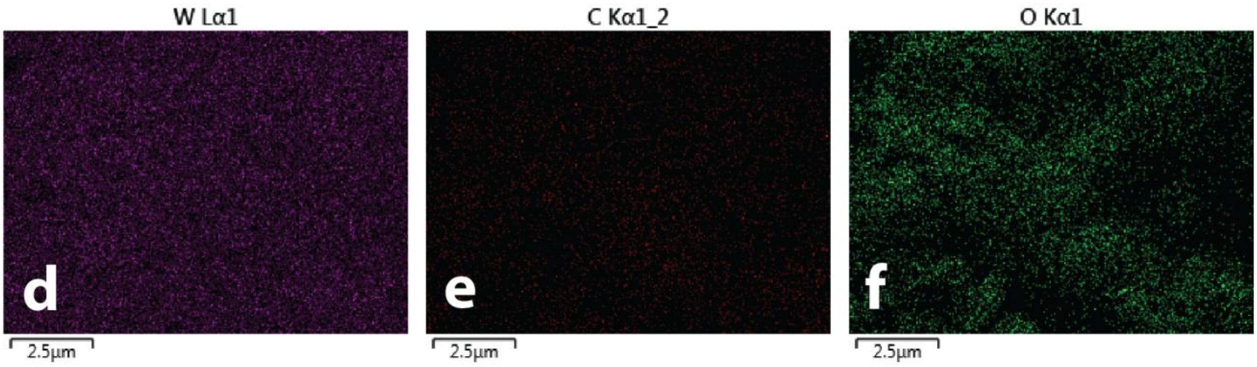

Figure S68. EDX study of WC powder after MW treatment: SEM image (a); EDX spectrum of this area (b); element composition (c) and maps of tungsten (d), carbon (e), and oxygen (f) distributions.

SEM images of initial sample of TiC powder

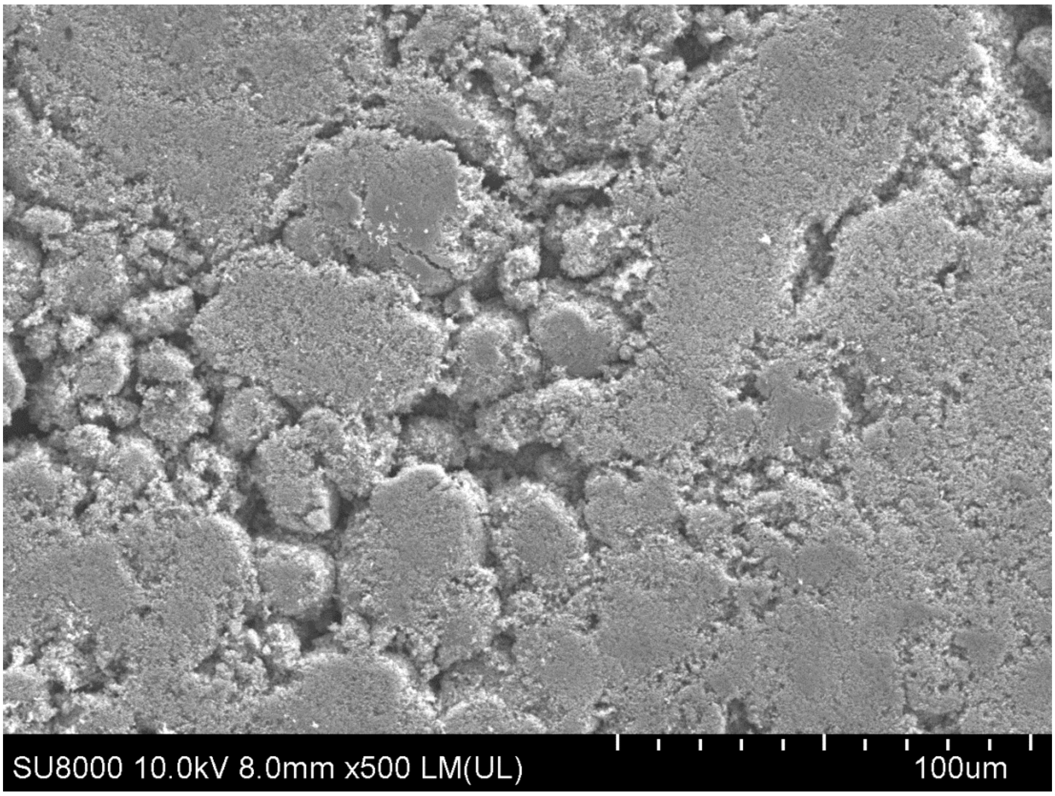

Figure S69. SEM image of initial sample of TiC powder.

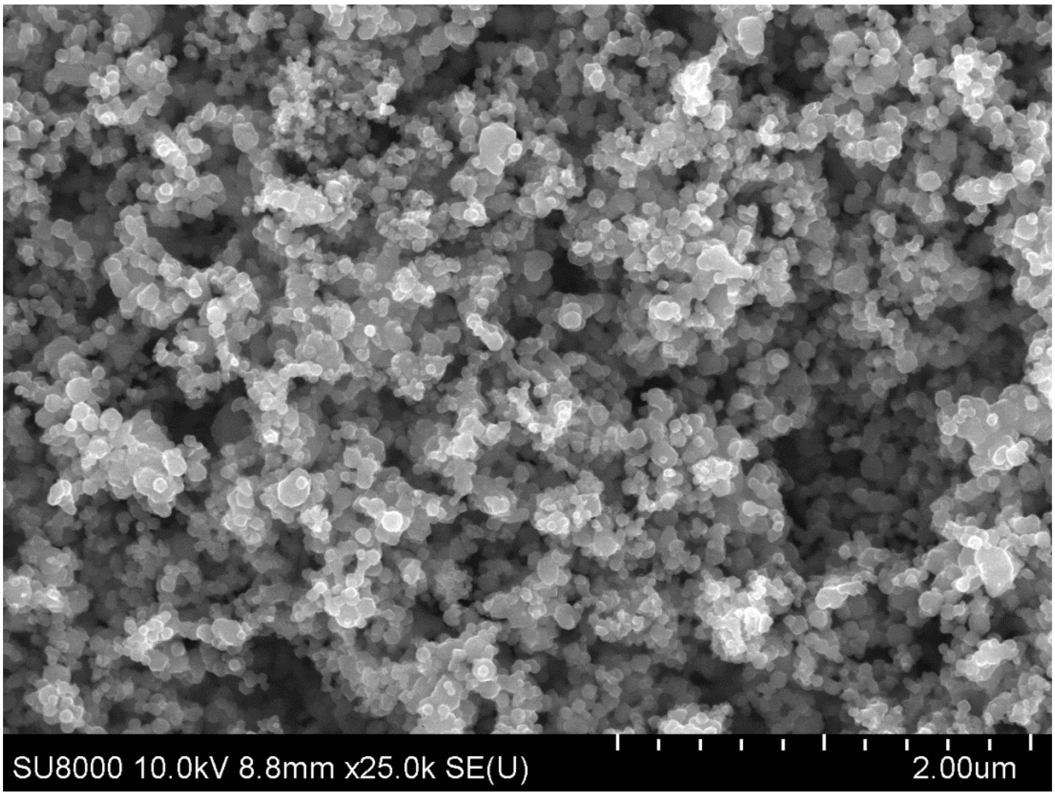

Figure S70. SEM image of initial sample of TiC powder.

EDX data of initial sample of TiC powder

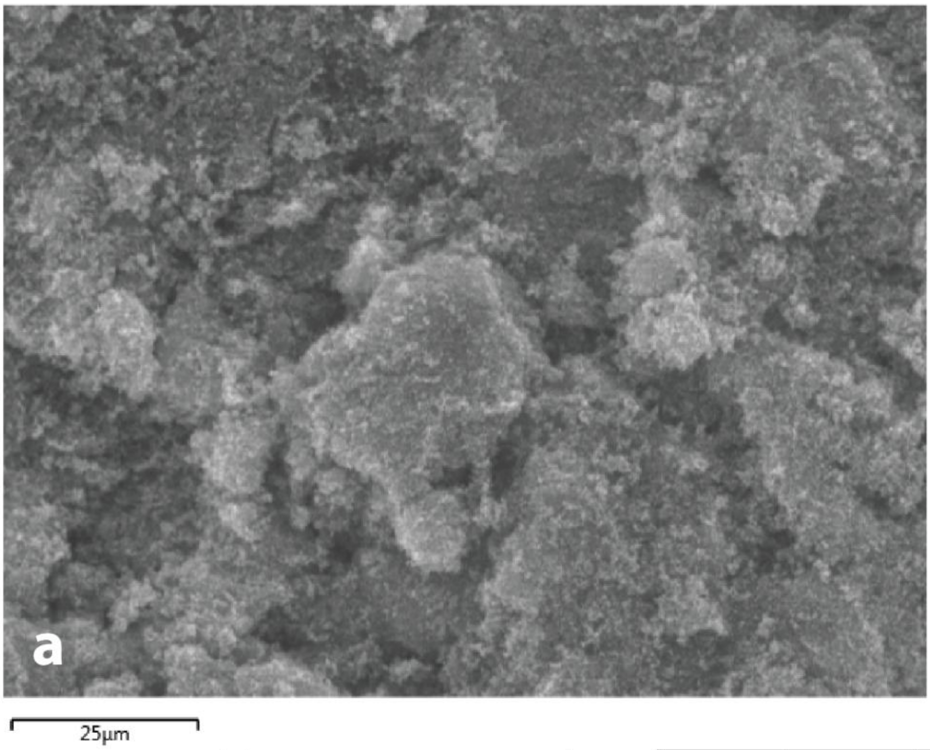

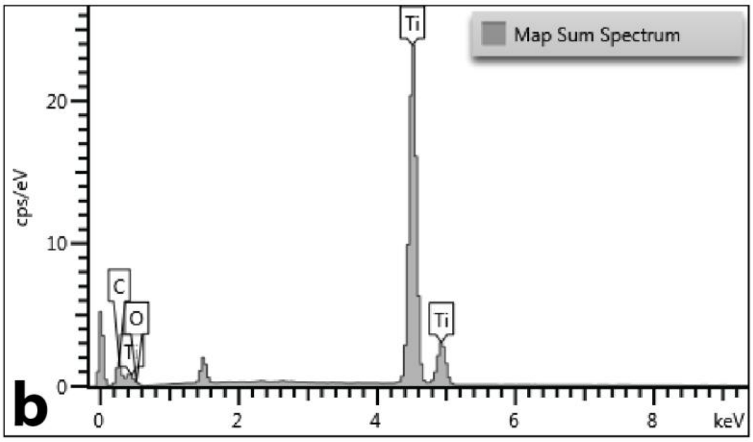

c

Element	Wt%	At%
Ti	67.5	35.0
C	28.2	58.3
O	4.3	6.6
Total:	100	100

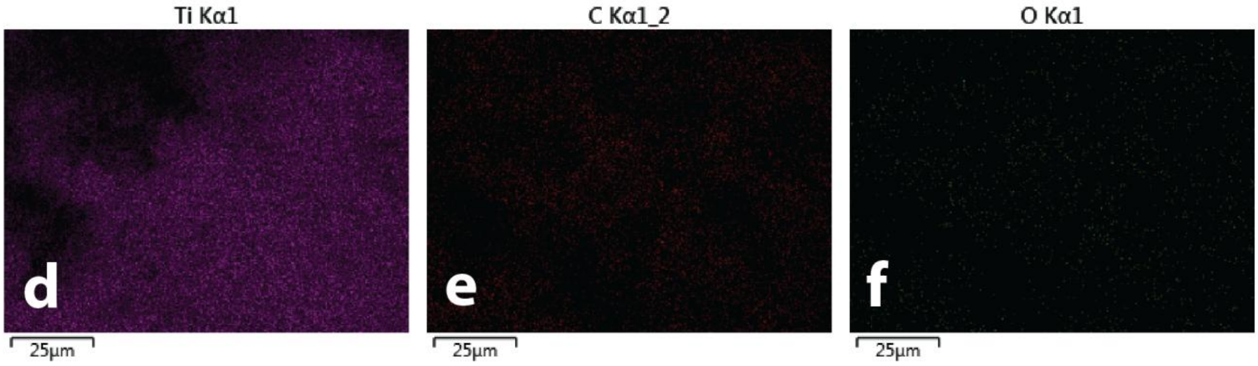

Figure S71. EDX study of initial TiC sample: SEM image (a); EDX spectrum of this area (b); element composition (c) and maps of titanium (d), carbon (e), and oxygen (f) distributions. The increased oxygen content in the EDX analysis result by its presence in aluminum stub which fixed samples for SEM. In addition, an oxide film on the surface of the sample can be a source of the oxygen signal.

SEM images of TiC powder sample after MW treatment

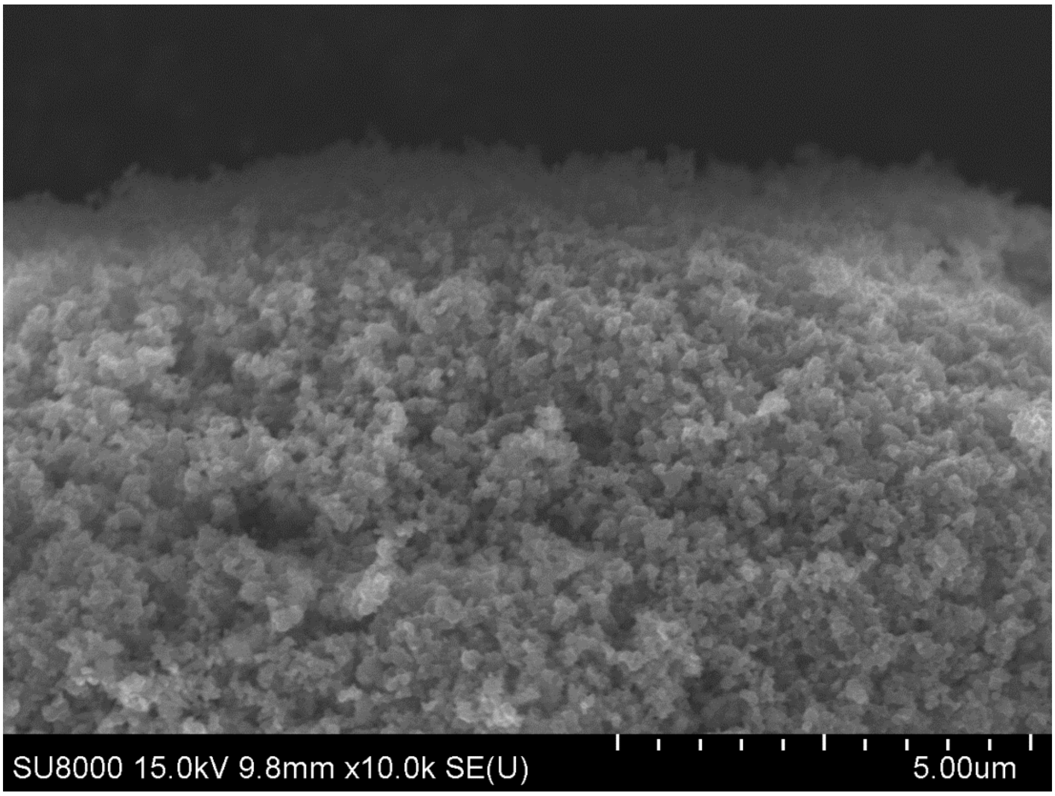

Figure S72. SEM image of TiC powder sample after MW treatment.

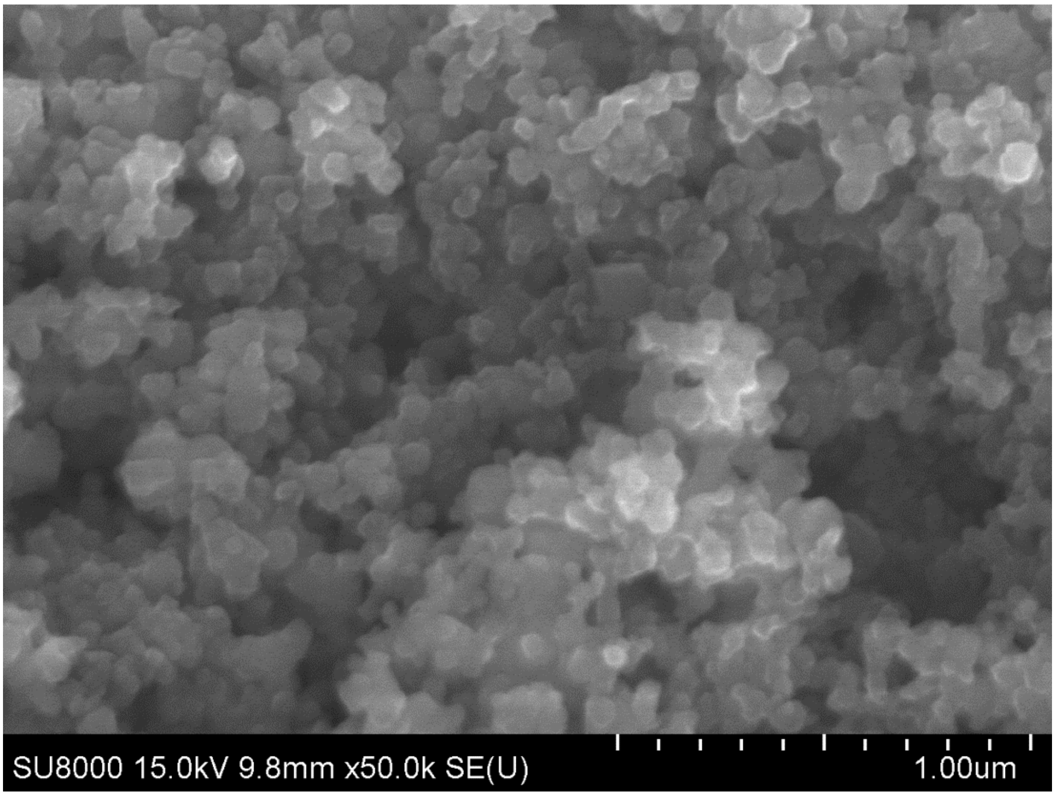

Figure S73. SEM image of TiC powder sample after MW treatment.

EDX data of TiC powder sample after MW treatment

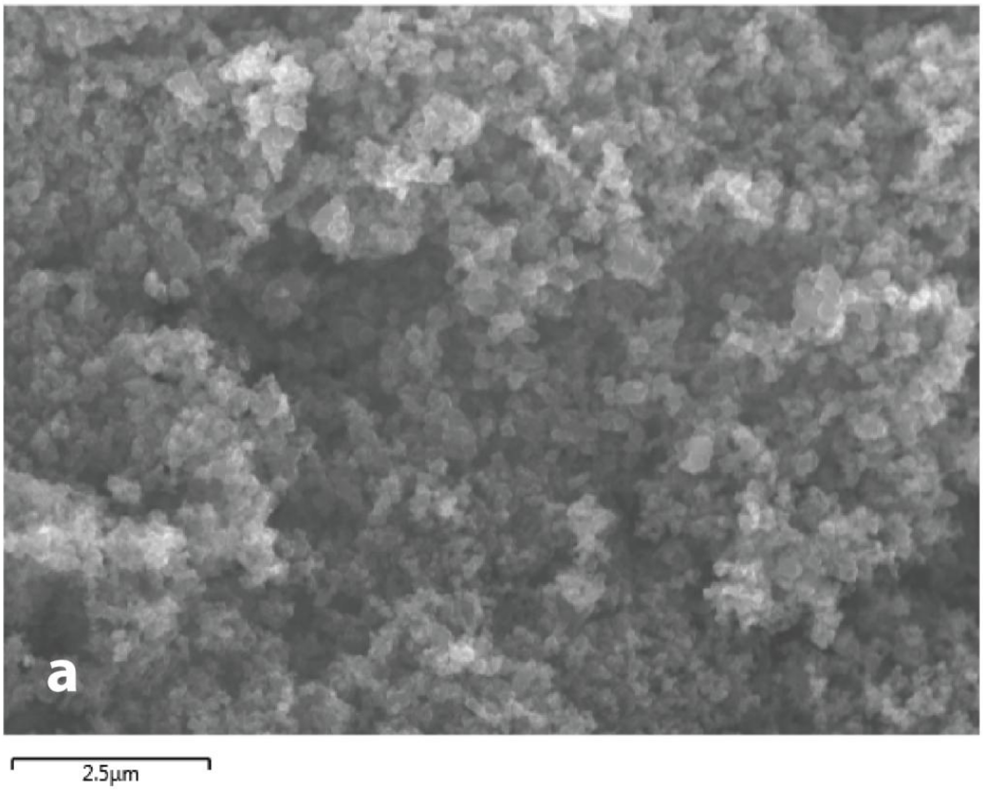

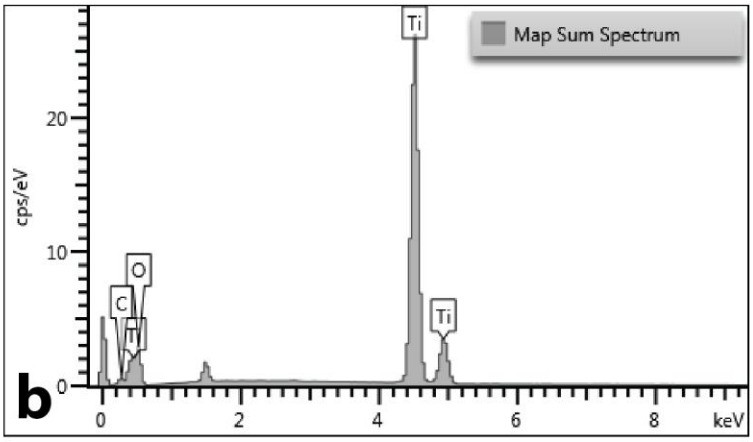

c

Element	Wt%	At%
Ti	49.3	23.9
C	4.7	9.1
O	46.0	67.0
Total:	100	100

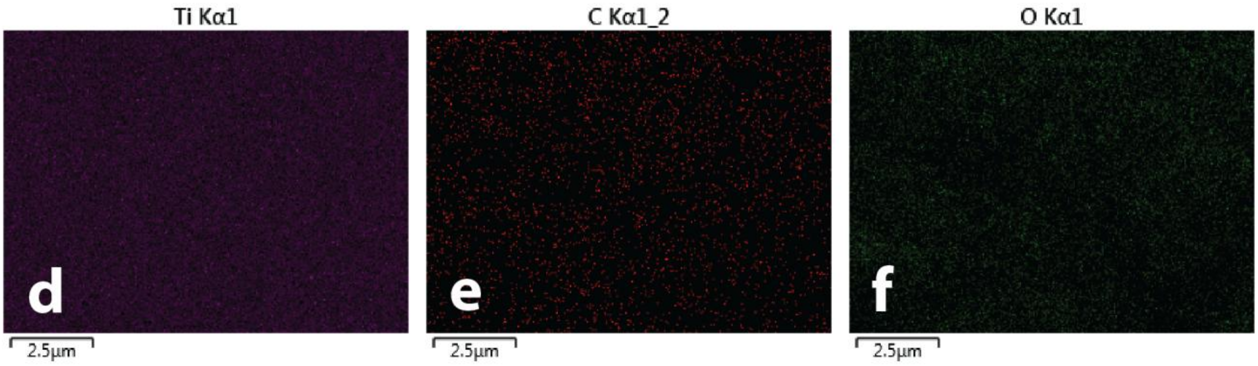

Figure S74. EDX study of TiC powder after MW treatment: SEM image (a); EDX spectrum of this area (b); element composition (c) and maps of titanium (d), carbon (e), and oxygen (f) distributions.

SEM images of initial sample of W-C powder

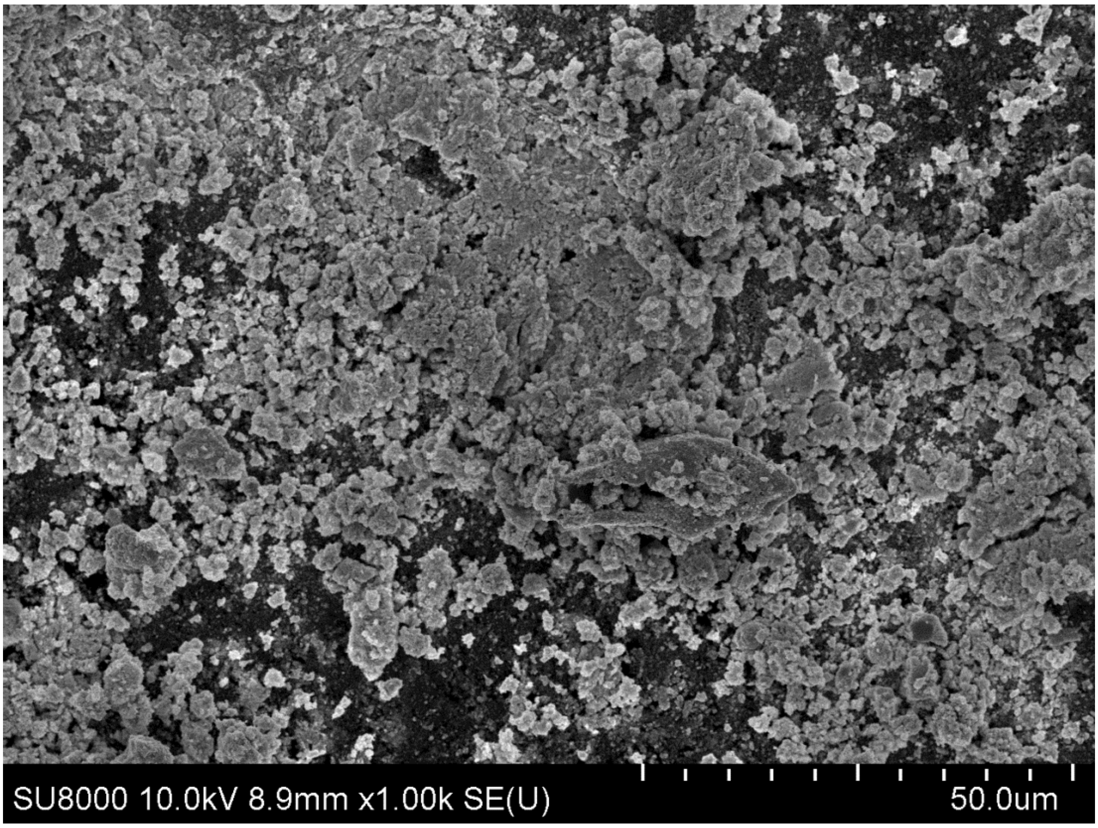

Figure S75. SEM image of initial sample of W-C powder.

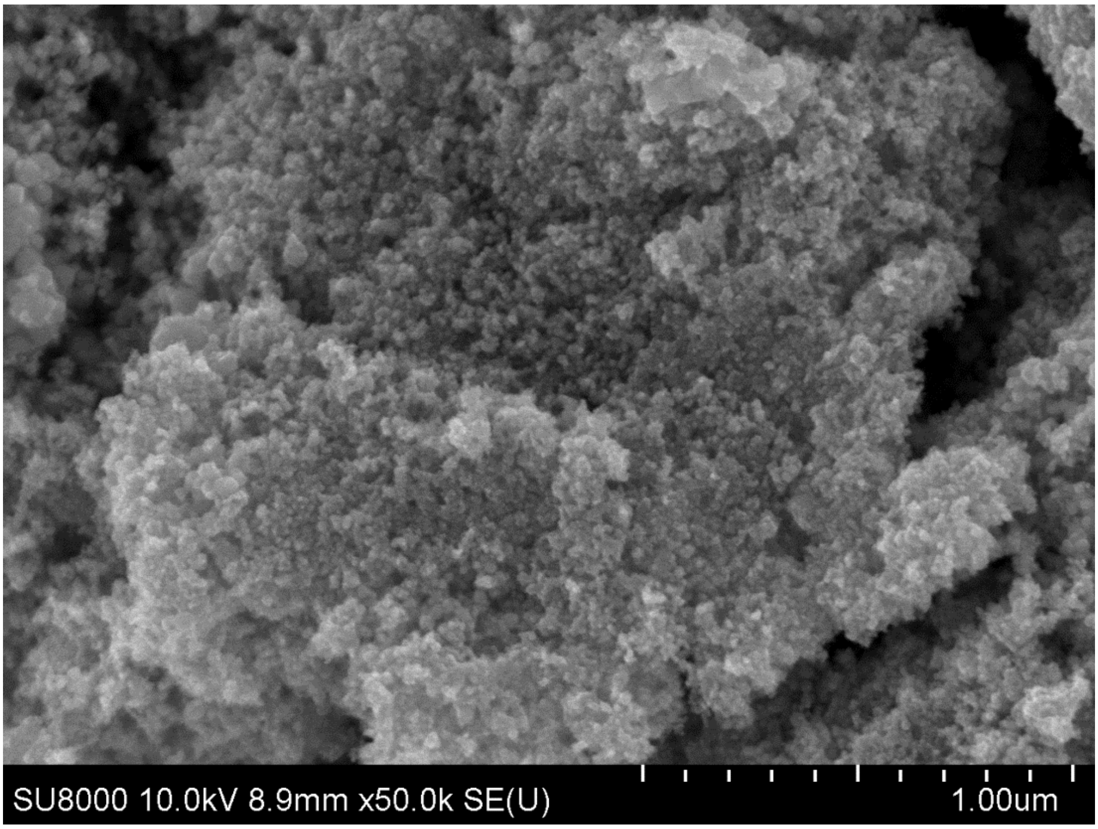

Figure S76. SEM image of initial sample of W-C powder.

EDX data of initial sample of W-C powder

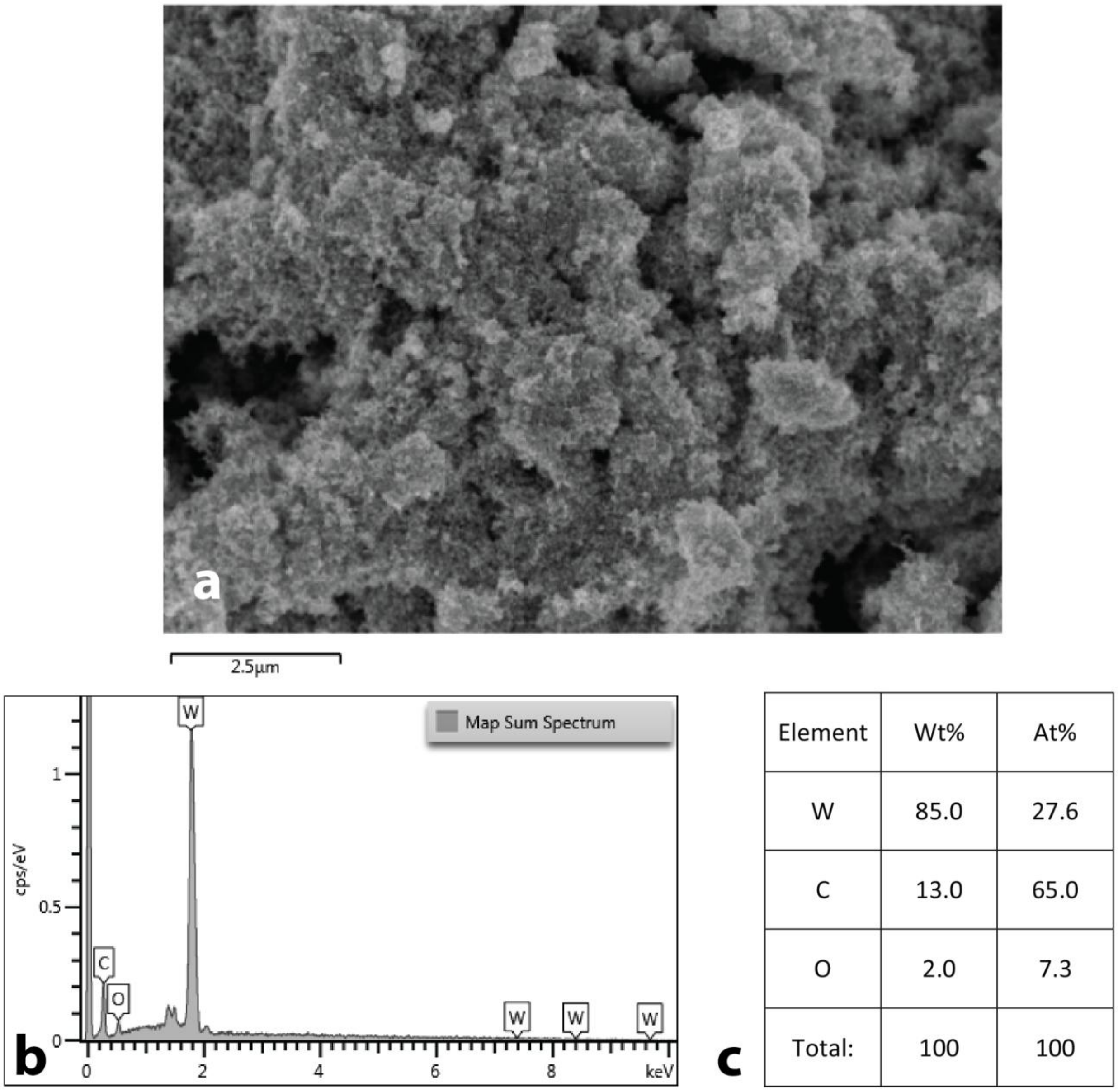

c

Element	Wt%	At%
W	85.0	27.6
C	13.0	65.0
O	2.0	7.3
Total:	100	100

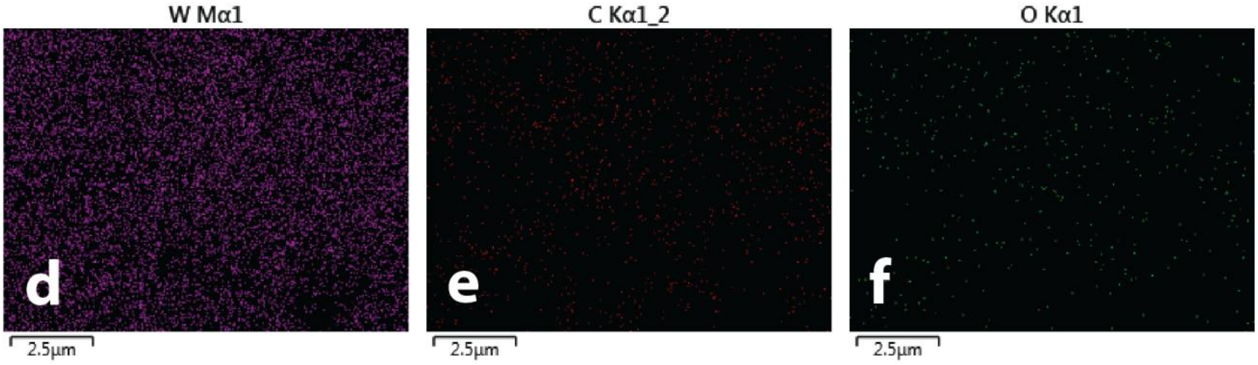

Figure S77. EDX study of initial W-C sample: SEM image (a); EDX spectrum of this area (b); element composition (c) and maps of tungsten (d), carbon (e), and oxygen (f) distributions. The increased oxygen content in the EDX analysis result by its presence in aluminum stub which fixed samples for SEM. In addition, an oxide film on the surface of the sample can be a source of the oxygen signal.

SEM images of W-C powder sample after MW treatment

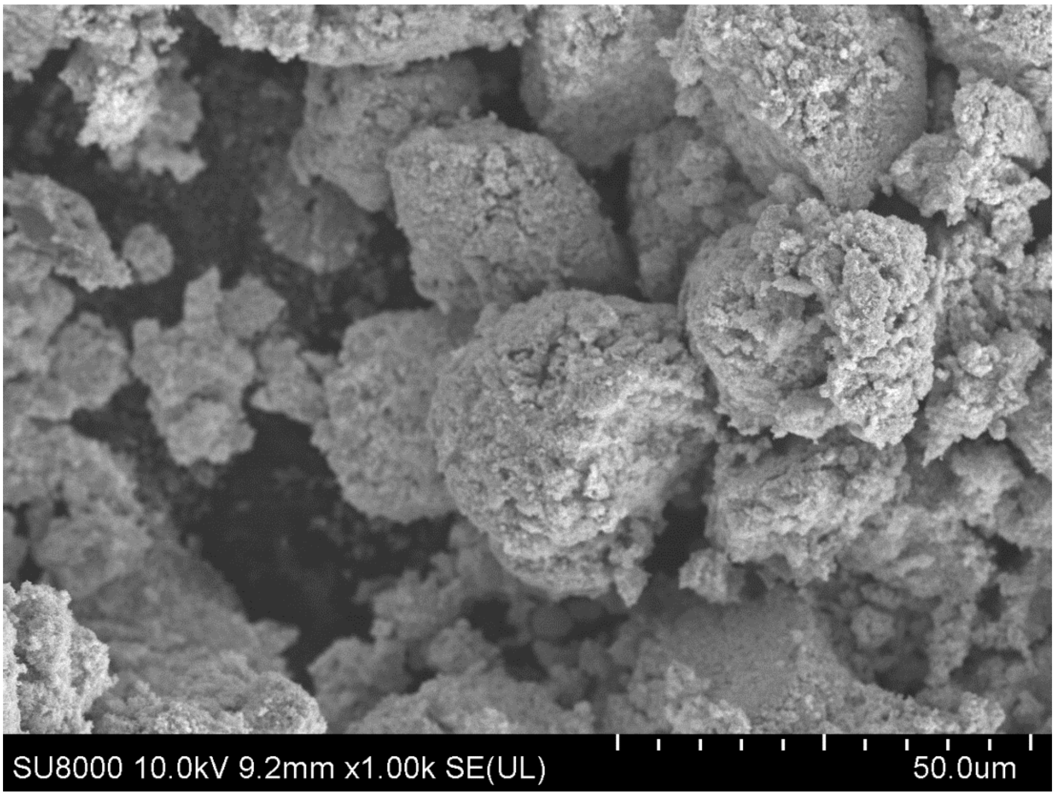

Figure S78. SEM image of W-C powder sample after MW treatment.

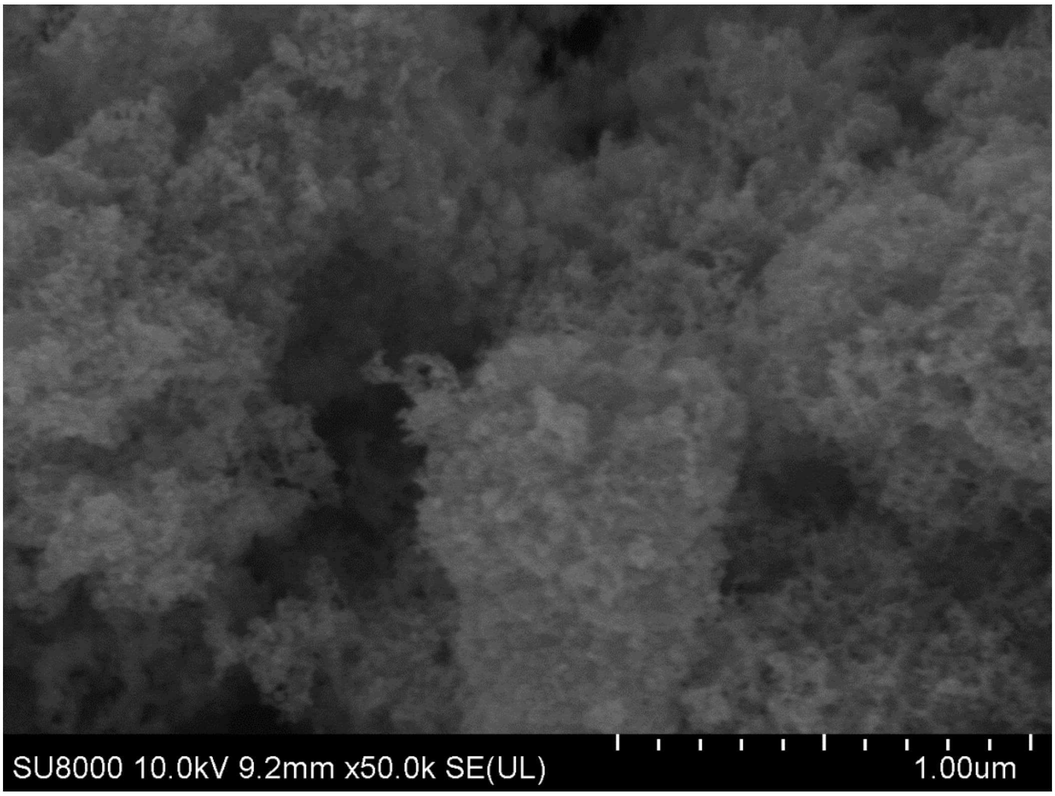

Figure S79. SEM image of W-C powder sample after MW treatment.

EDX data of W-C powder sample after MW treatment

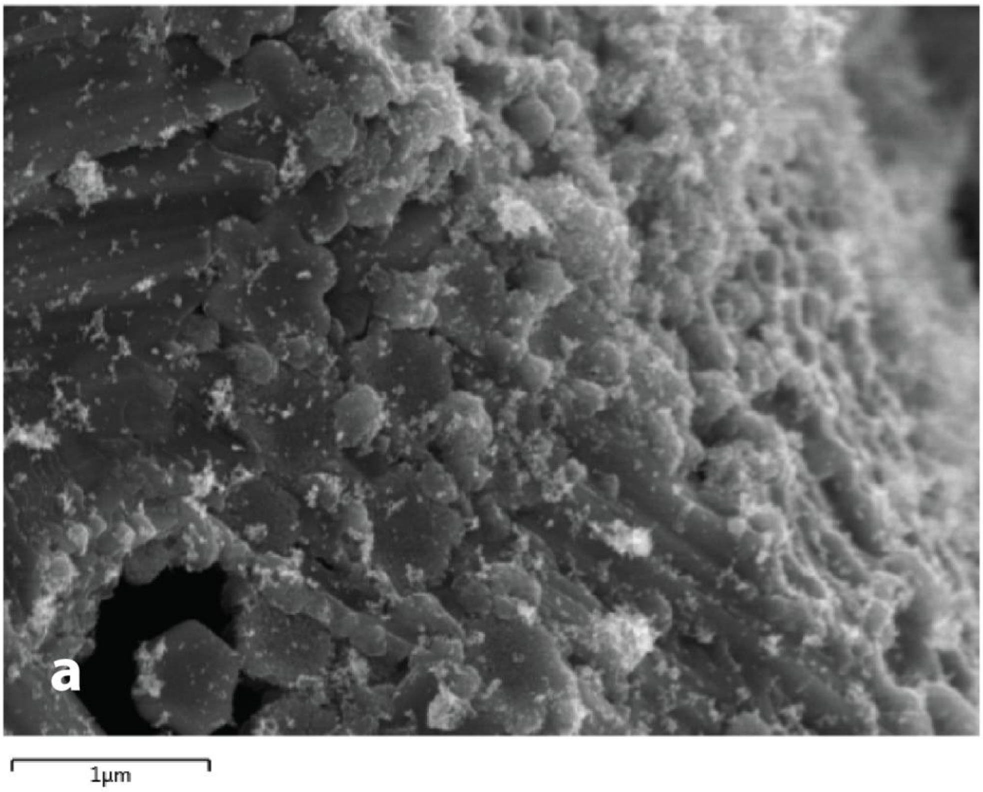

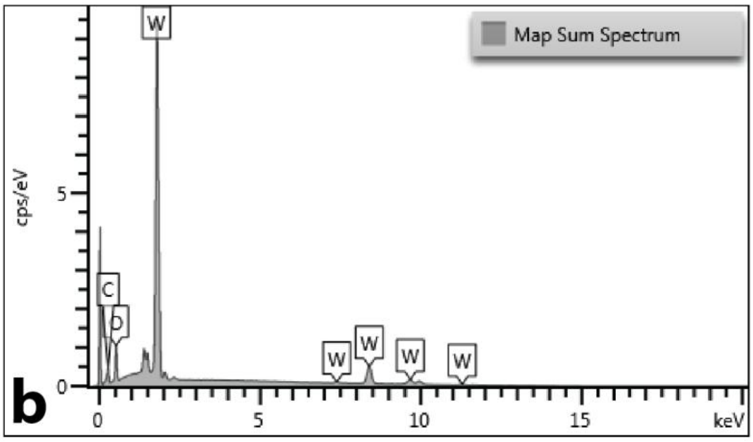

c

Element	Wt%	At%
W	84.8	29.8
C	6.6	35.4
O	8.6	34.8
Total:	100	100

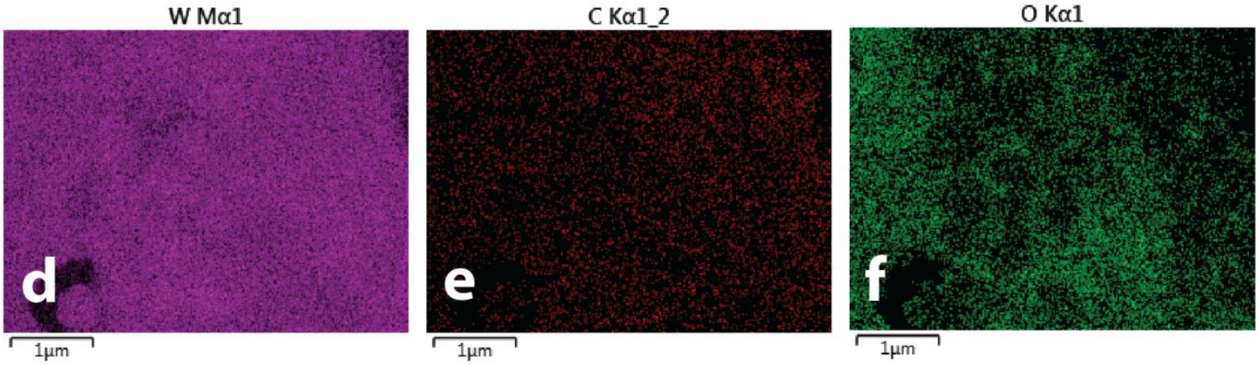

Figure S80. EDX study of W-C powder after MW treatment: SEM image (a); EDX spectrum of this area (b); element composition (c) and maps of tungsten (d), carbon (e), and oxygen (f) distributions.

SEM images of initial sample of V-C powder

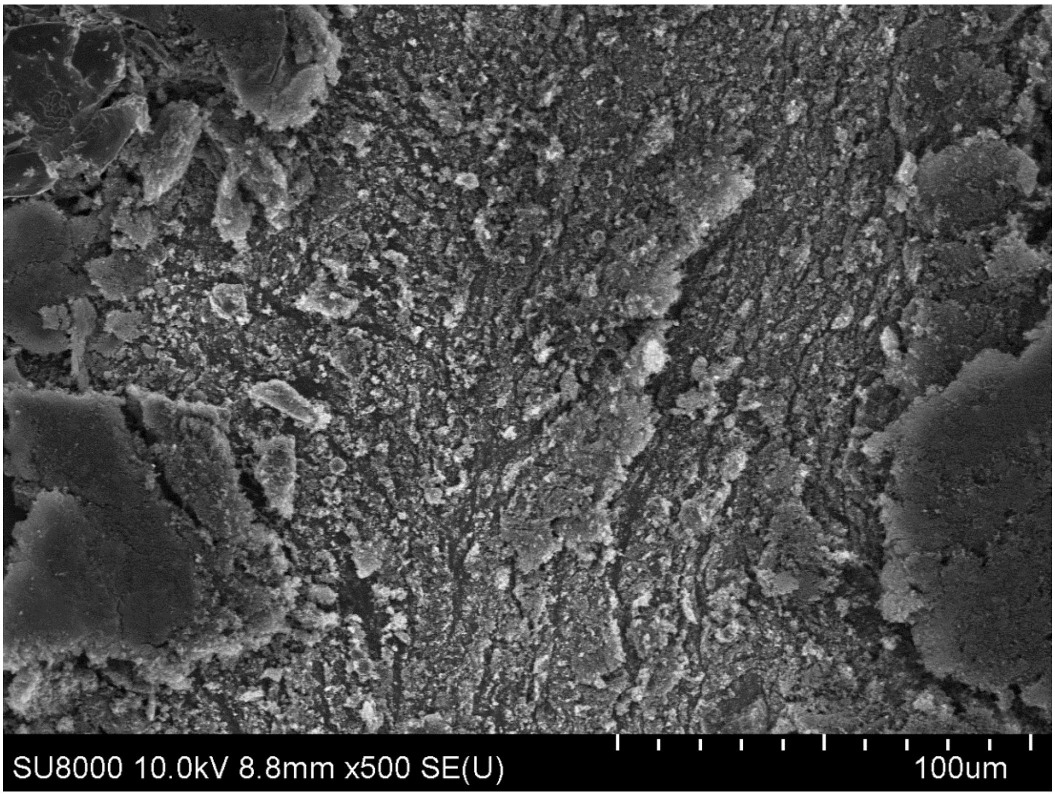

Figure S81. SEM image of initial sample of V-C powder.

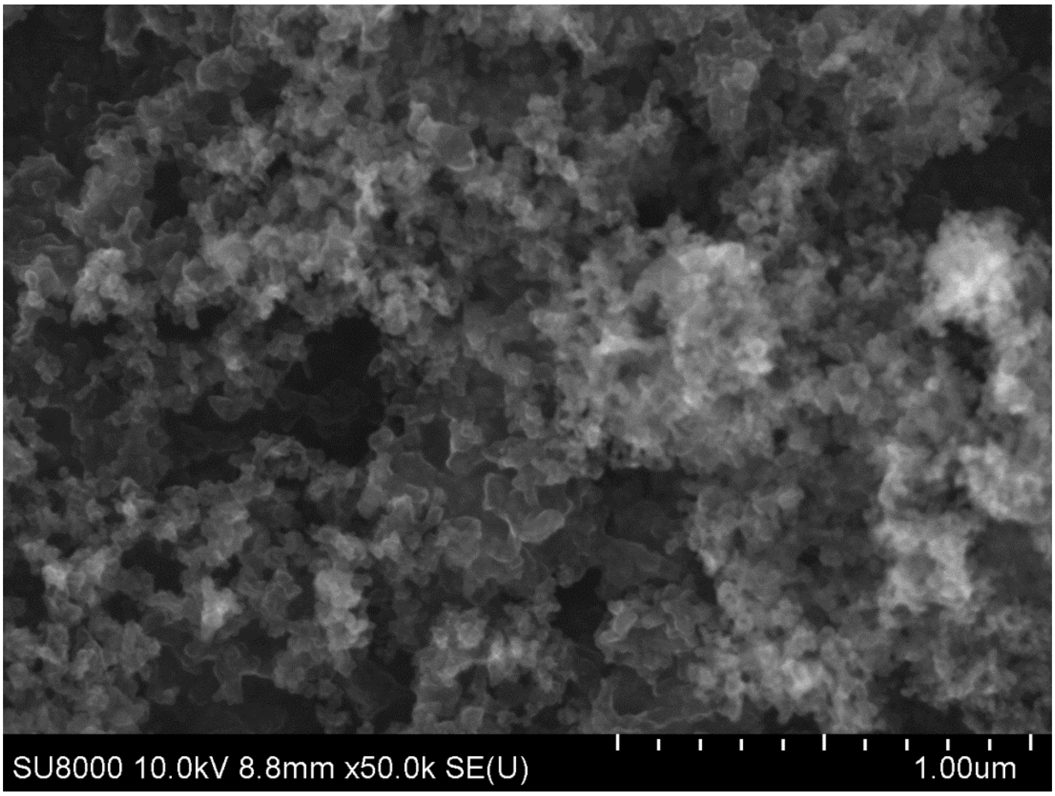

Figure S82. SEM image of initial sample of V-C powder.

SEM images of V-C powder sample after MW treatment

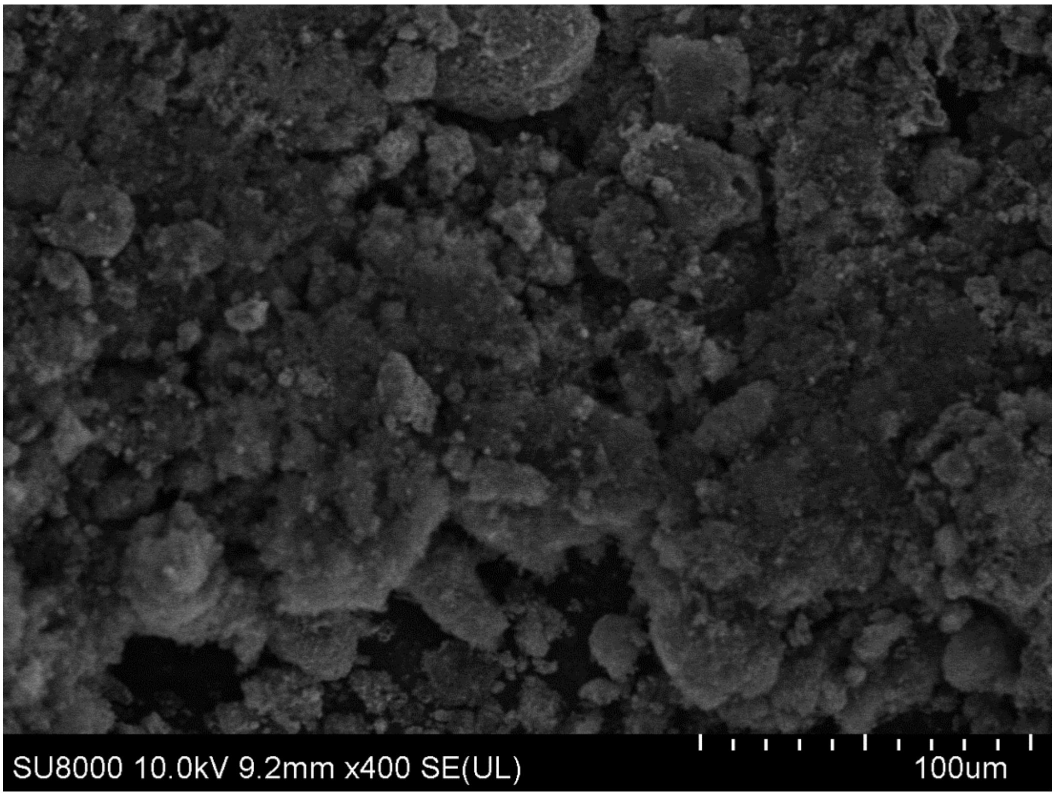

Figure S83. SEM image of V-C powder sample after MW treatment.

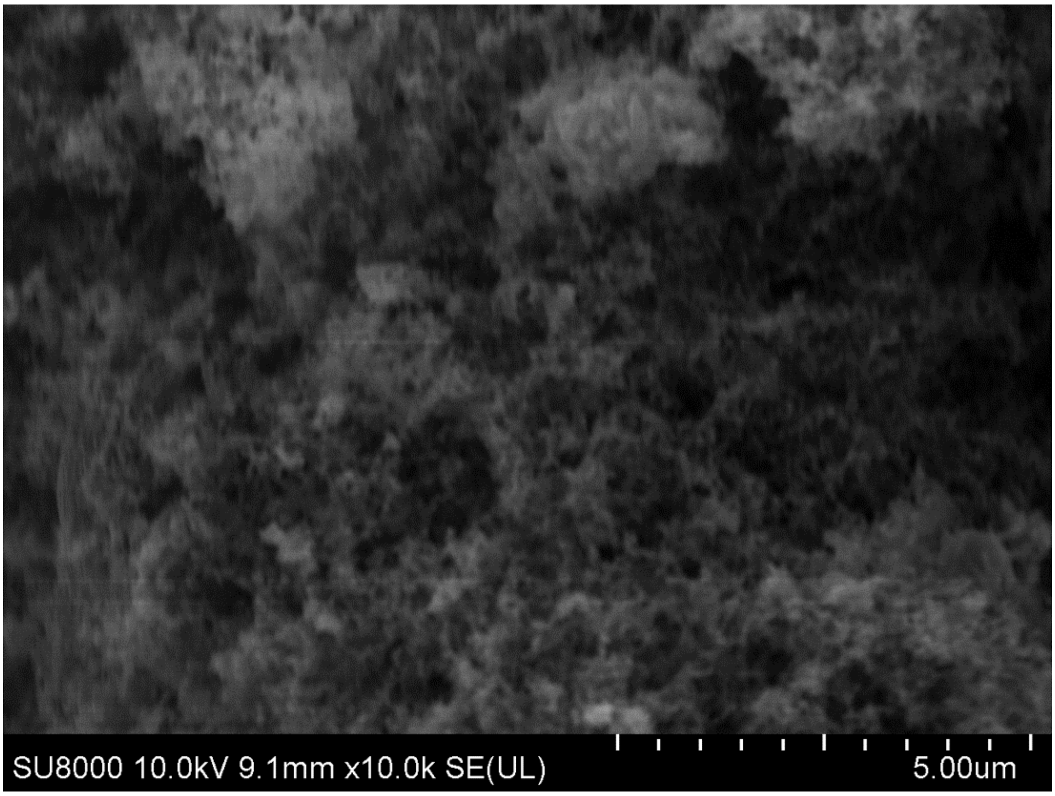

Figure S84. SEM image of V-C powder sample after MW treatment.

SEM images of initial sample of Cr-C powder

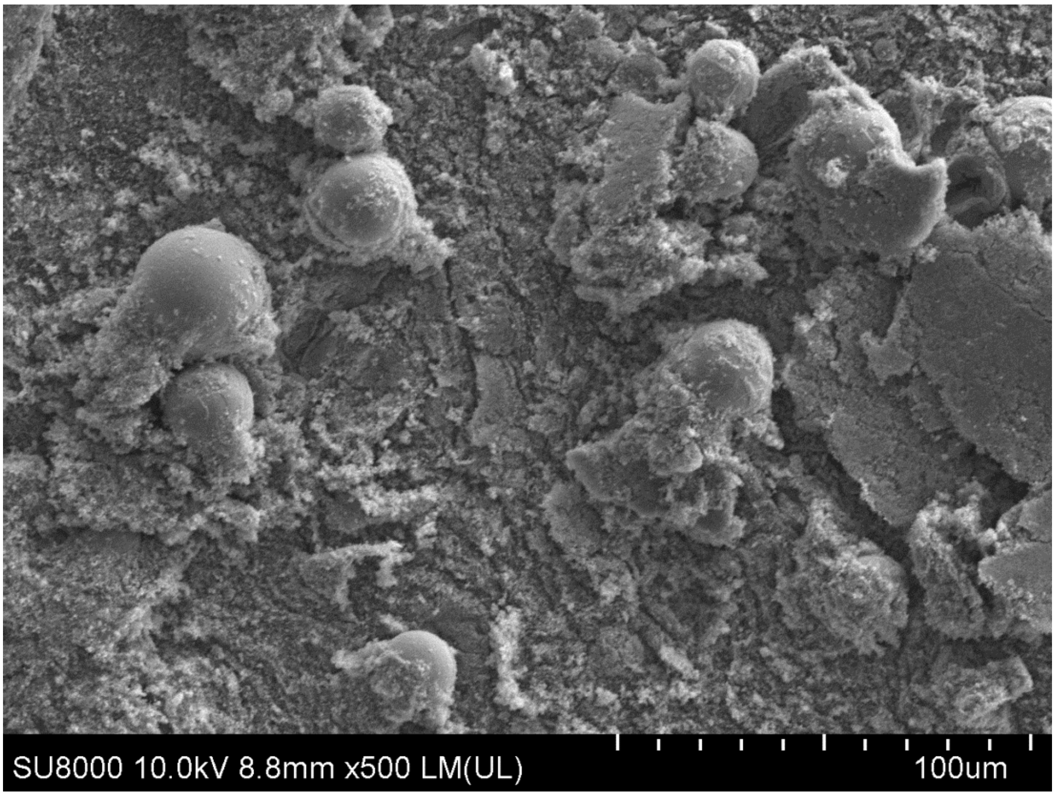

Figure S85. SEM image of initial sample of Cr-C powder.

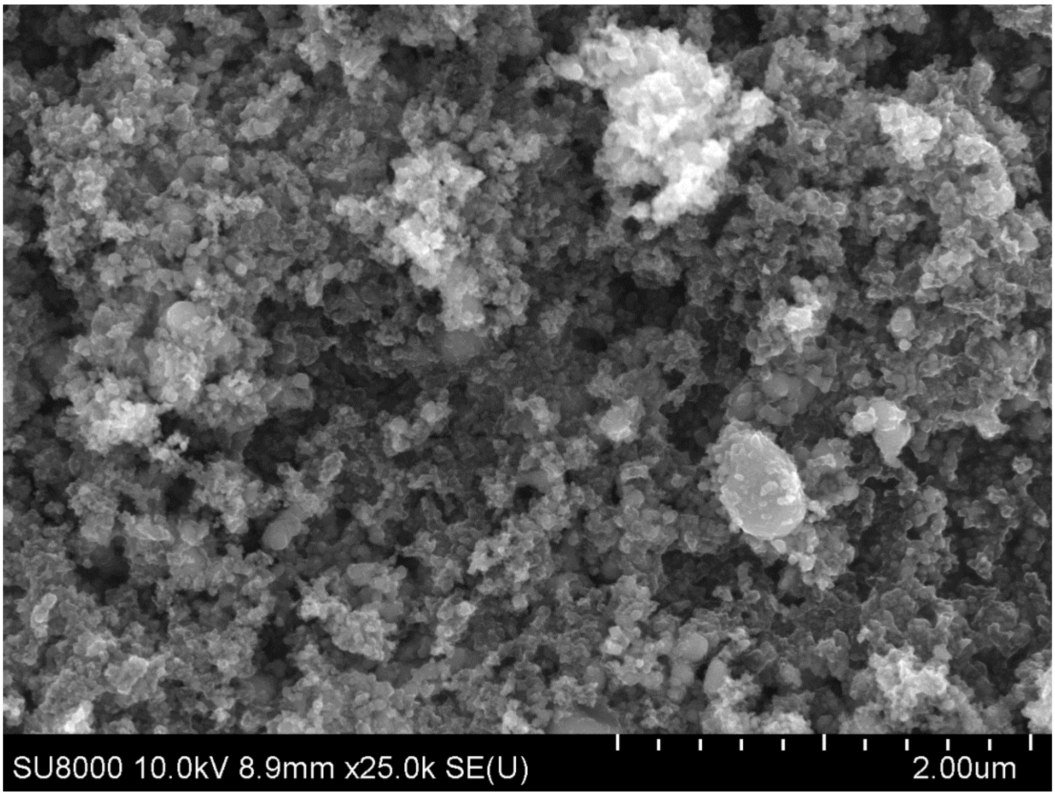

Figure S86. SEM image of initial sample of Cr-C powder.

EDX data of initial sample of Cr-C powder

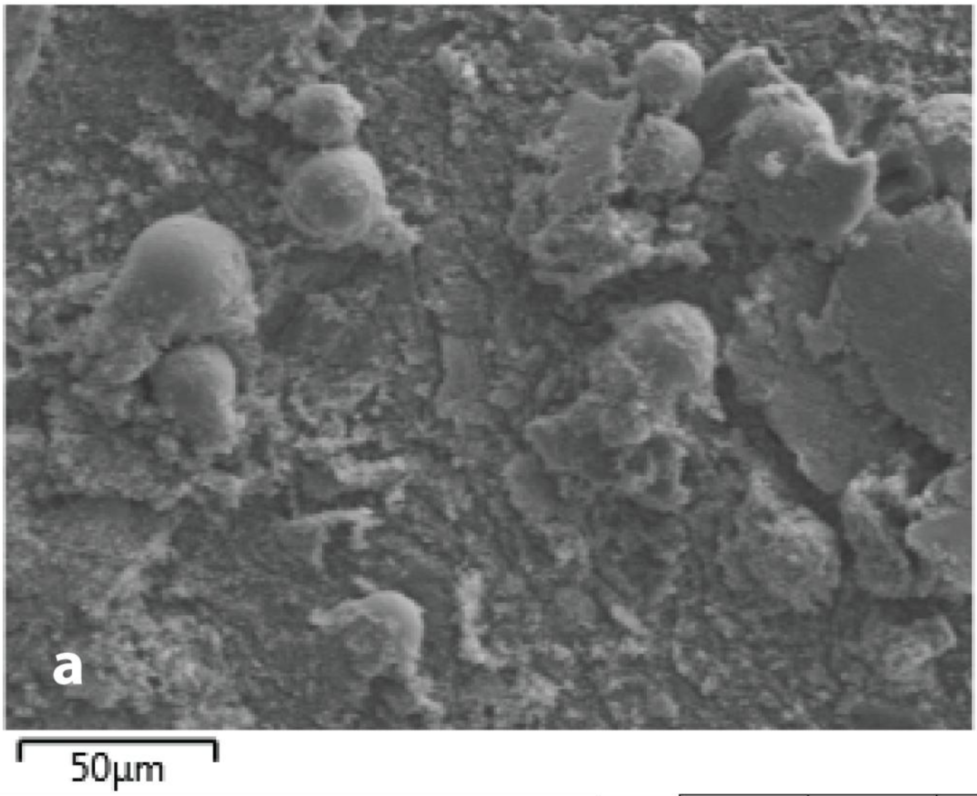

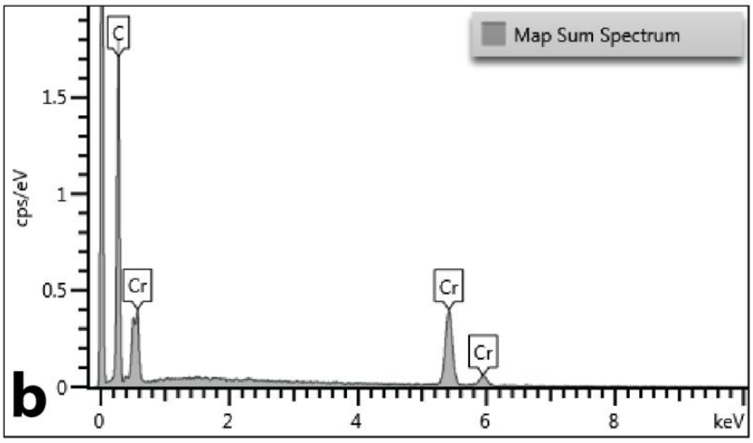

c

Element	Wt%	At%
C	35.81	70.85
Cr	64.19	29.15
Total:	100	100

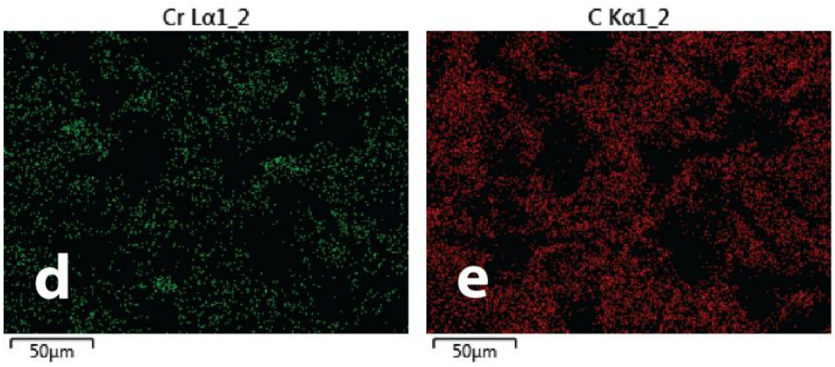

Figure S87. EDX study of initial sample of Cr-C powder: SEM image (a); EDX spectrum of this area (b); element composition (c) and maps of chromium (d) and carbon (e) distributions.

SEM images of Cr-C powder sample after MW treatment

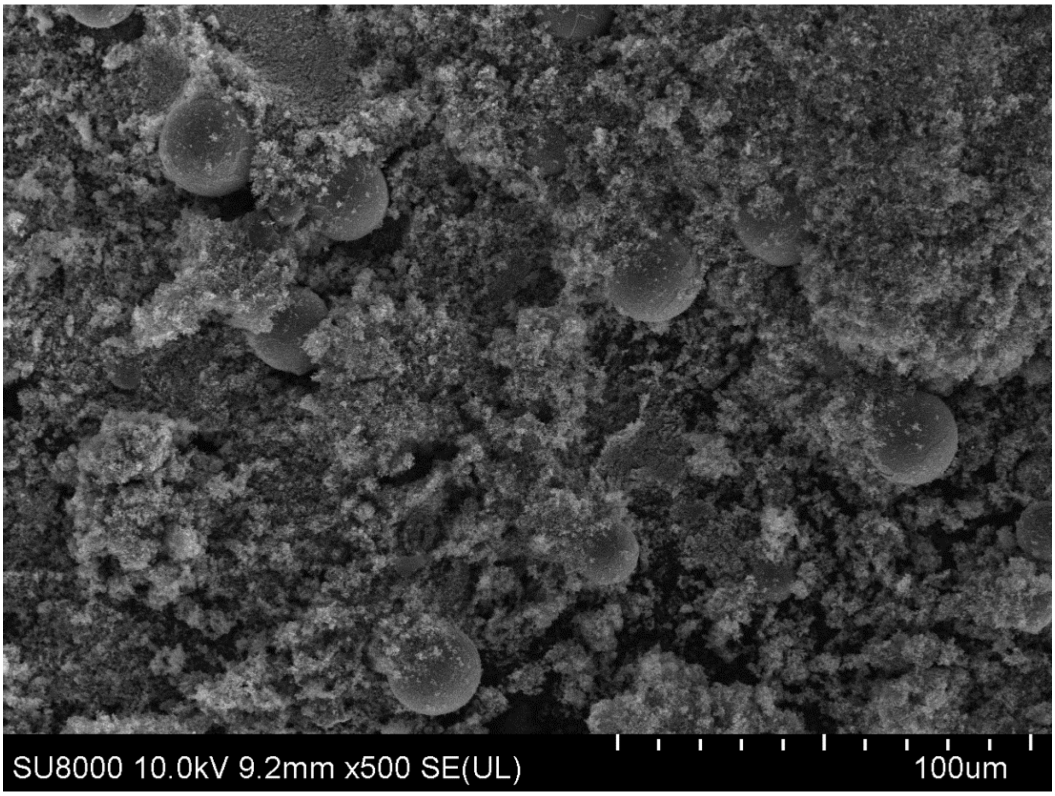

Figure S88. SEM image of Cr-C powder sample after MW treatment.

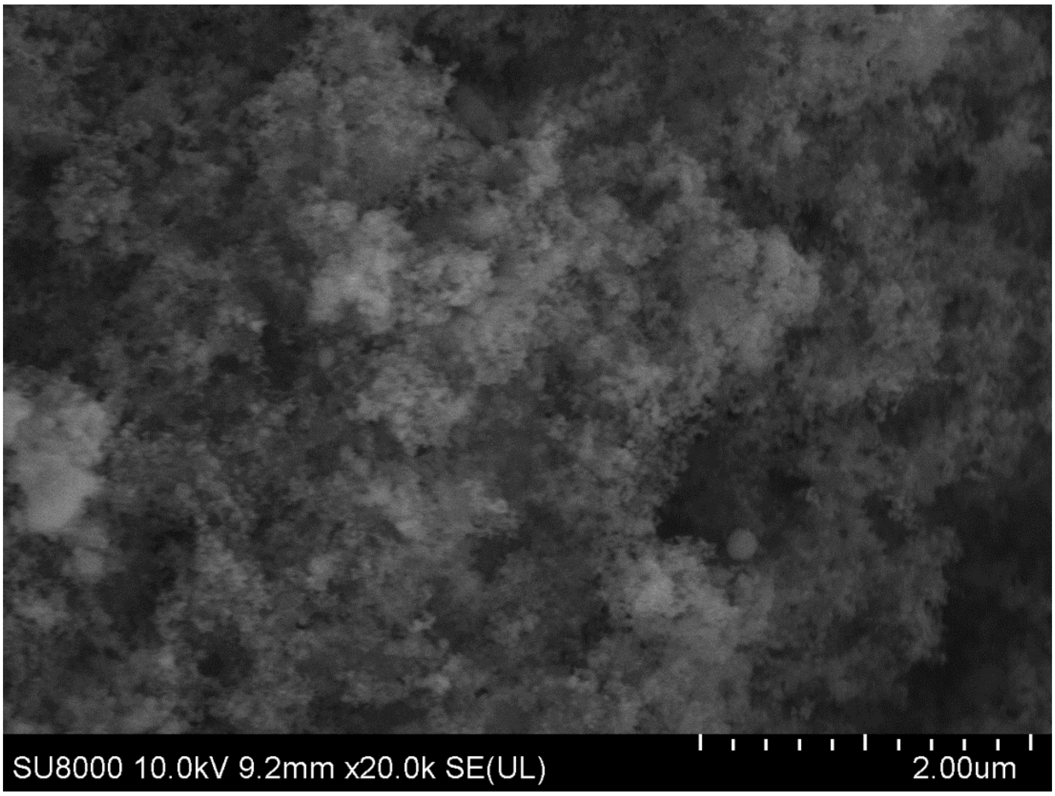

Figure S89. SEM image of Cr-C powder sample after MW treatment.

EDX data of Cr-C powder sample after MW treatment

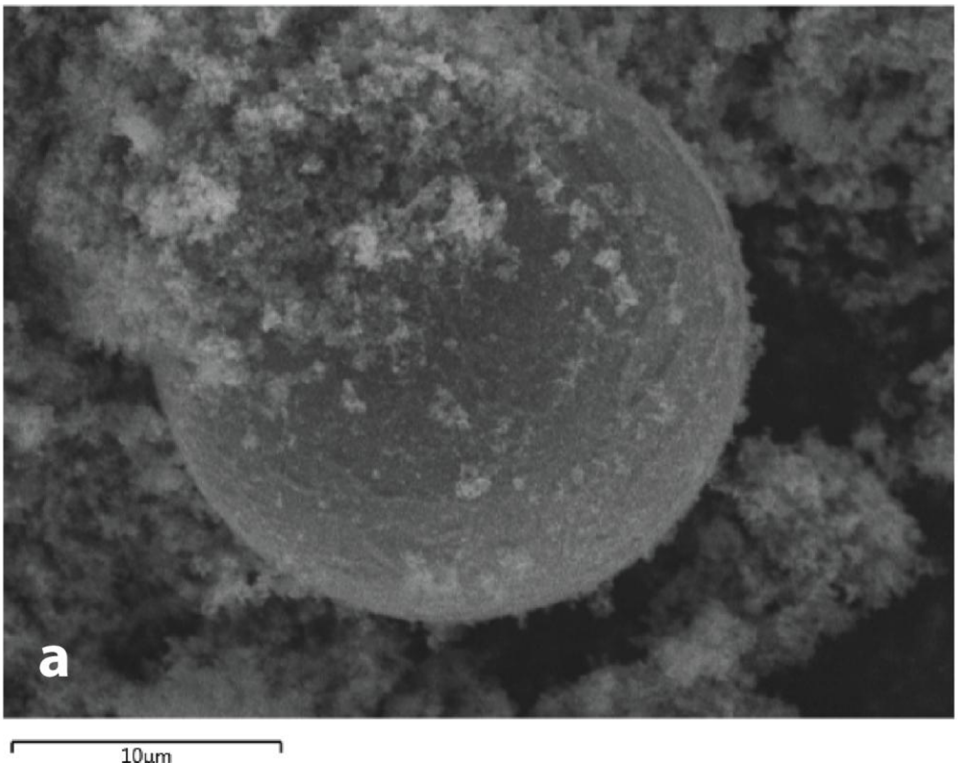

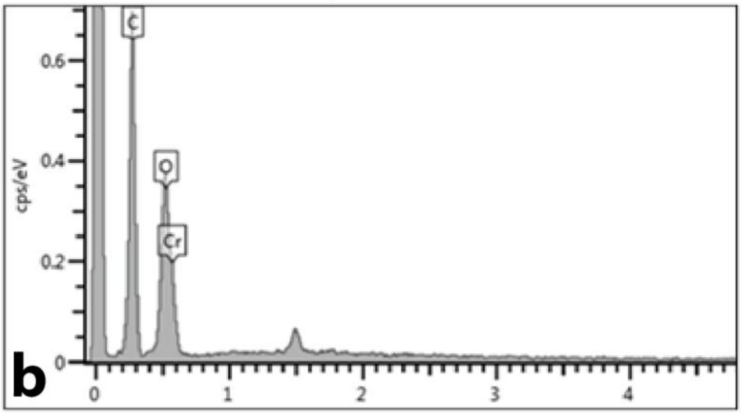

c

Element	Wt%	At%%
C	30.58	59.98
O	8.43	12.41
Cr	60.99	27.61
Total:	100	100

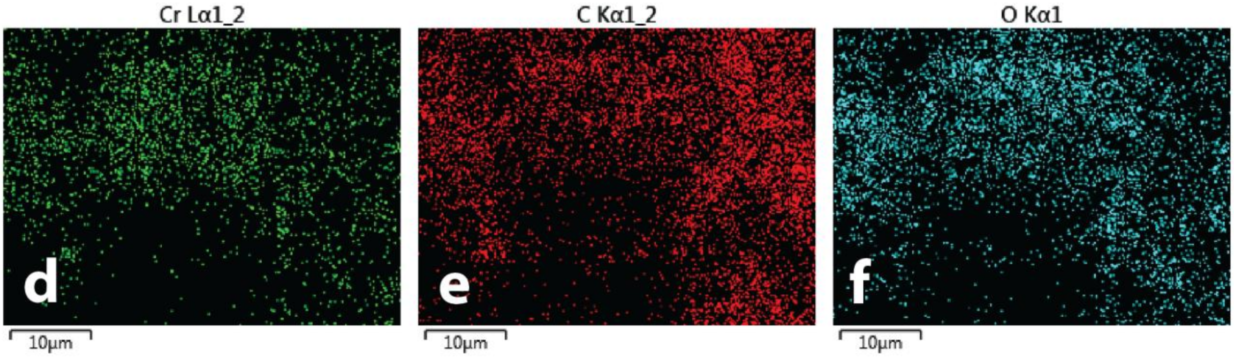

Figure S90. EDX study of Cr-C powder sample after MW treatment: SEM image (a); EDX spectrum of this area (b); element composition (c) and maps of chromium (d), carbon (e) and oxygen (f) distributions.

SEM images of initial sample of W-V-C powder

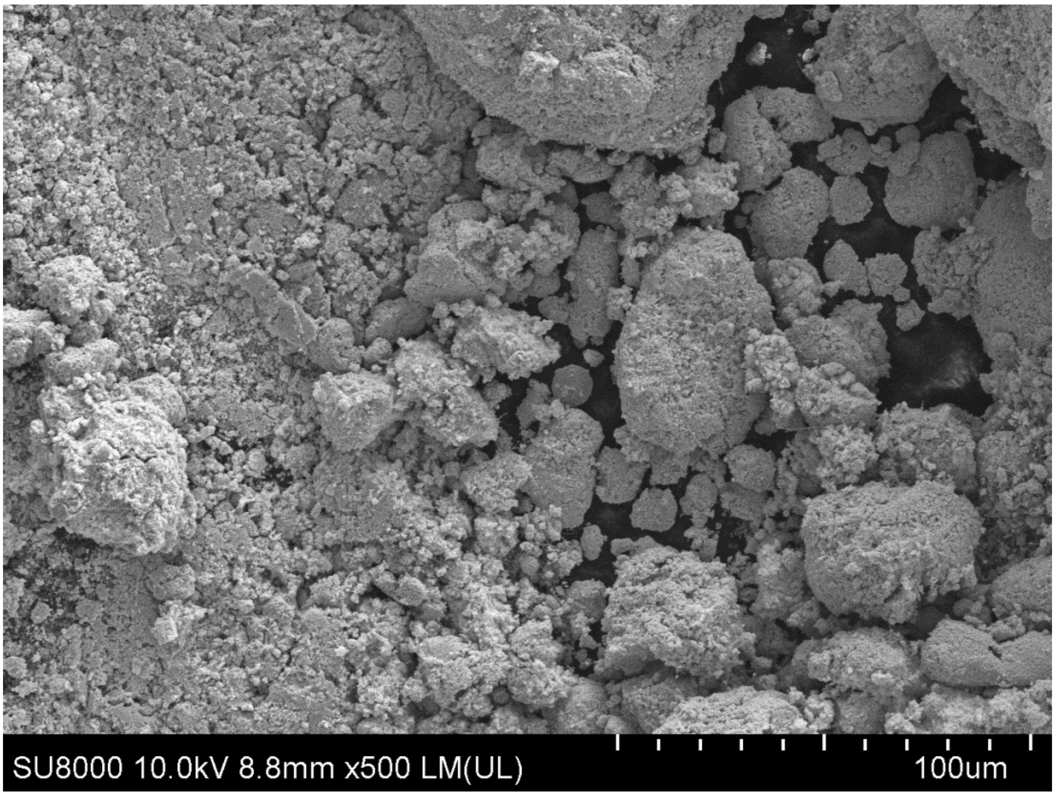

Figure S91. SEM image of initial sample of W-V-C powder.

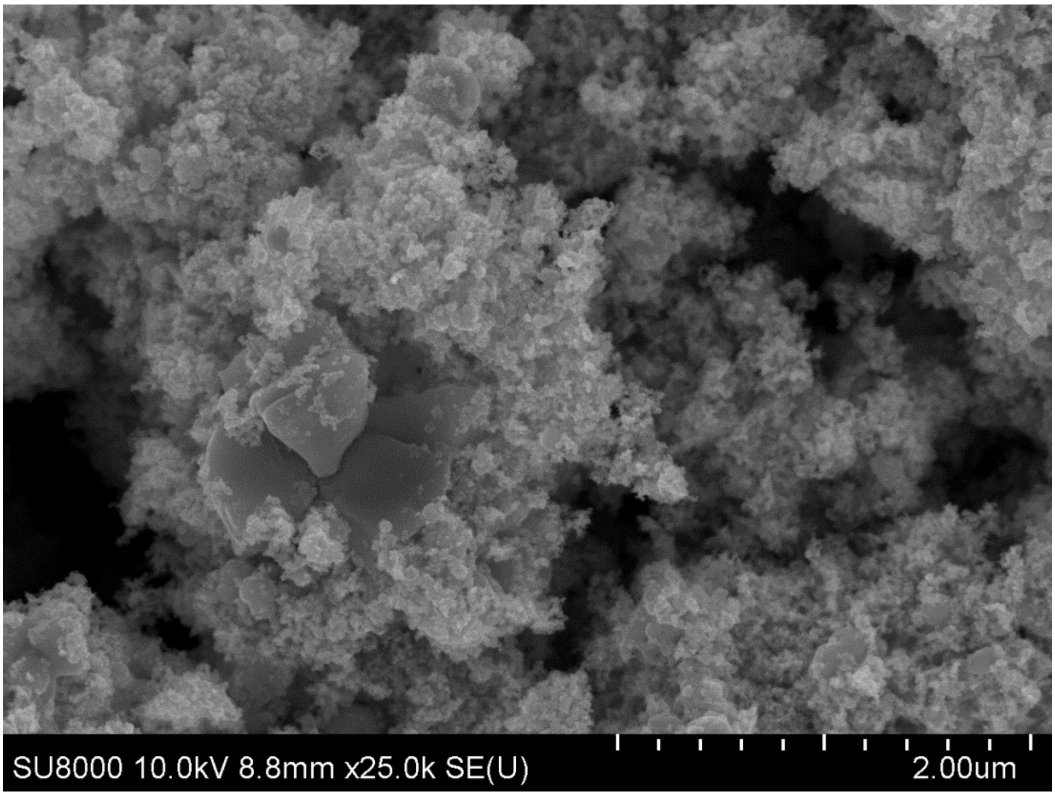

Figure S92. SEM image of initial sample of W-V-C powder.

EDX data of initial sample of W-V-C powder

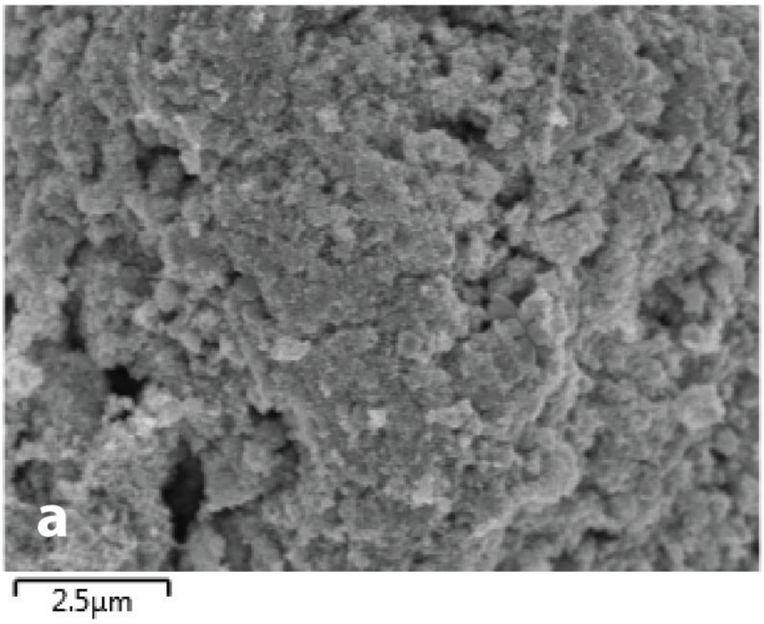

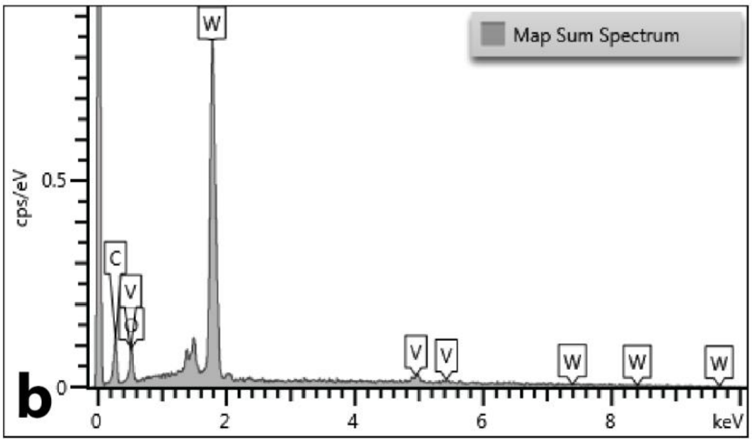

c

Element	Wt%	At%
W	82.0	28.1
V	4.2	5.2
C	9.5	49.5
O	4.4	17.2
Total:	100	100

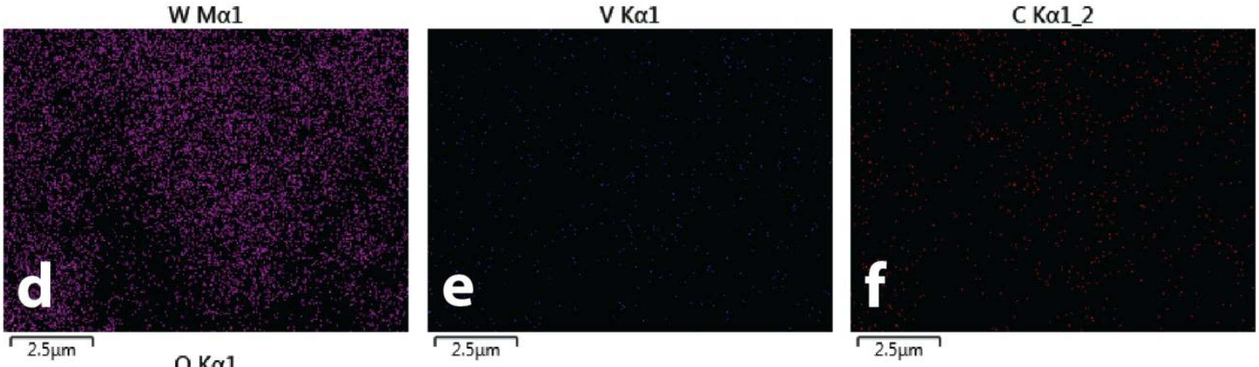

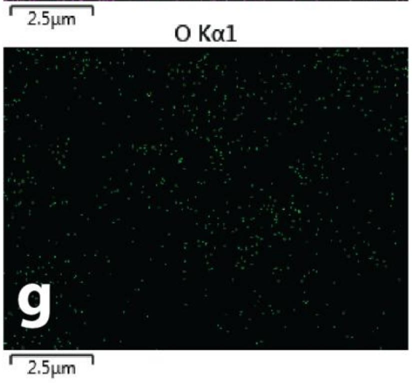

Figure S93. EDX study of initial sample of W-V-C powder: SEM image (a); EDX spectrum of this area (b); element composition (c) and maps of tungsten (d), vanadium (e), carbon (f) and oxygen (g) distributions. The increased oxygen content in the EDX analysis result by its presence in aluminum stub which fixed samples for SEM. In addition, an oxide film on the surface of the sample can be a source of the oxygen signal.

SEM images of W-V-C powder sample after MW treatment

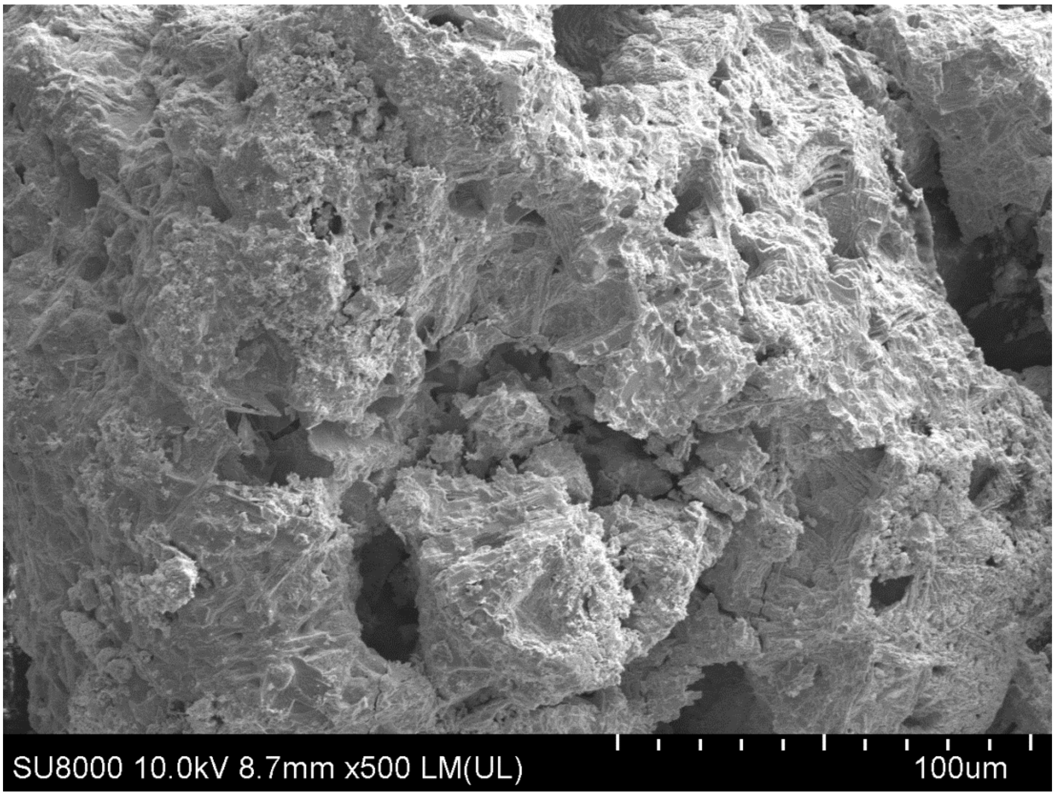

Figure S94. SEM image of W-V-C powder sample after MW treatment.

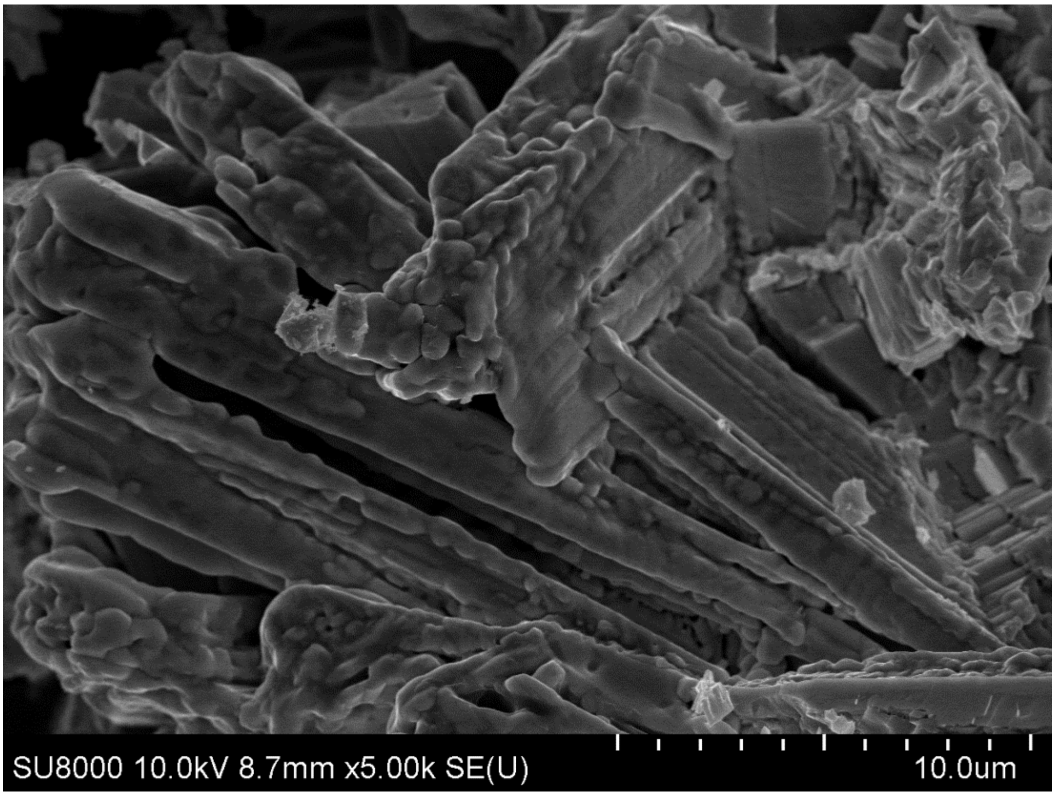

Figure S95. SEM image of W-V-C powder sample after MW treatment.

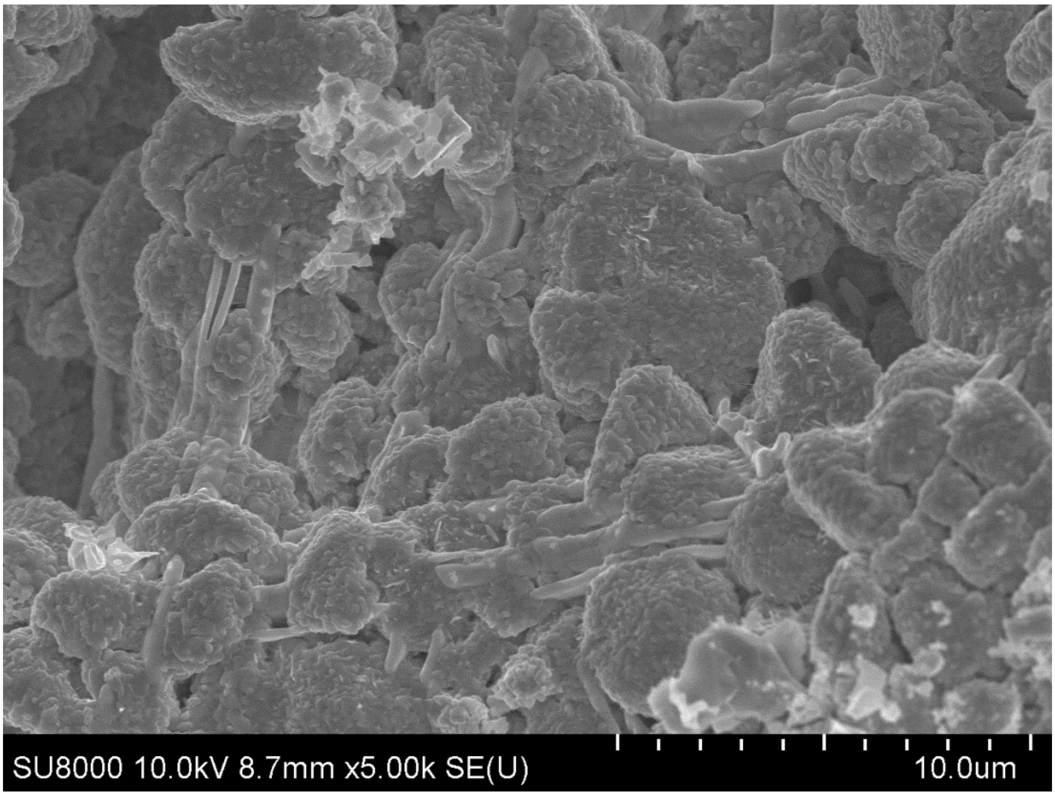

Figure S96. SEM image of W-V-C powder sample after MW treatment.

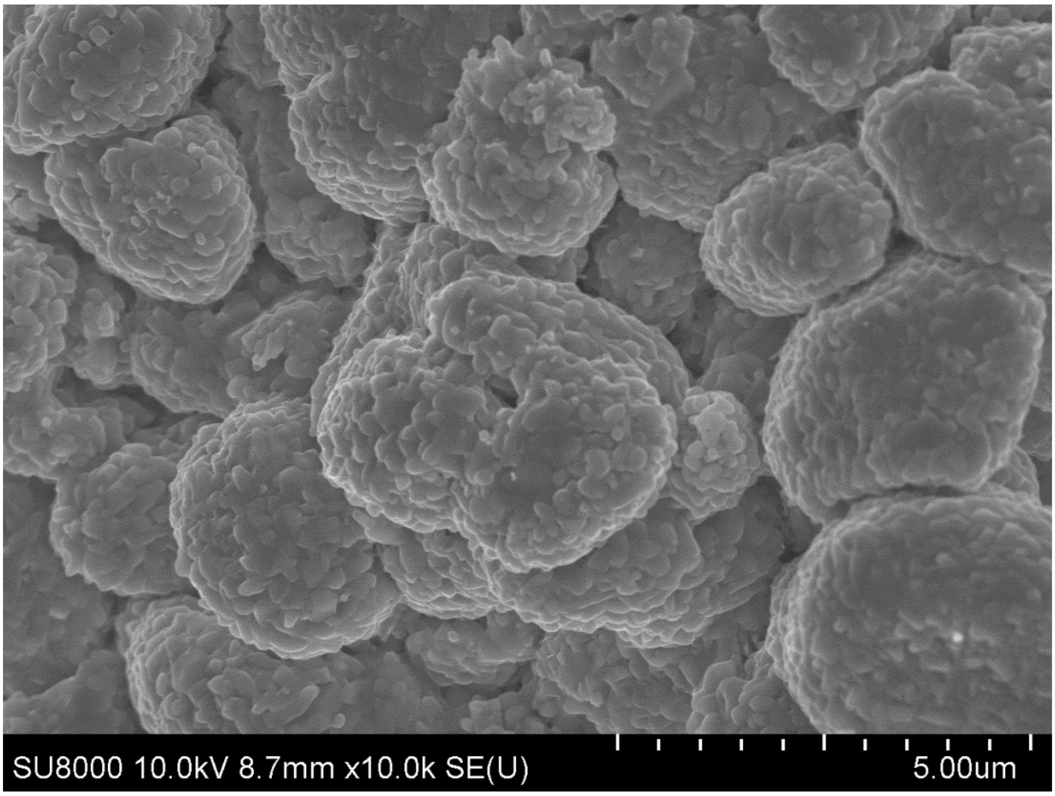

Figure S97. SEM image of W-V-C powder sample after MW treatment.

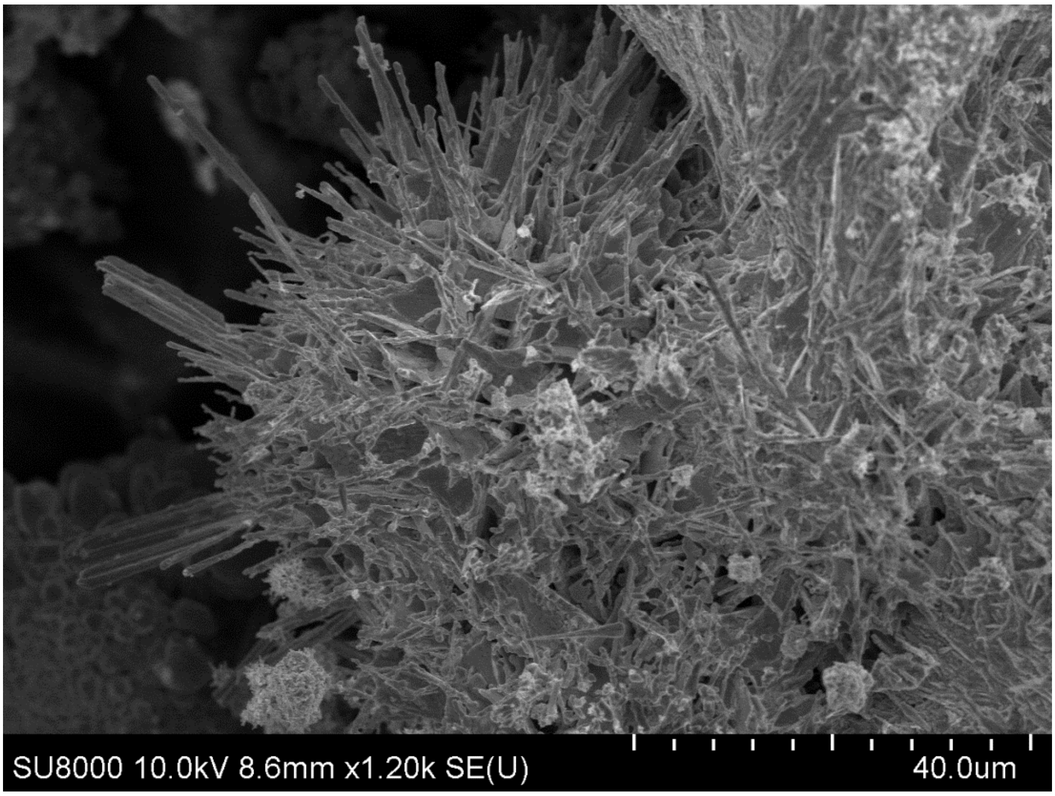

Figure S98. SEM image of W-V-C powder sample after MW treatment.

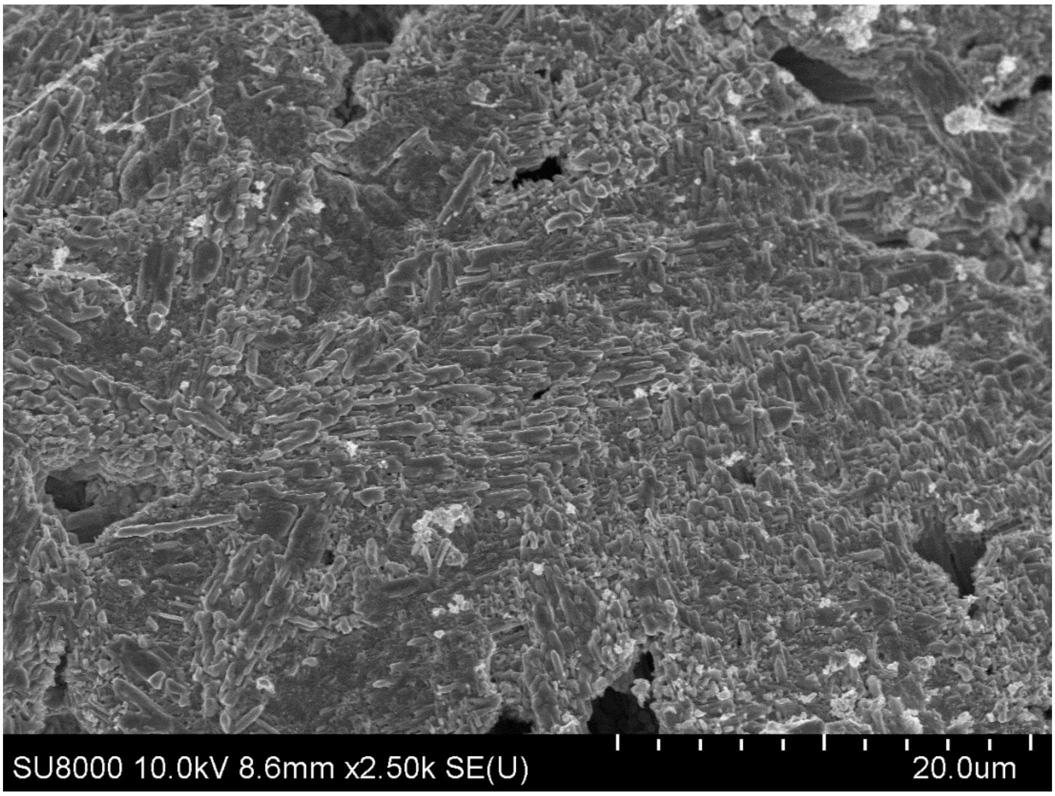

Figure S99. SEM image of W-V-C powder sample after MW treatment.

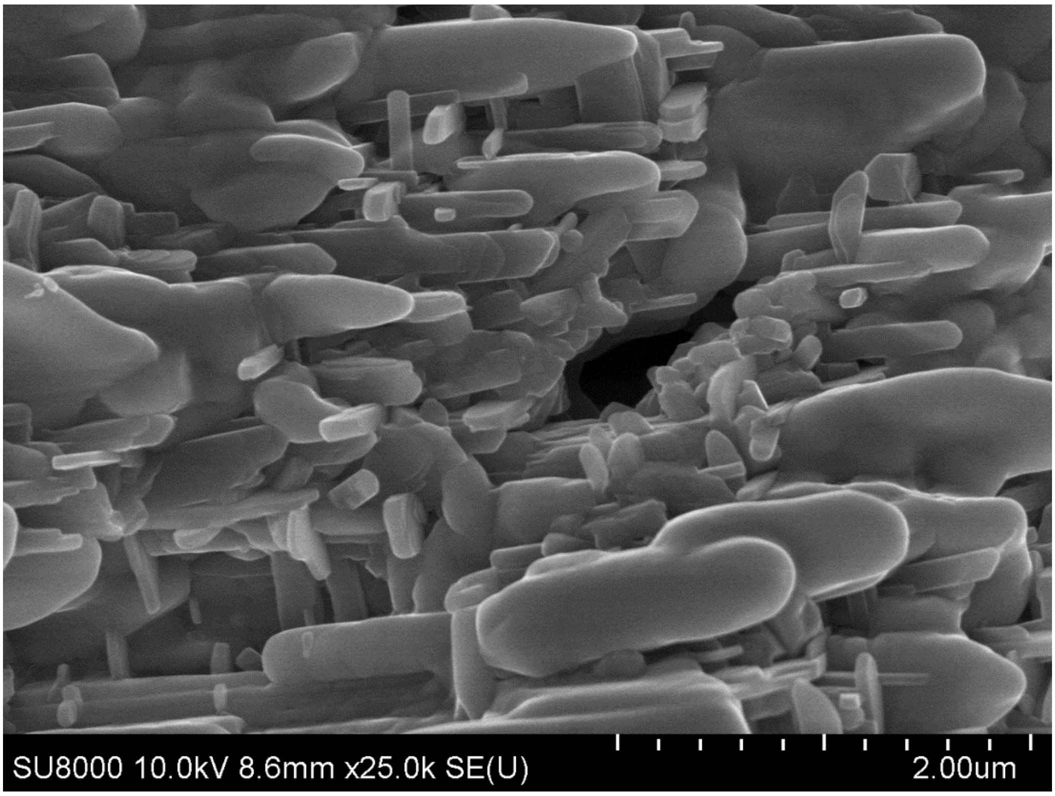

Figure S100. SEM image of W-V-C powder sample after MW treatment.

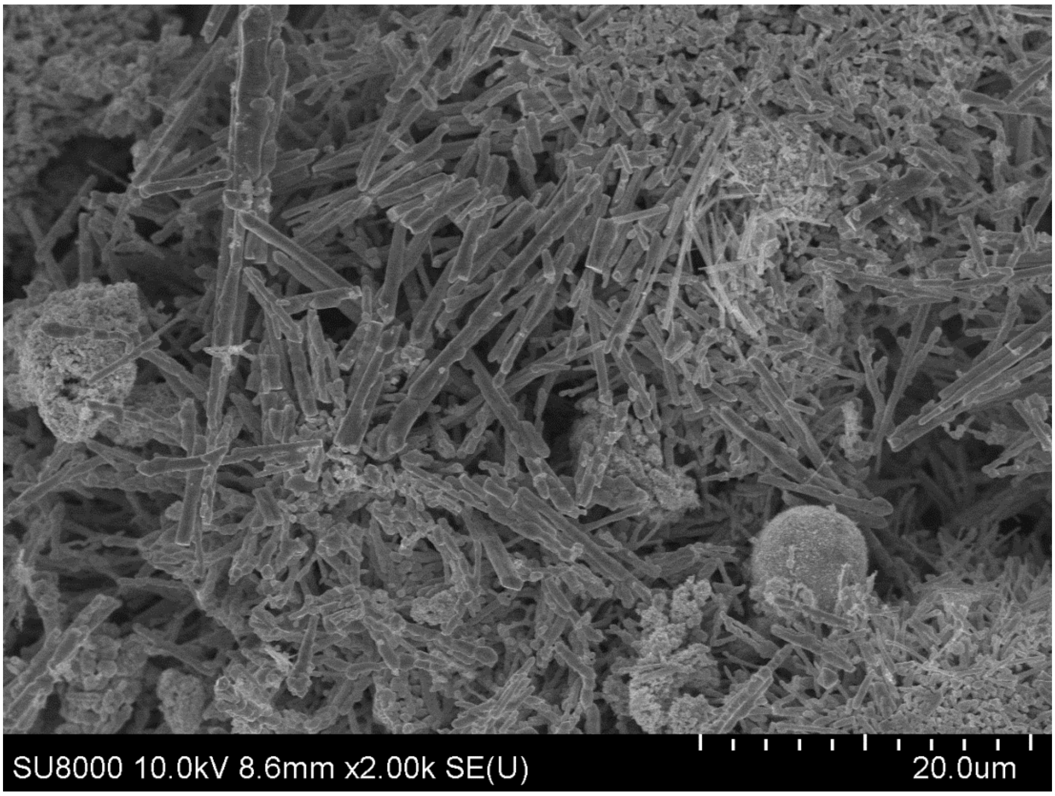

Figure S101. SEM image of W-V-C powder sample after MW treatment.

EDX data of W-V-C powder sample after MW treatment

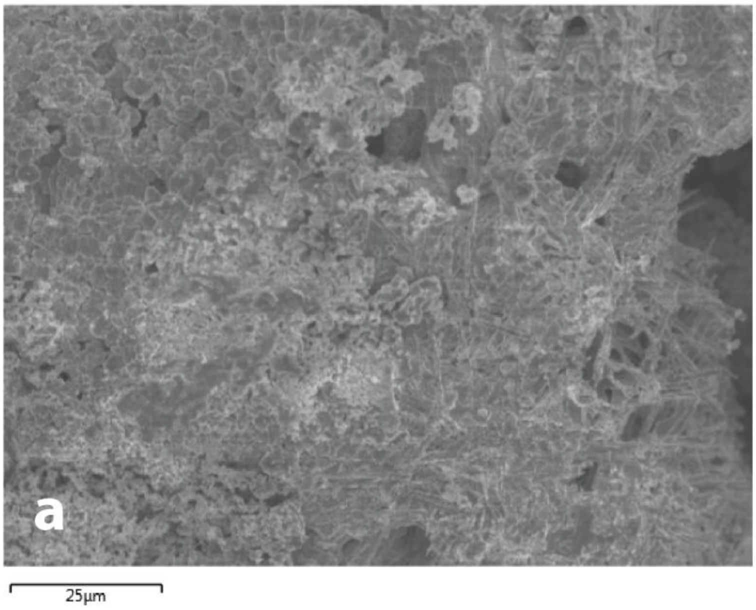

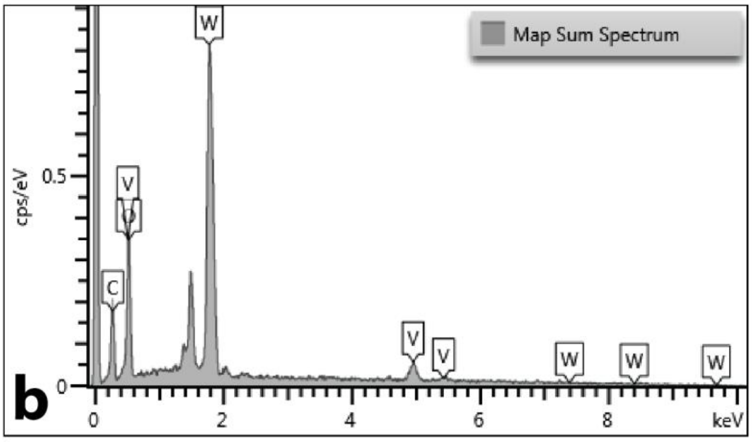

c

Element	Wt%	At%
W	64.3	14.5
V	9.4	7.5
C	11.3	39.1
O	15.0	38.9
Total:	100	100

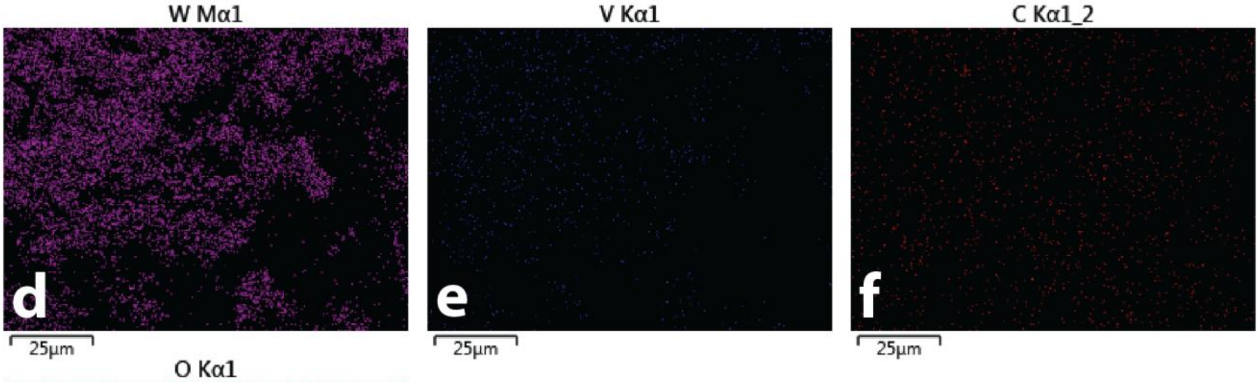

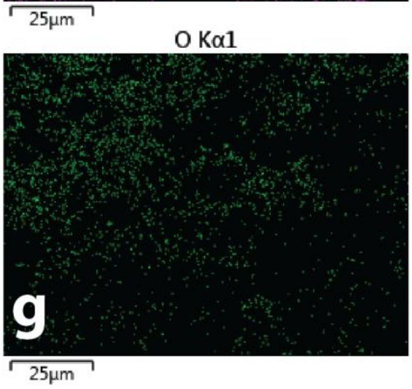

Figure S102. EDX study of W-V-C powder sample after MW treatment: SEM image (a); EDX spectrum of this area (b); element composition (c) and maps of tungsten (d), vanadium (e), carbon (f) and oxygen (g) distributions.

Images of changes in the morphology of graphite in the presence of metal compounds under microwave treatment conditions

SEM images of morphology of pure graphite after MW treatment

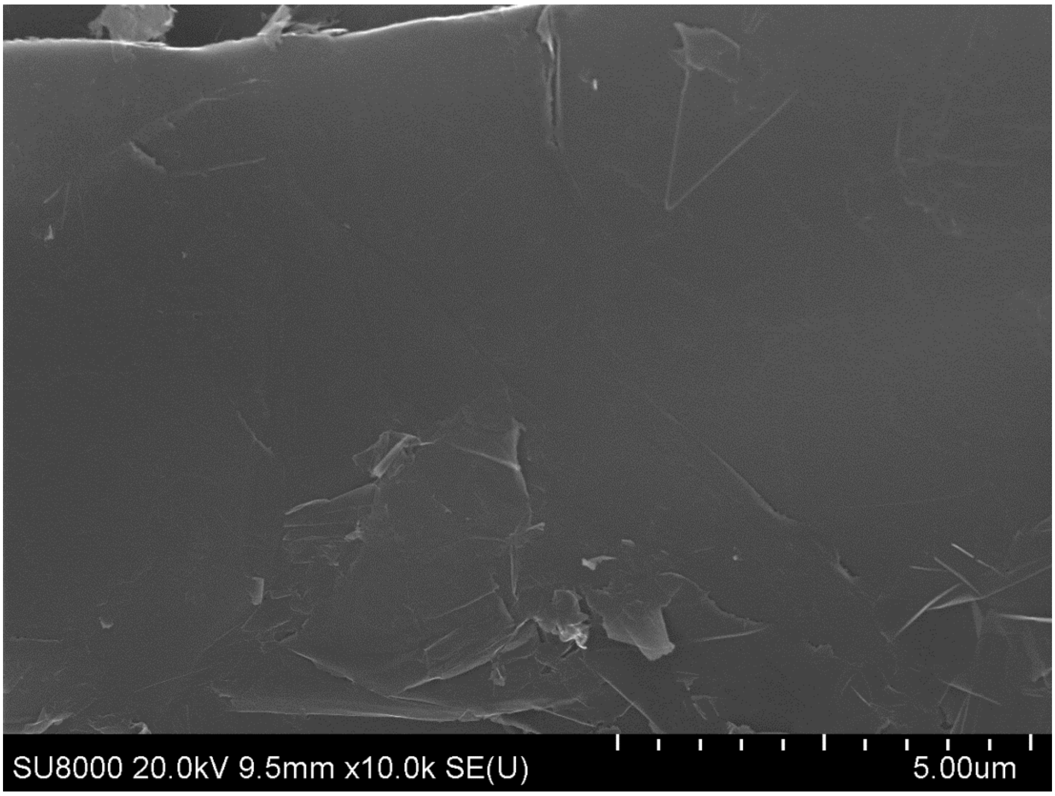

Figure S103. SEM image of pure graphite powder after MW treatment.

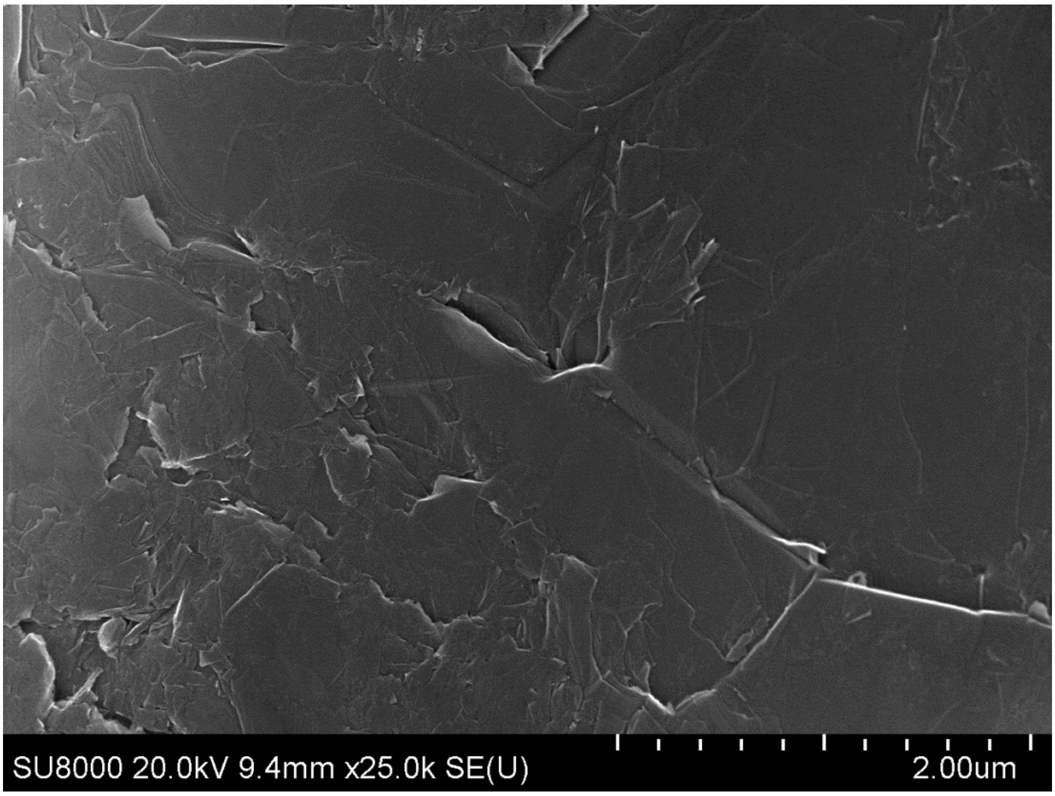

Figure S104. SEM image of pure graphite powder after MW treatment.

SEM images of changes in graphite morphology in the presence of Pt after MW treatment

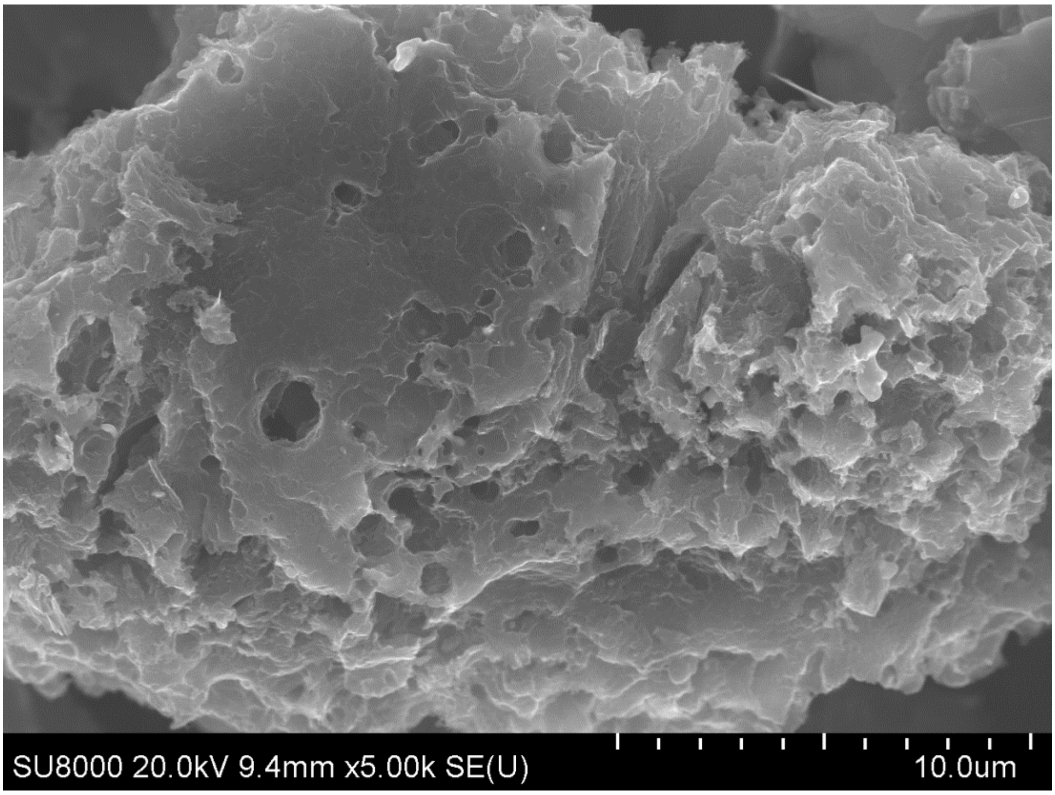

Figure S105. SEM image of graphite powder with Pt sample after MW treatment.

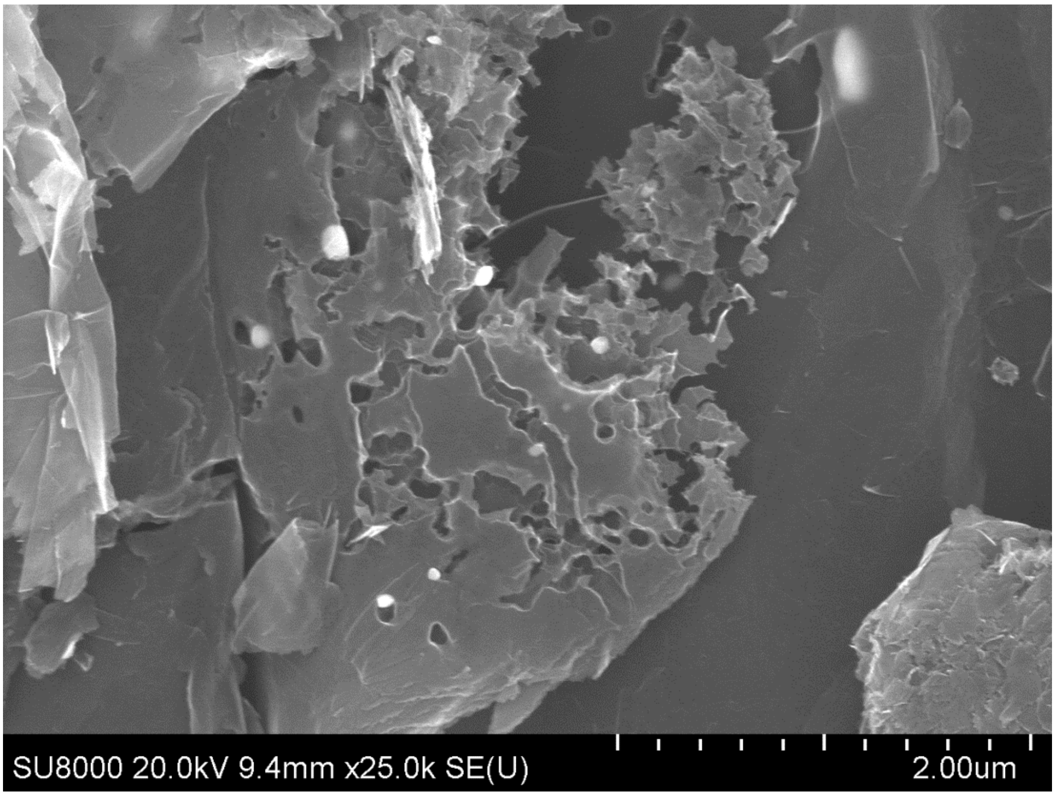

Figure S106. SEM image of graphite powder with Pt sample after MW treatment.

SEM images of changes in graphite morphology in the presence of Re after MW treatment

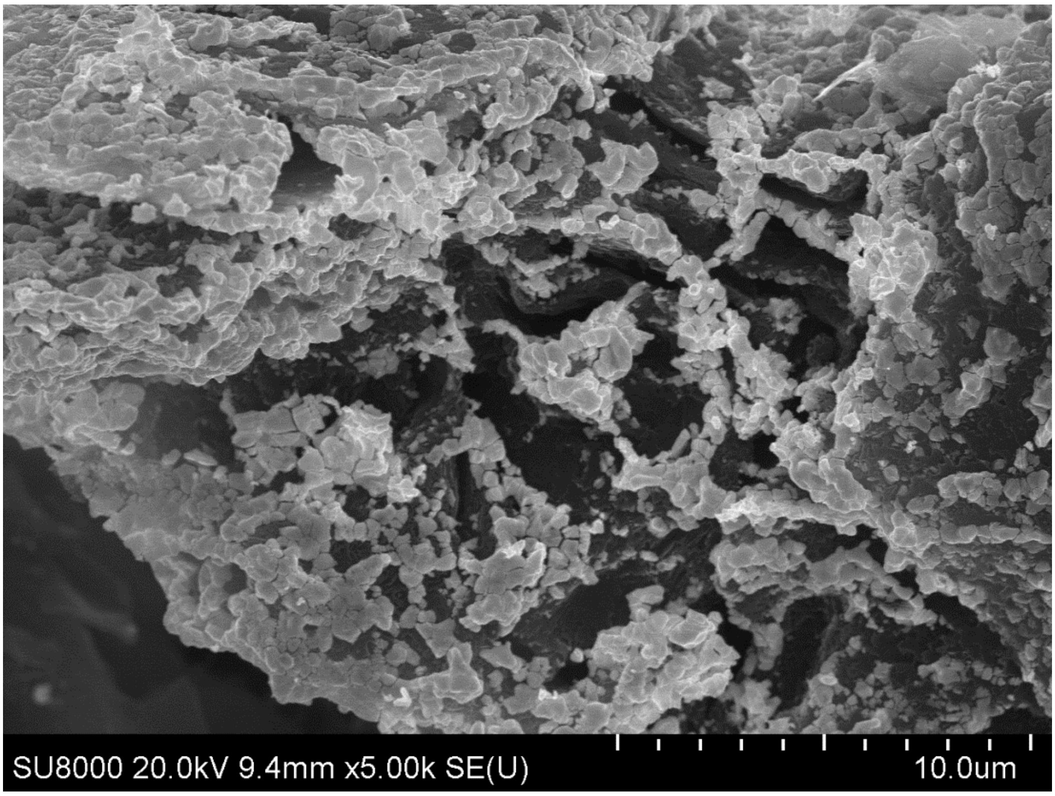

Figure S107. SEM image of graphite powder with Re sample after MW treatment.

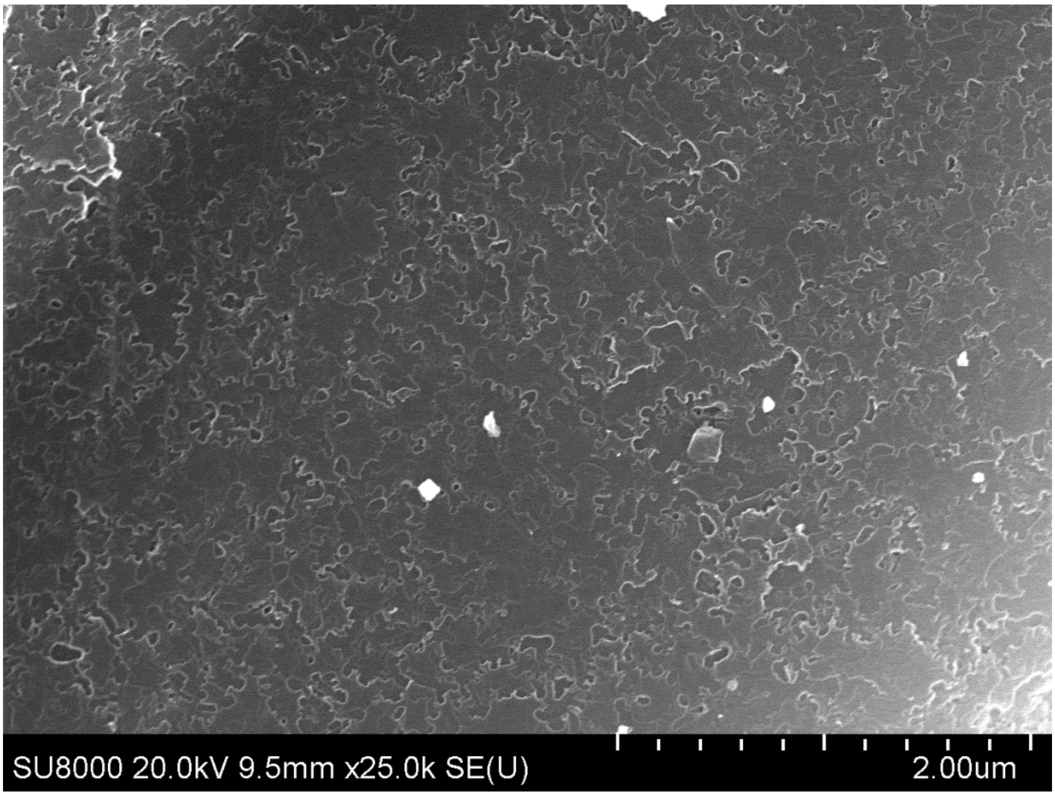

Figure S108. SEM image of graphite powder with Re sample after MW treatment.

SEM images of changes in graphite morphology in the presence of Ag after MW treatment

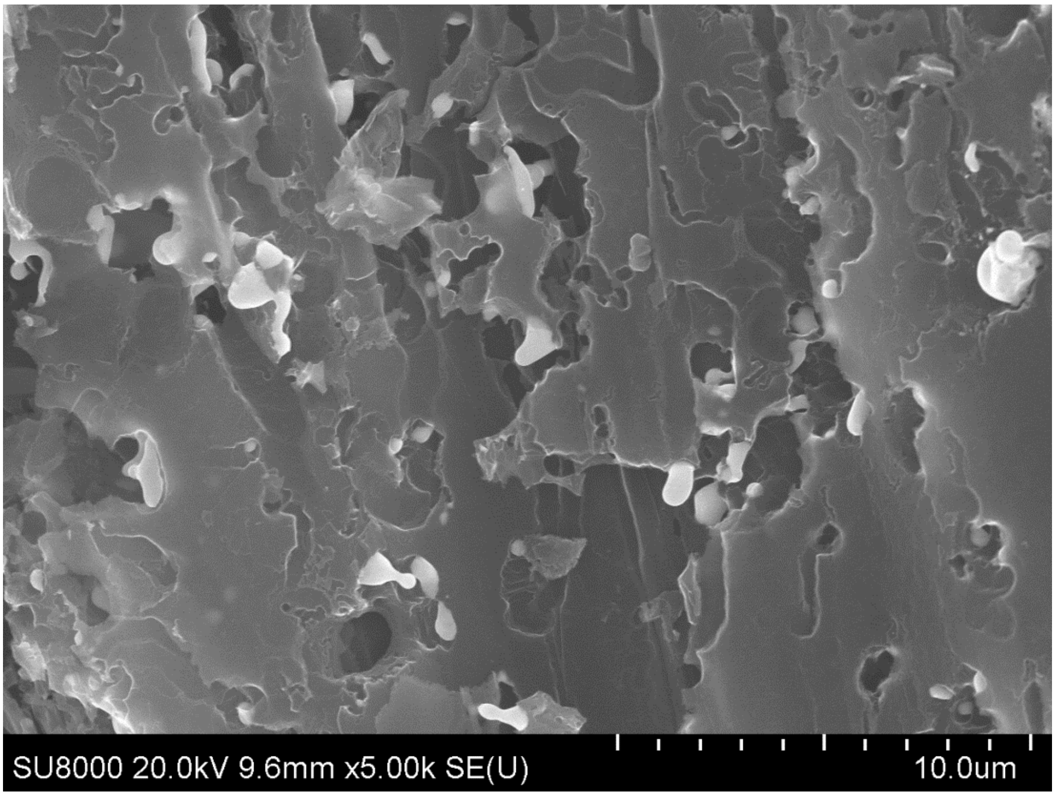

Figure S109. SEM image of graphite powder with Ag sample after MW treatment.

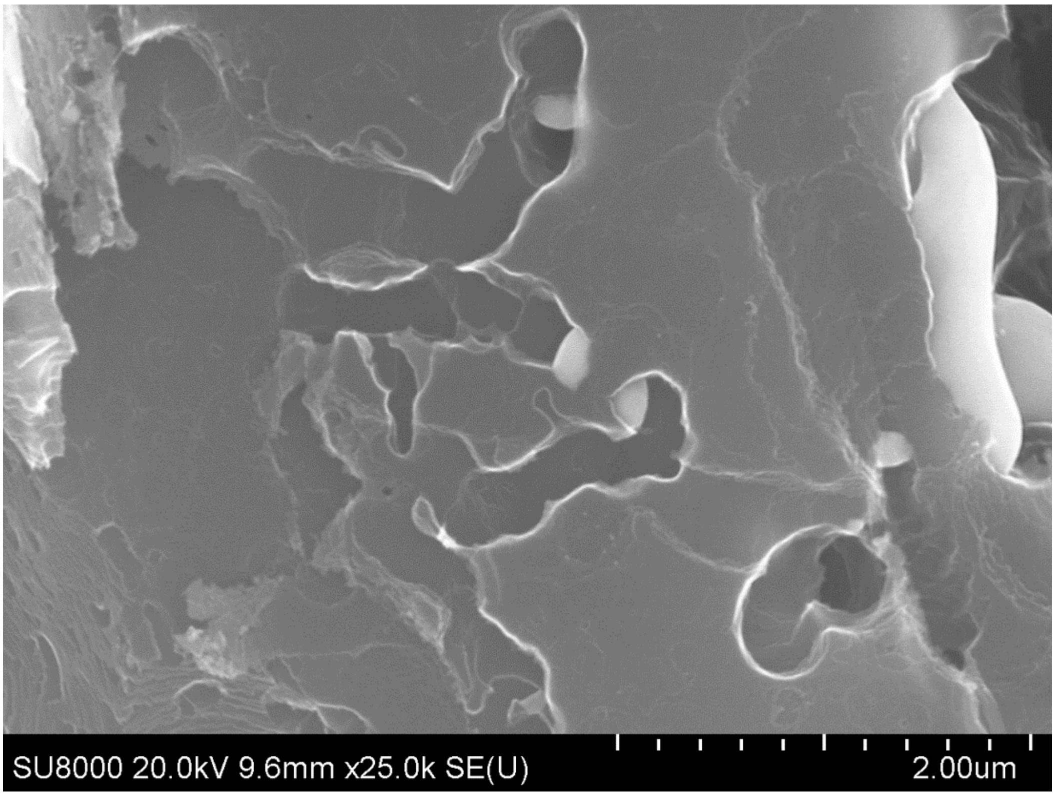

Figure S110. SEM image of graphite powder with Ag sample after MW treatment.

SEM images of changes in graphite morphology in the presence of Co after MW treatment

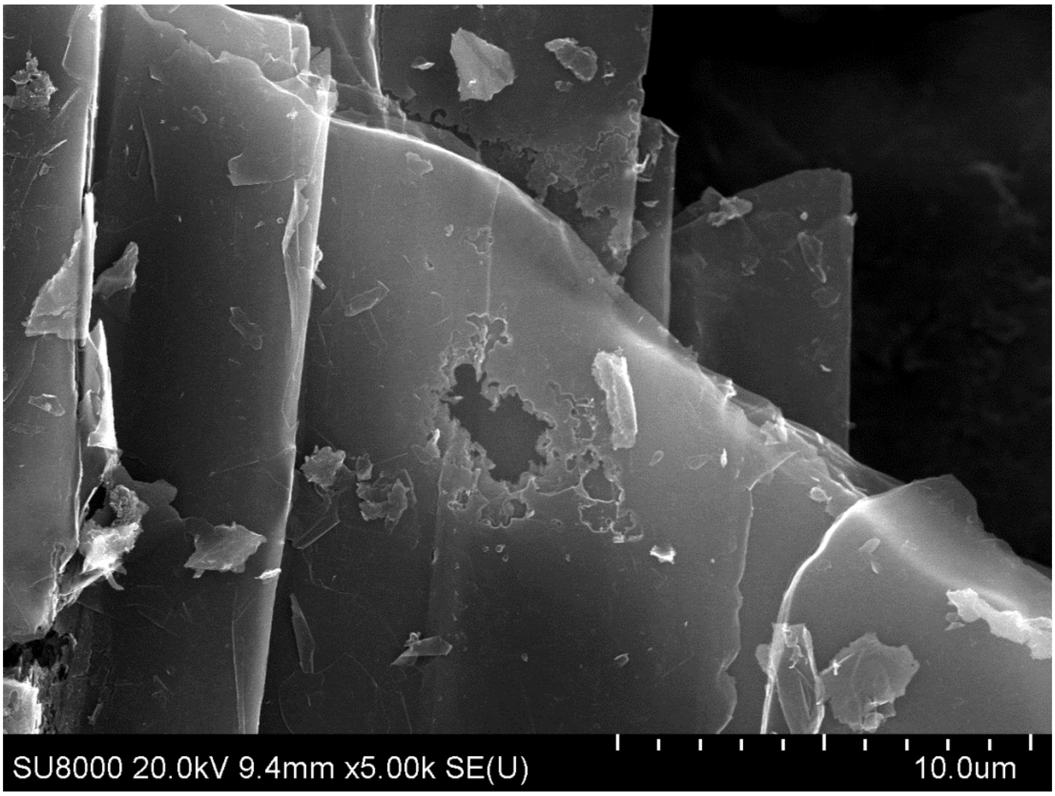

Figure S111. SEM image of graphite powder with Co sample after MW treatment.

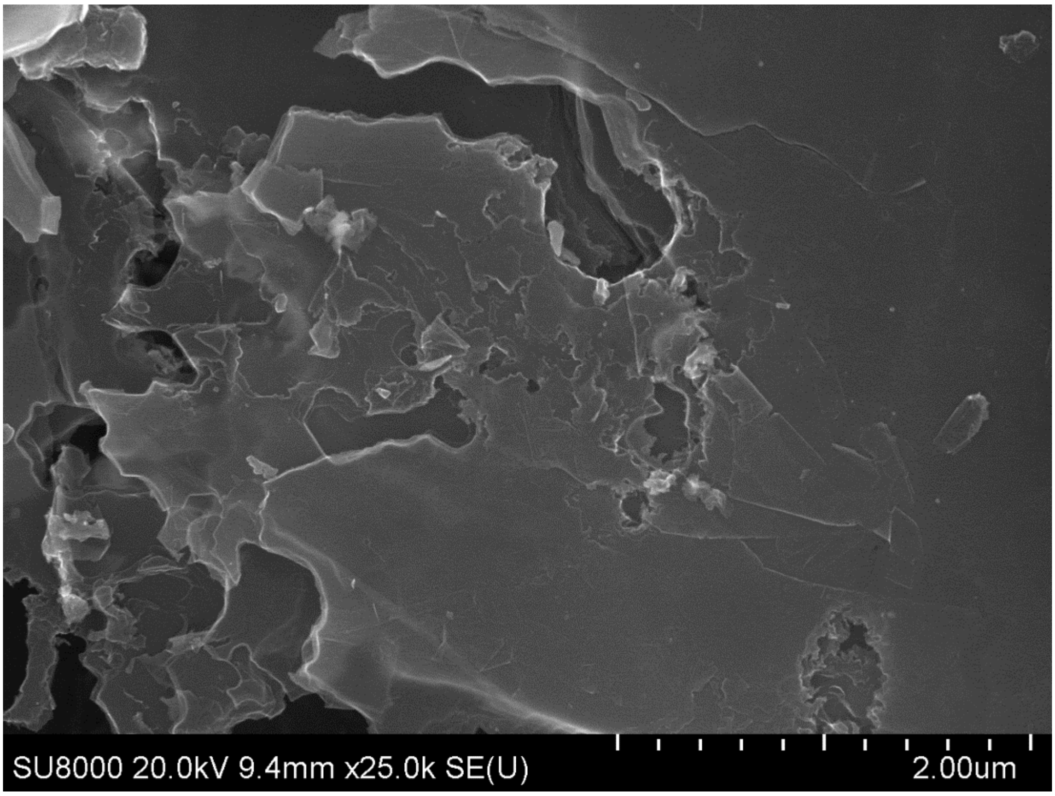

Figure S112. SEM image of graphite powder with Co sample after MW treatment.

SEM images of changes in graphite morphology in the presence of Fe/C after MW treatment

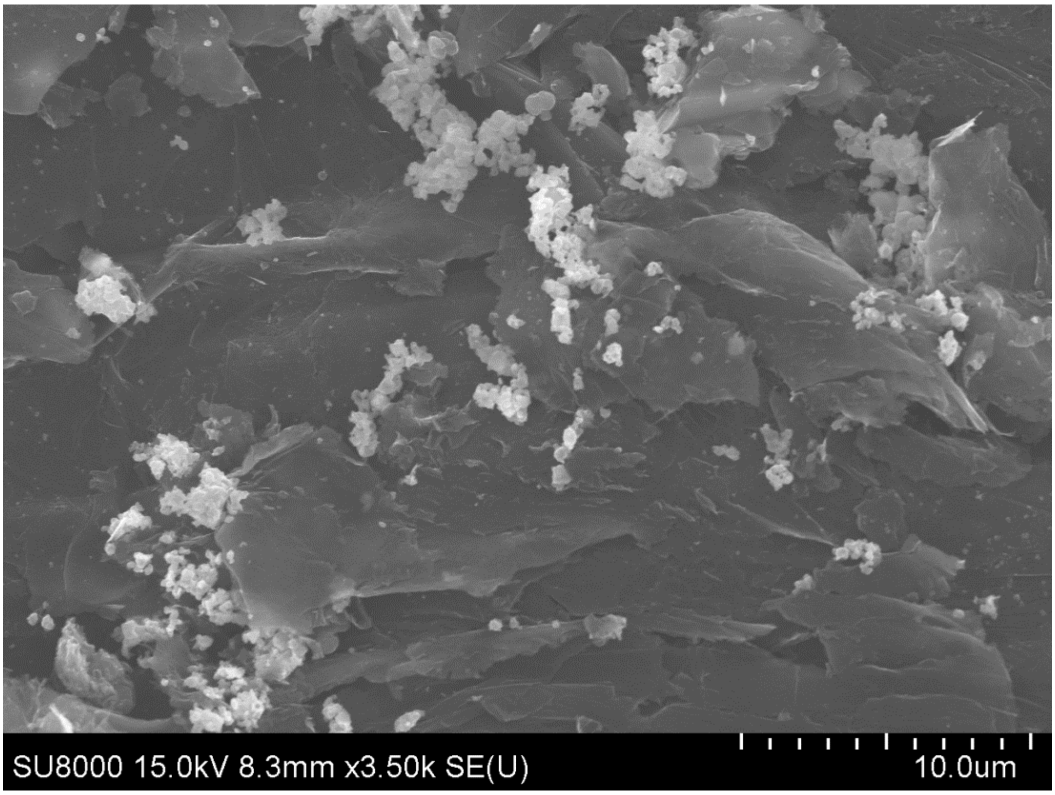

Figure S113. SEM image of graphite powder with Fe/C sample after MW treatment.

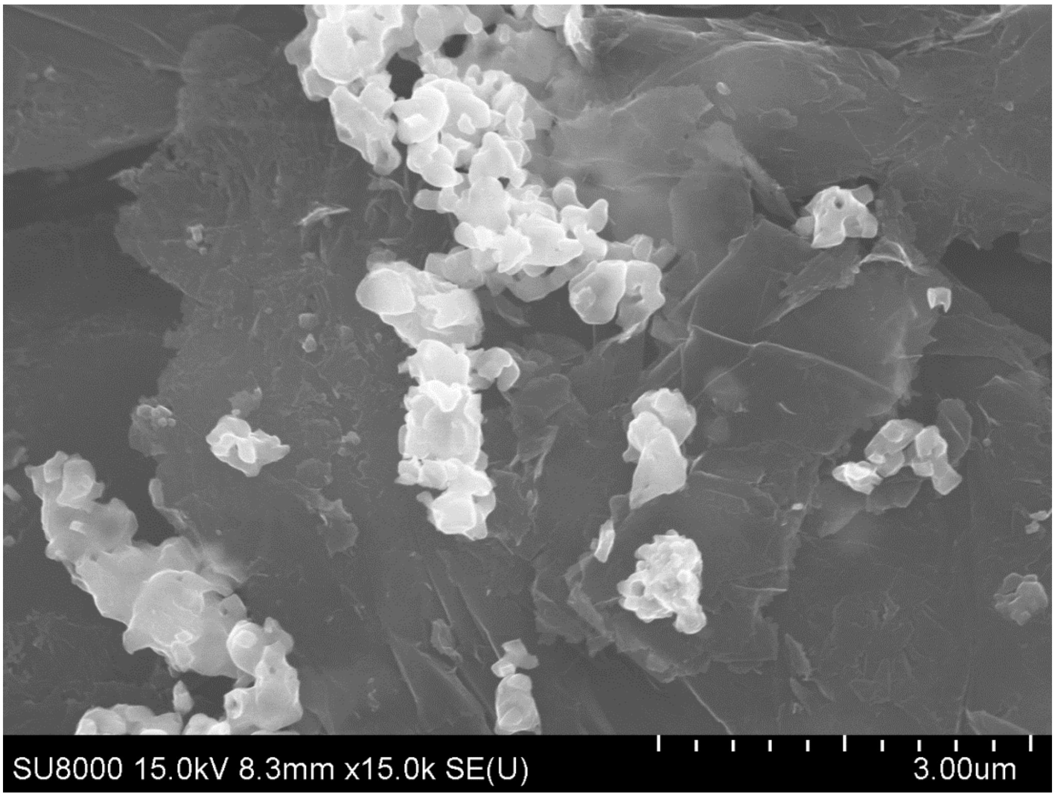

Figure S114. SEM image of graphite powder with Fe/C sample after MW treatment.

SEM images of changes in graphite morphology in the presence of Ni after MW treatment

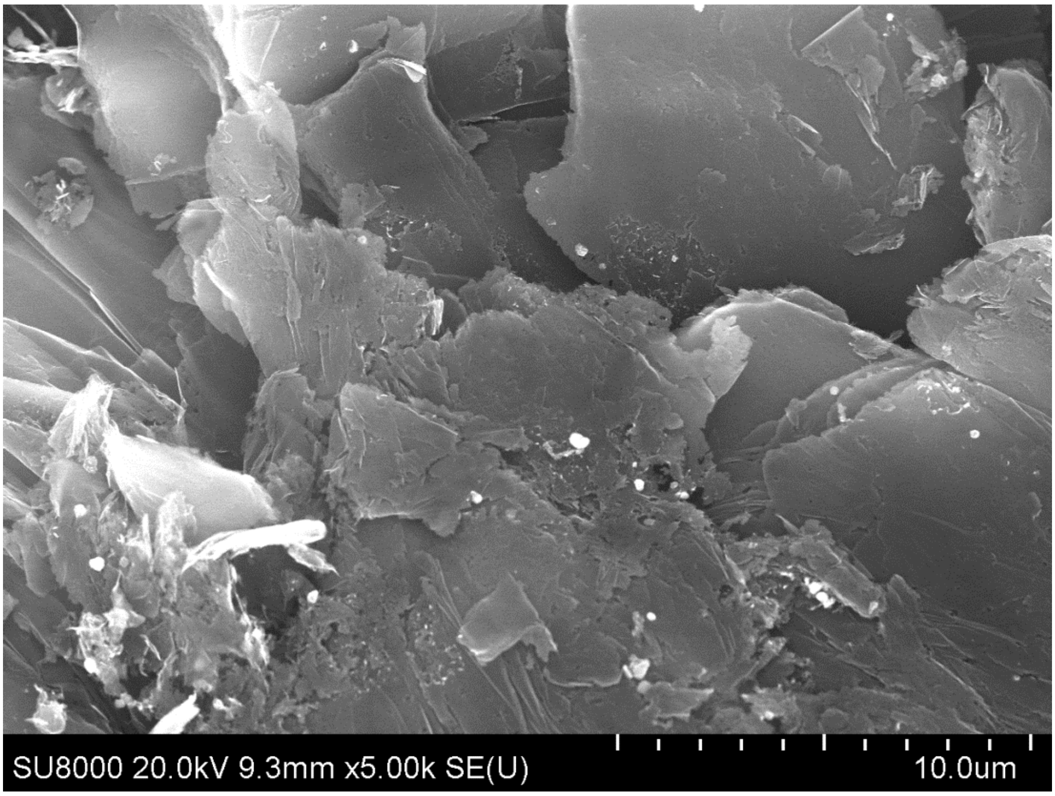

Figure S115. SEM image of graphite powder with Ni sample after MW treatment.

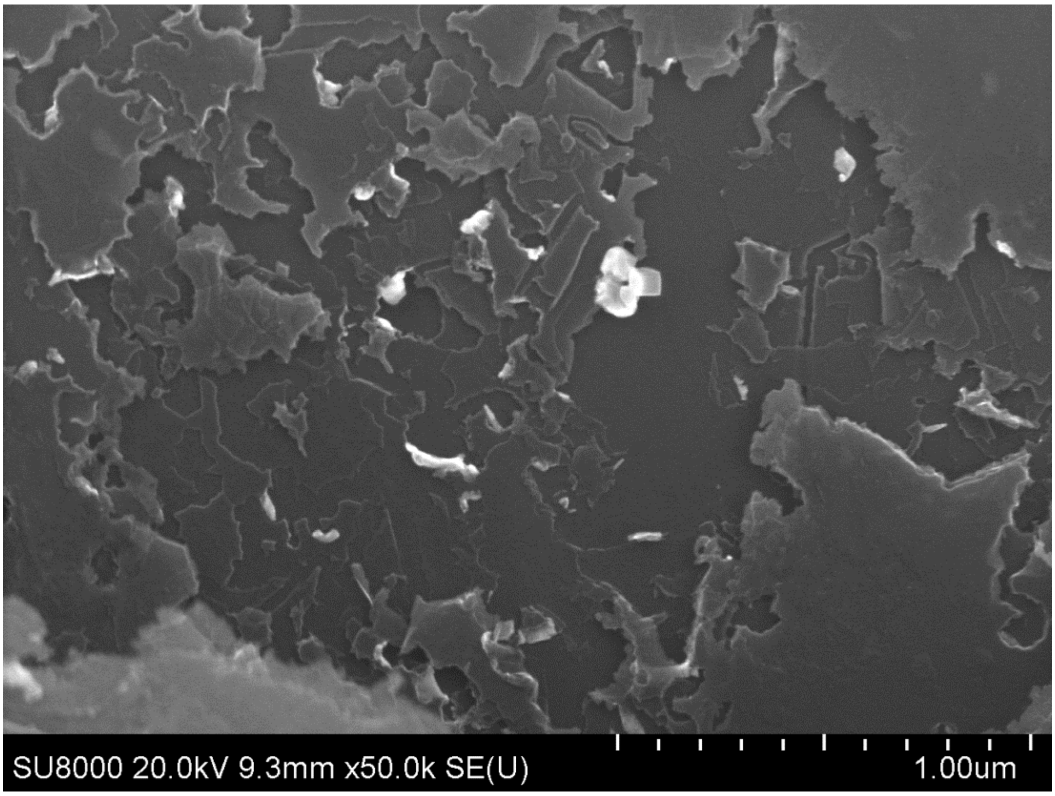

Figure S116. SEM image of graphite powder with Ni sample after MW treatment.

SEM images of changes in graphite morphology in the presence of Cu after MW treatment

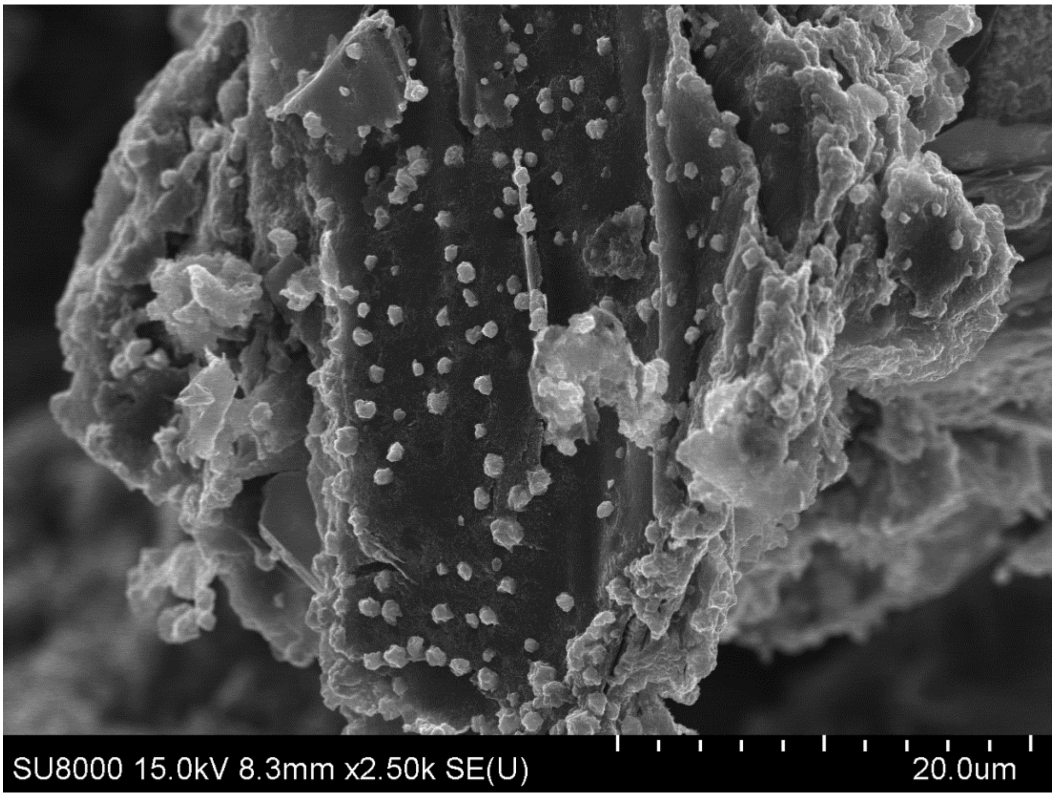

Figure S117. SEM image of graphite powder with Cu sample after MW treatment.

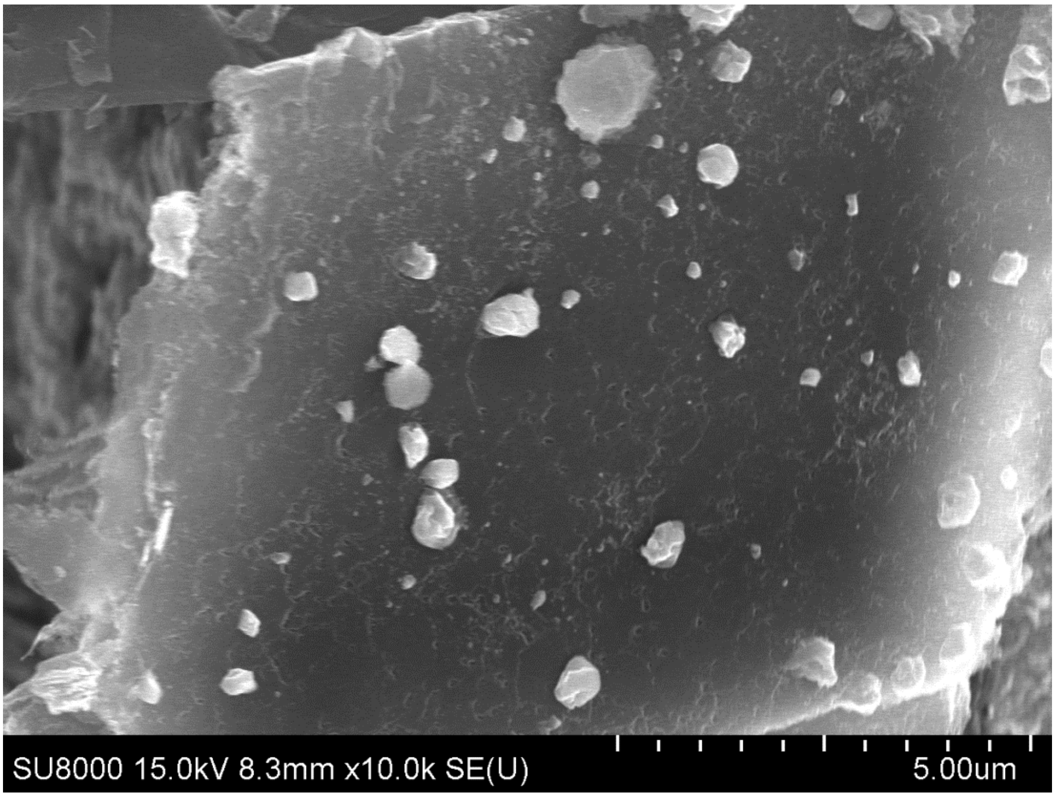

Figure S118. SEM image of graphite powder with Cu sample after MW treatment.

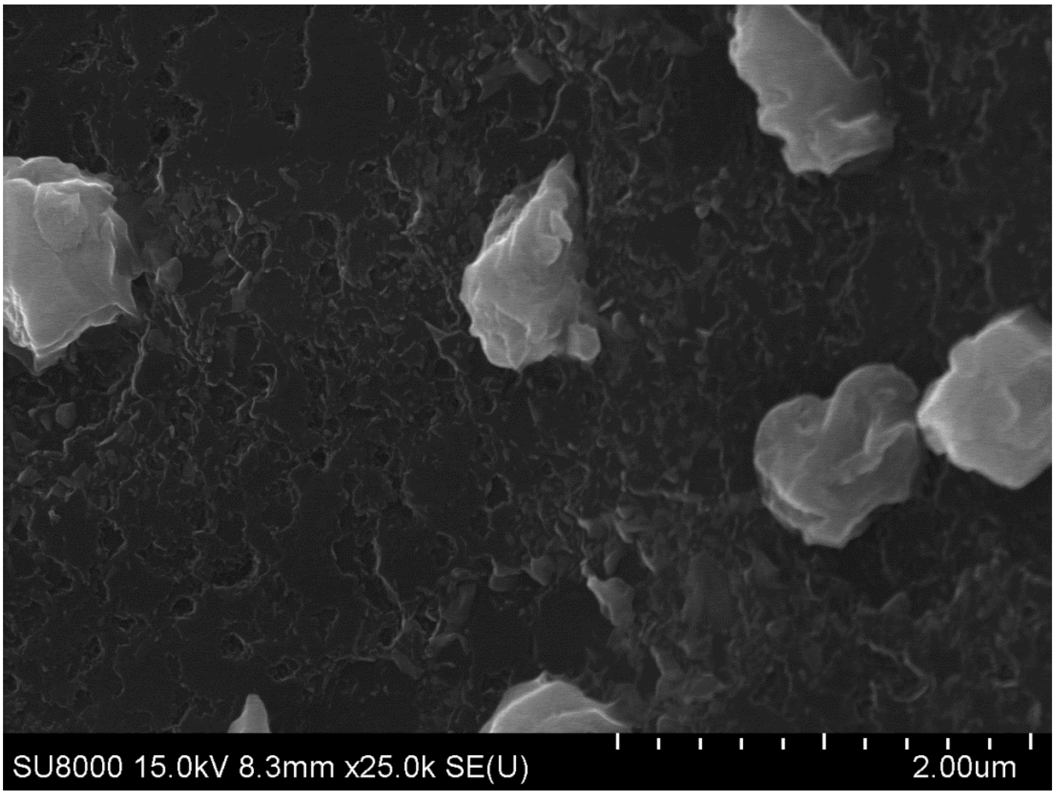

Figure S119. SEM image of graphite powder with Cu sample after MW treatment.

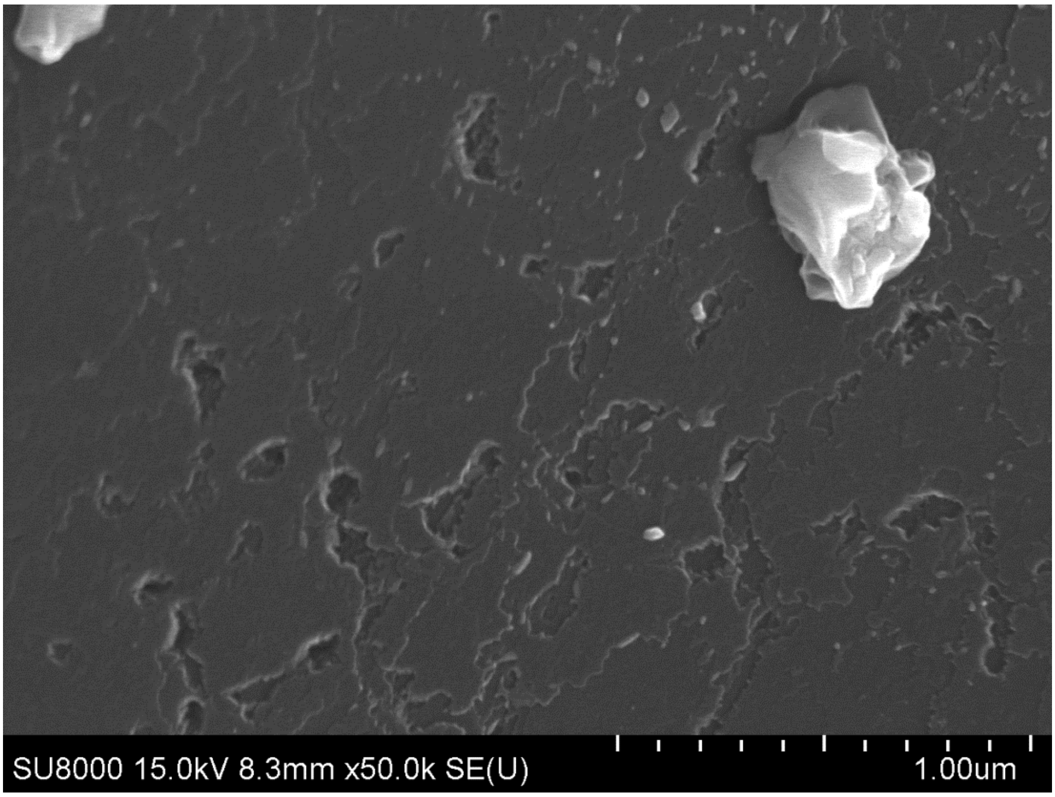

Figure S120. SEM image of graphite powder with Cu sample after MW treatment.

SEM images of changes in graphite morphology in the presence of Cu/C after MW treatment

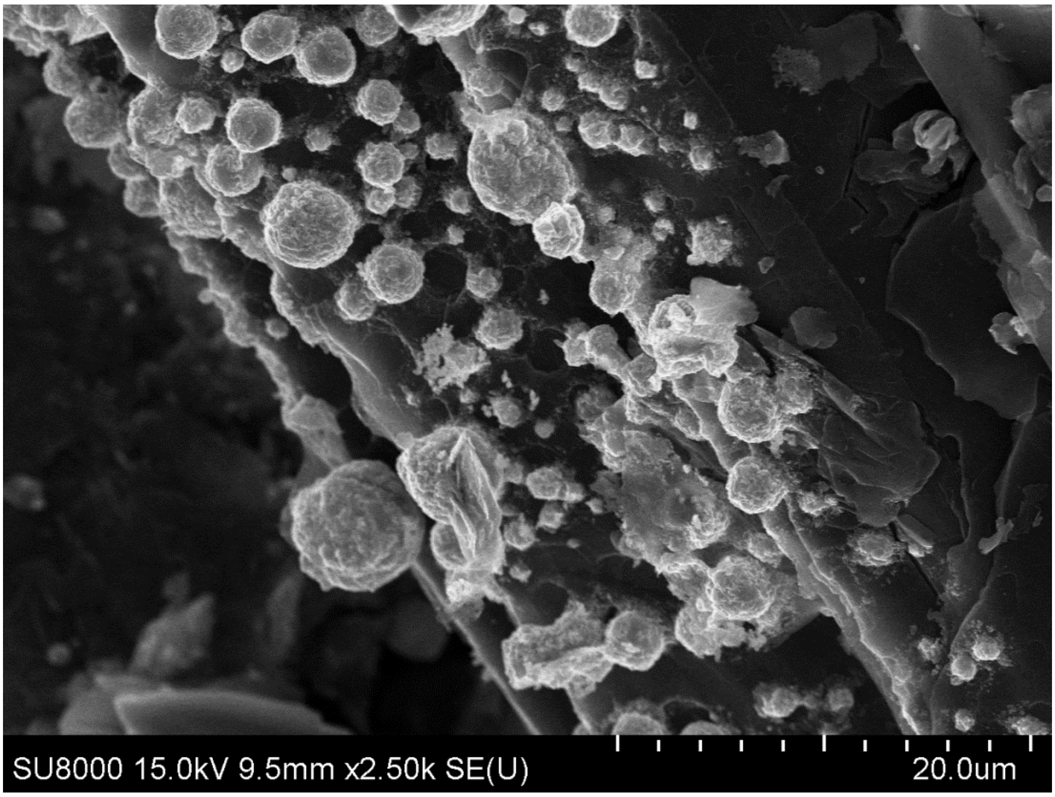

Figure S121. SEM image of graphite powder with Cu/C sample after MW treatment.

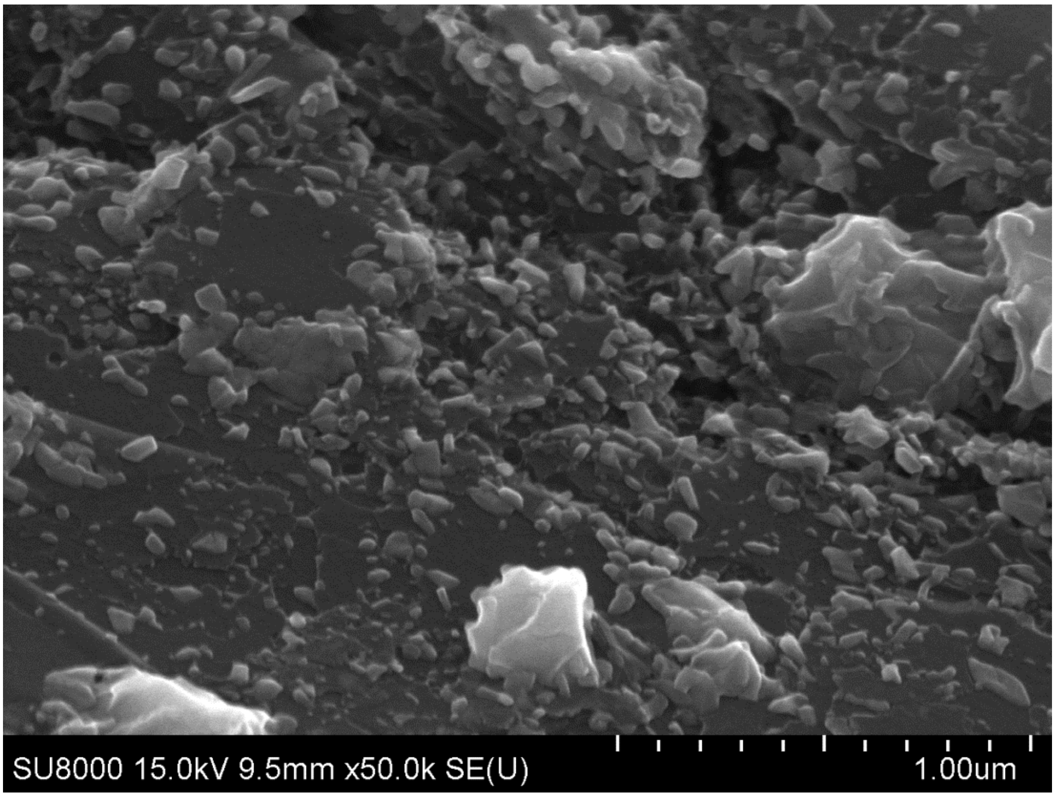

Figure S122. SEM image of graphite powder with Cu/C sample after MW treatment.

SEM images of changes in graphite morphology in the presence of W-Ni-Fe after MW treatment

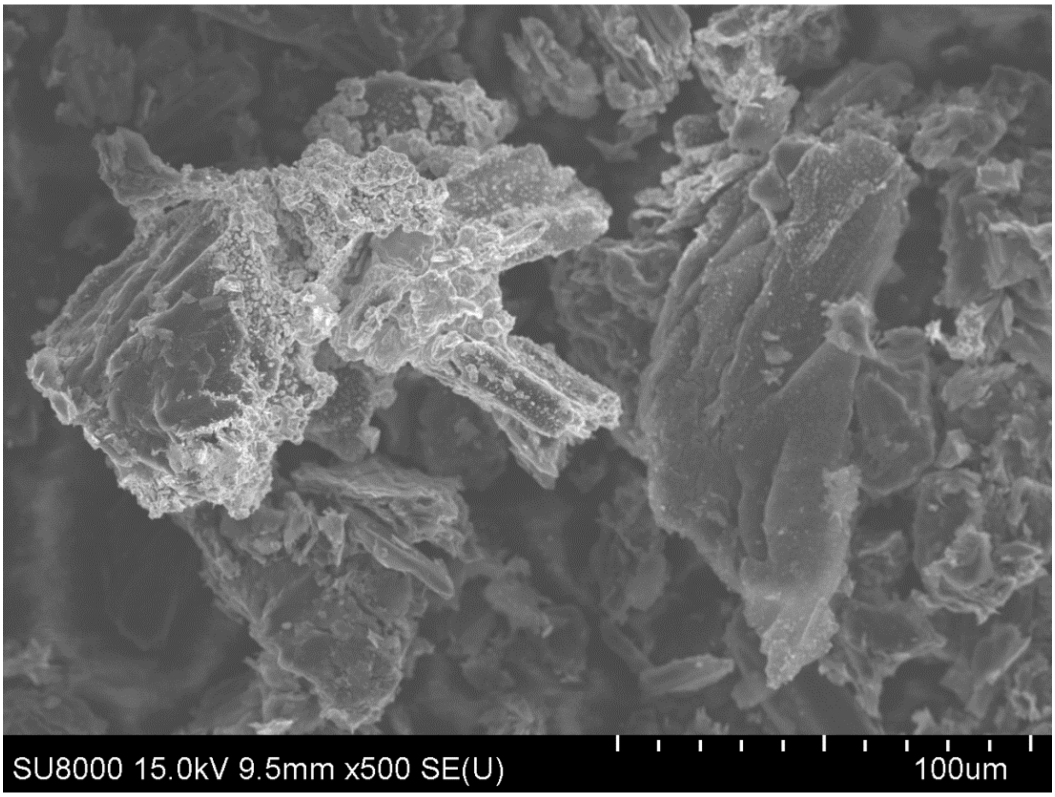

Figure S123. SEM image of graphite powder with W-Ni-Fe sample after MW treatment.

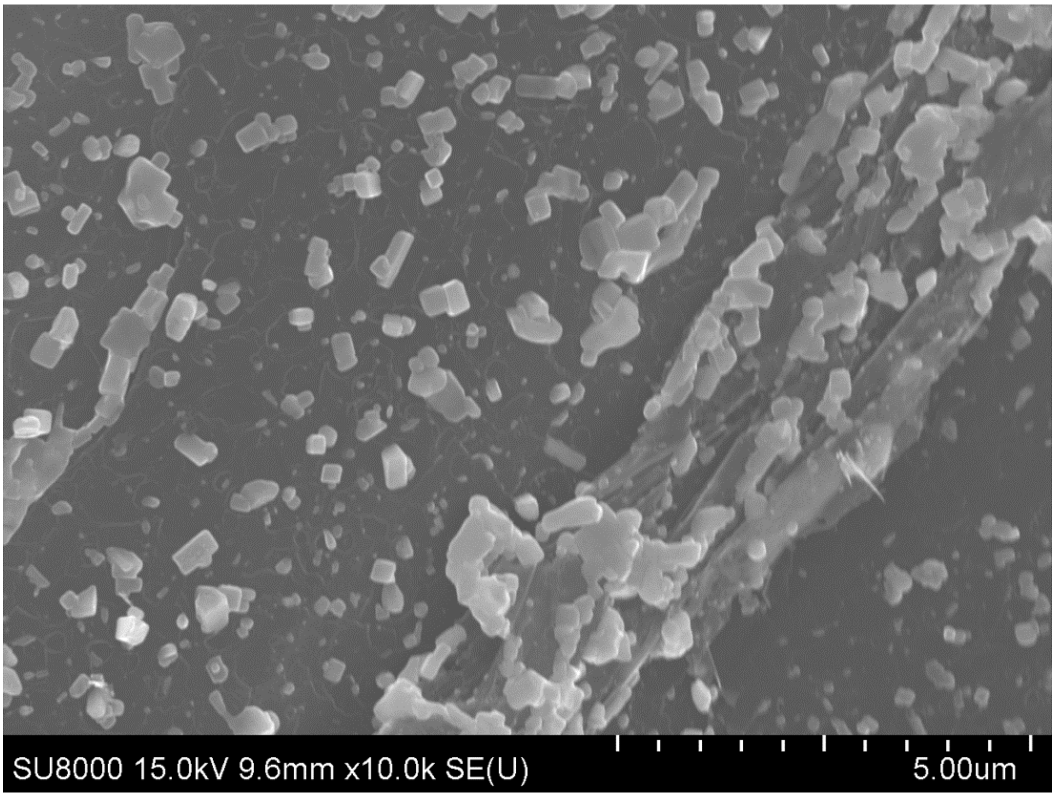

Figure S124. SEM image of graphite powder with W-Ni-Fe sample after MW treatment.

SEM images of changes in graphite morphology in the presence of Mo/C after MW treatment

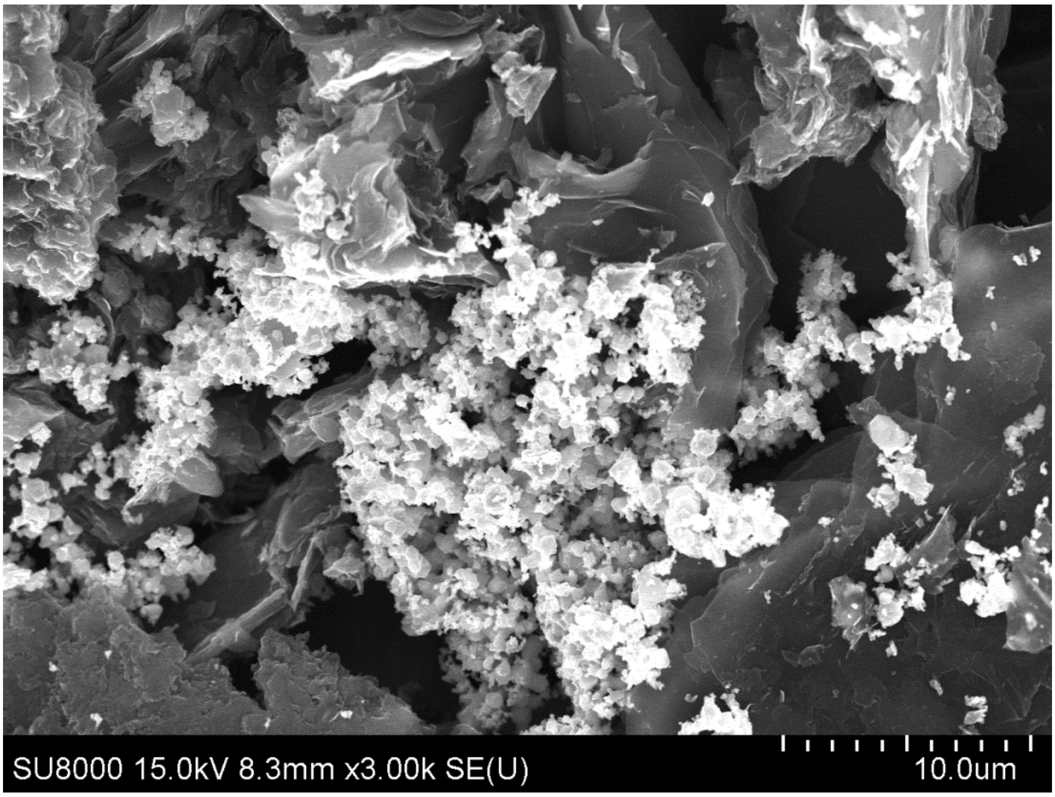

Figure S125. SEM image of graphite powder with Mo/C sample after MW treatment.

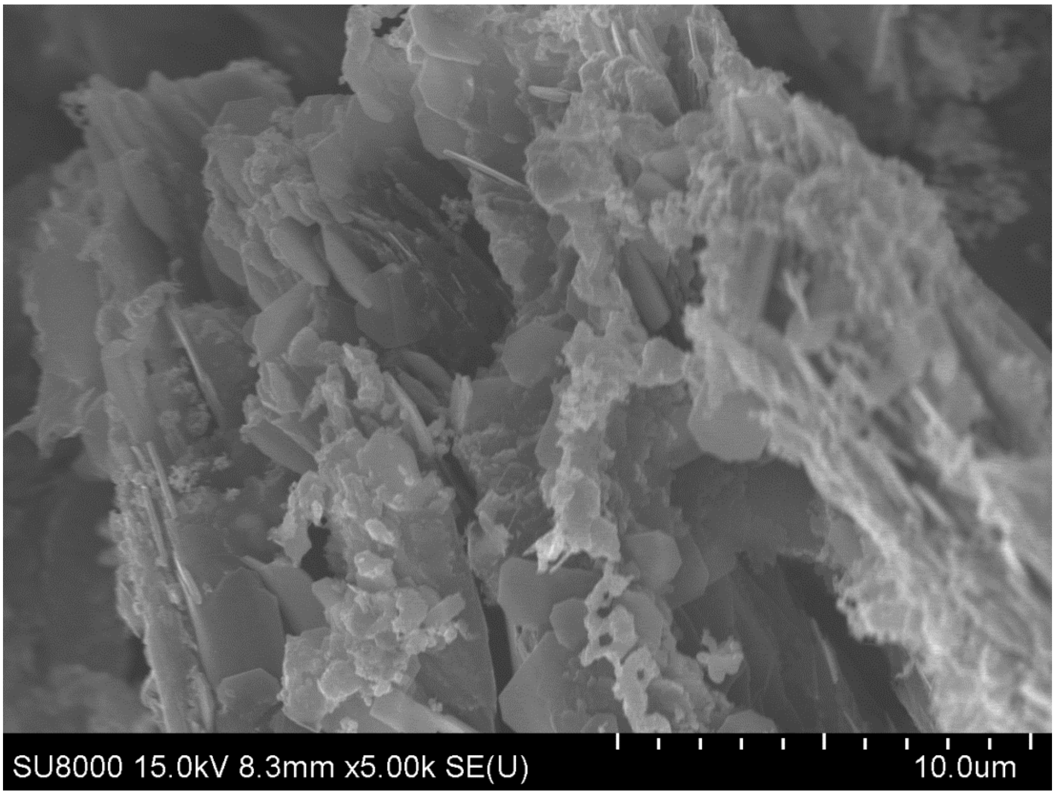

Figure S126. SEM image of graphite powder with Mo/C sample after MW treatment.

SEM images of changes in graphite morphology in the presence of Mo-Fe-C after MW treatment

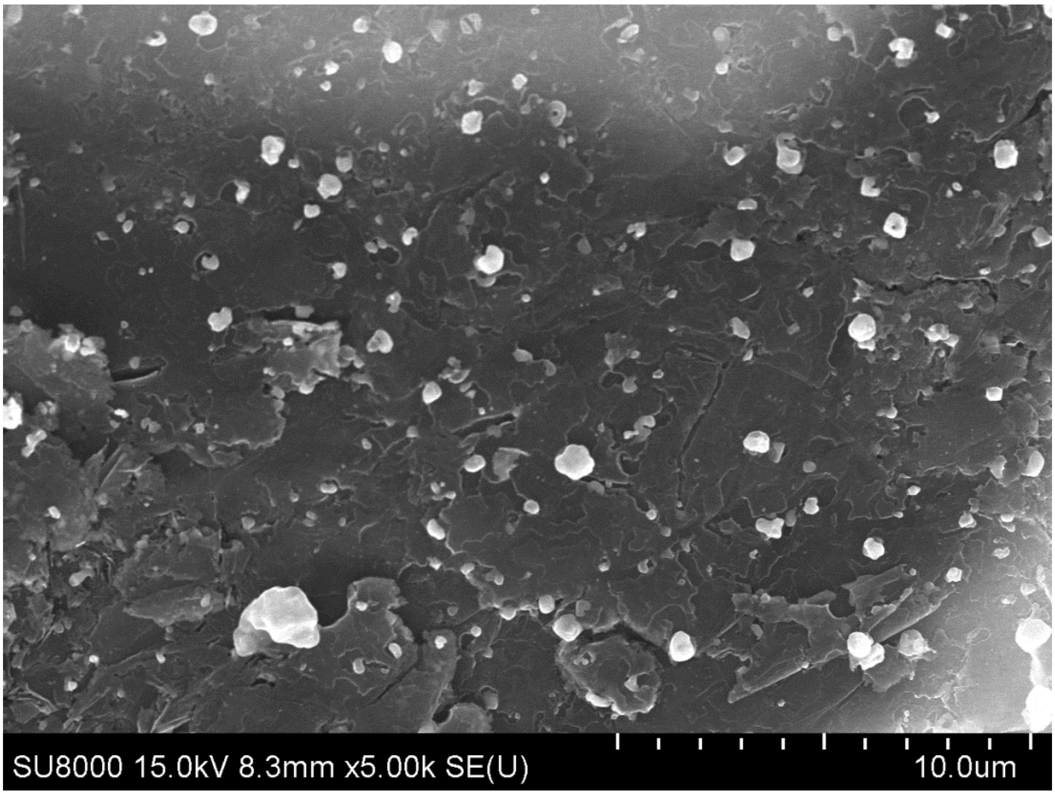

Figure S127. SEM image of graphite powder with Mo-Fe-C sample after MW treatment.

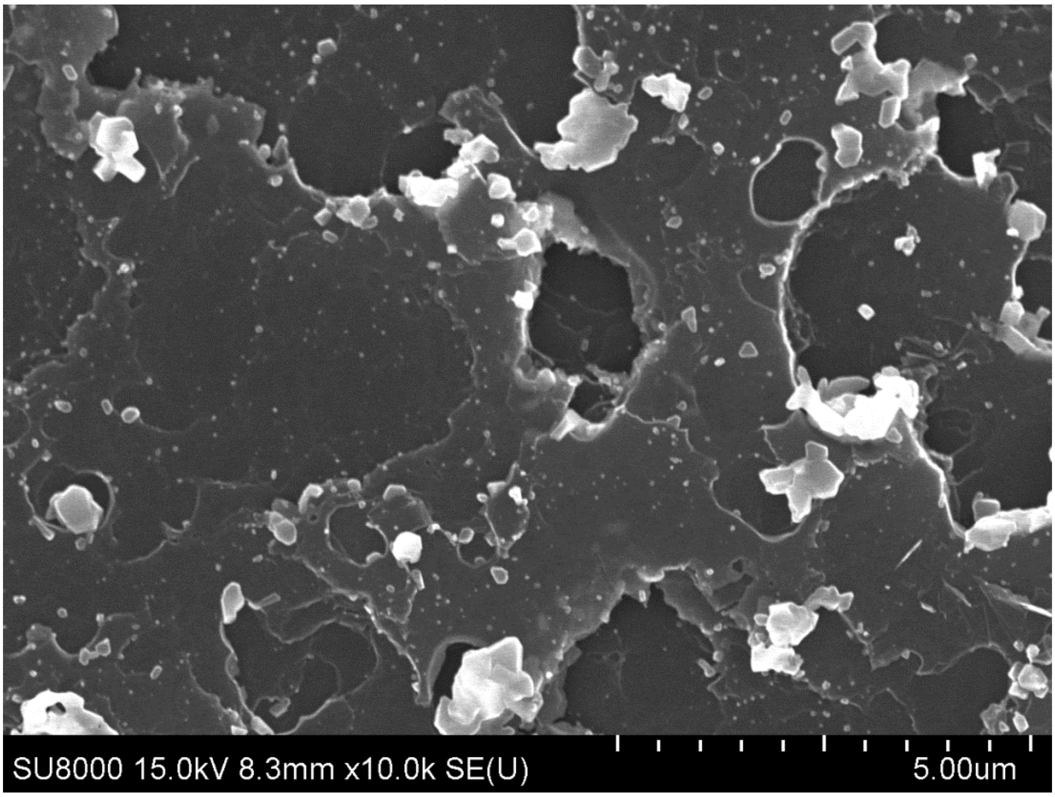

Figure S128. SEM image of graphite powder with Mo-Fe-C sample after MW treatment.

SEM images of changes in graphite morphology in the presence of Cu-W after MW treatment

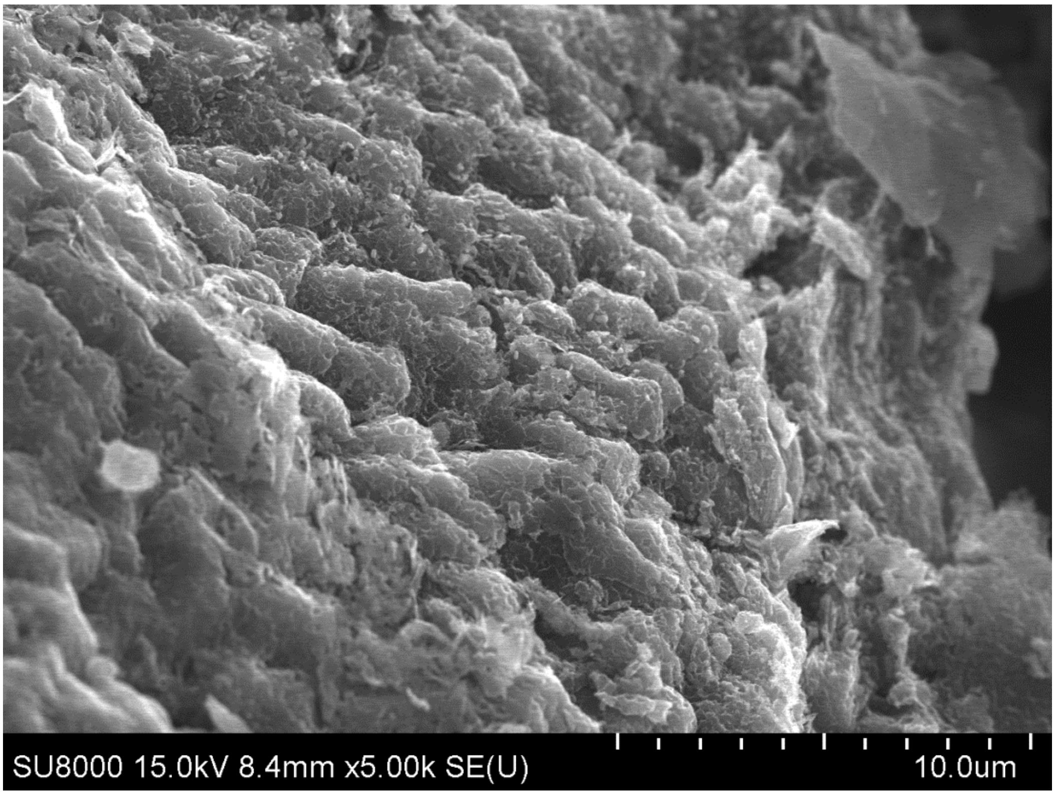

Figure S129. SEM image of graphite powder with Cu-W sample after MW treatment.

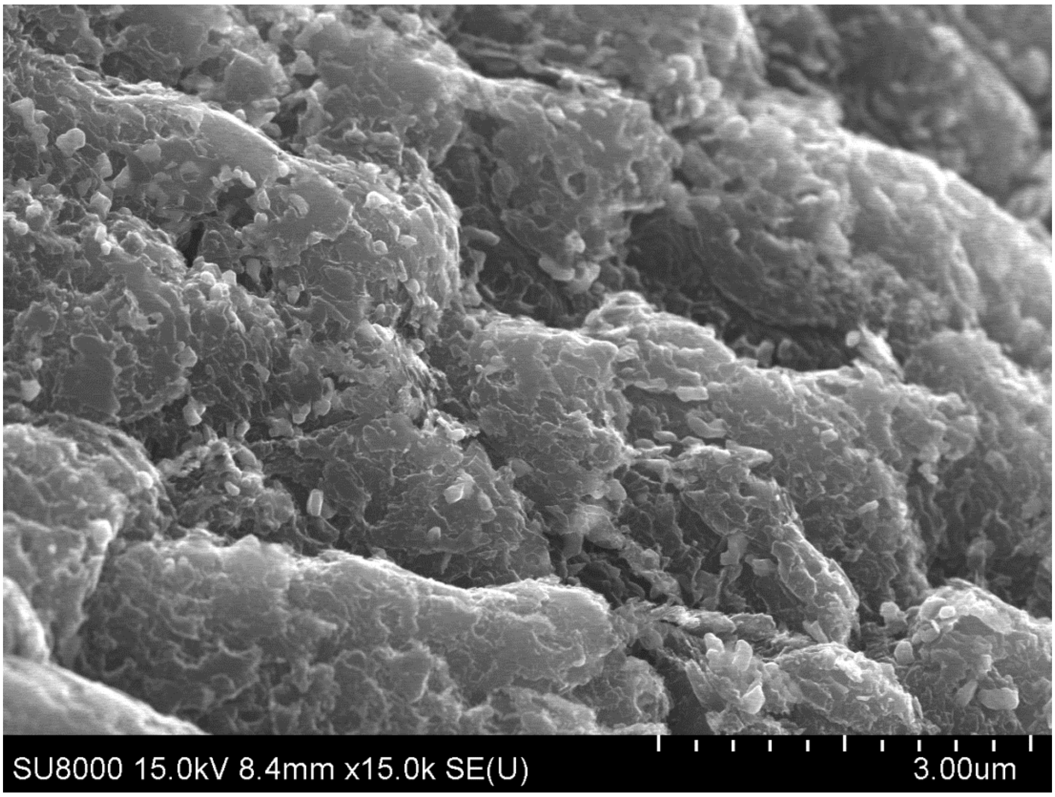

Figure S130. SEM image of graphite powder with Cu-W sample after MW treatment.

SEM images of changes in graphite morphology in the presence of W-Cu after MW treatment

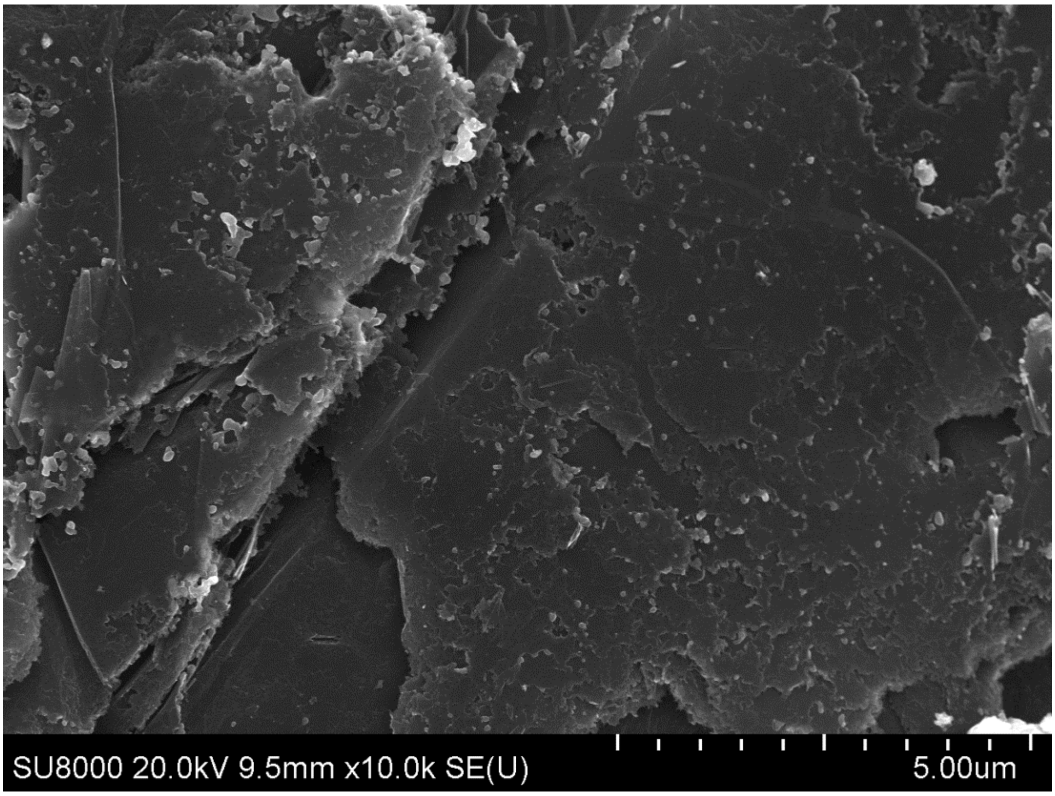

Figure S131. SEM image of graphite powder with W-Cu sample after MW treatment.

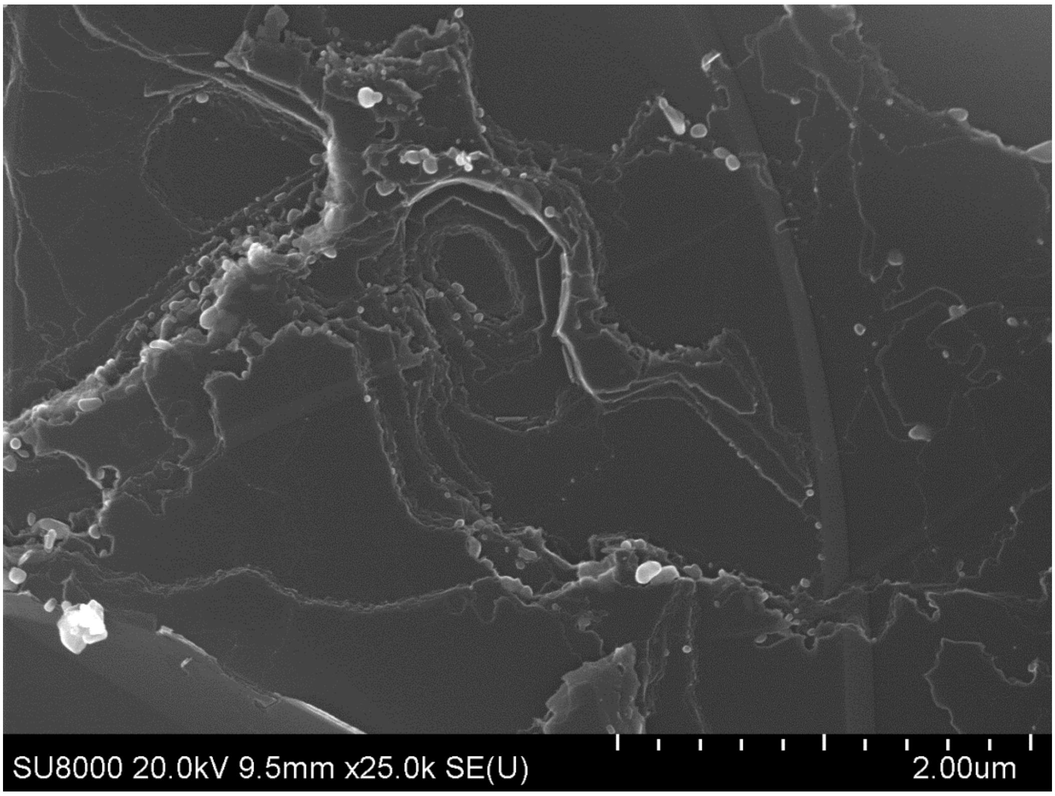

Figure S132. SEM image of graphite powder with W-Cu sample after MW treatment.

SEM images of changes in graphite morphology in the presence of WC after MW treatment

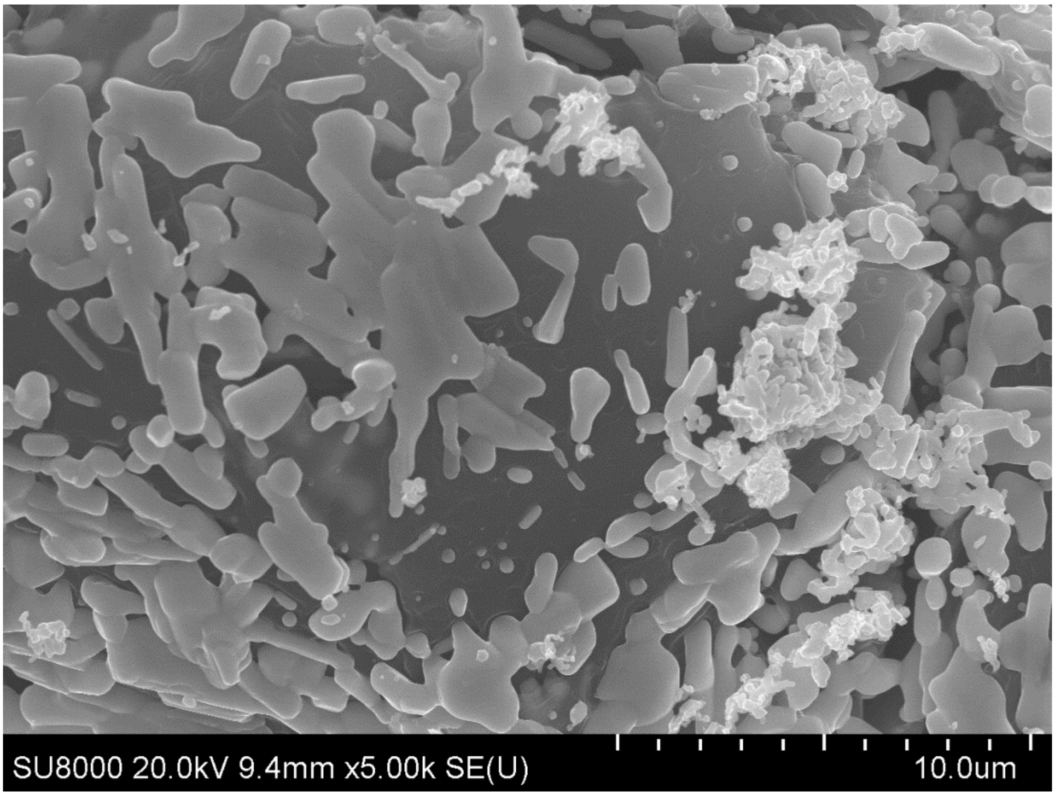

Figure S133. SEM image of graphite powder with WC sample after MW treatment.

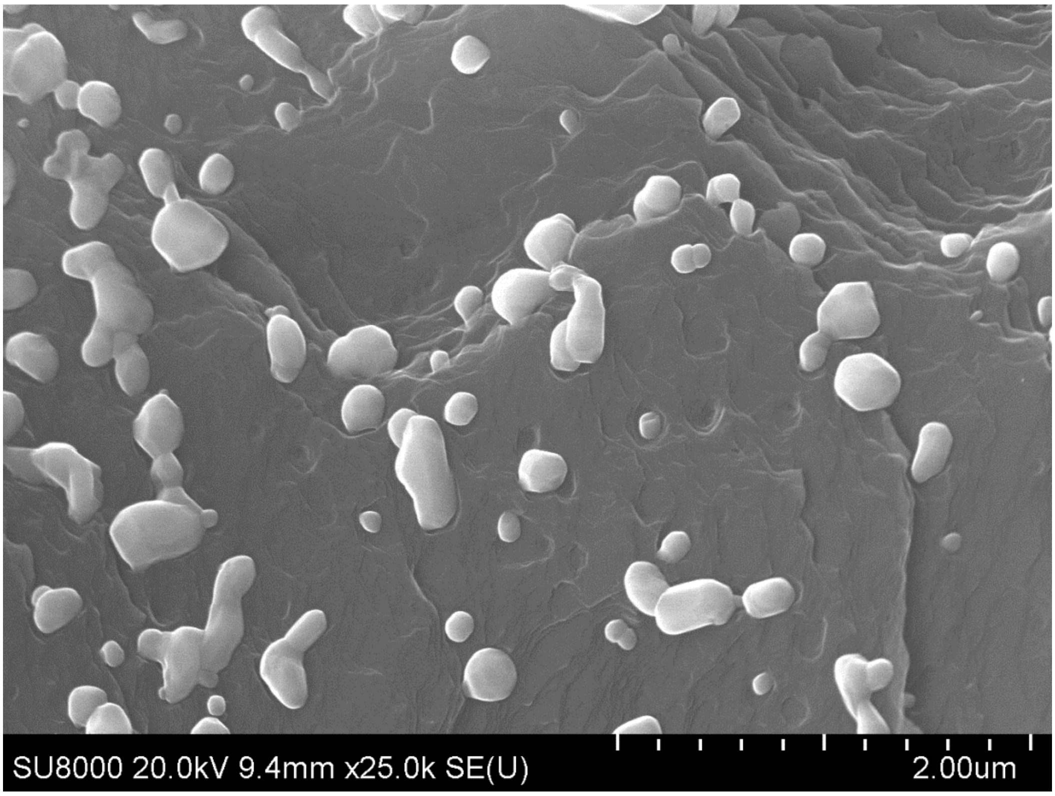

Figure S134. SEM image of graphite powder with WC sample after MW treatment.

EDX data of graphite powder with WC sample after MW treatment

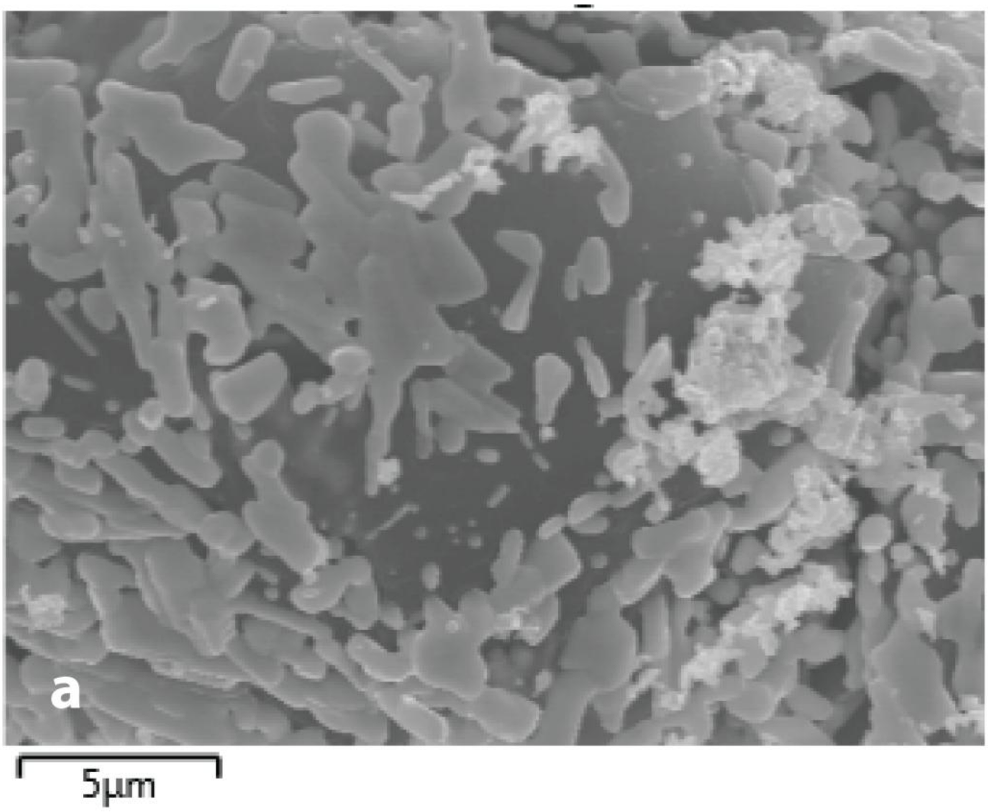

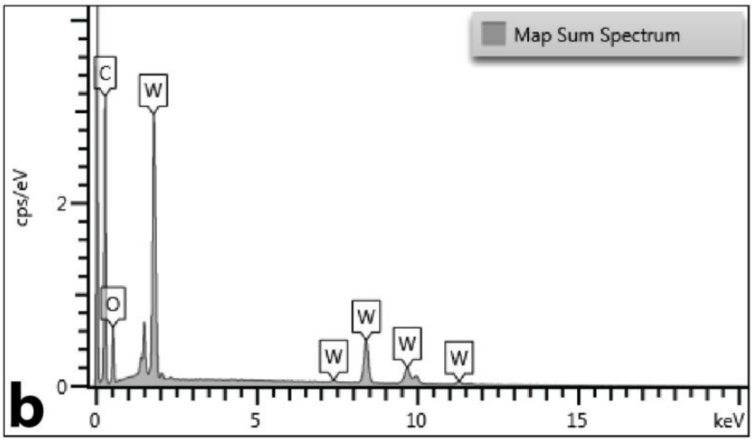

c

Element	Wt%	At%
C	10.87	57.49
W	85.88	29.63
O	3.25	12.88
Total:	100	100

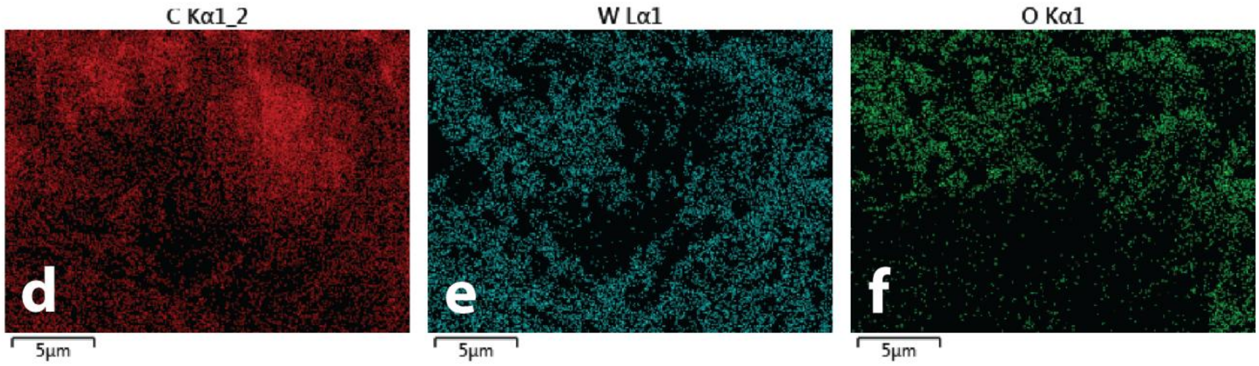

Figure S135. EDX study of graphite powder with WC sample after MW treatment: SEM image (a); EDX spectrum of this area (b); element composition (c) and maps of carbon (d), tungsten (e) and oxygen (f) distributions.

SEM images of changes in graphite morphology in the presence of AlN after MW treatment

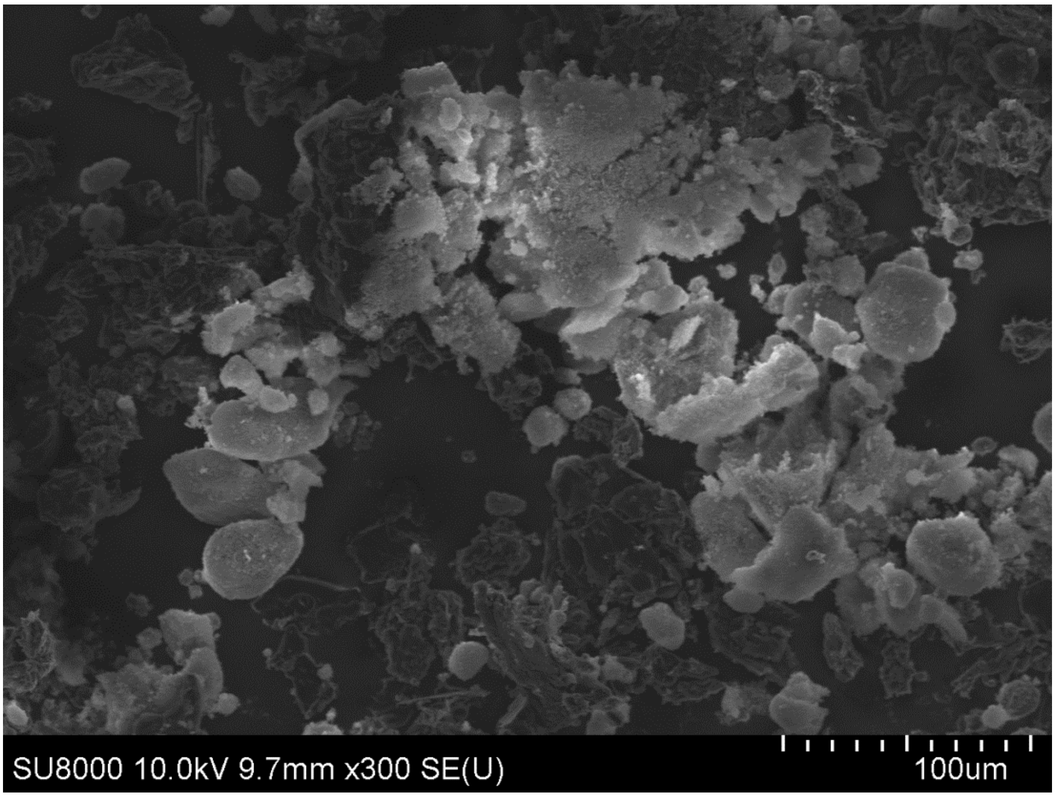

Figure S136. SEM image of graphite powder with AlN sample after MW treatment.

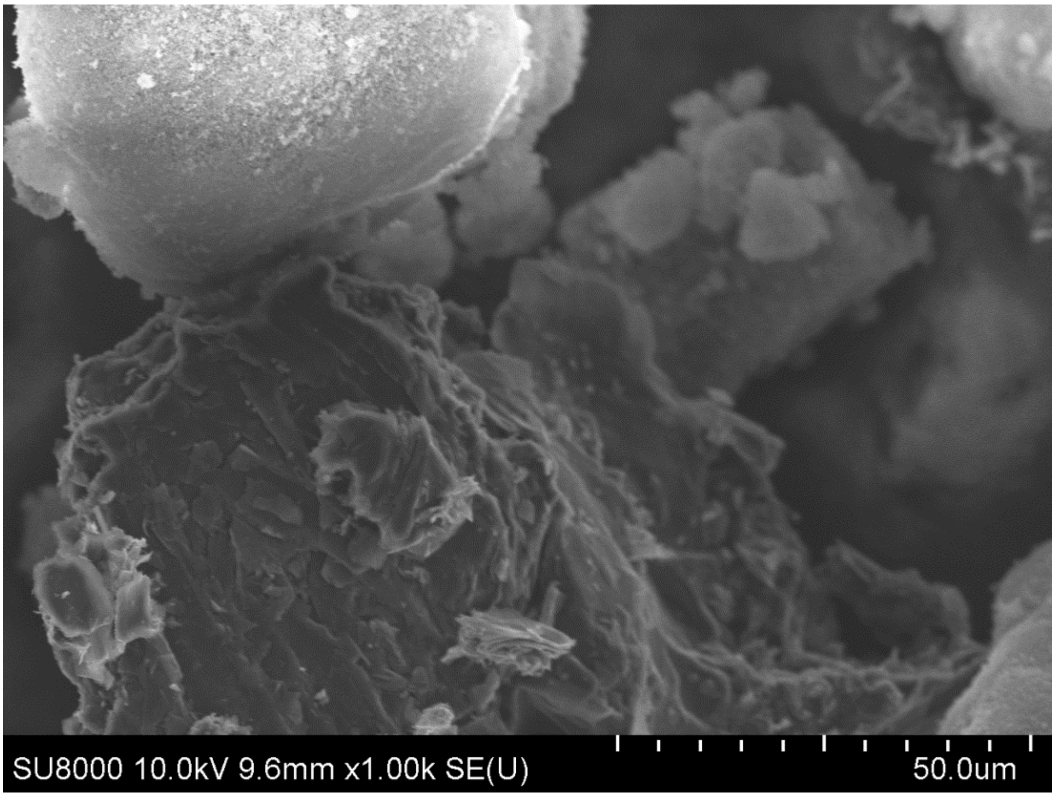

Figure S137. SEM image of graphite powder with AlN sample after MW treatment.

SEM images of changes in graphite morphology in the presence of AlON after MW treatment

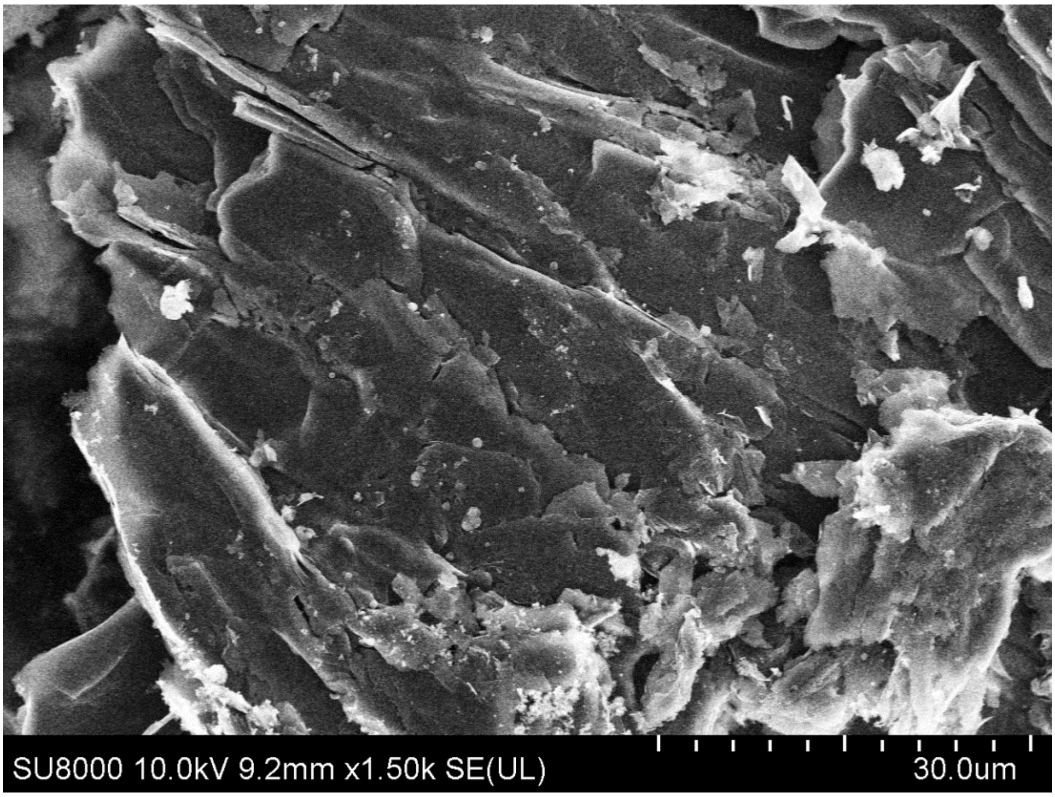

Figure S138. SEM image of graphite powder with AlON sample after MW treatment.

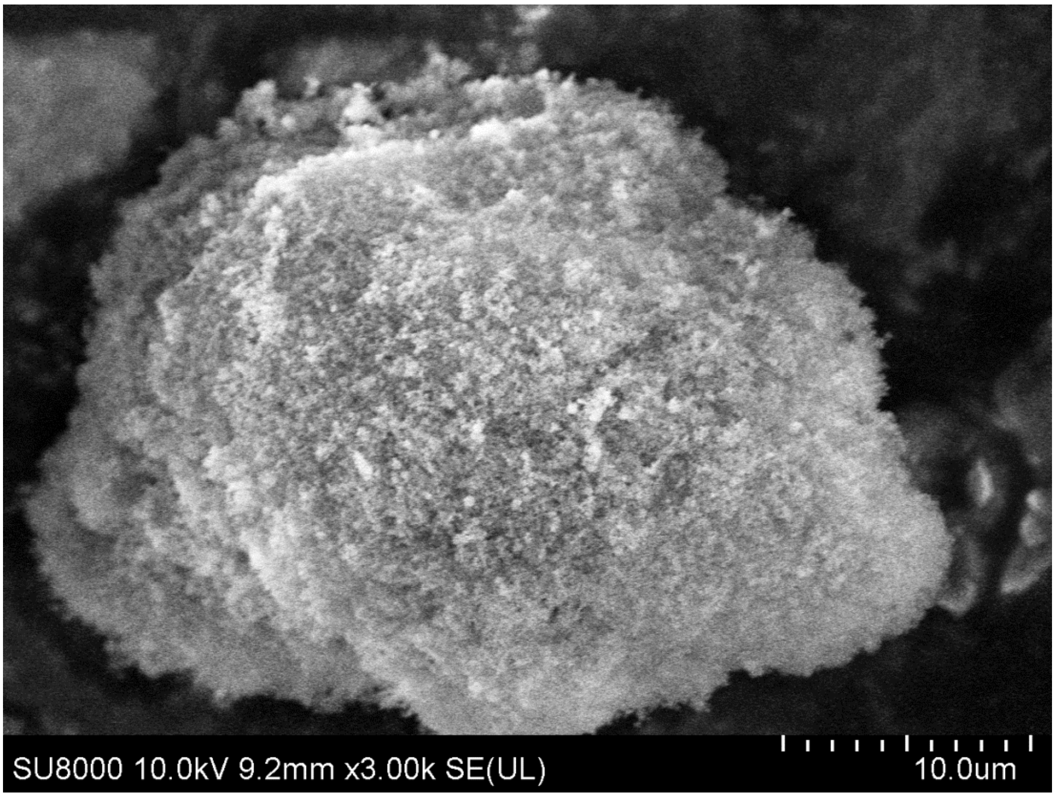

Figure S139. SEM image of graphite powder with AlON sample after MW treatment.

SEM images of changes in graphite morphology in the presence of TiN after MW treatment

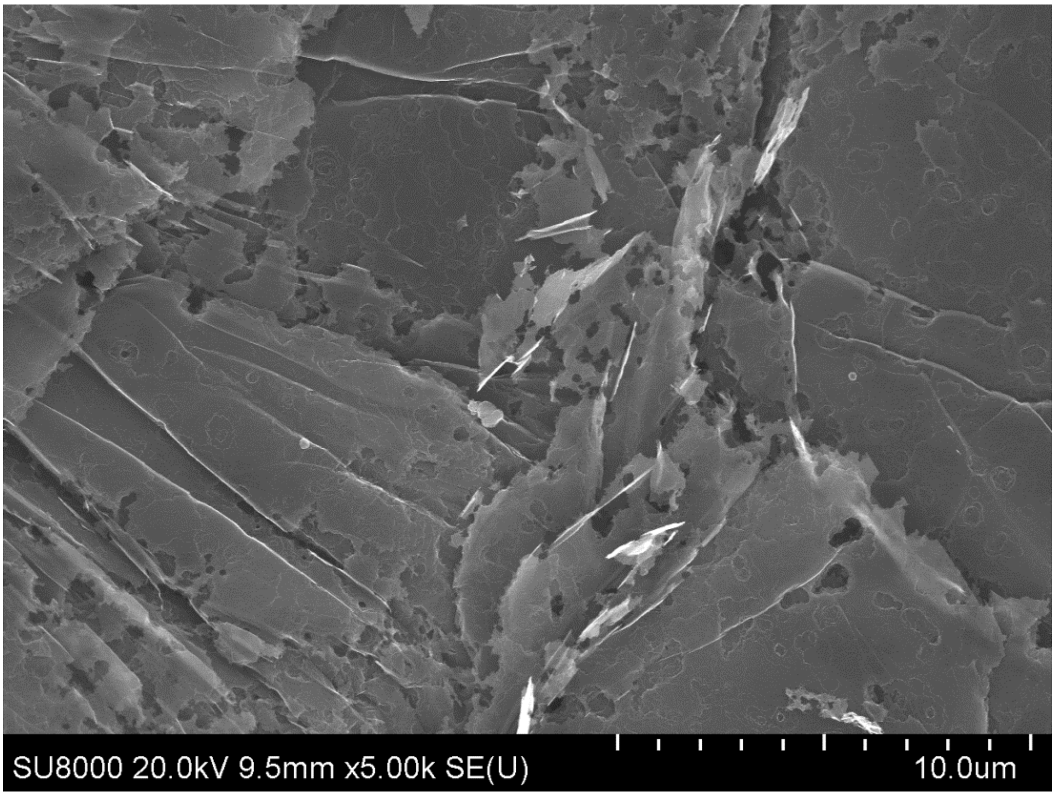

Figure S140. SEM image of graphite powder with TiN sample after MW treatment.

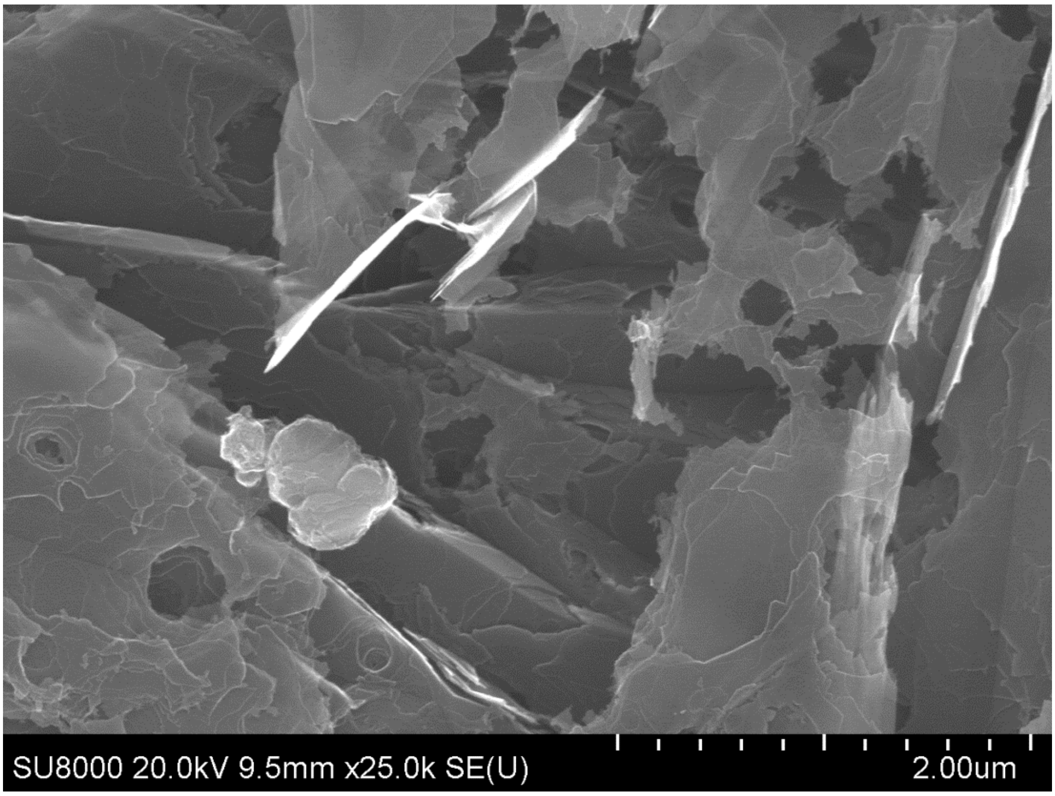

Figure S141. SEM image of graphite powder with TiN sample after MW treatment.

SEM images of changes in graphite morphology in the presence of TiCN after MW treatment

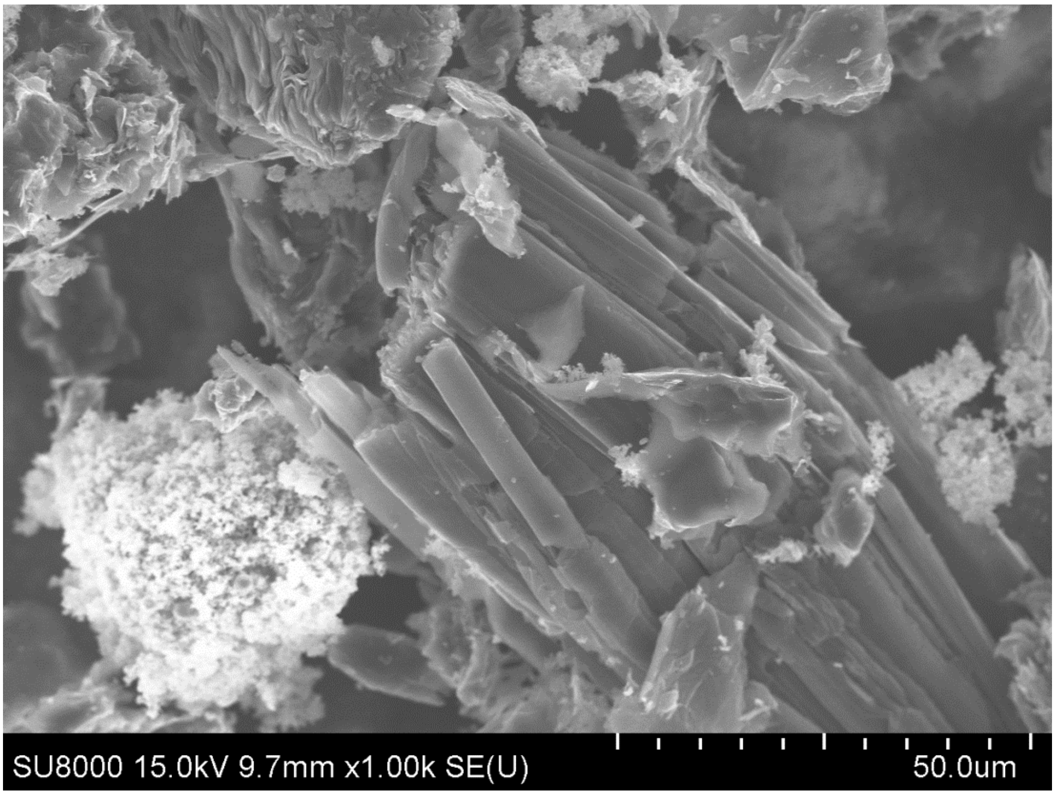

Figure S142. SEM image of graphite powder with TiCN sample after MW treatment.

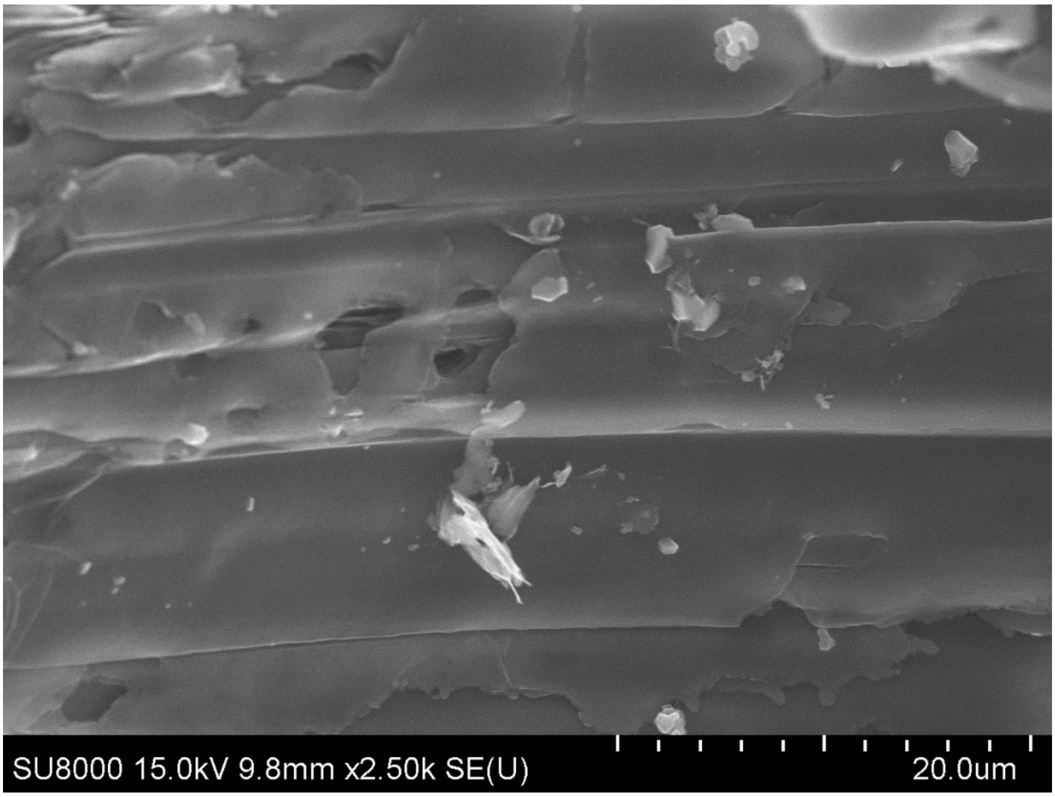

Figure S143. SEM image of graphite powder with TiCN sample after MW treatment.

SEM images of changes in graphite morphology in the presence of TiC after MW treatment

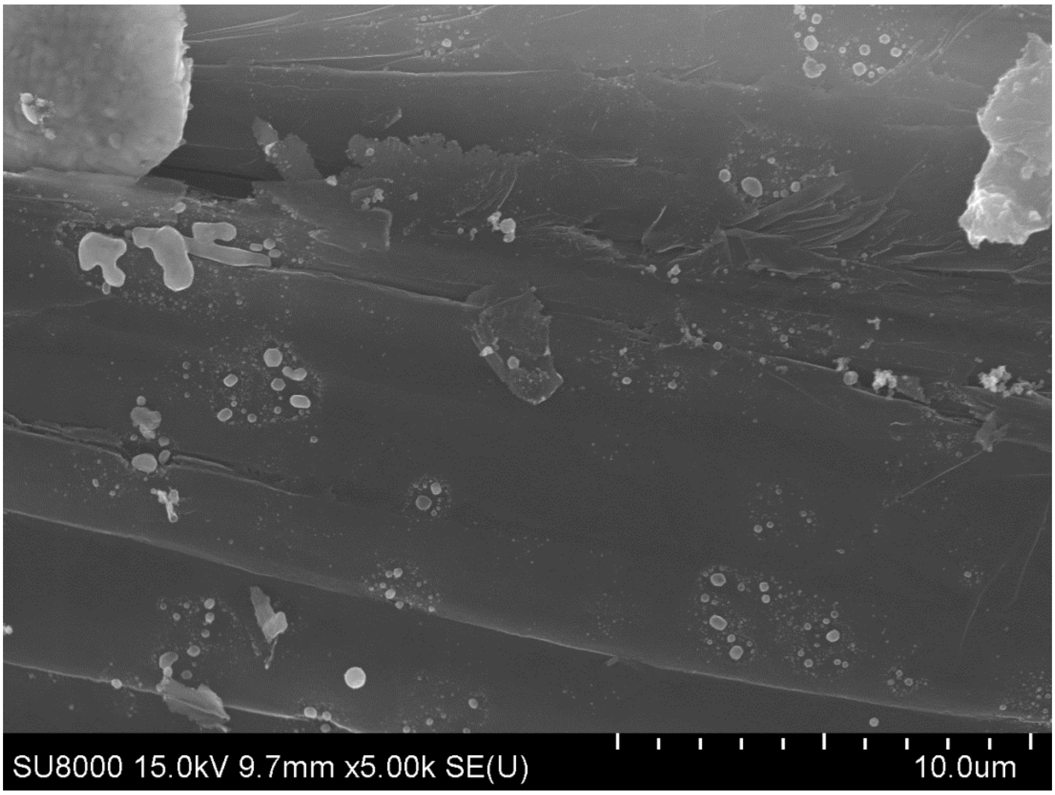

Figure S144. SEM image of graphite powder with TiC sample after MW treatment.

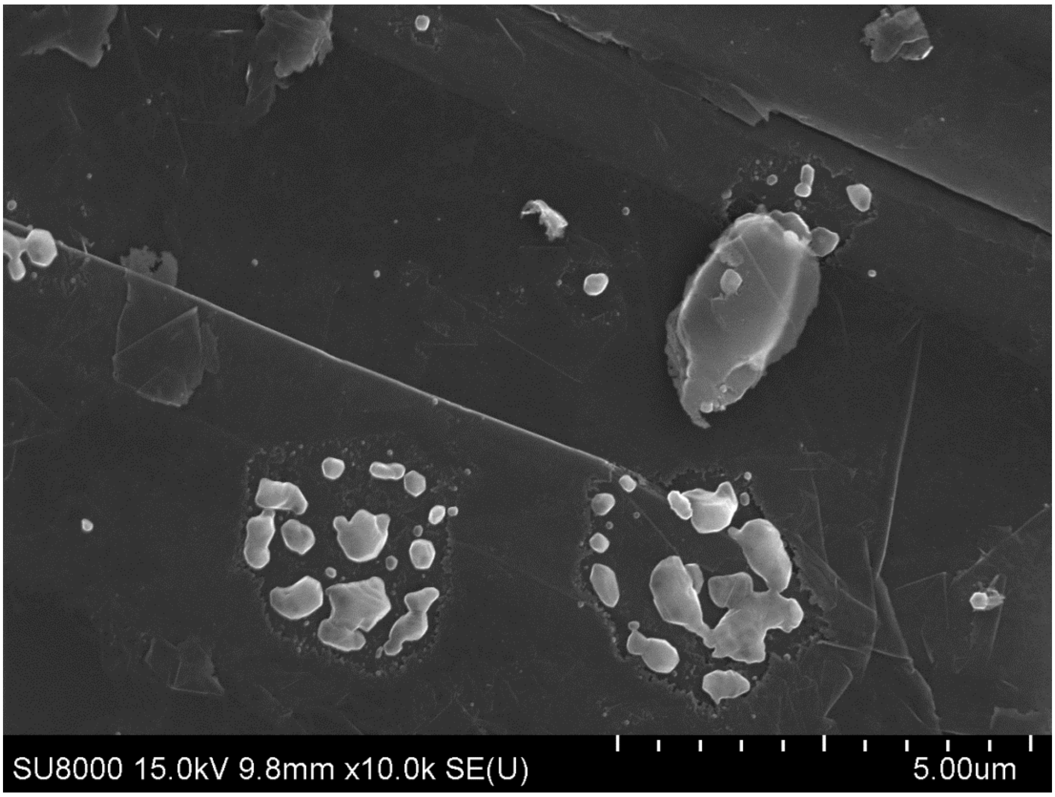

Figure S145. SEM image of graphite powder with TiC sample after MW treatment.

EDX data of graphite powder with TiC sample after MW treatment

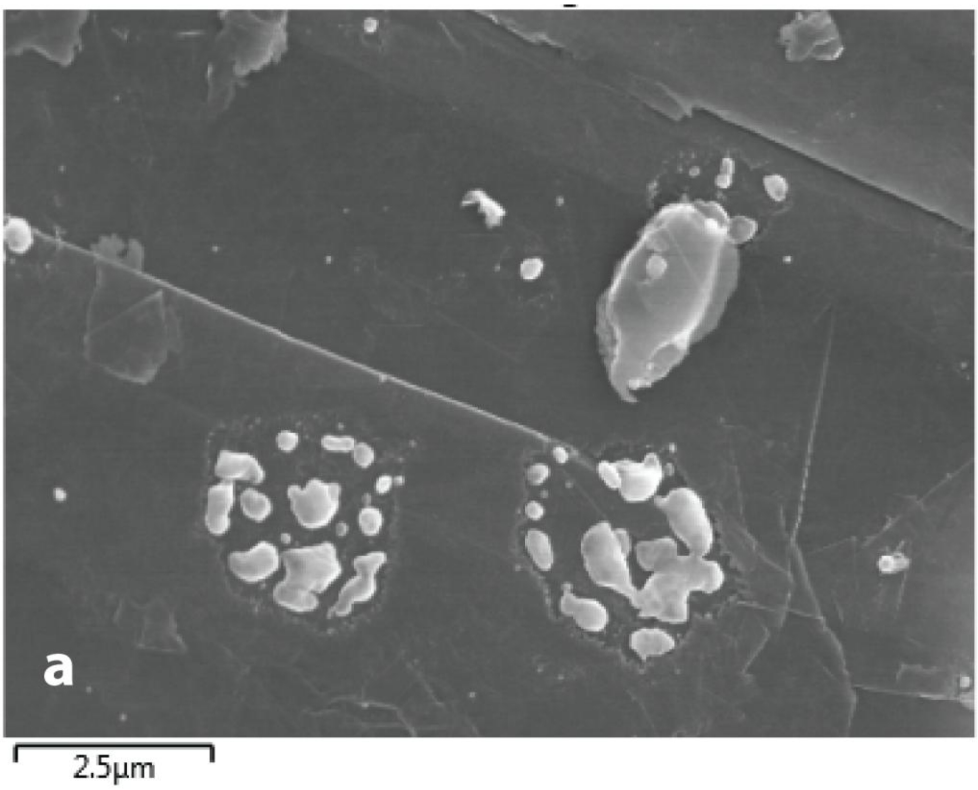

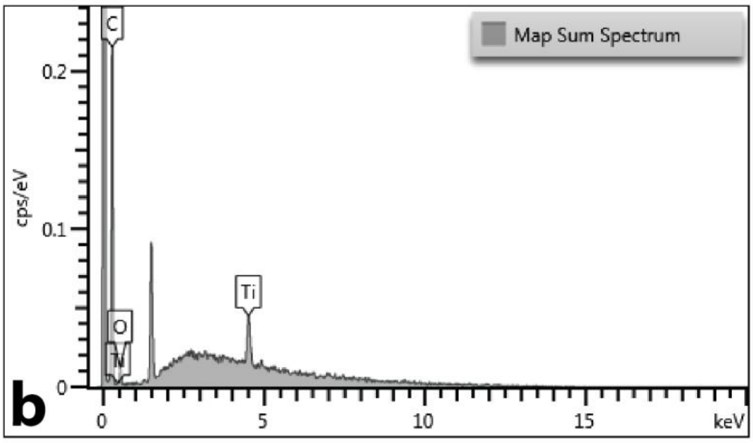

c

Element	Wt%	At%%
C	69.33	84.26
Ti	20.19	6.11
O	10.49	9.60
Total:	100	100

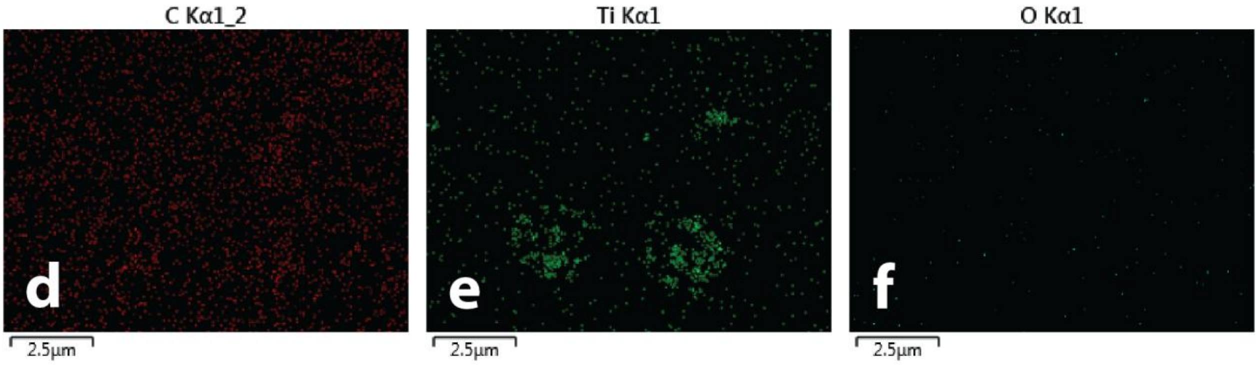

Figure S146. EDX study of graphite powder with TiC sample after MW treatment: SEM image (a); EDX spectrum of this area (b); element composition (c) and maps of carbon (d), titanium (e) and oxygen (f) distributions.

SEM images of changes in graphite morphology in the presence of SiC №1 after MW treatment

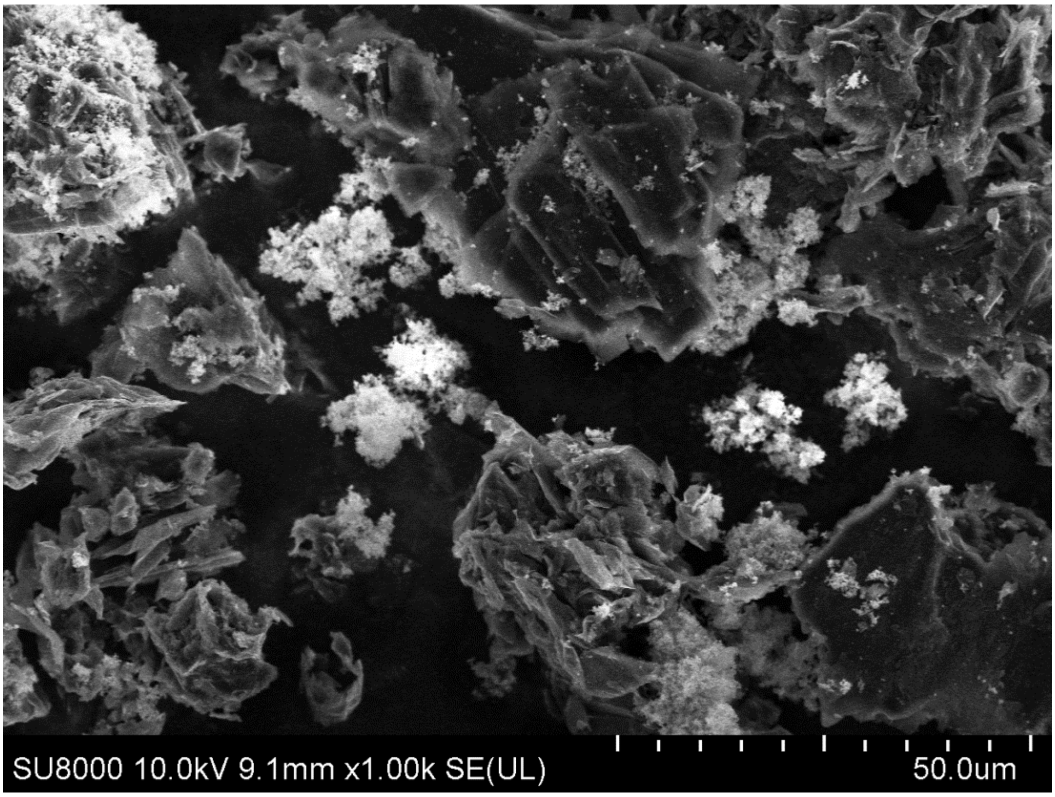

Figure S147. SEM image of graphite powder with SiC №1 sample after MW treatment.

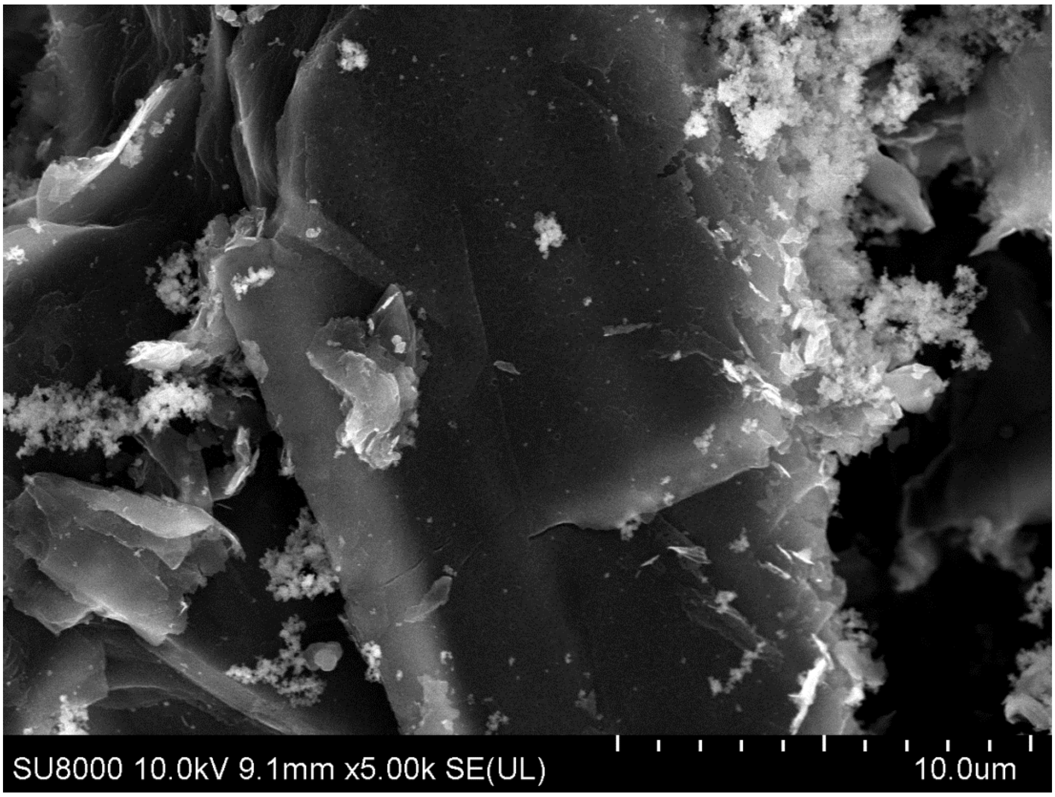

Figure S148. SEM image of graphite powder with SiC №1 sample after MW treatment.

SEM images of changes in graphite morphology in the presence of SiC №2 after MW treatment

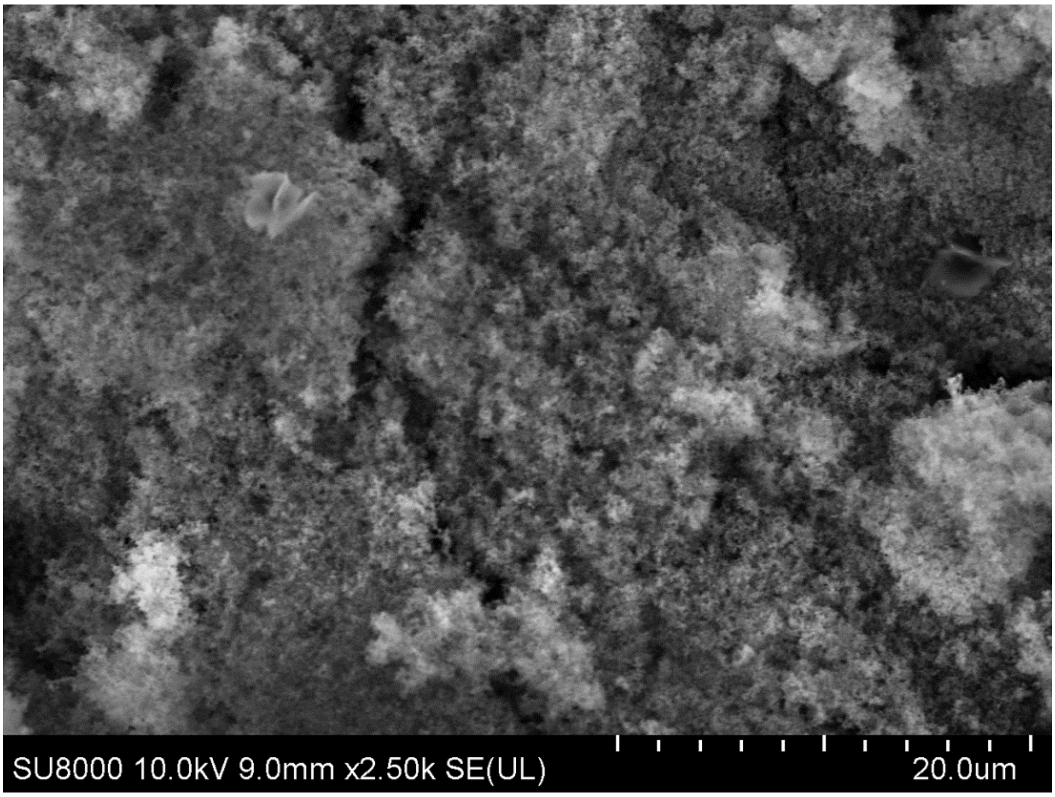

Figure S149. SEM image of graphite powder with SiC №2 sample after MW treatment.

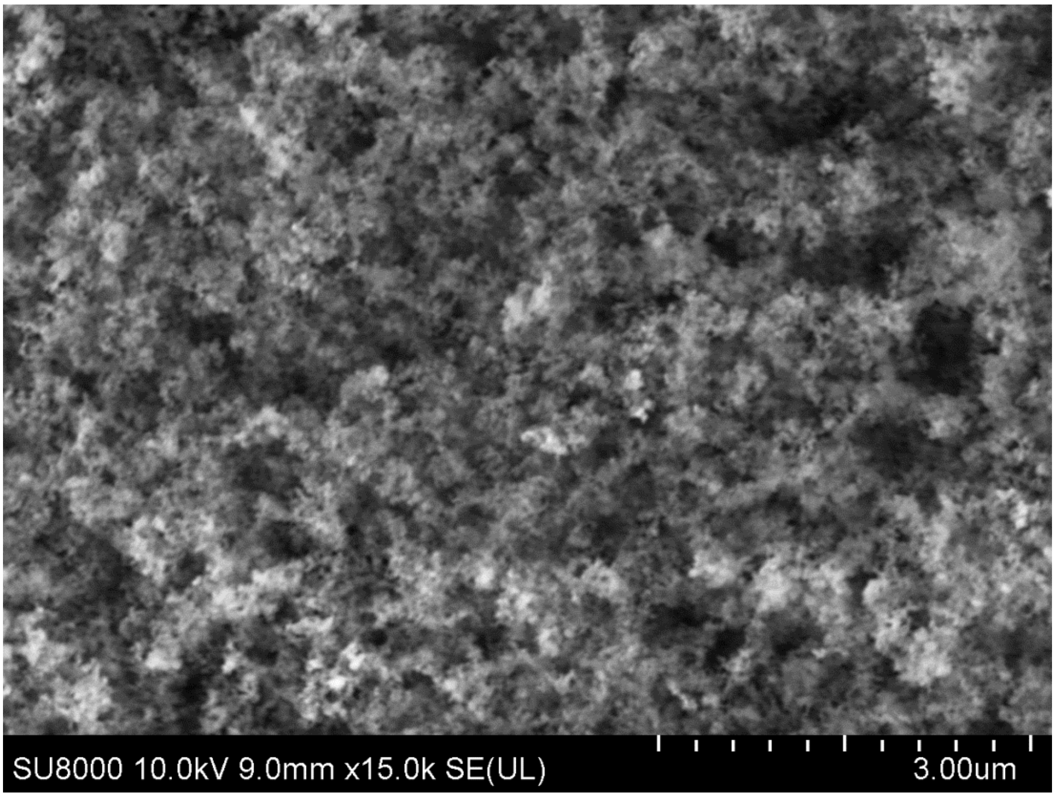

Figure S150. SEM image of graphite powder with SiC №2 sample after MW treatment.

SEM images of changes in graphite morphology in the presence of MoS_2 after MW treatment

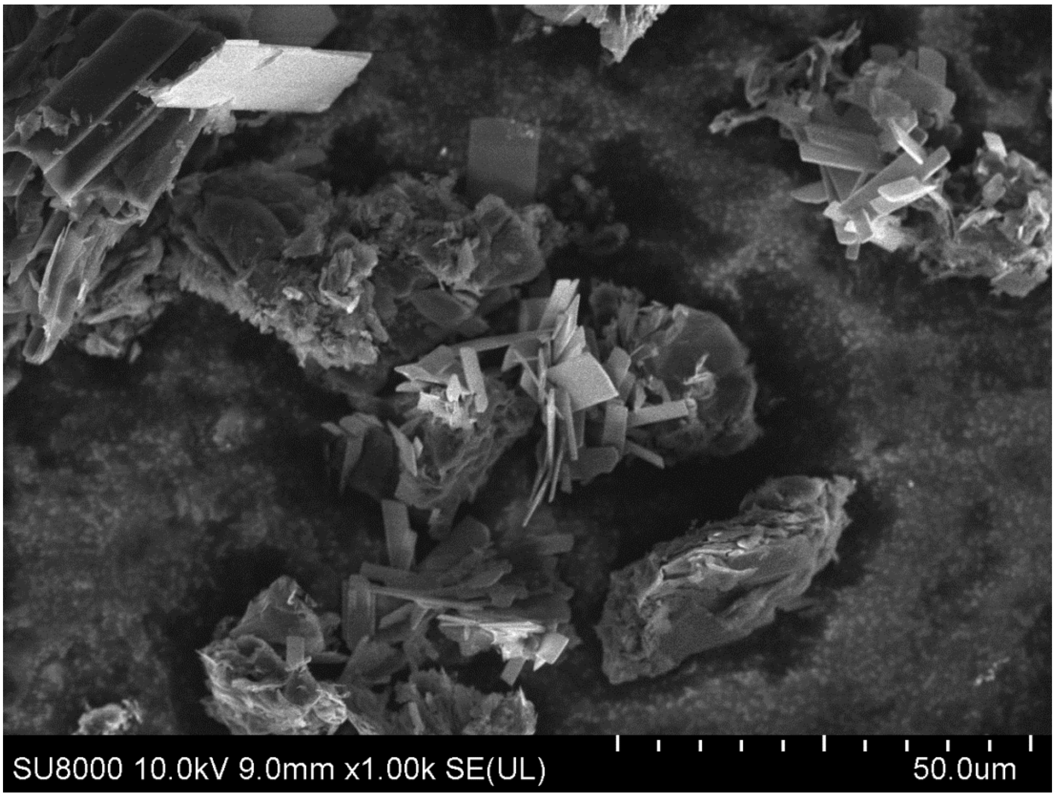

Figure S151. SEM image of graphite powder with MoS_2 sample after MW treatment.

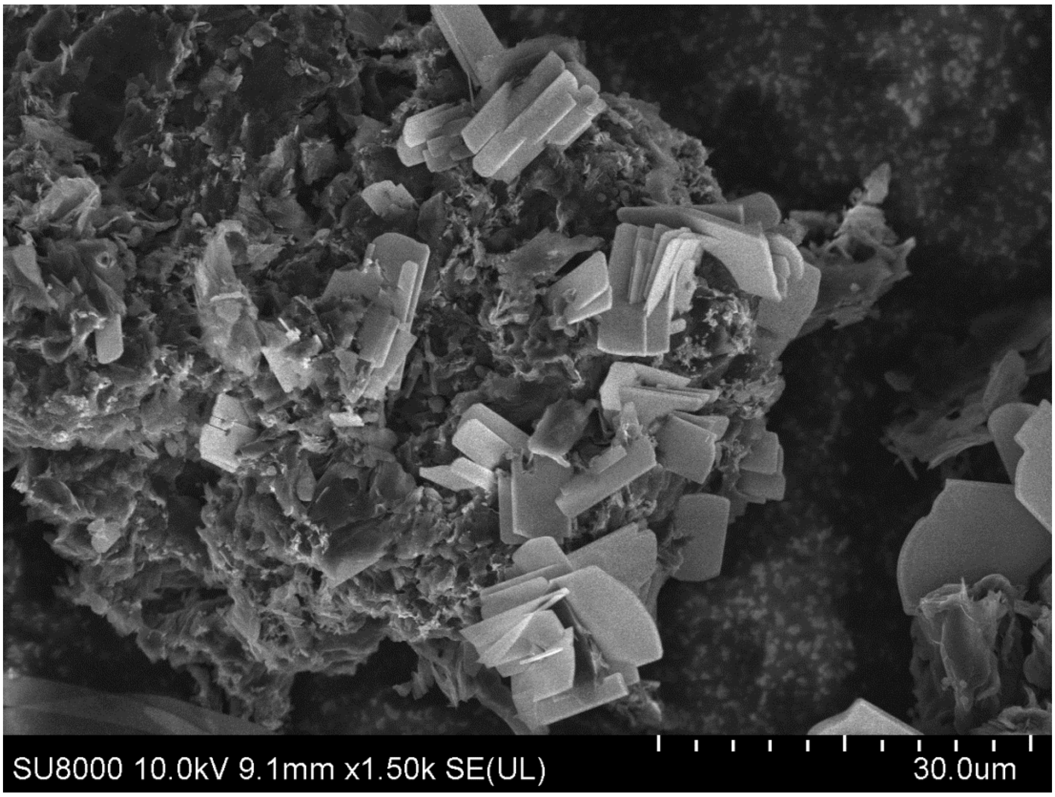

Figure S152. SEM image of graphite powder with MoS_2 sample after MW treatment.

EDX data of graphite powder with MoS_2 sample after MW treatment

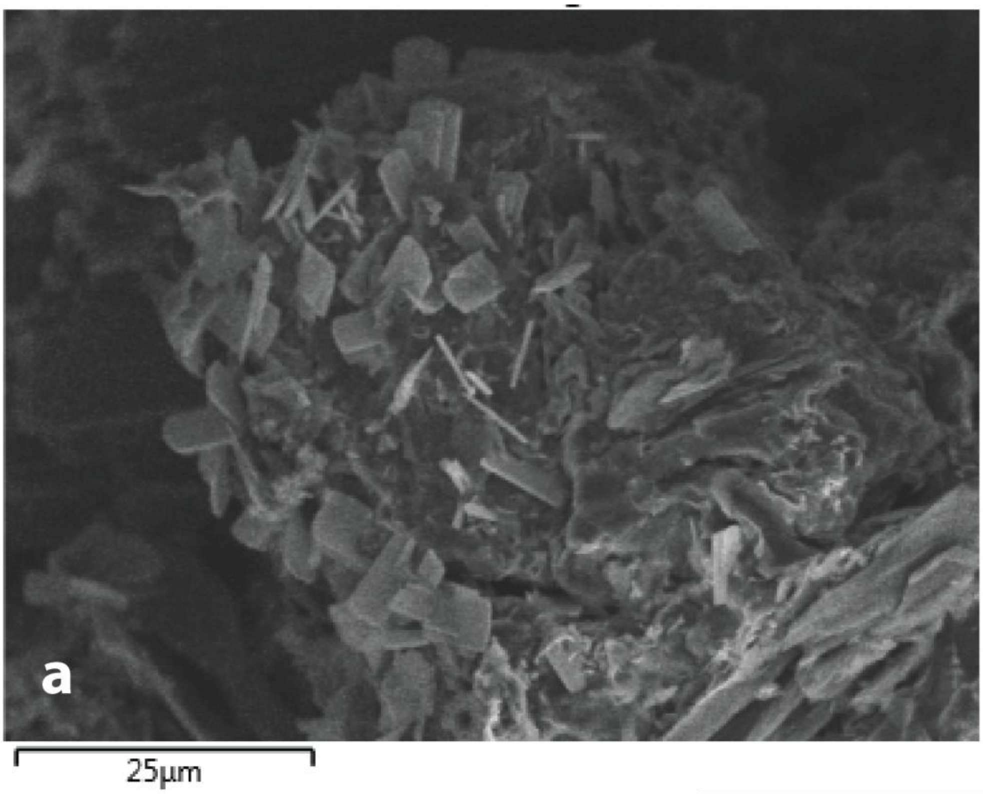

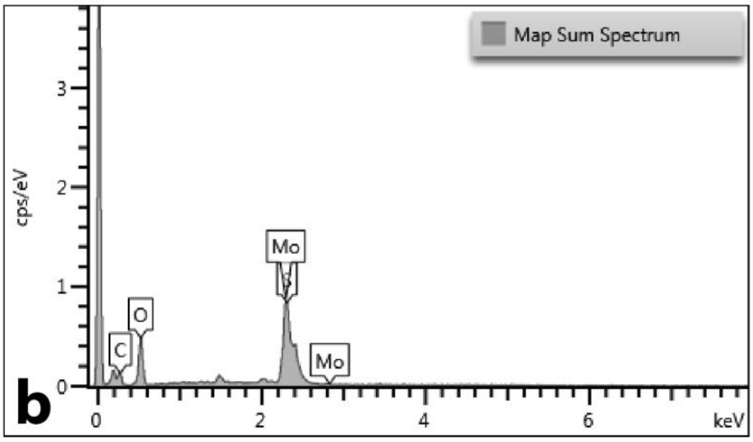

c

Element	Wt%	At%
C	82.34	93.08
Mo	11.35	1.63
O	6.31	5.30
Total:	100	100

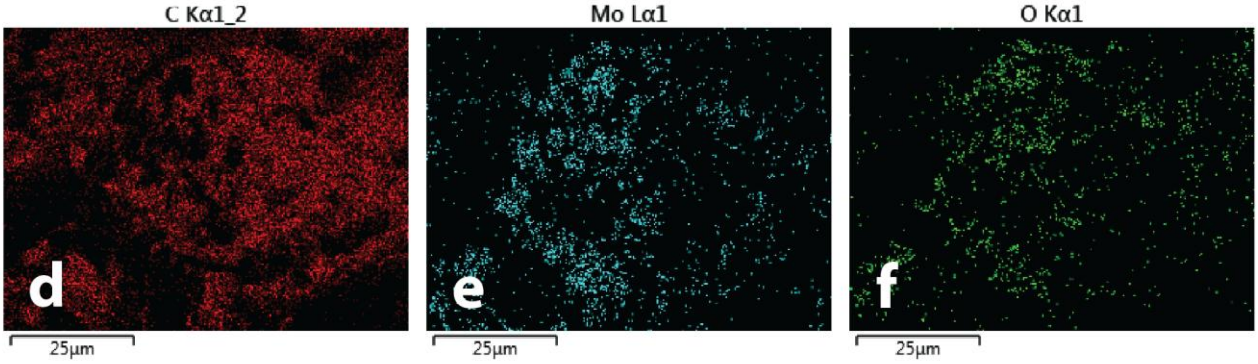

Figure S153. EDX study of graphite powder with MoS_2 sample after MW treatment: SEM image (a); EDX spectrum of this area (b); element composition (c) and maps of carbon (d), molybdenum (e) and oxygen (f) distributions.

SEM images of changes in graphite morphology in the presence of Al-B after MW treatment

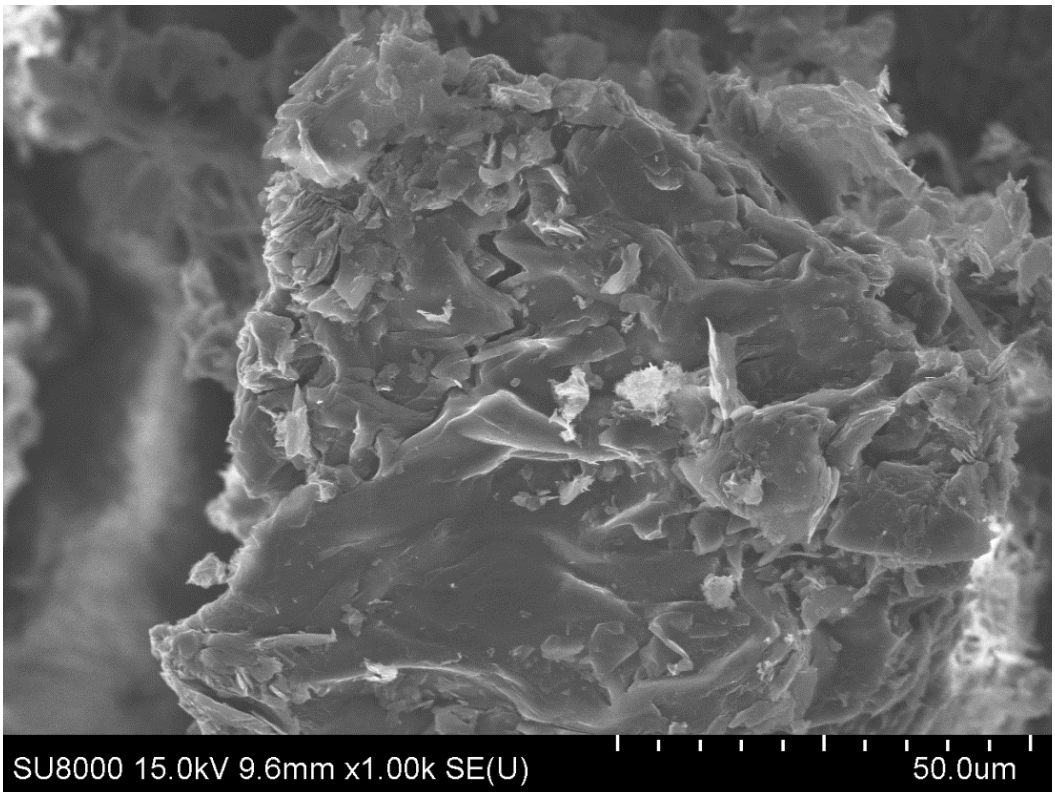

Figure S154. SEM image of graphite powder with Al-B sample after MW treatment.

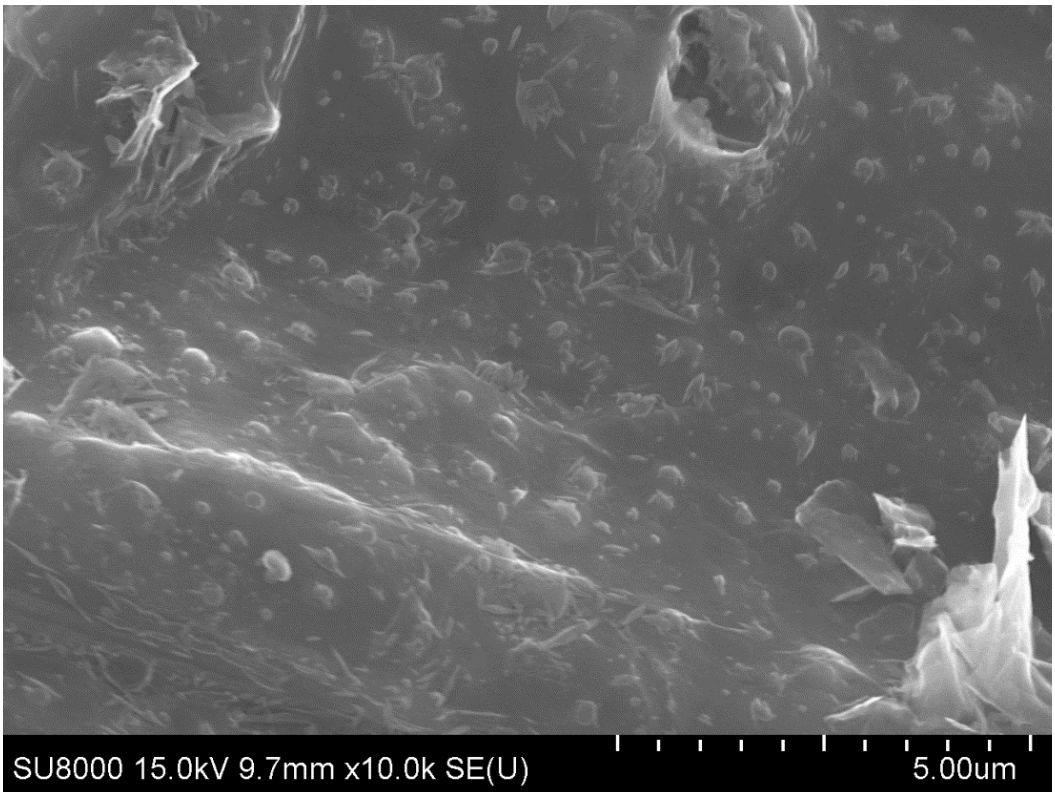

Figure S155. SEM image of graphite powder with Al-B sample after MW treatment.

SEM images of changes in graphite morphology in the presence of W-C after MW treatment

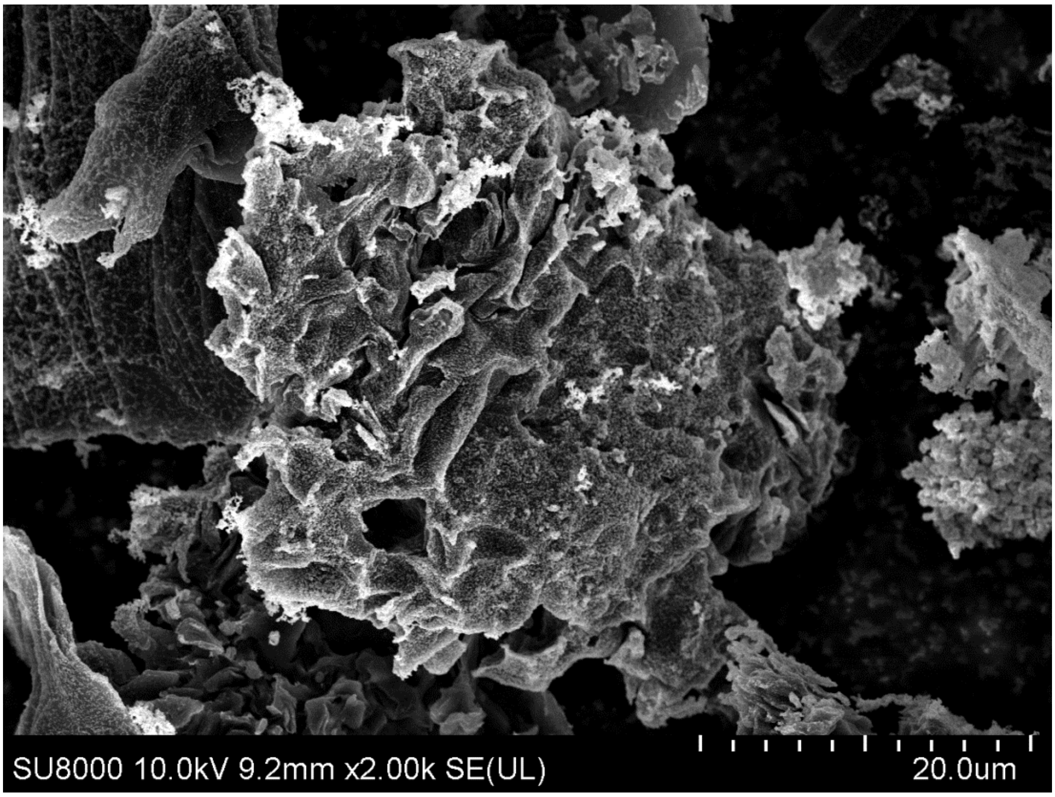

Figure S156. SEM image of graphite powder with W-C sample after MW treatment.

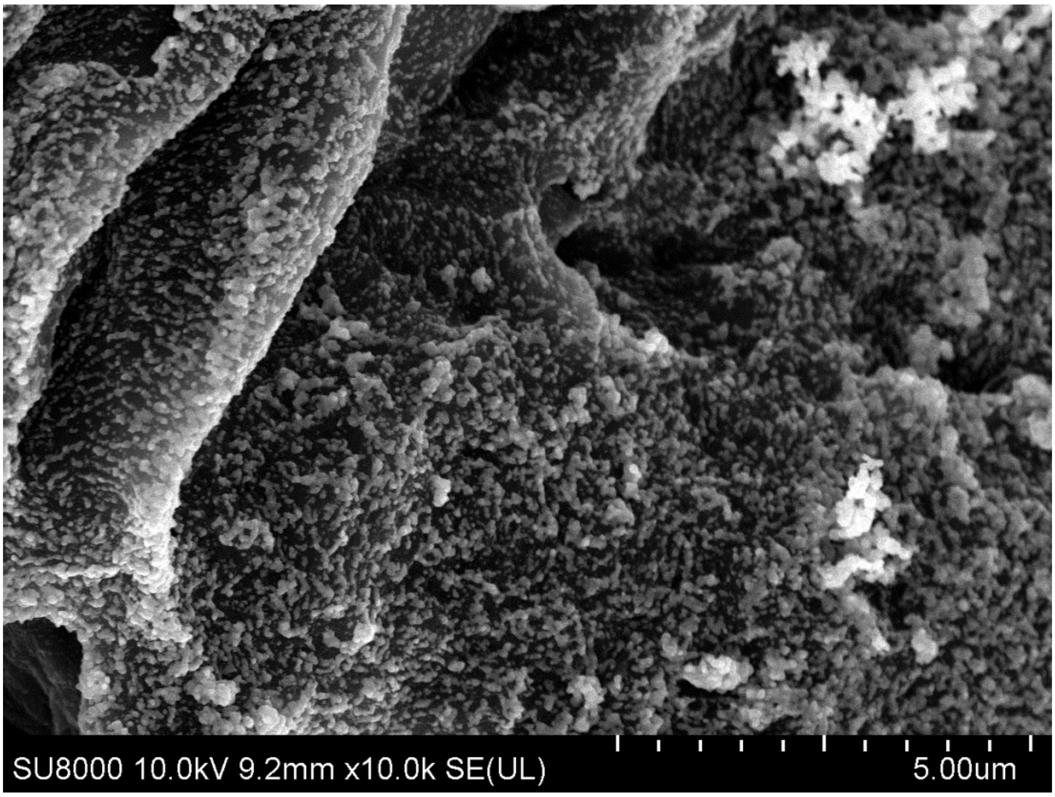

Figure S157. SEM image of graphite powder with W-C sample after MW treatment.

SEM images of changes in graphite morphology in the presence of V-C after MW treatment

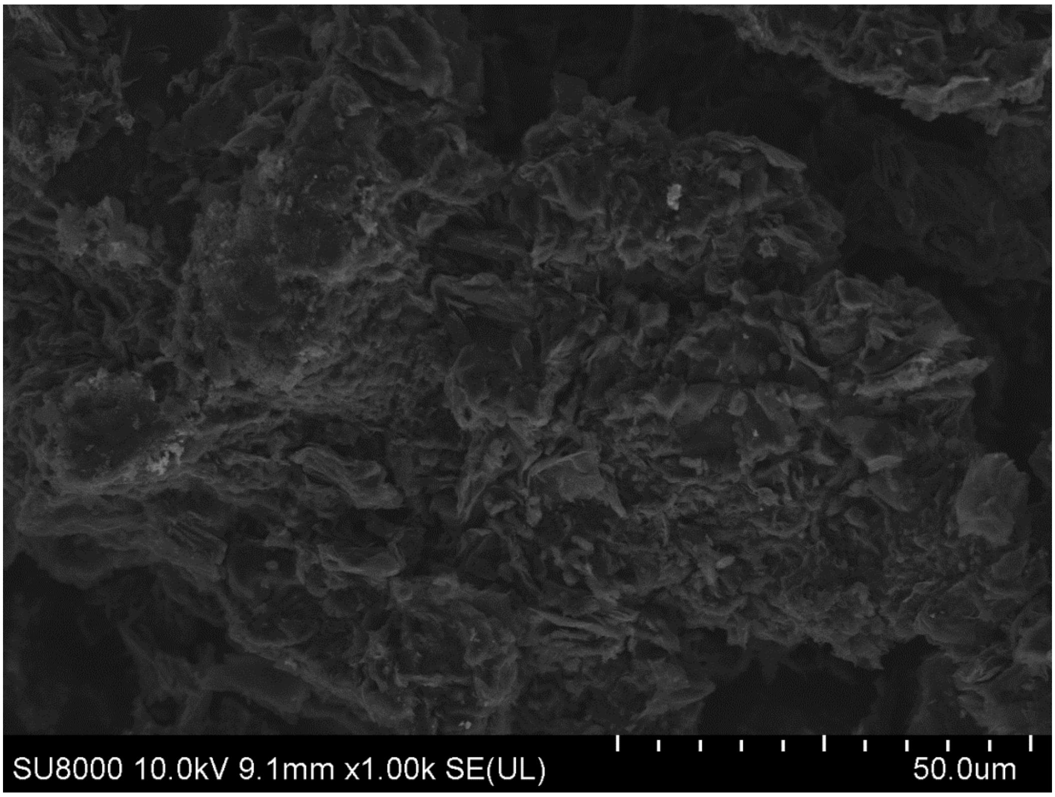

Figure S158. SEM image of graphite powder with V-C sample after MW treatment.

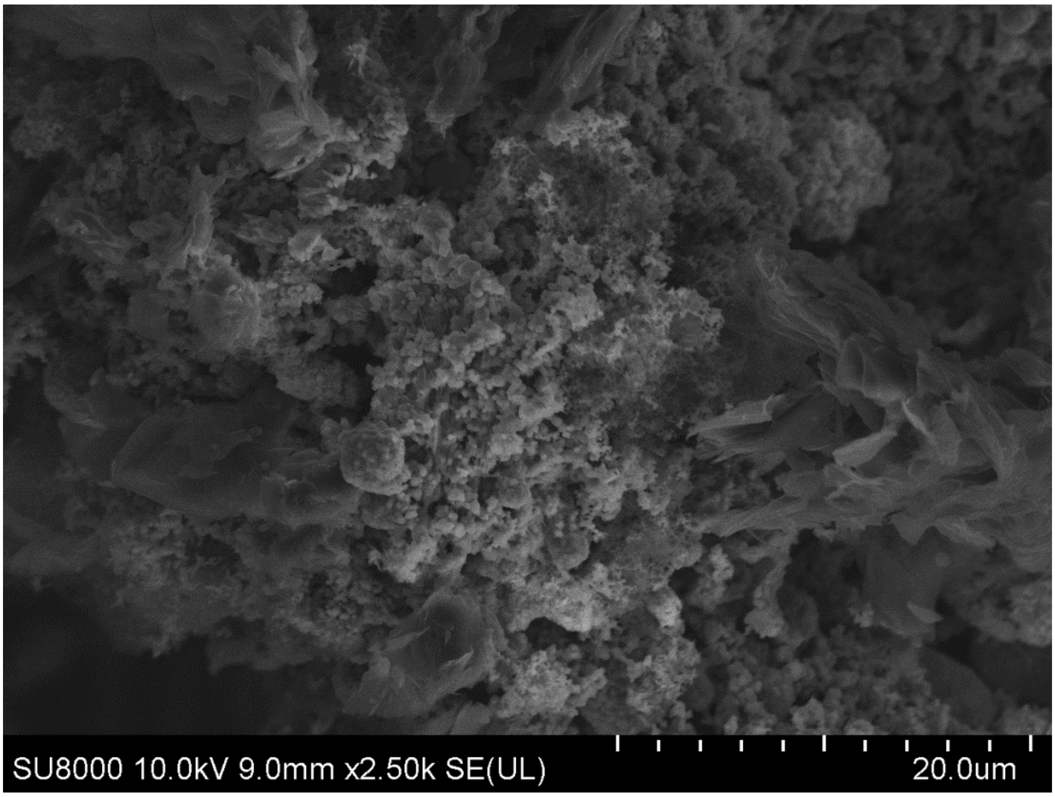

Figure S159. SEM image of graphite powder with V-C sample after MW treatment.

SEM images of changes in graphite morphology in the presence of Cr-C after MW treatment

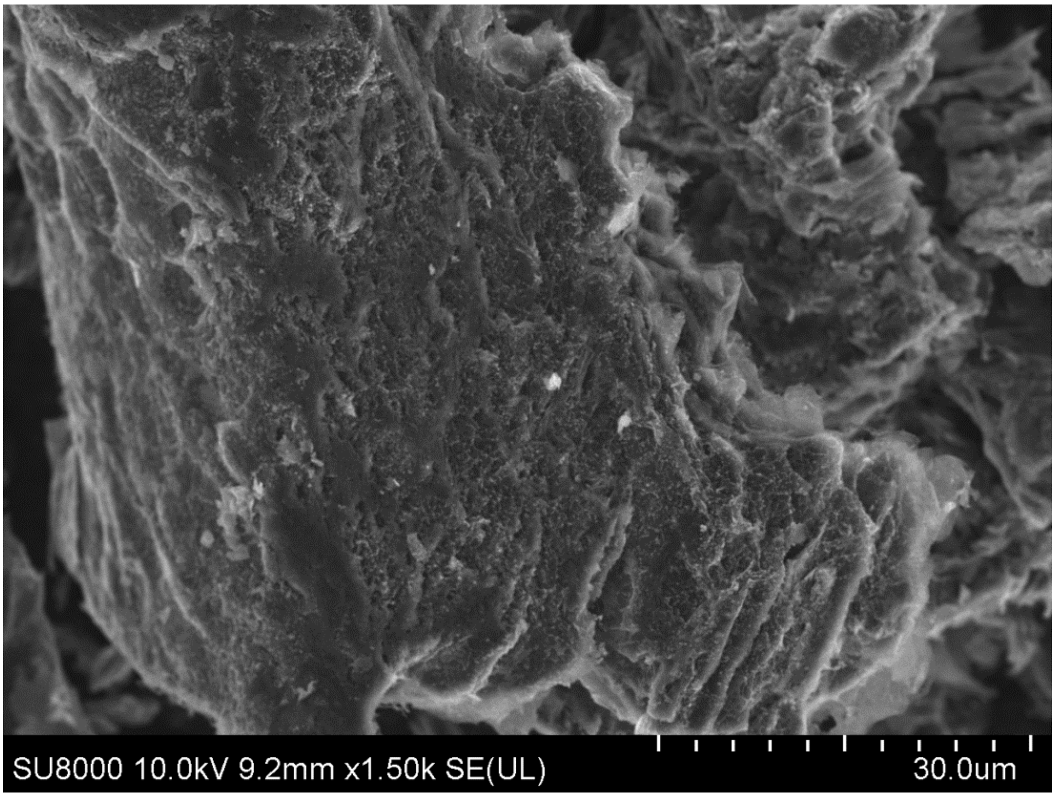

Figure S160. SEM image of graphite powder with Cr-C sample after MW treatment.

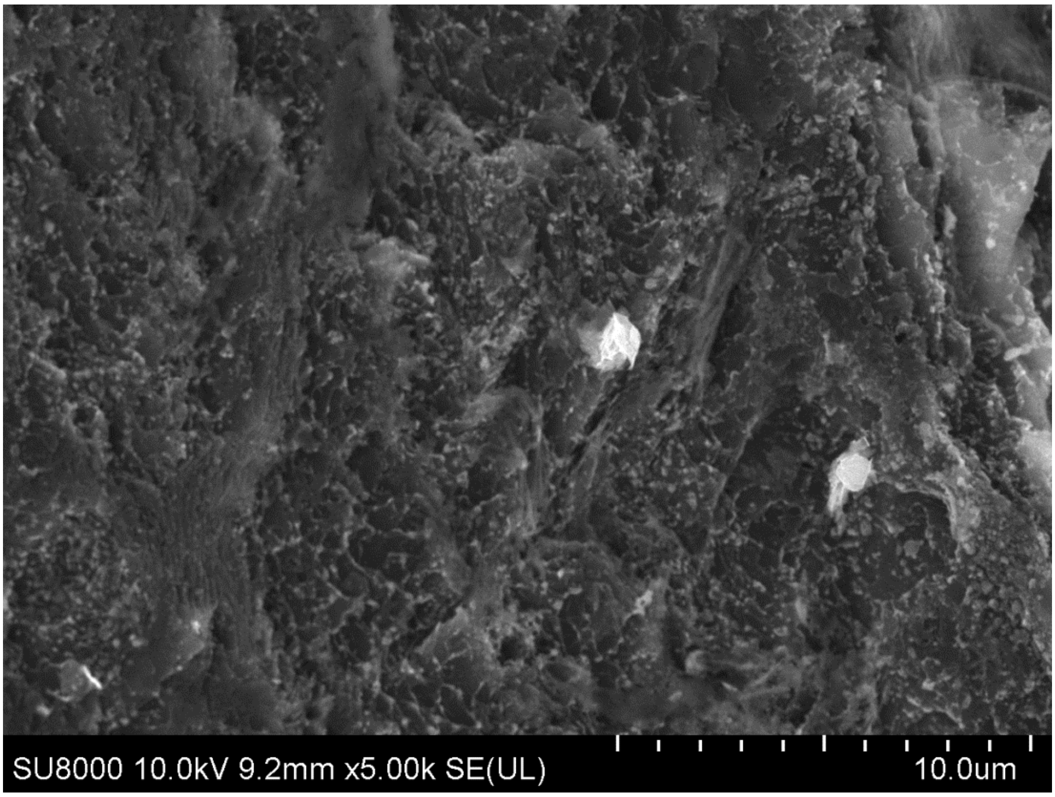

Figure S161. SEM image of graphite powder with Cr-C sample after MW treatment.

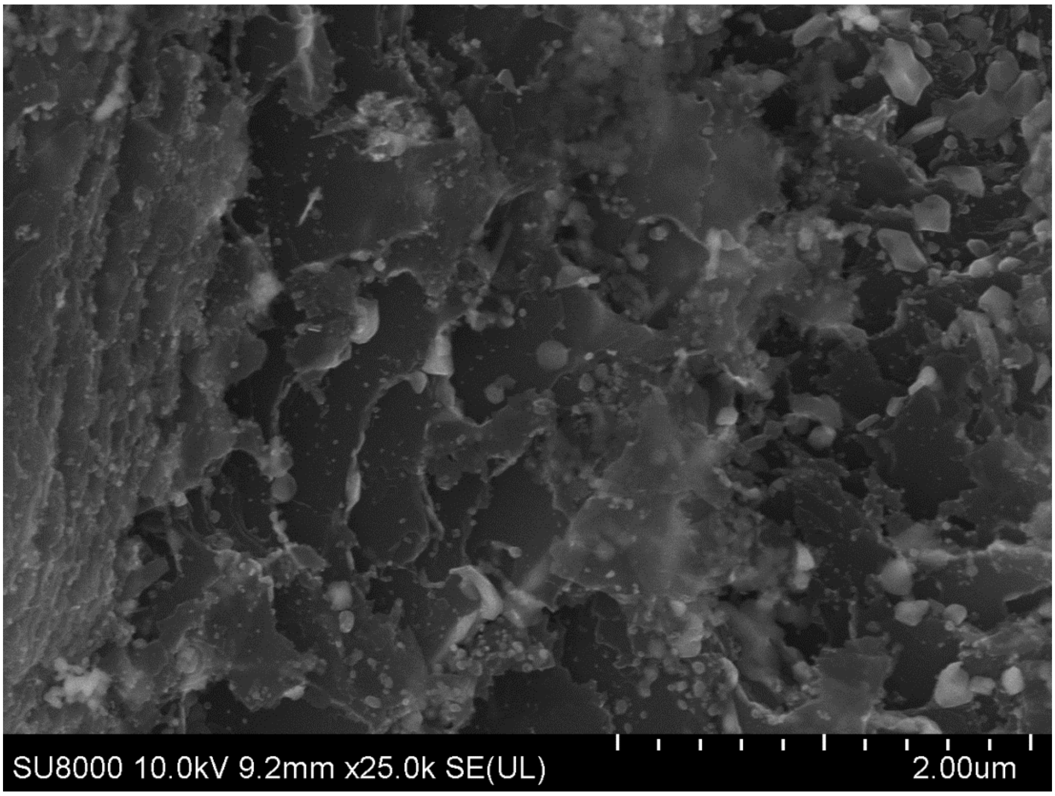

Figure S162. SEM image of graphite powder with Cr-C sample after MW treatment.

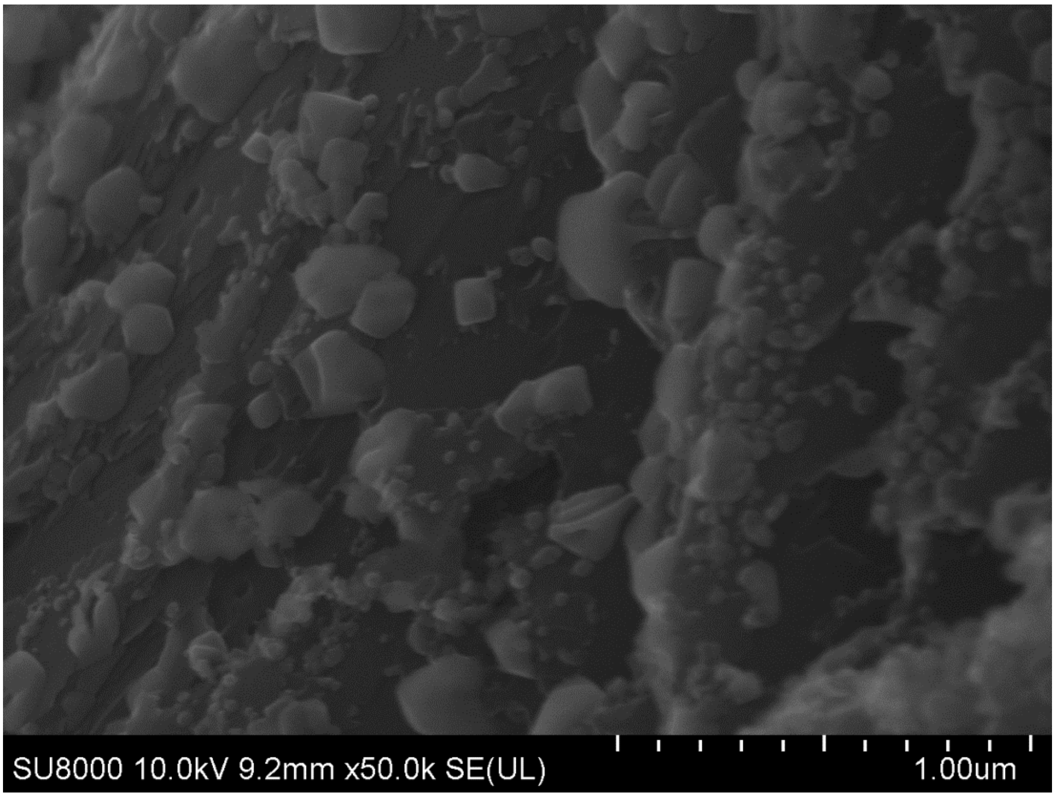

Figure S163. SEM image of graphite powder with Cr-C sample after MW treatment.

EDX data of graphite powder with Cr-C sample after MW treatment

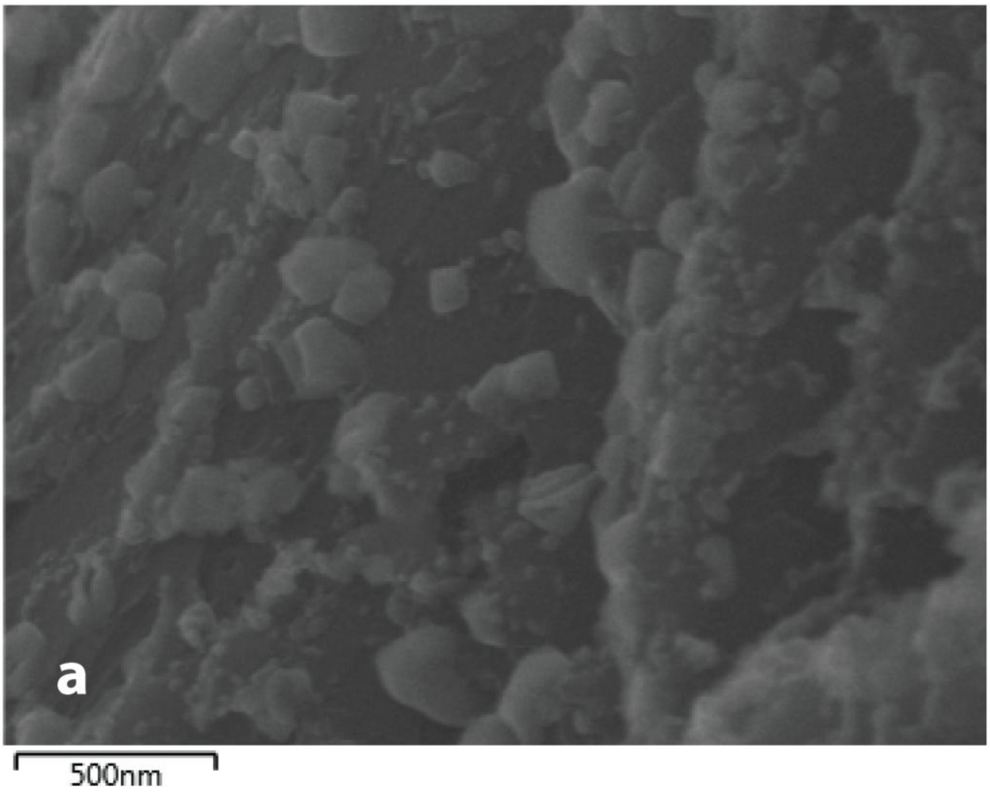

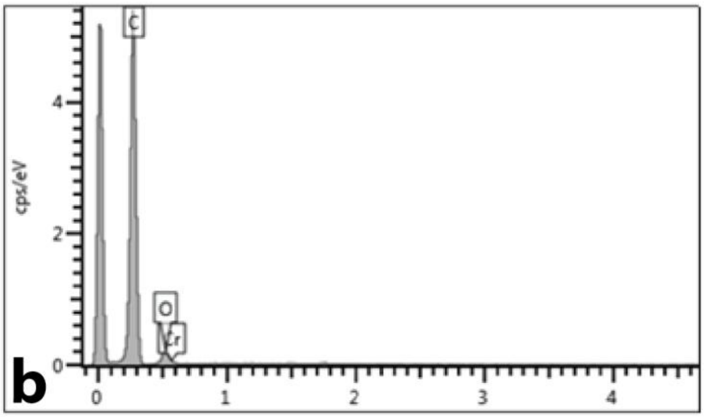

c

Element	Wt%	At%
C	87.04	92.95
Cr	6.13	1.54
O	6.83	5.51
Total:	100	100

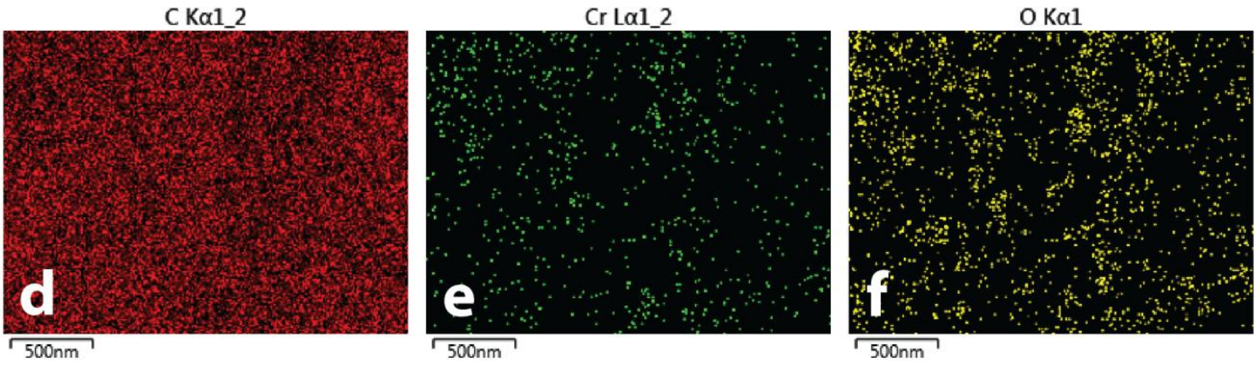

Figure S164. EDX study of graphite powder with Cr-C sample after MW treatment: SEM image (a); EDX spectrum of this area (b); element composition (c) and maps of carbon (d), chromium (e) and oxygen (f) distributions.

SEM images of changes in graphite morphology in the presence of W-V-C after MW treatment

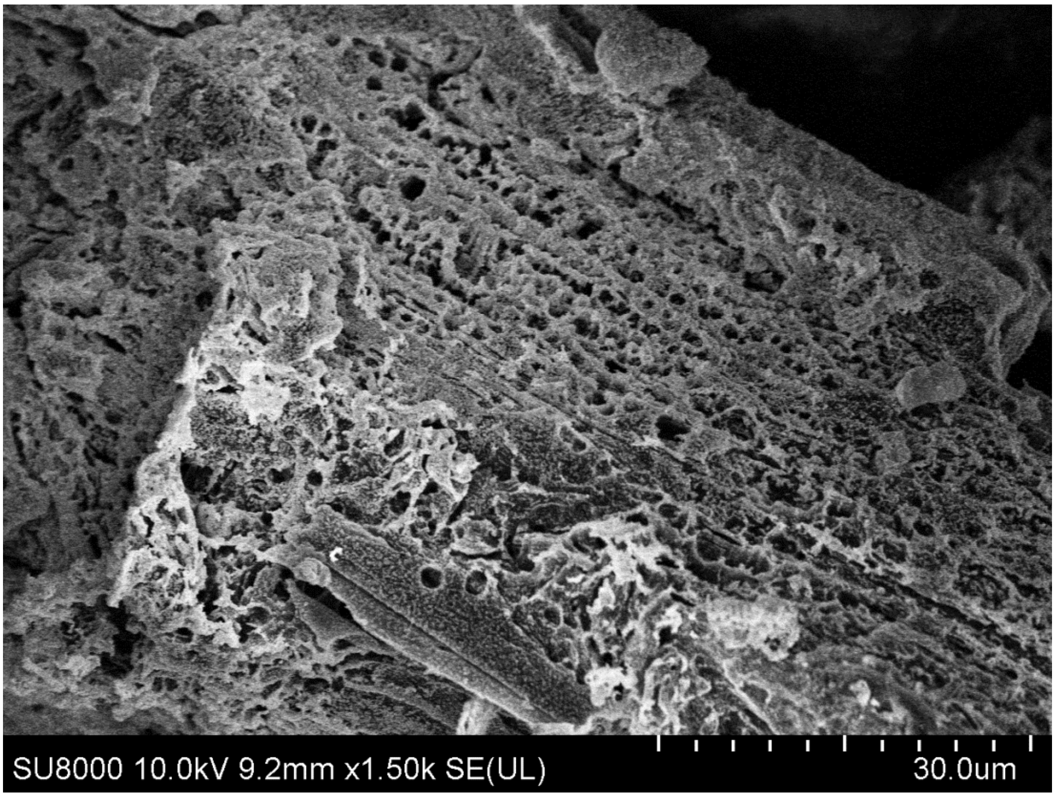

Figure S165. SEM image of graphite powder with W-V-C sample after MW treatment.

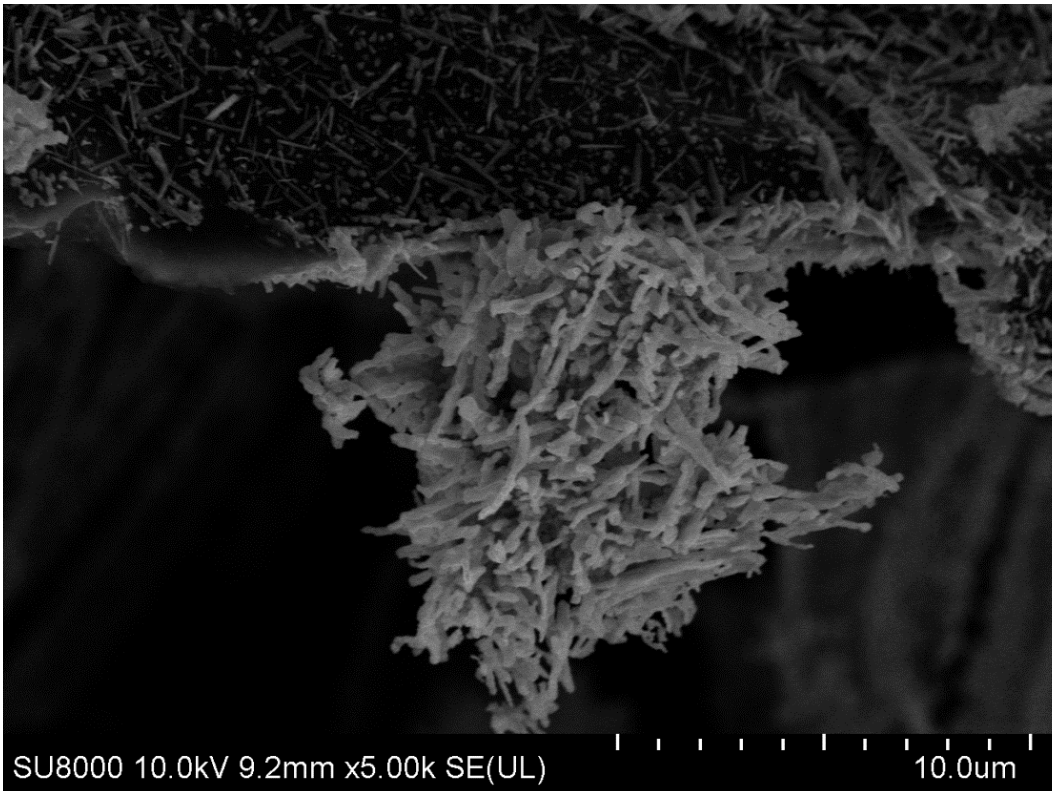

Figure S166. SEM image of graphite powder with W-V-C sample after MW treatment.

SEM images of changes in graphite morphology in the presence of Al_2O_3 after MW treatment

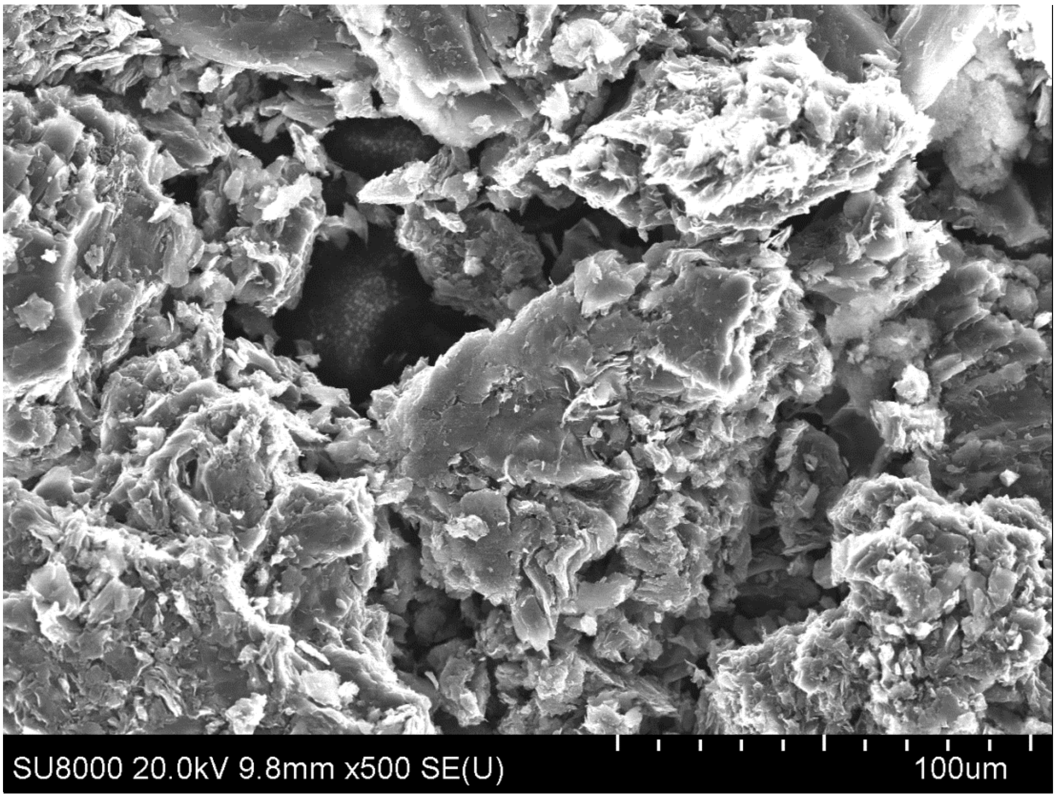

Figure S167. SEM image of graphite powder with Al_2O_3 sample after MW treatment.

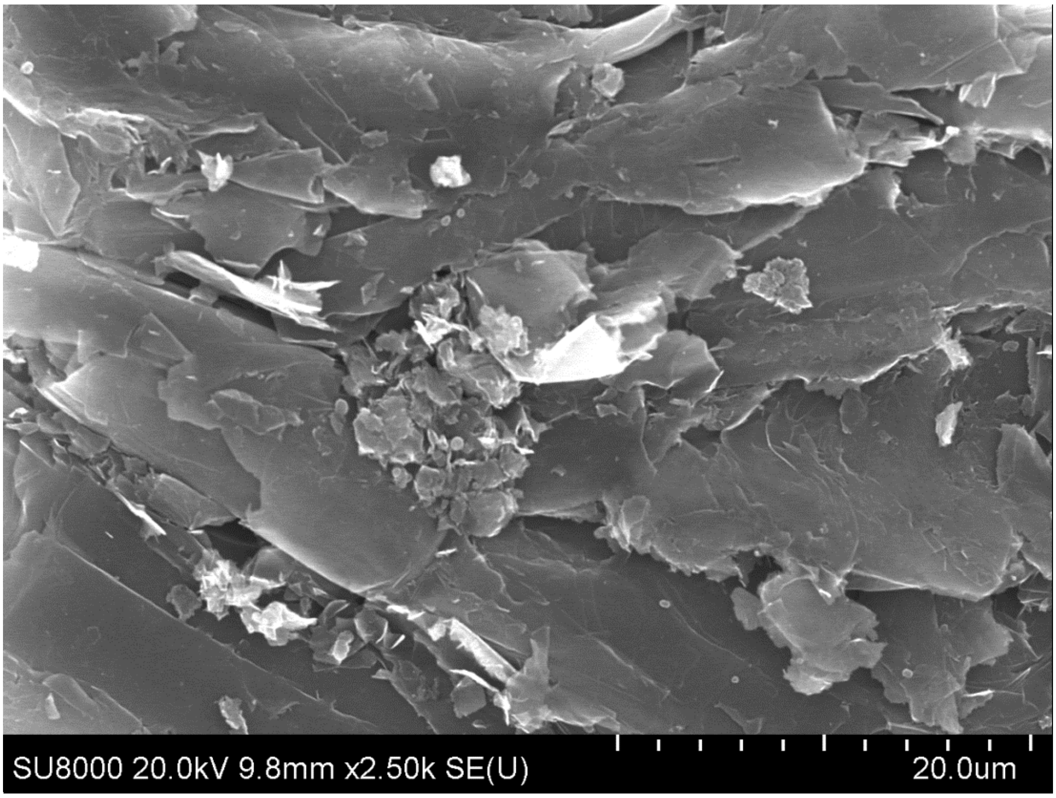

Figure S168. SEM image of graphite powder with Al_2O_3 sample after MW treatment.

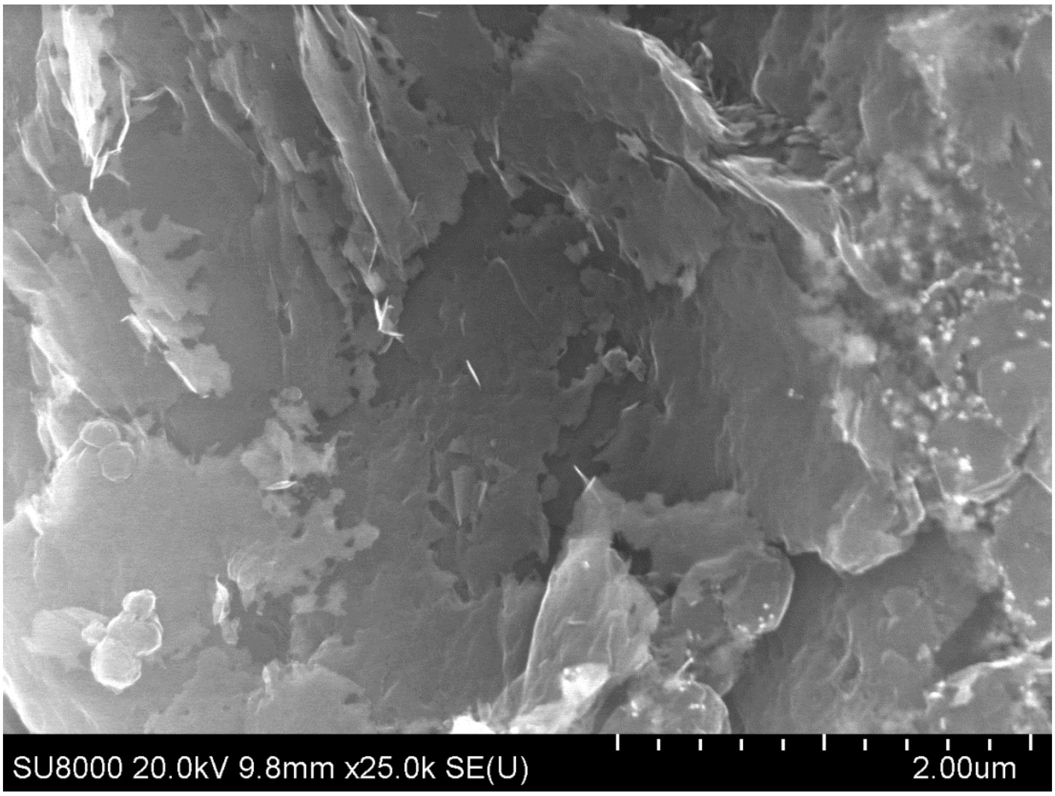

Figure S169. SEM image of graphite powder with Al_2O_3 sample after MW treatment.

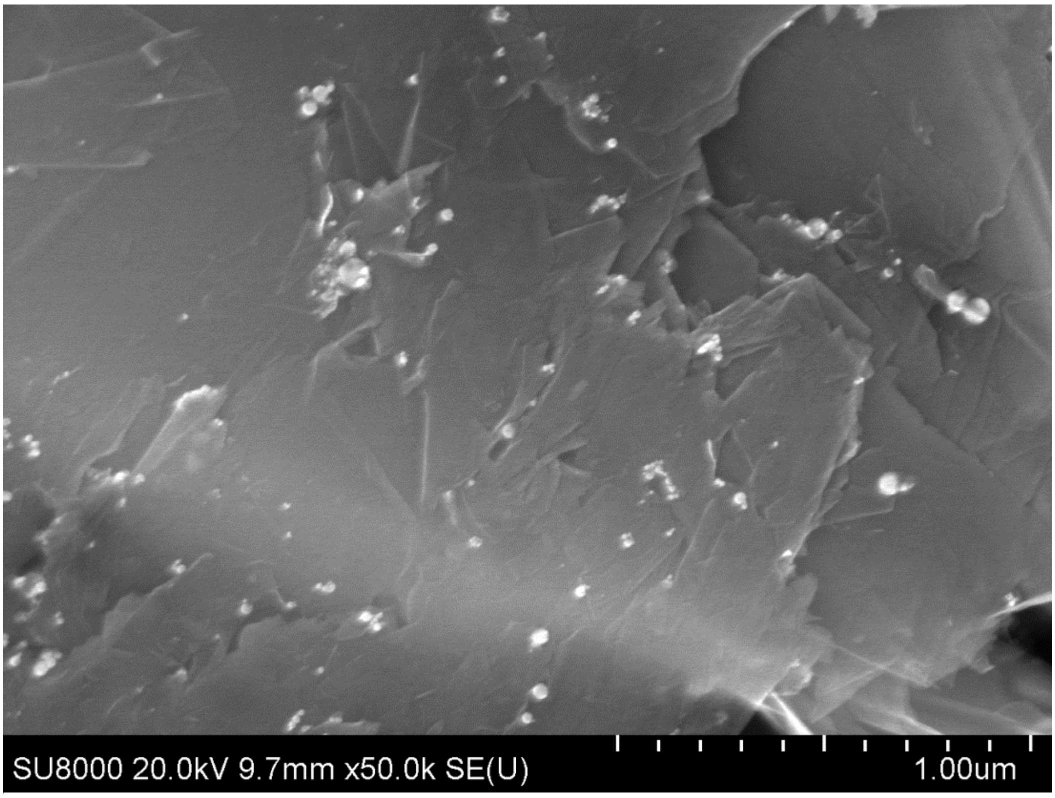

Figure S170. SEM image of graphite powder with Al_2O_3 sample after MW treatment.

SEM images of changes in graphite morphology in the presence of SiO_2 after MW treatment

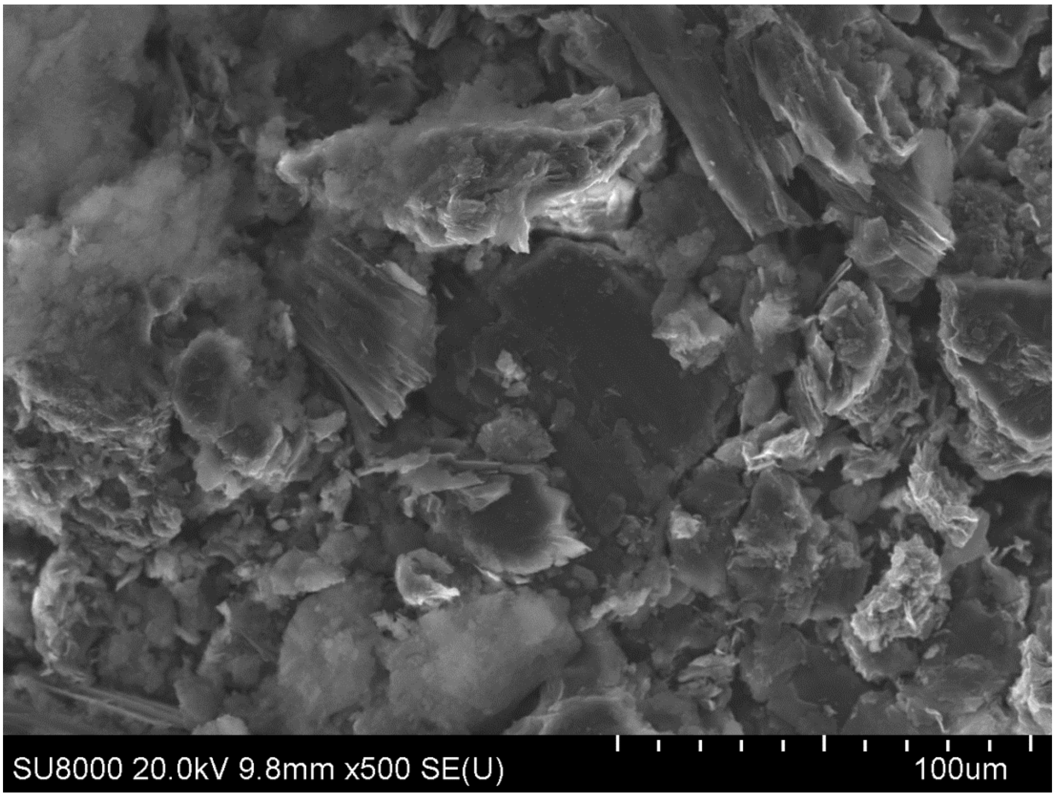

Figure S171. SEM image of graphite powder with SiO_2 sample after MW treatment.

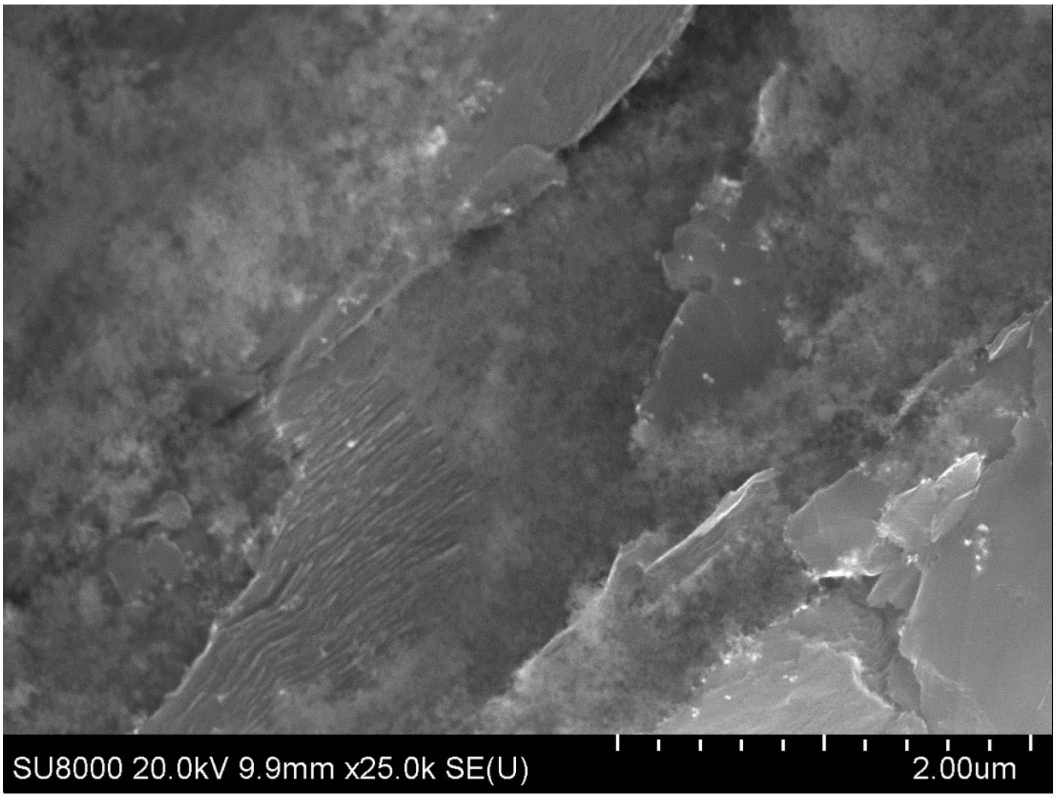

Figure S172. SEM image of graphite powder with SiO_2 sample after MW treatment.

SEM images of changes in graphite morphology in the presence of WO_3 after MW treatment

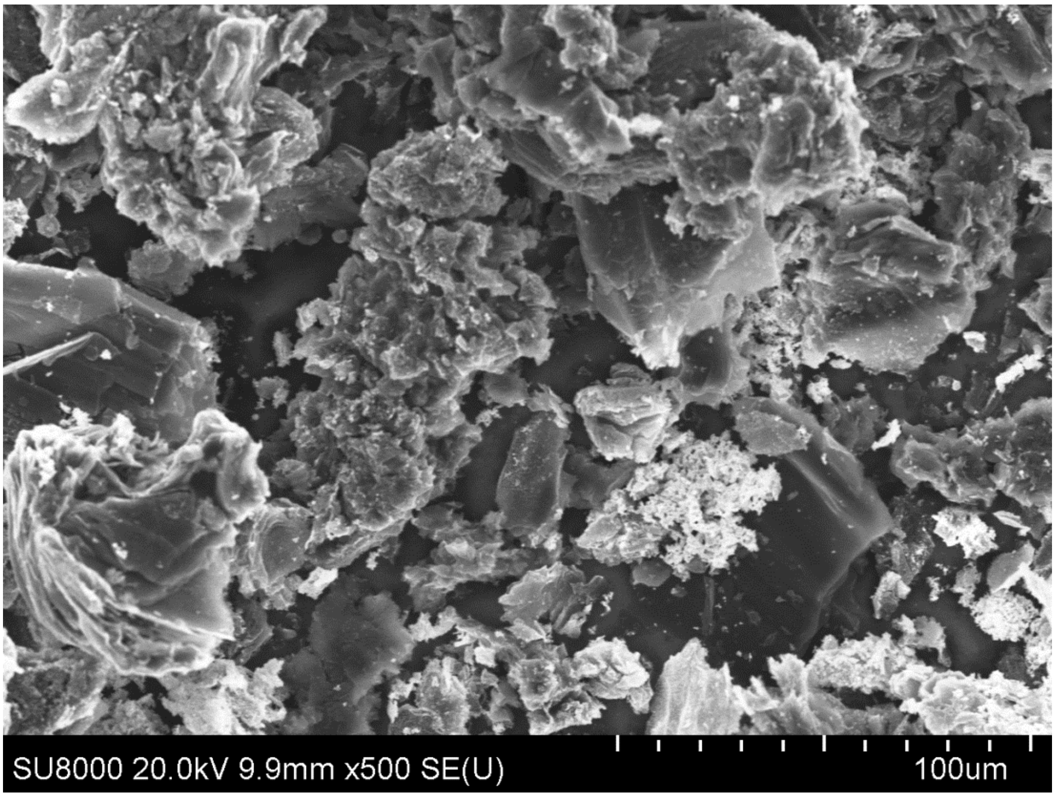

Figure S173. SEM image of graphite powder with WO_3 sample after MW treatment.

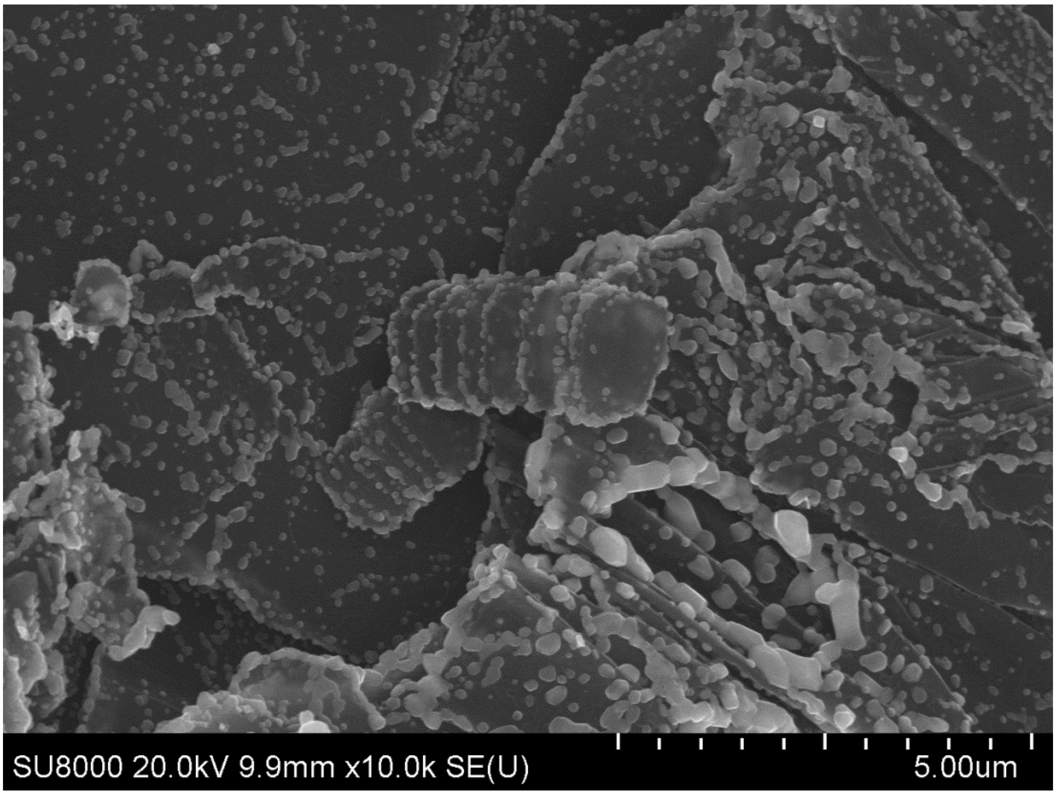

Figure S174. SEM image of graphite powder with WO_3 sample after MW treatment.

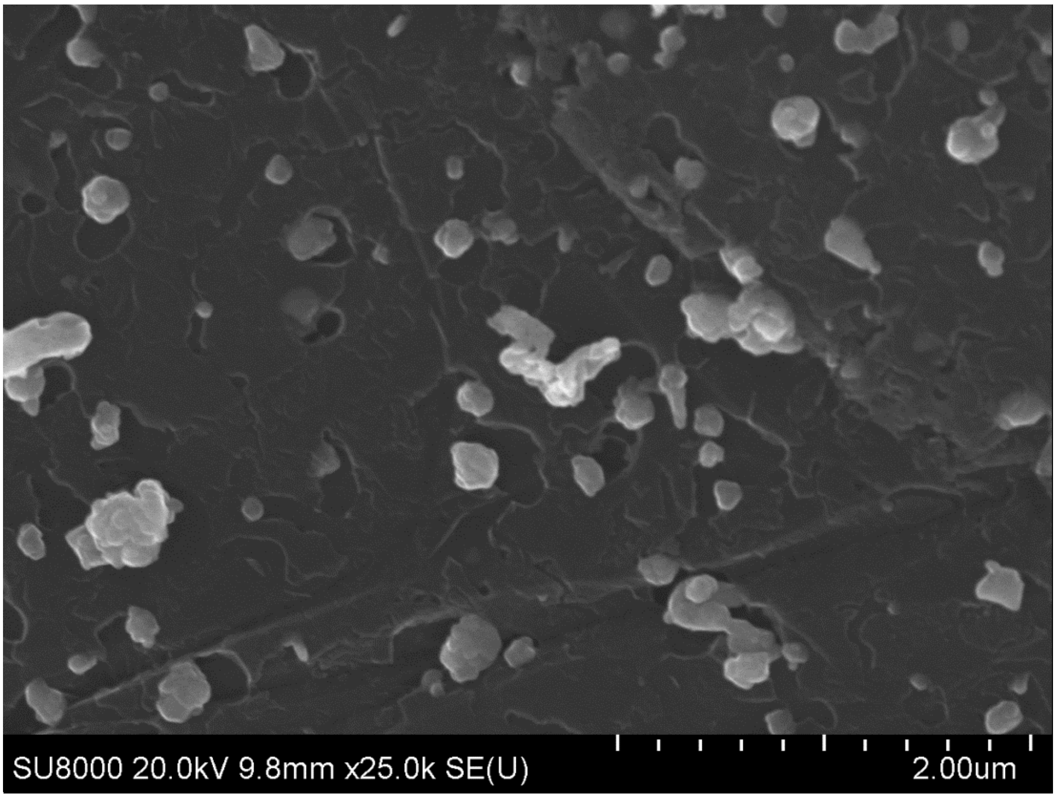

Figure S175. SEM image of graphite powder with WO_3 sample after MW treatment.

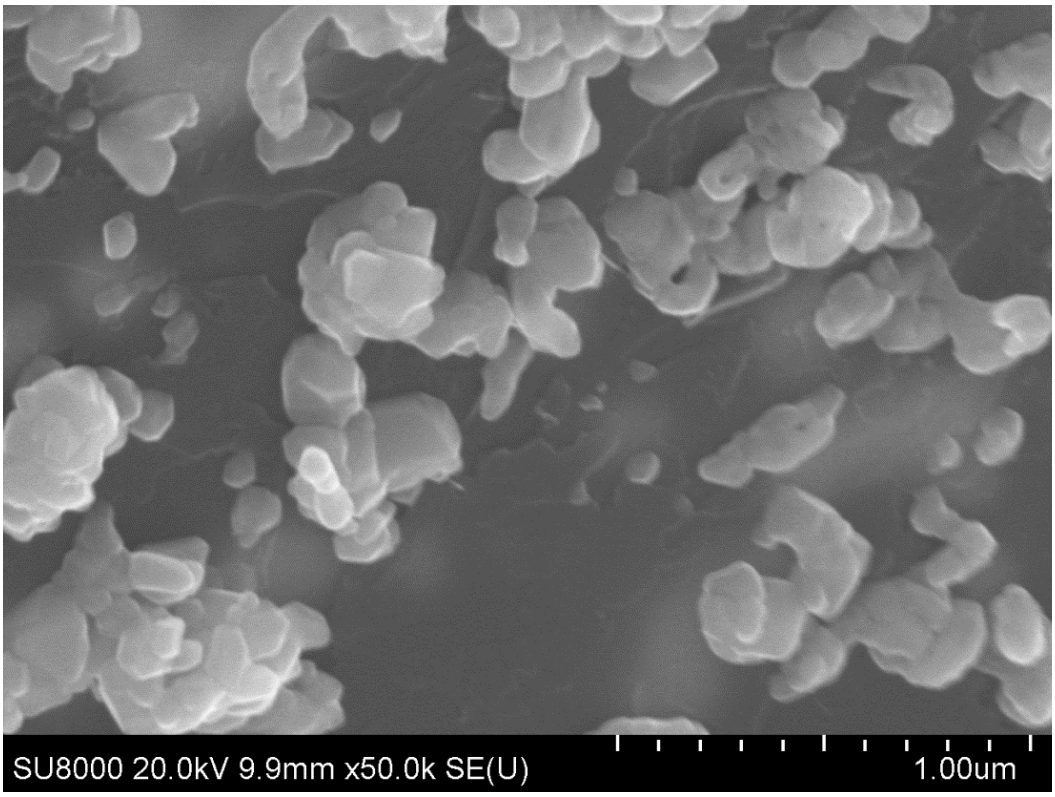

Figure S176. SEM image of graphite powder with WO_3 sample after MW treatment.

SEM images of changes in graphite morphology in the presence of ZnO after MW treatment

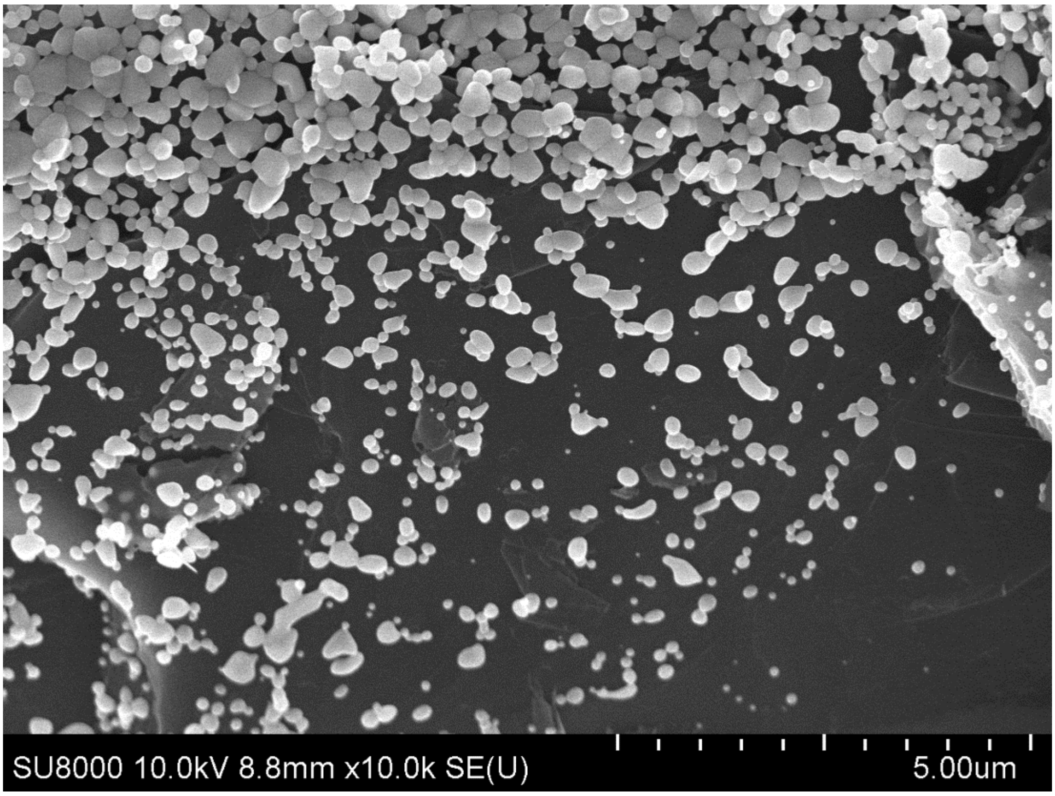

Figure S177. SEM image of graphite powder with ZnO sample after MW treatment.

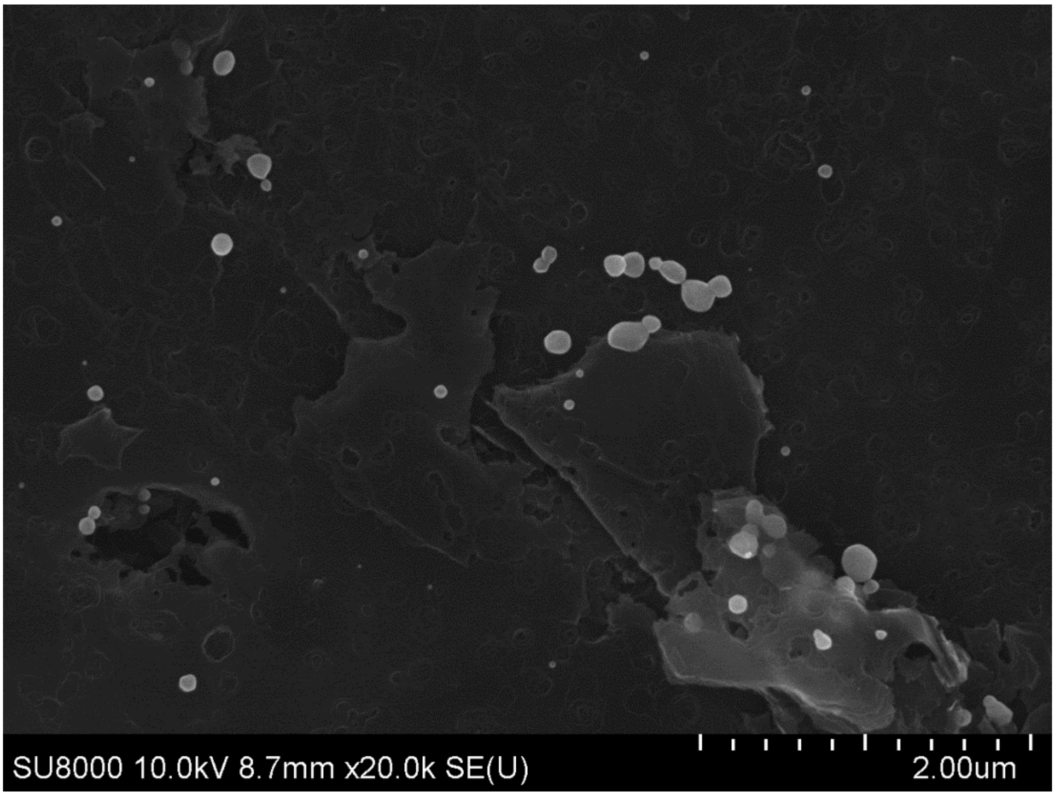

Figure S178. SEM image of graphite powder with ZnO sample after MW treatment.

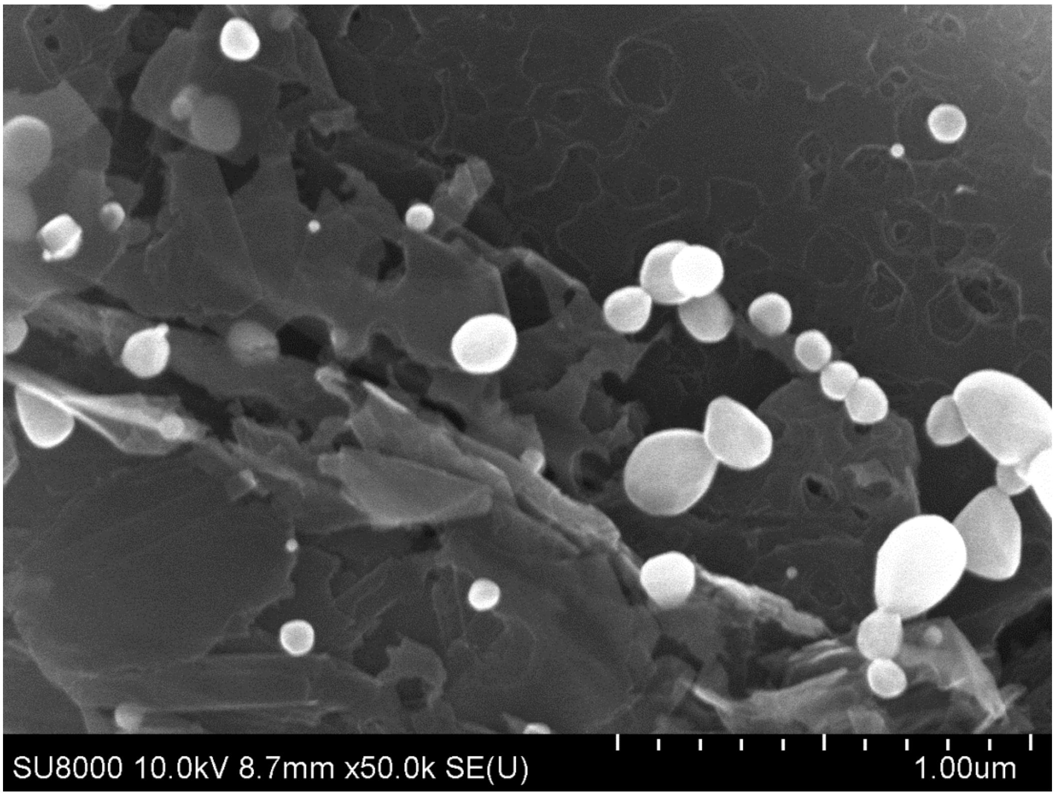

Figure S179. SEM image of graphite powder with ZnO sample after MW treatment.

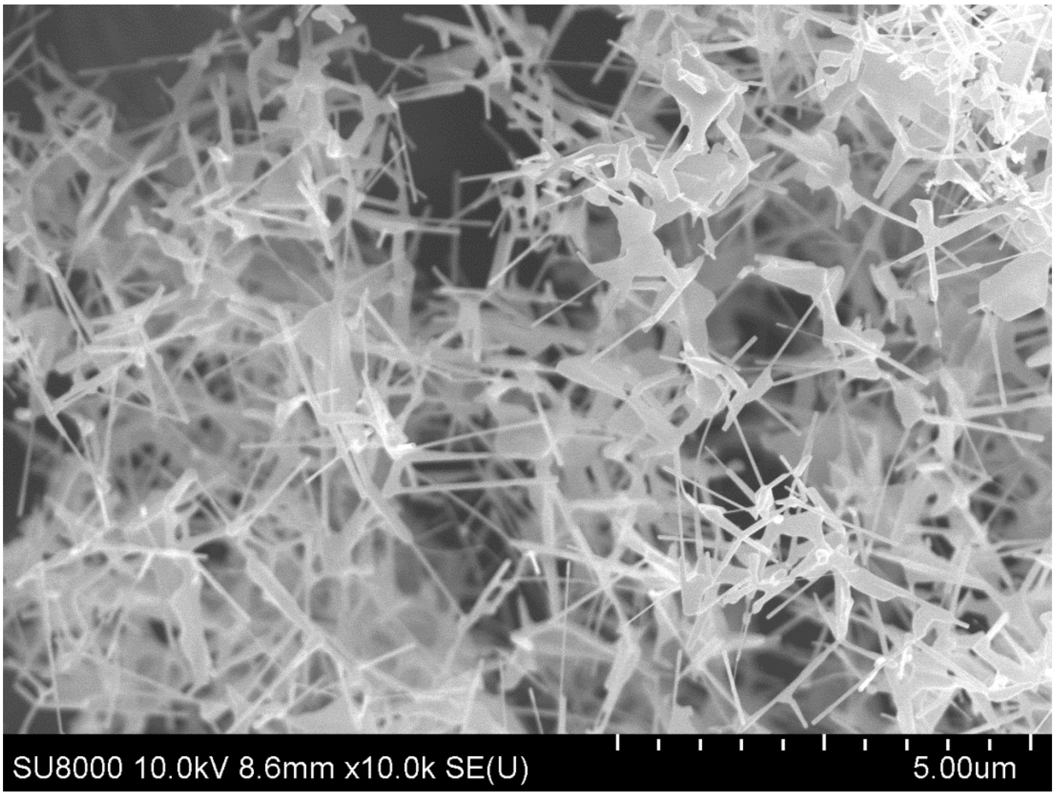

Figure S180. SEM image of graphite powder with ZnO sample after MW treatment (sublimated particles).

SEM images of changes in graphite morphology in the presence of ZrO_2 after MW treatment

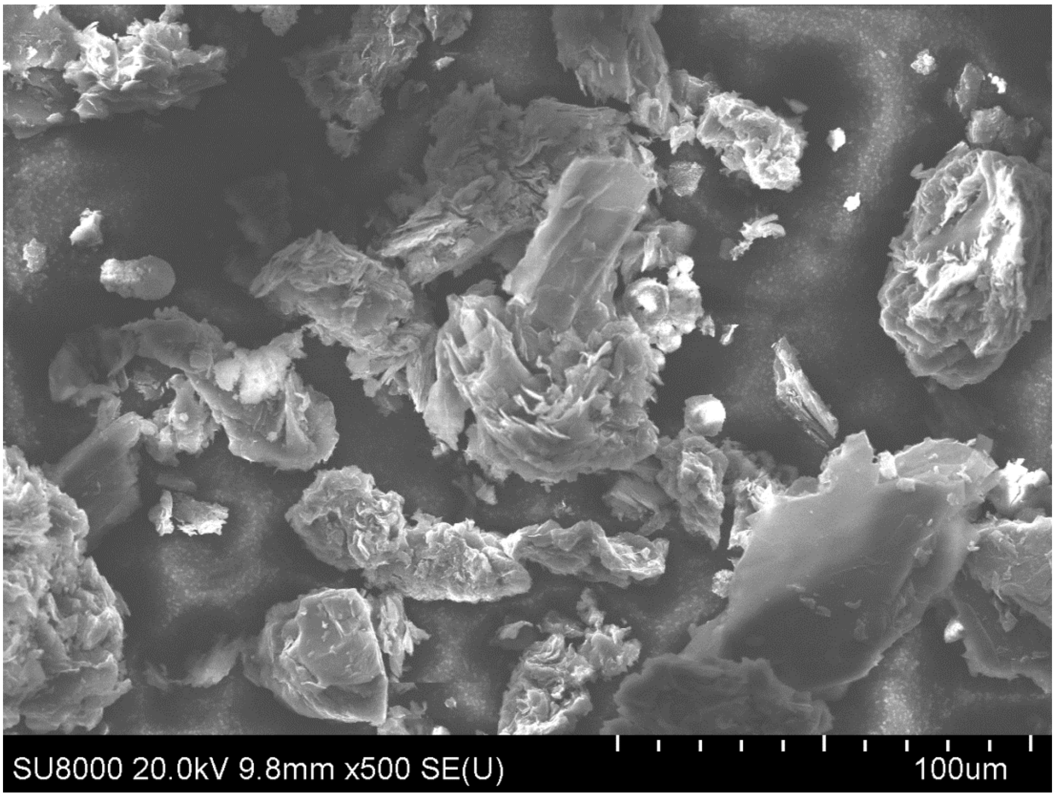

Figure S181. SEM image of graphite powder with ZrO_2 sample after MW treatment.

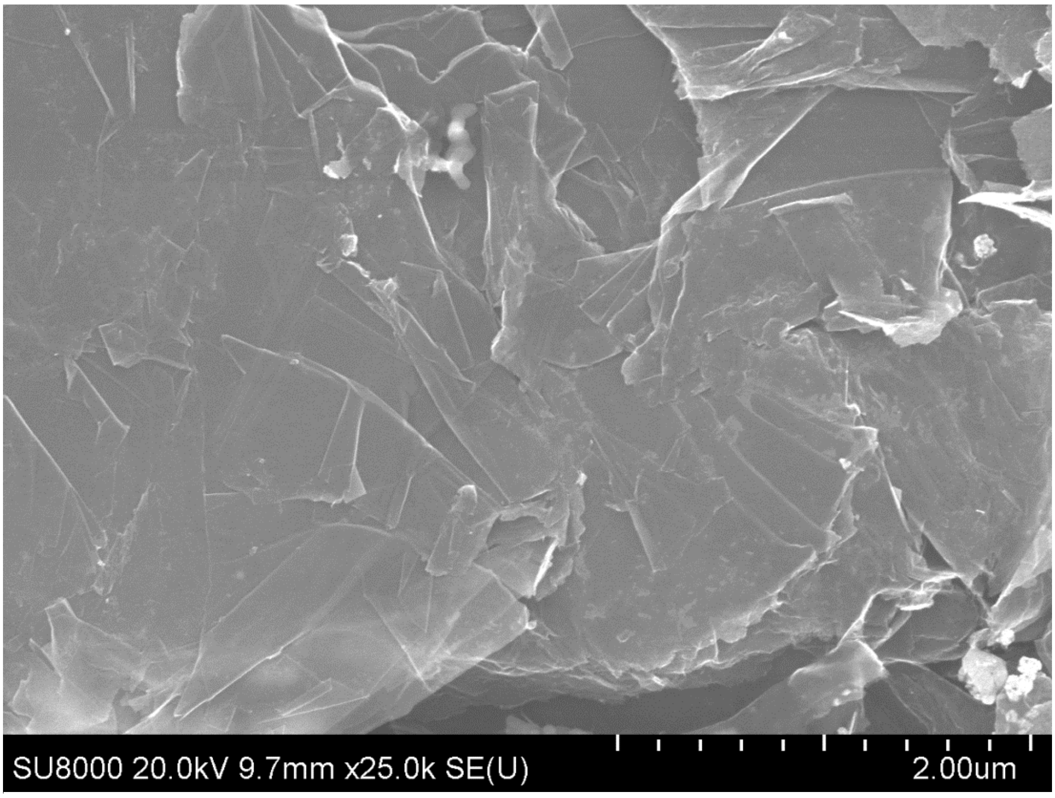

Figure S182. SEM image of graphite powder with ZrO_2 sample after MW treatment.

SEM images of changes in graphite morphology in the presence of TiO_2 after MW treatment

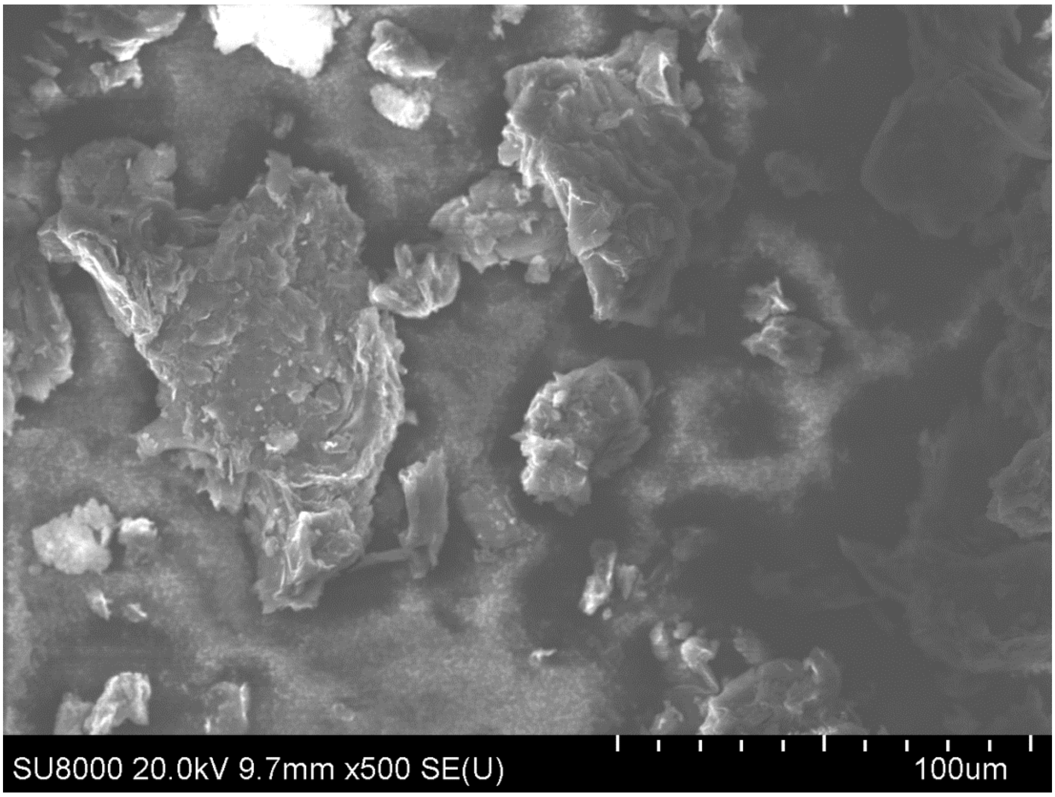

Figure S183. SEM image of graphite powder with TiO_2 sample after MW treatment.

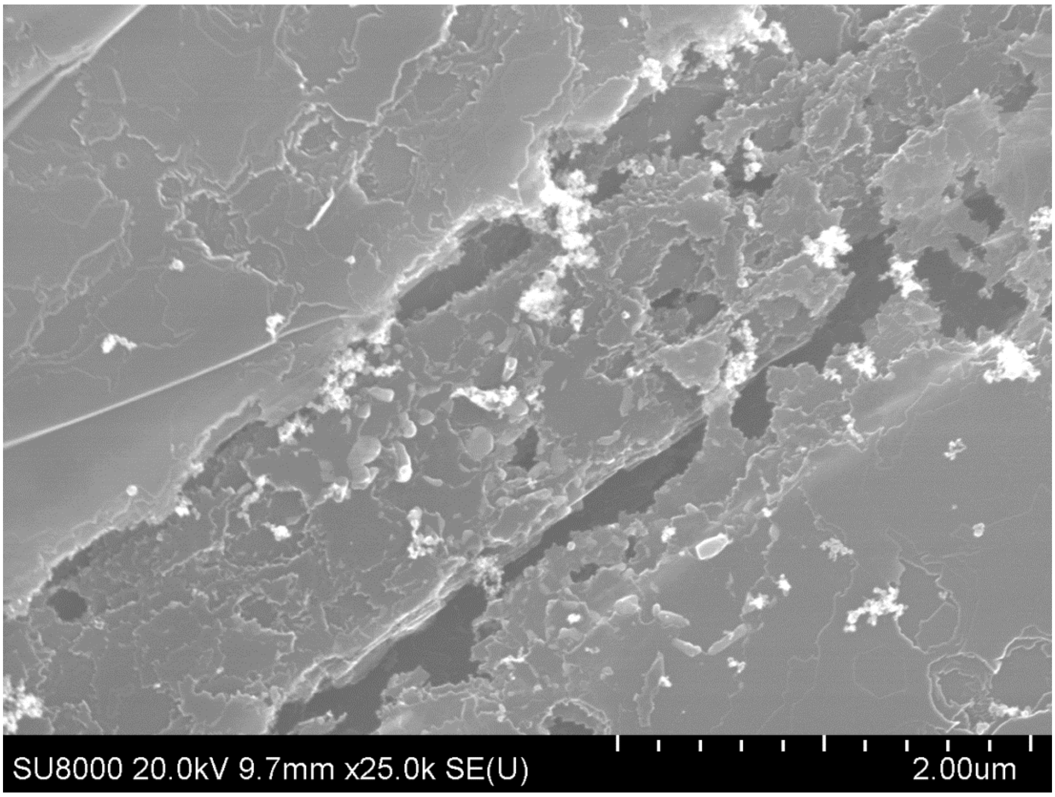

Figure S184. SEM image of graphite powder with TiO_2 sample after MW treatment.

SEM images of changes in graphite morphology in the presence of CoO after MW treatment

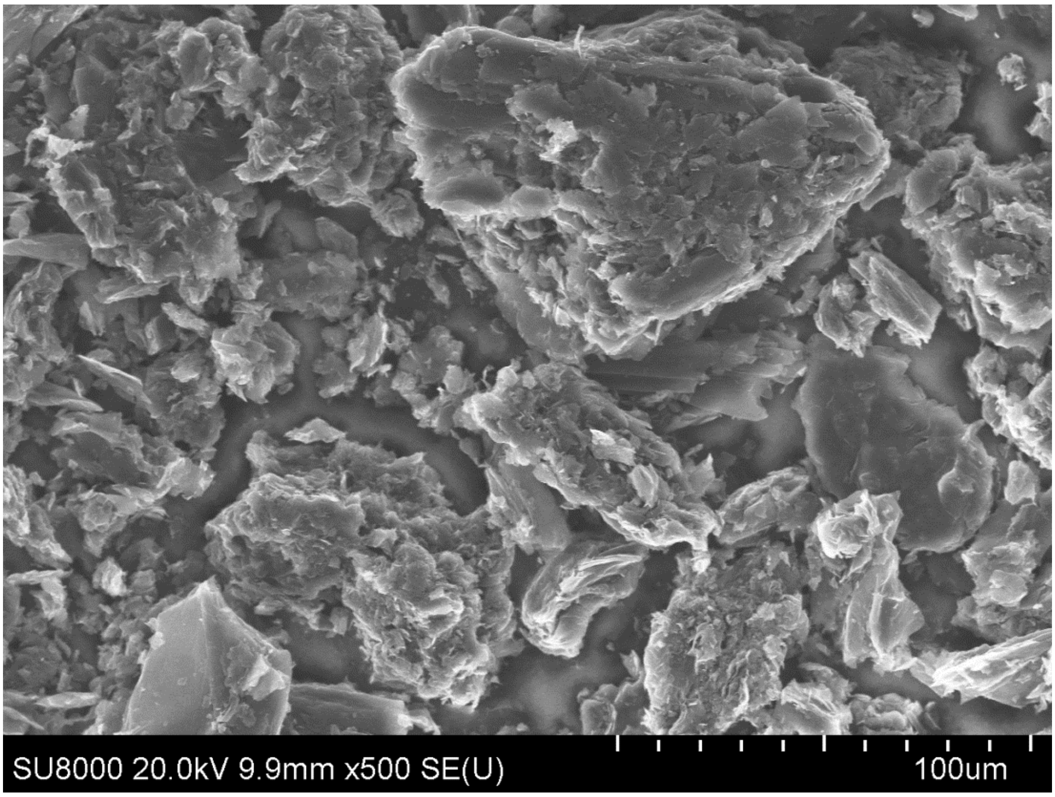

Figure S185. SEM image of graphite powder with CoO sample after MW treatment.

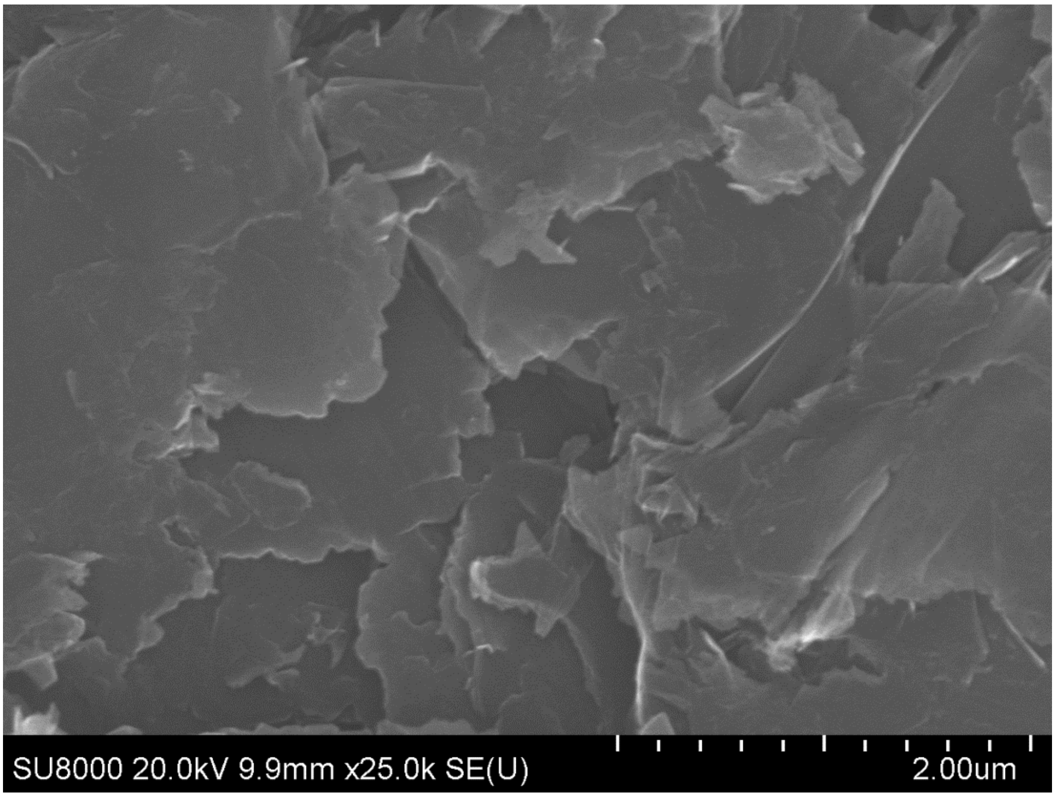

Figure S186. SEM image of graphite powder with CoO sample after MW treatment.

SEM images of changes in graphite morphology in the presence of Fe_2O_3 after MW treatment

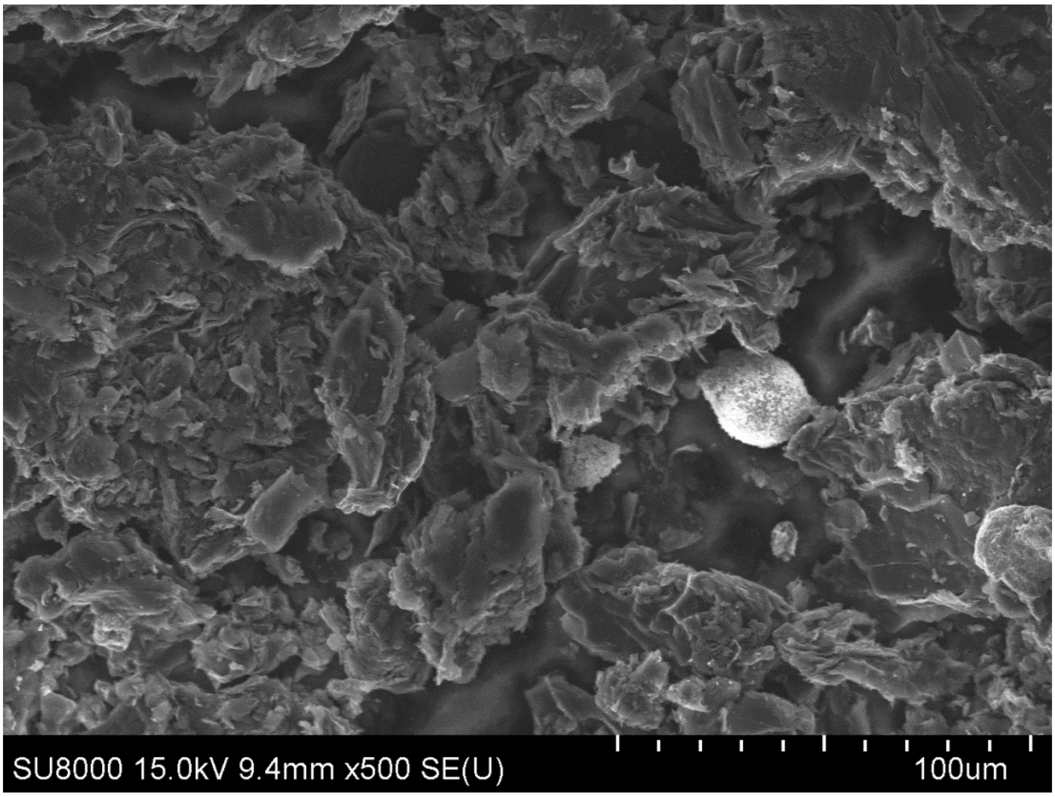

Figure S187. SEM image of graphite powder with Fe_2O_3 sample after MW treatment.

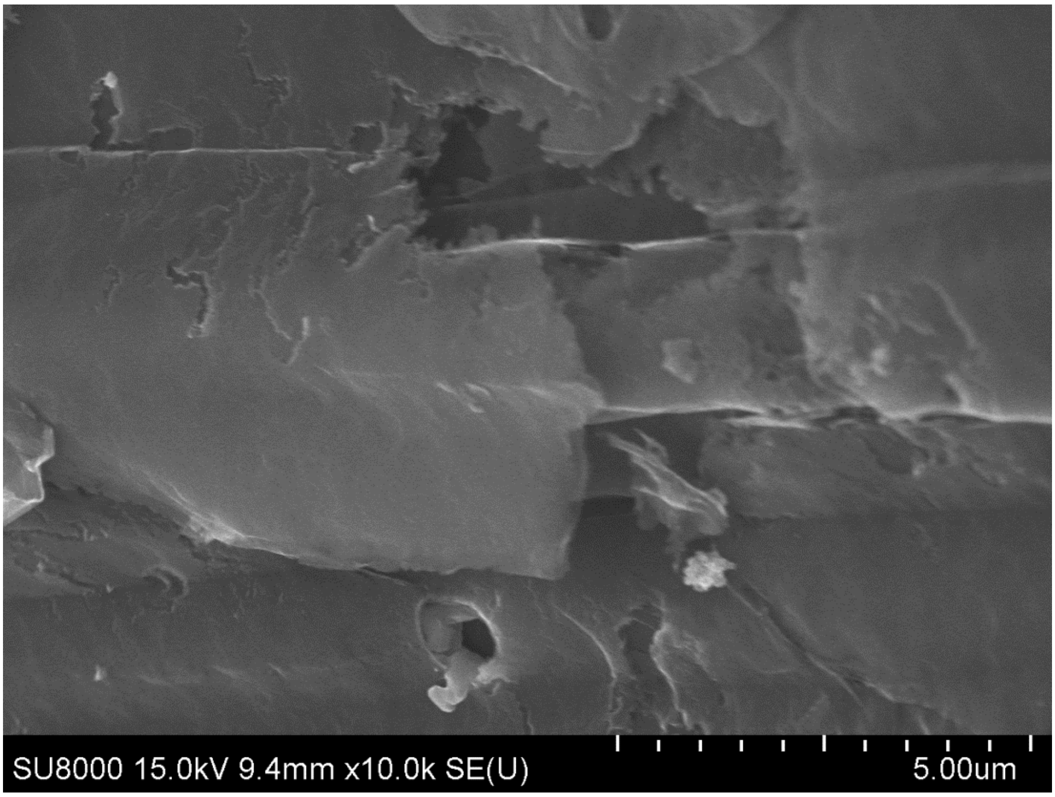

Figure S188. SEM image of graphite powder with Fe_2O_3 sample after MW treatment.

SEM images of changes in graphite morphology in the presence of SnO_2 after MW treatment

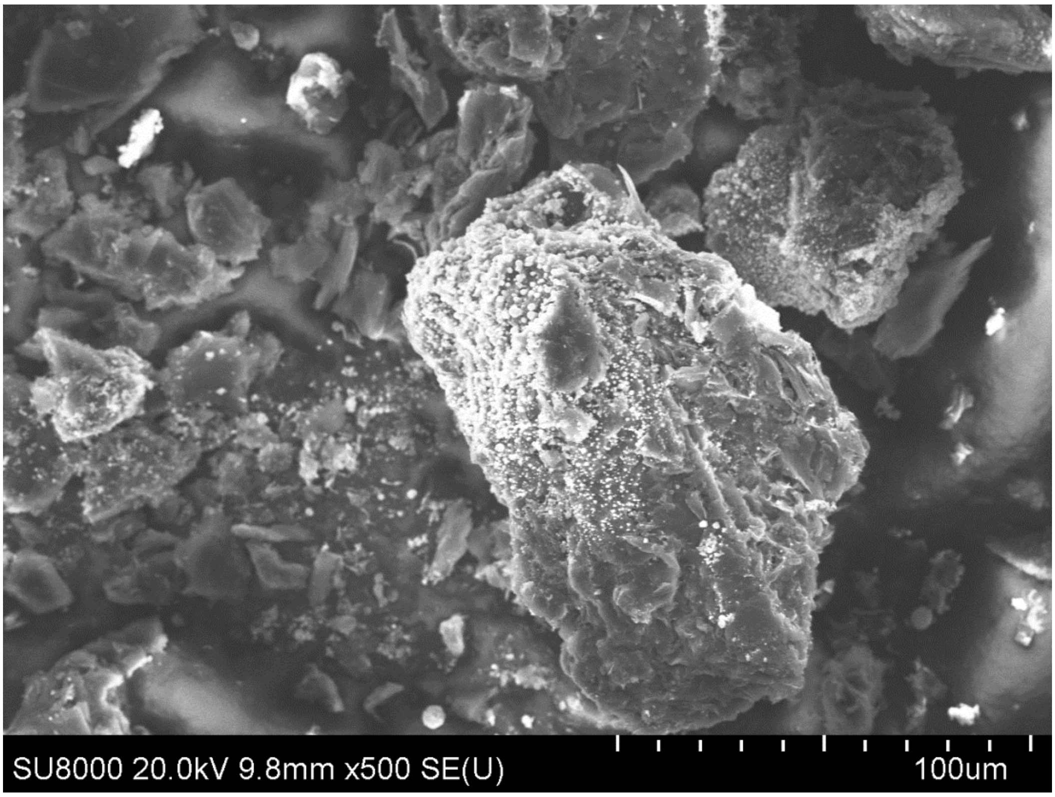

Figure S189. SEM image of graphite powder with SnO_2 sample after MW treatment.

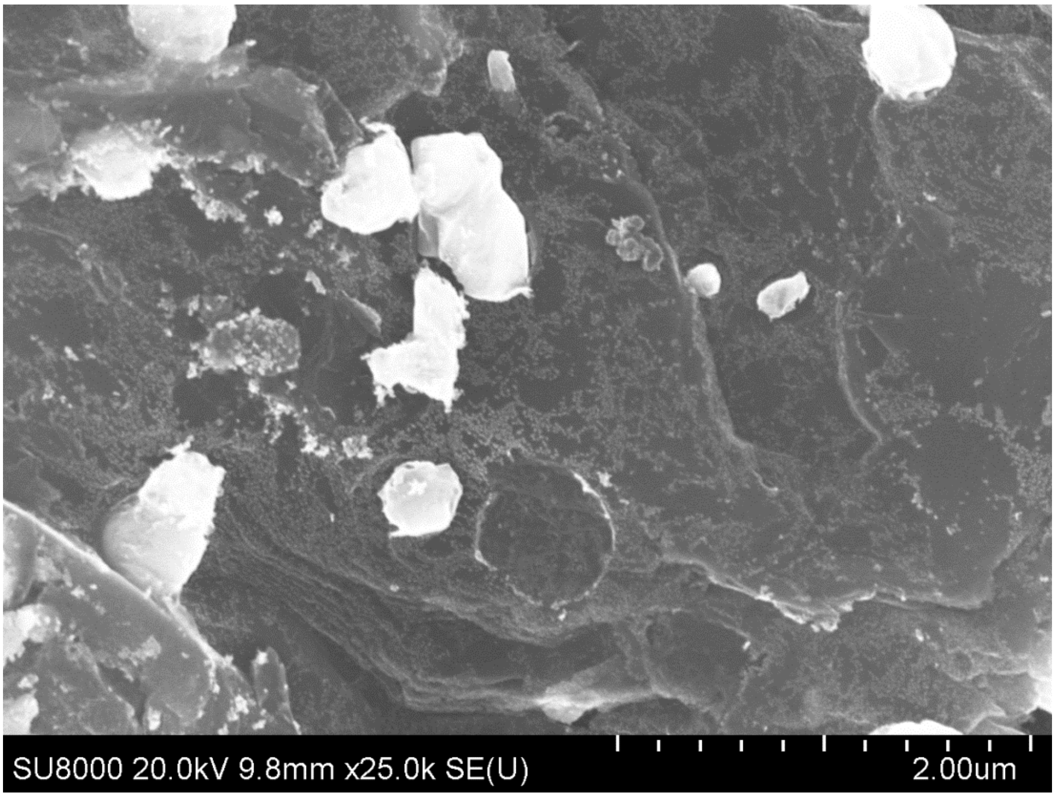

Figure S190. SEM image of graphite powder with SnO_2 sample after MW treatment.

SEM images of changes in graphite morphology in the presence of CuO after MW treatment

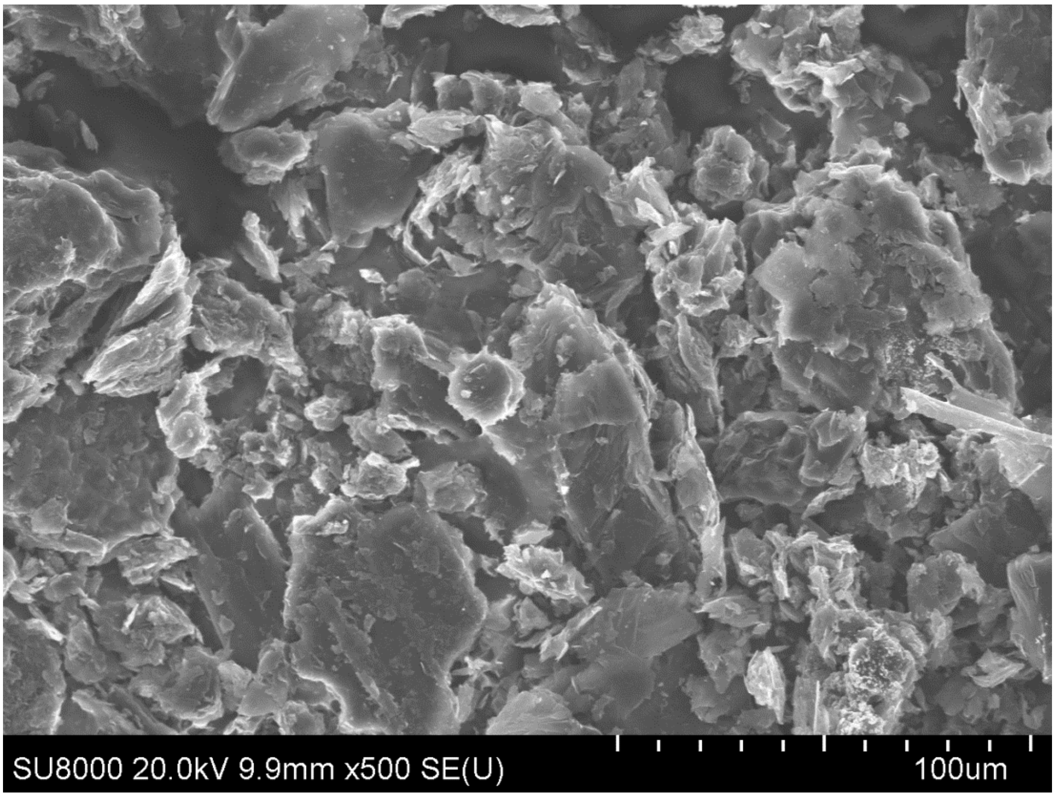

Figure S191. SEM image of graphite powder with CuO sample after MW treatment.

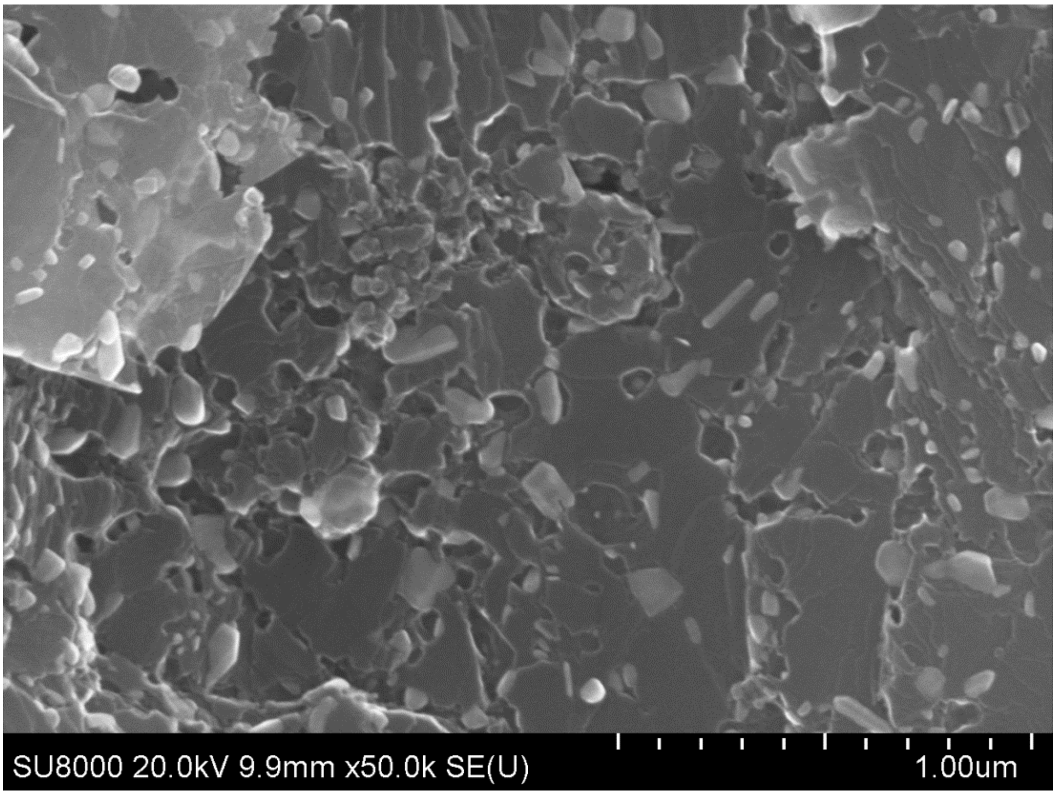

Figure S192. SEM image of graphite powder with CuO sample after MW treatment.

SEM images of changes in graphite morphology in the presence of Y_2O_3 after MW treatment

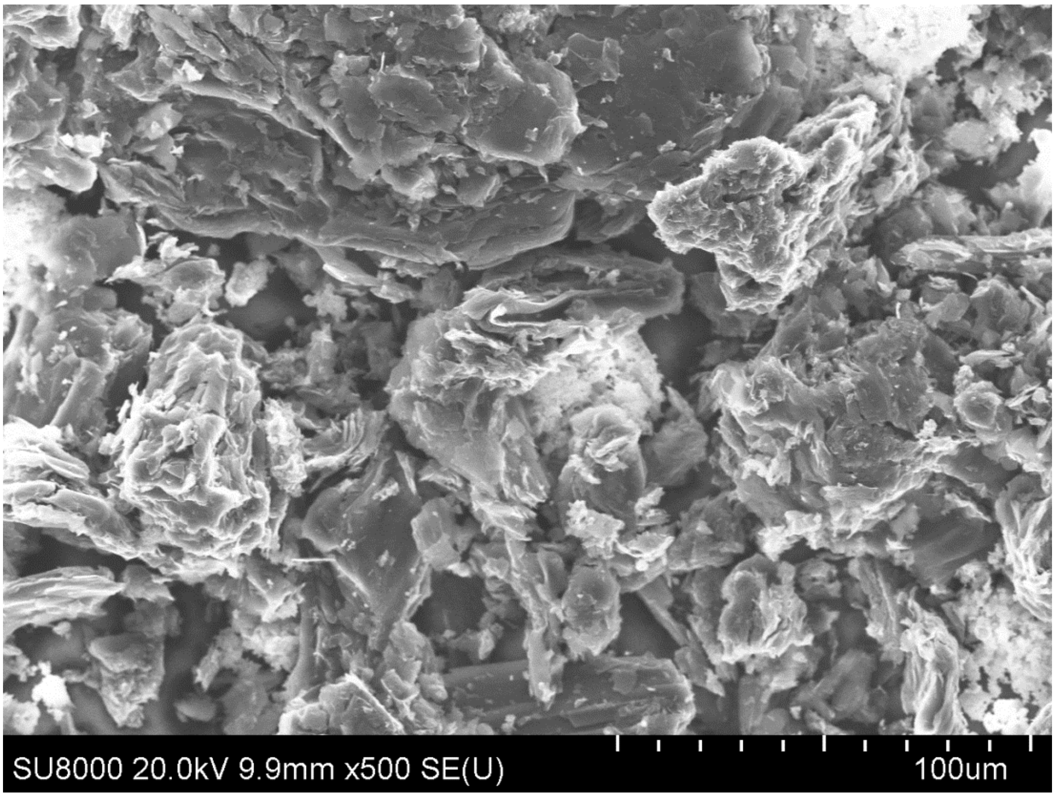

Figure S193. SEM image of graphite powder with Y_2O_3 sample after MW treatment.

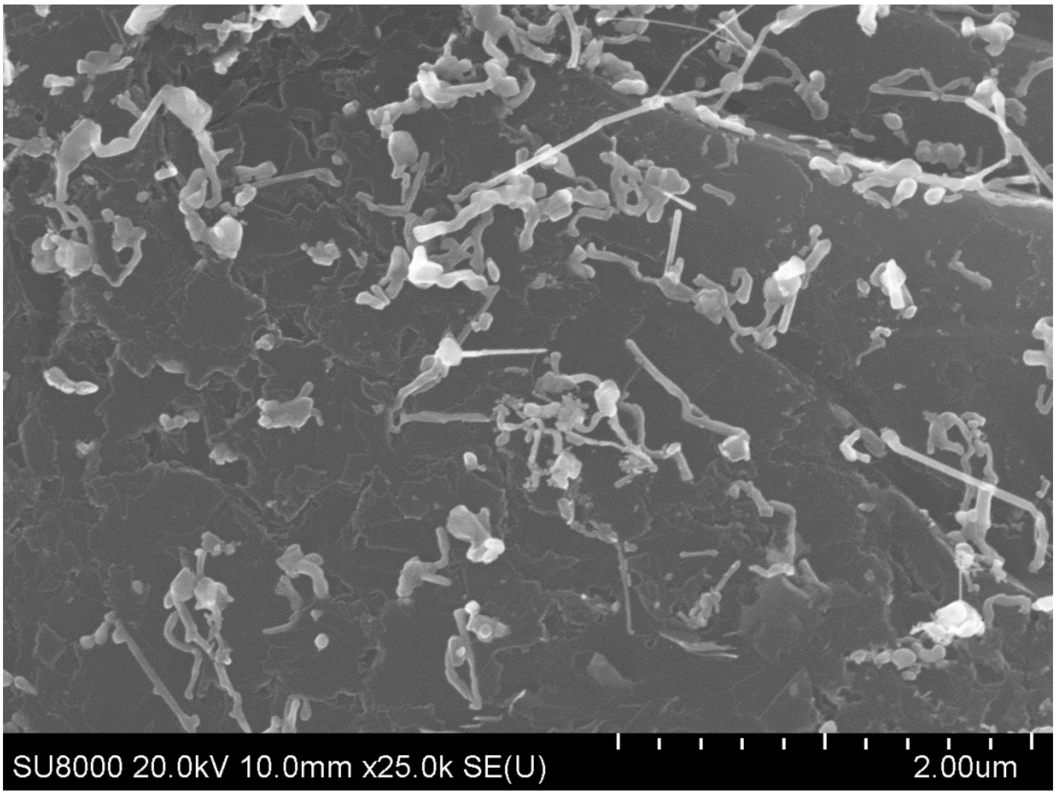

Figure S194. SEM image of graphite powder with Y_2O_3 sample after MW treatment.

SEM images of changes in graphite morphology in the presence of MgO after MW treatment

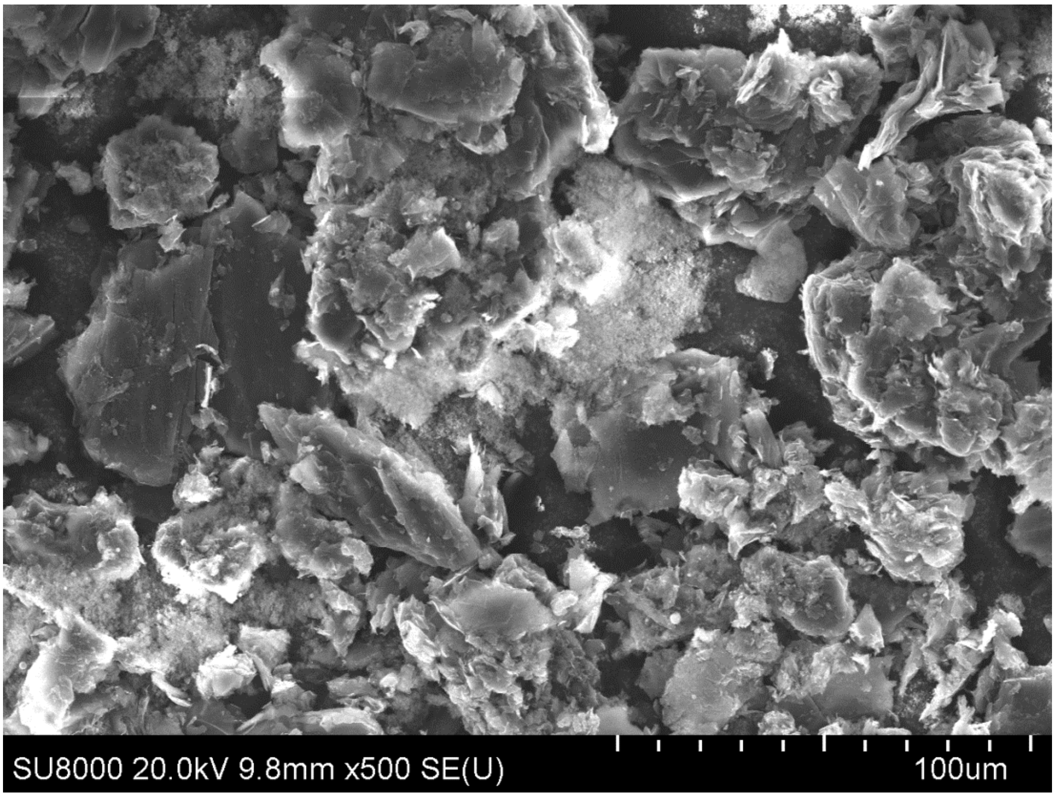

Figure S195. SEM image of graphite powder with MgO sample after MW treatment.

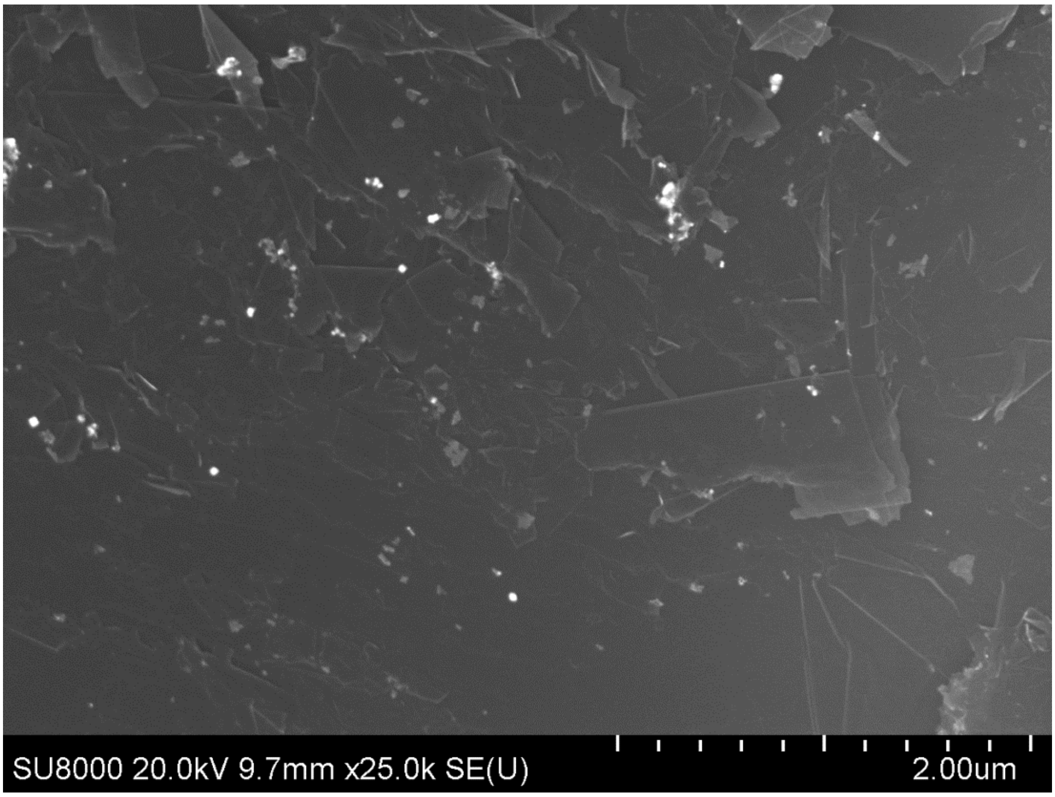

Figure S196. SEM image of graphite powder with MgO sample after MW treatment.

SEM images of changes in graphite morphology in the presence of Cr_2O_3 after MW treatment

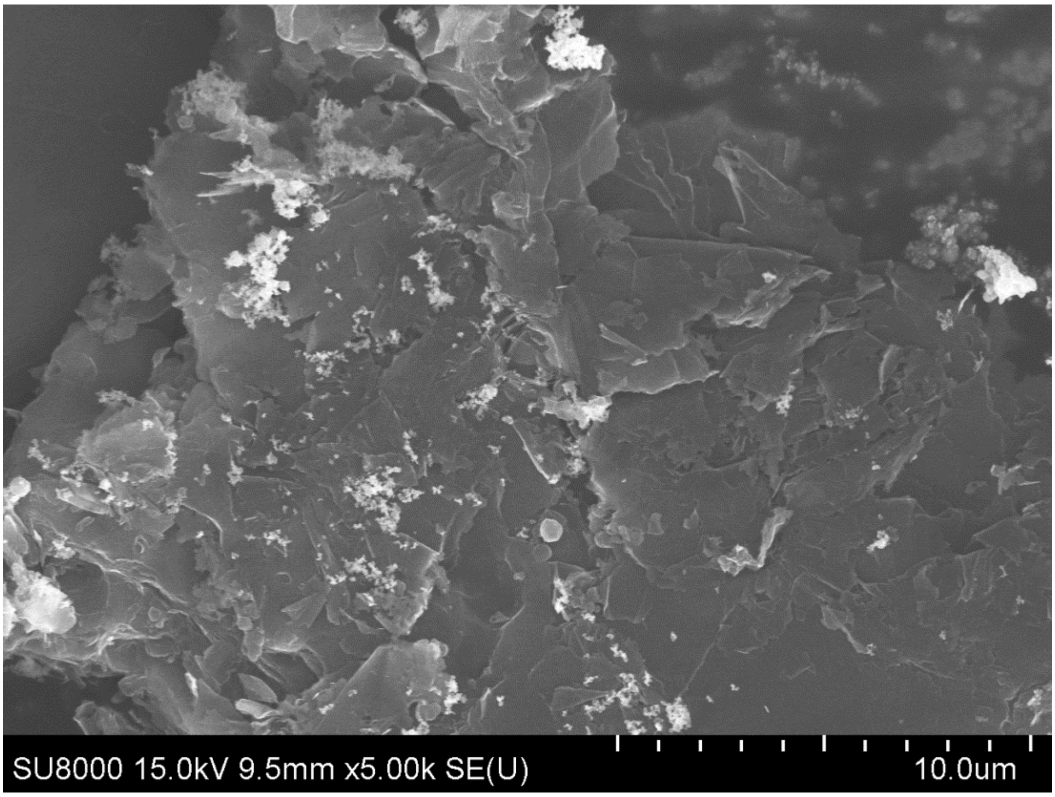

Figure S197. SEM image of graphite powder with Cr_2O_3 sample after MW treatment.

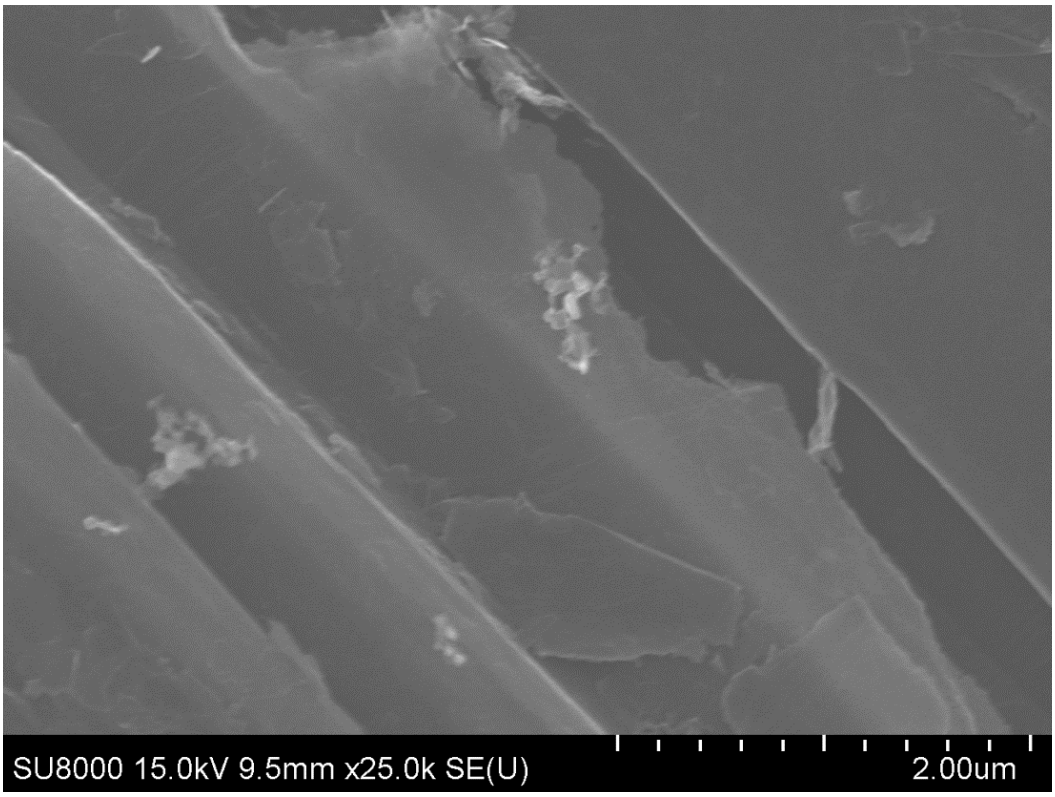

Figure S198. SEM image of graphite powder with Cr_2O_3 sample after MW treatment.

SEM images of changes in graphite morphology in the presence of ZrO_2-SiO_2 after MW treatment

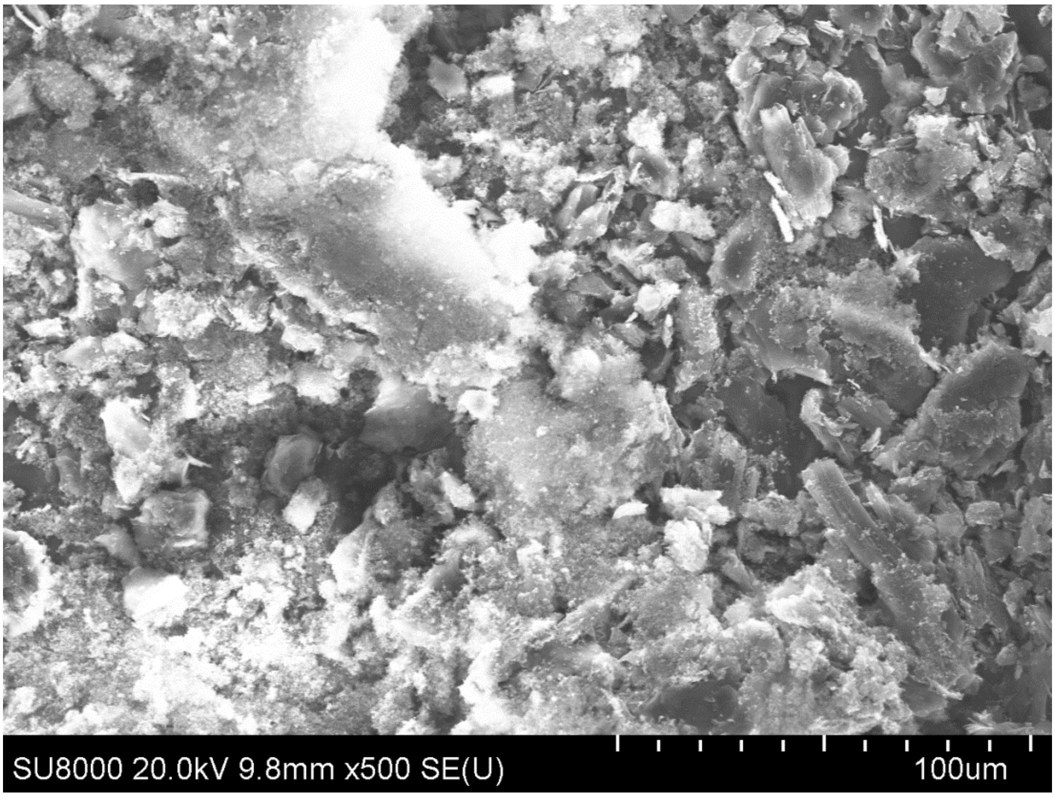

Figure S199. SEM image of graphite powder with ZrO_2-SiO_2 sample after MW treatment.

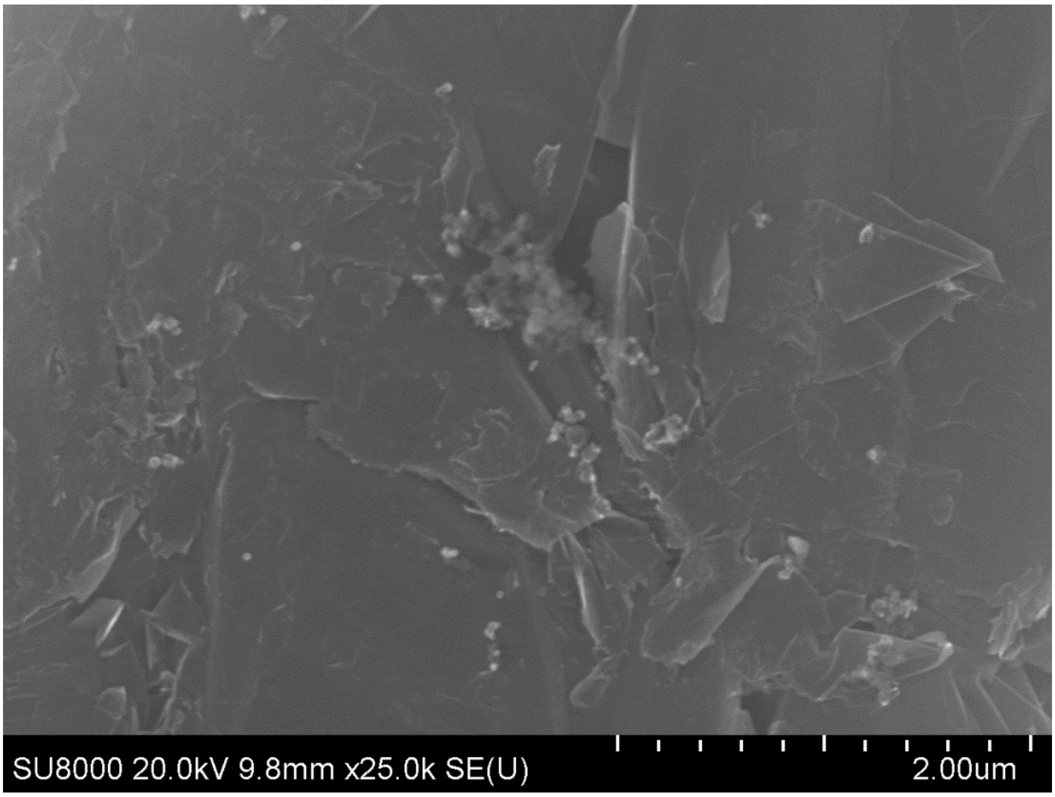

Figure S200. SEM image of graphite powder with ZrO_2-SiO_2 sample after MW treatment.

SEM images and the macro photographs of samples before and after MW treatment

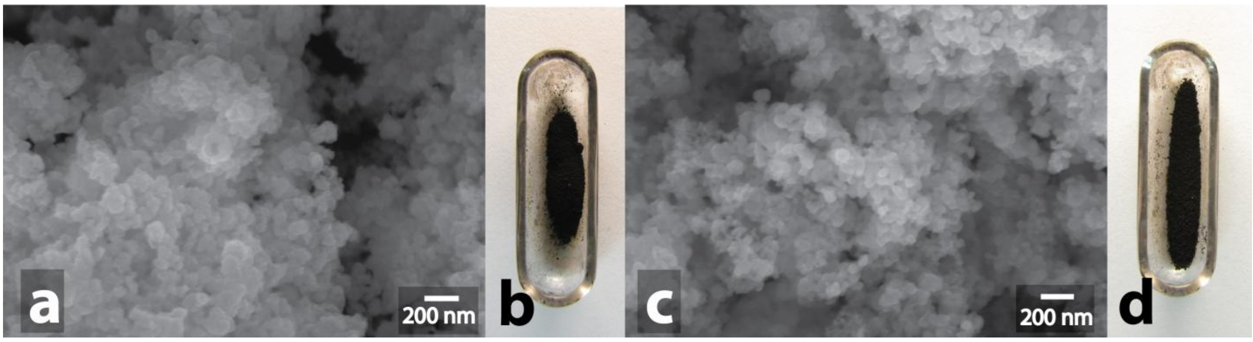

Figure S201. Initial Cu powder: SEM image (a) and macrophotography (b); Cu powder after MW-treatment: SEM image (c) and macrophotography (d).

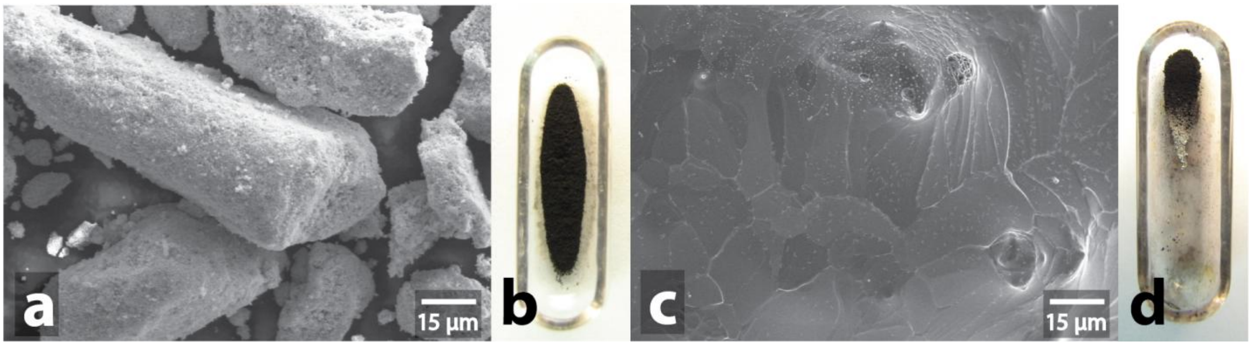

Figure S202. Initial Pt powder: SEM image (a) and macrophotography (b); Pt powder after MW-treatment: SEM image (c) and macrophotography (d).

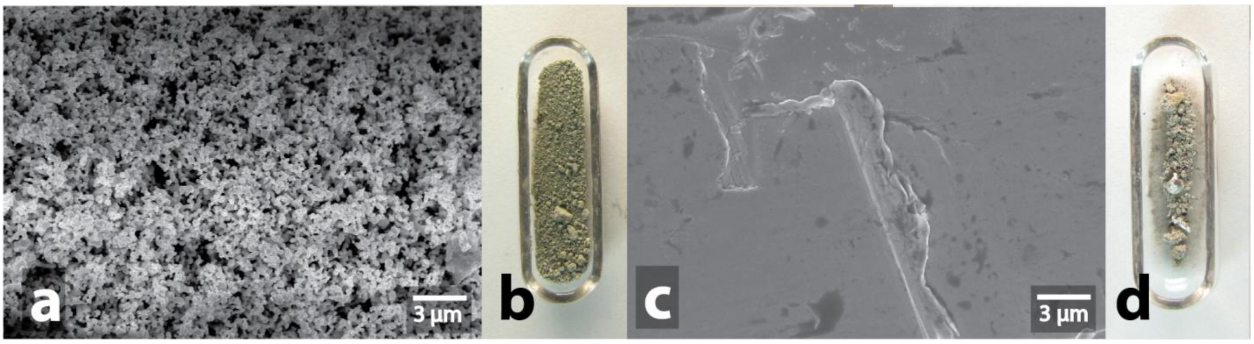

Figure S203. Initial Ag powder: SEM image (a) and macrophotography (b); Ag powder after MW-treatment: SEM image (c) and macrophotography (d).

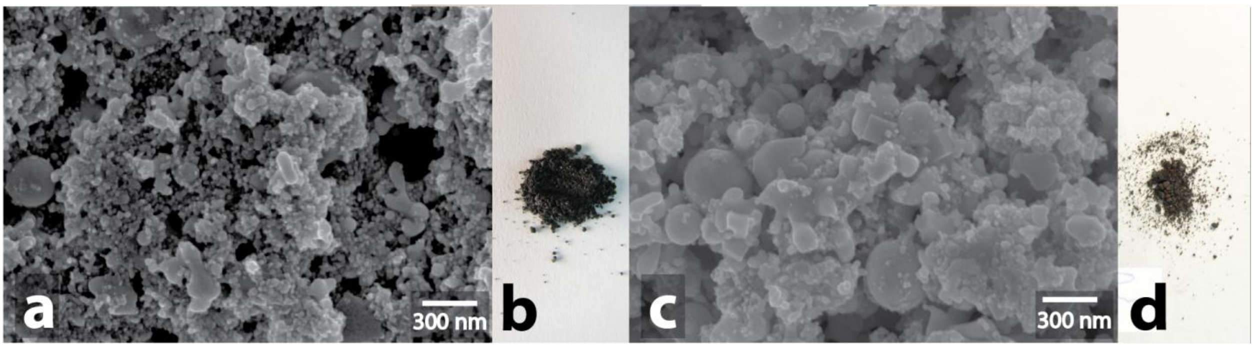

Figure S204. Initial Re powder: SEM image (a) and macrophotography (b); Re powder after MW-treatment: SEM image (c) and macrophotography (d).

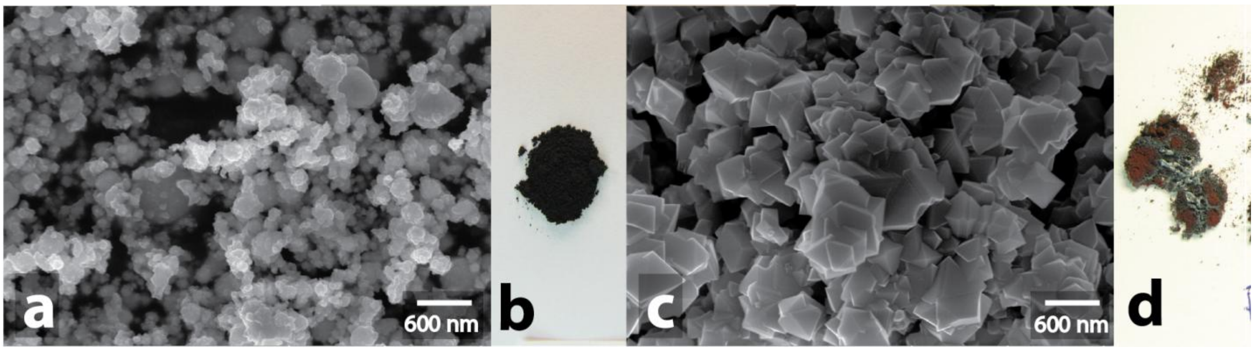

Figure S205. Initial Fe/C powder: SEM image (a) and macrophotography (b); Fe/C powder after MW-treatment: SEM image (c) and macrophotography (d).

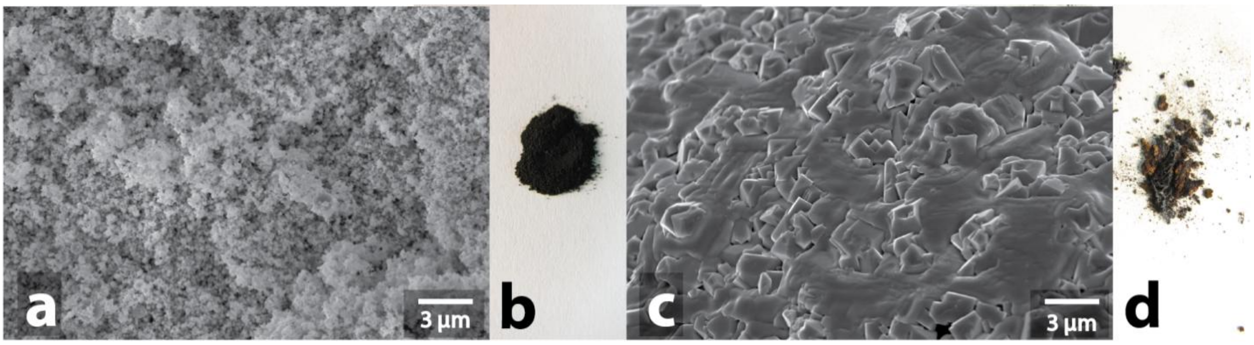

Figure S206. Initial Mo-Fe-C powder: SEM image (a) and macrophotography (b); Mo-Fe-C powder after MW-treatment: SEM image (c) and macrophotography (d).

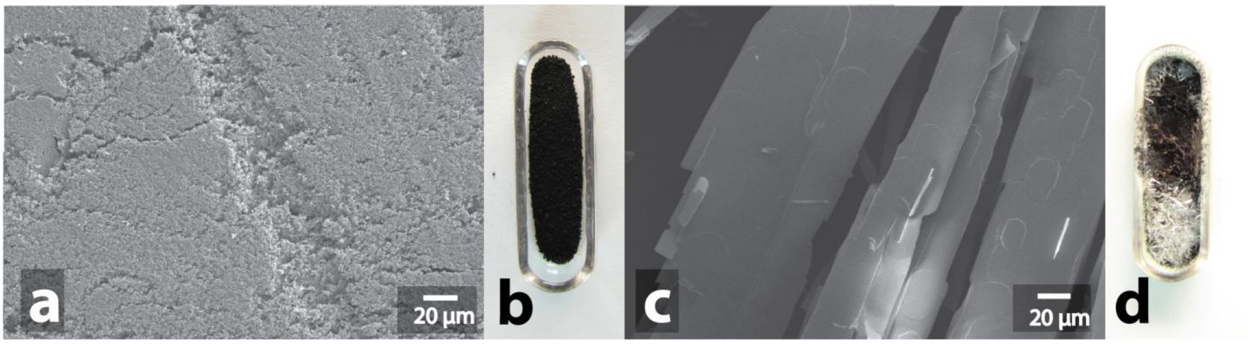

Figure S207. Initial Mo/C powder: SEM image (a) and macrophotography (b); Mo/C powder after MW-treatment: SEM image (c) and macrophotography (d).

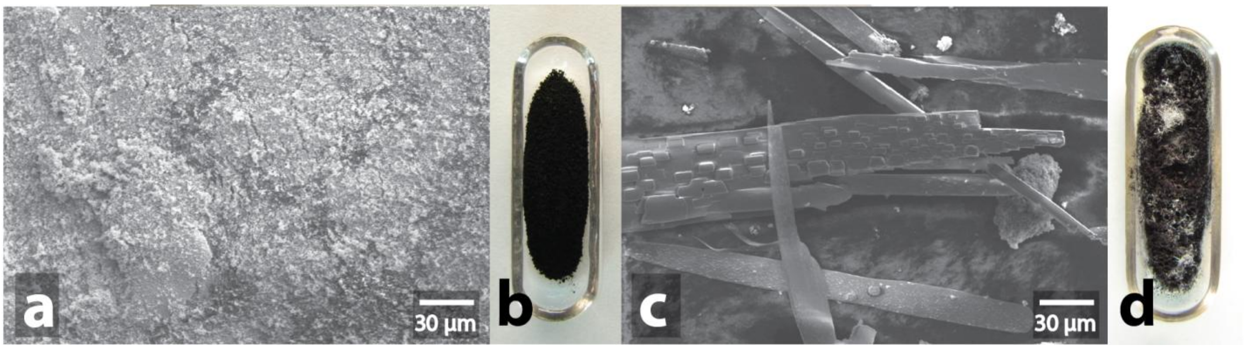

Figure S208. Initial MoS_2 powder: SEM image (a) and macrophotography (b); MoS_2 powder after MW-treatment: SEM image (c) and macrophotography (d).

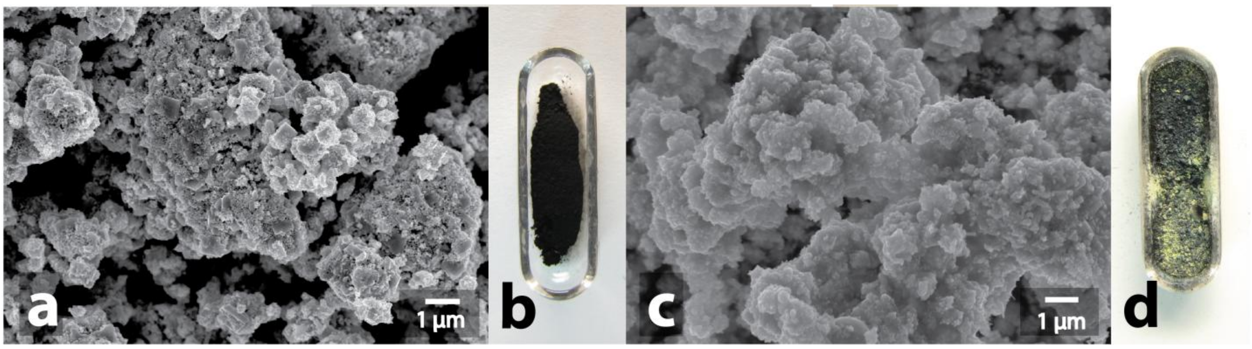

Figure S209. Initial WC powder: SEM image (a) and macrophotography (b); WC powder after MW-treatment: SEM image (c) and macrophotography (d).

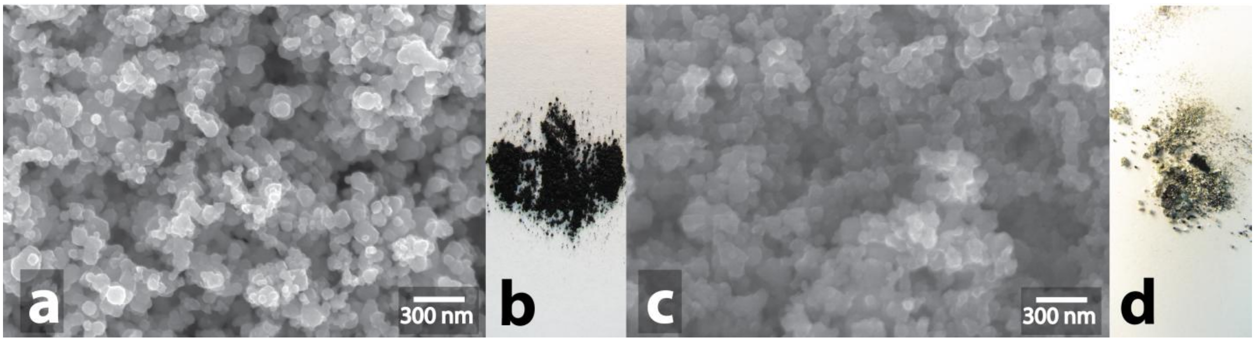

Figure S210. Initial TiC powder: SEM image (a) and macrophotography (b); TiC powder after MW-treatment: SEM image (c) and macrophotography (d).

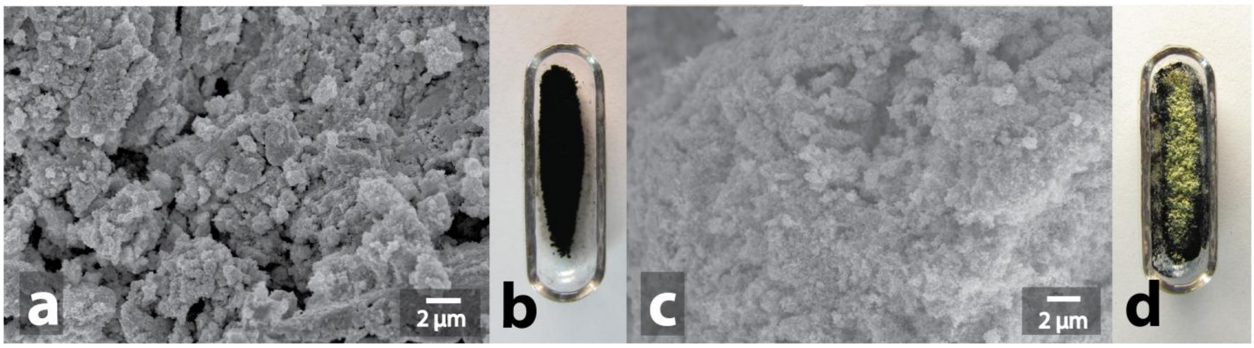

Figure S211. Initial W-C powder: SEM image (a) and macrophotography (b); W-C powder after MW-treatment: SEM image (c) and macrophotography (d).

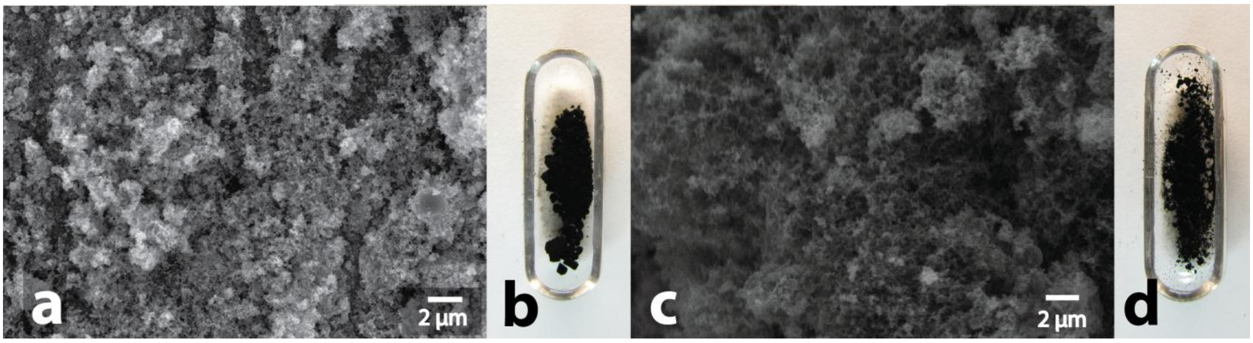

Figure S212. Initial V-C powder: SEM image (a) and macrophotography (b); V-C powder after MW-treatment: SEM image (c) and macrophotography (d).

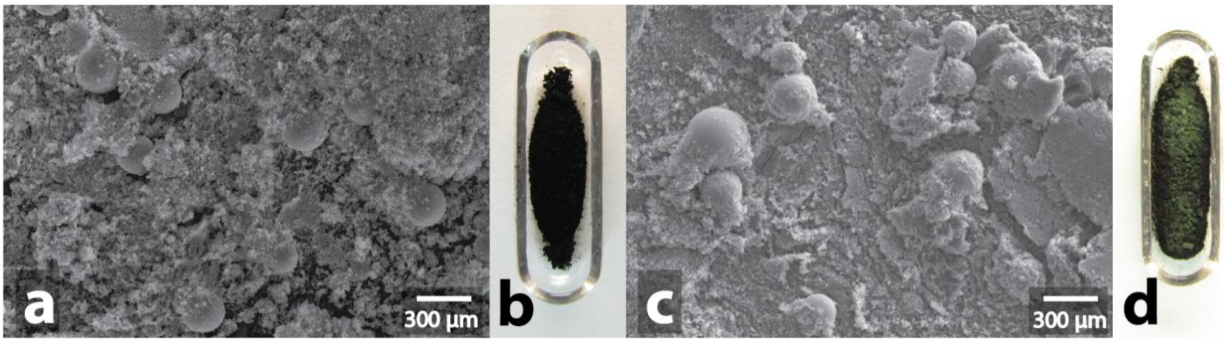

Figure S213. Initial Cr-C powder: SEM image (a) and macrophotography (b); Cr-C powder after MW-treatment: SEM image (c) and macrophotography (d).

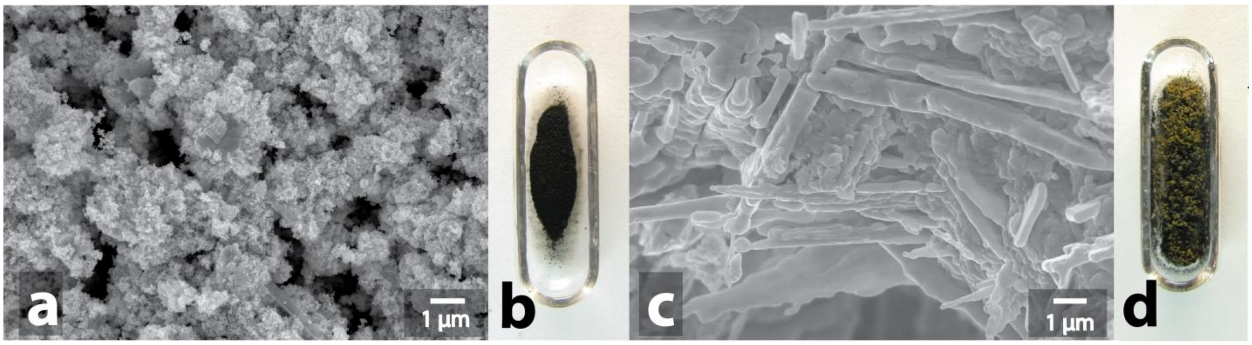

Figure S214. Initial W-V-C powder: SEM image (a) and macrophotography (b); W-V-C powder after MW-treatment: SEM image (c) and macrophotography (d).

XRD data of samples before and after MW processing

XRD diffraction pattern of Ag powder sample after MW treatment

Qualitative analysis results

Phase name	Formula	Figure of merit	Phase reg. detail
Silver	Ag	0.3871114388935761	30652871 (ICDD)

Phase data pattern

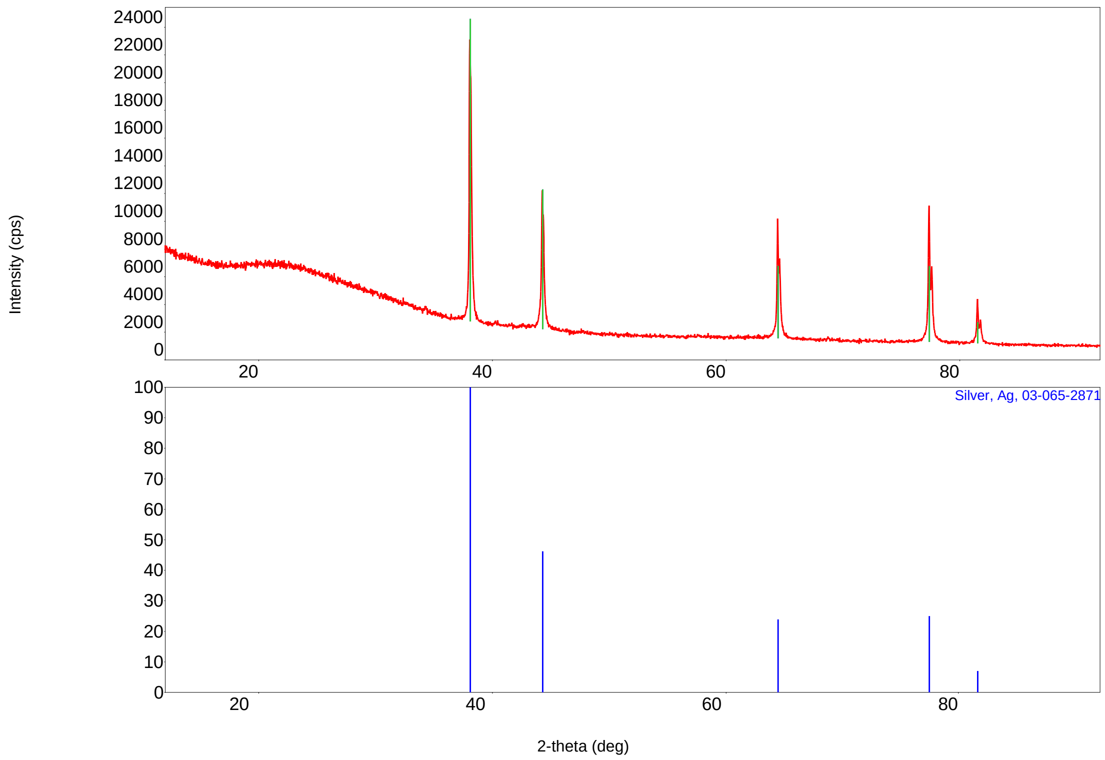

Figure S215. XRD diffraction pattern of Ag powder sample after MW treatment.

XRD diffraction pattern of initial Re powder

Qualitative analysis results

Phase name	Formula	Figure of merit	Phase reg. detail
Rhenium, syn (NR)	Re	0.1610858743832323	50702 (ICDD)
Rhenium Oxide	ReO_3	0.6673425846482536	401155 (ICDD)

Phase data pattern

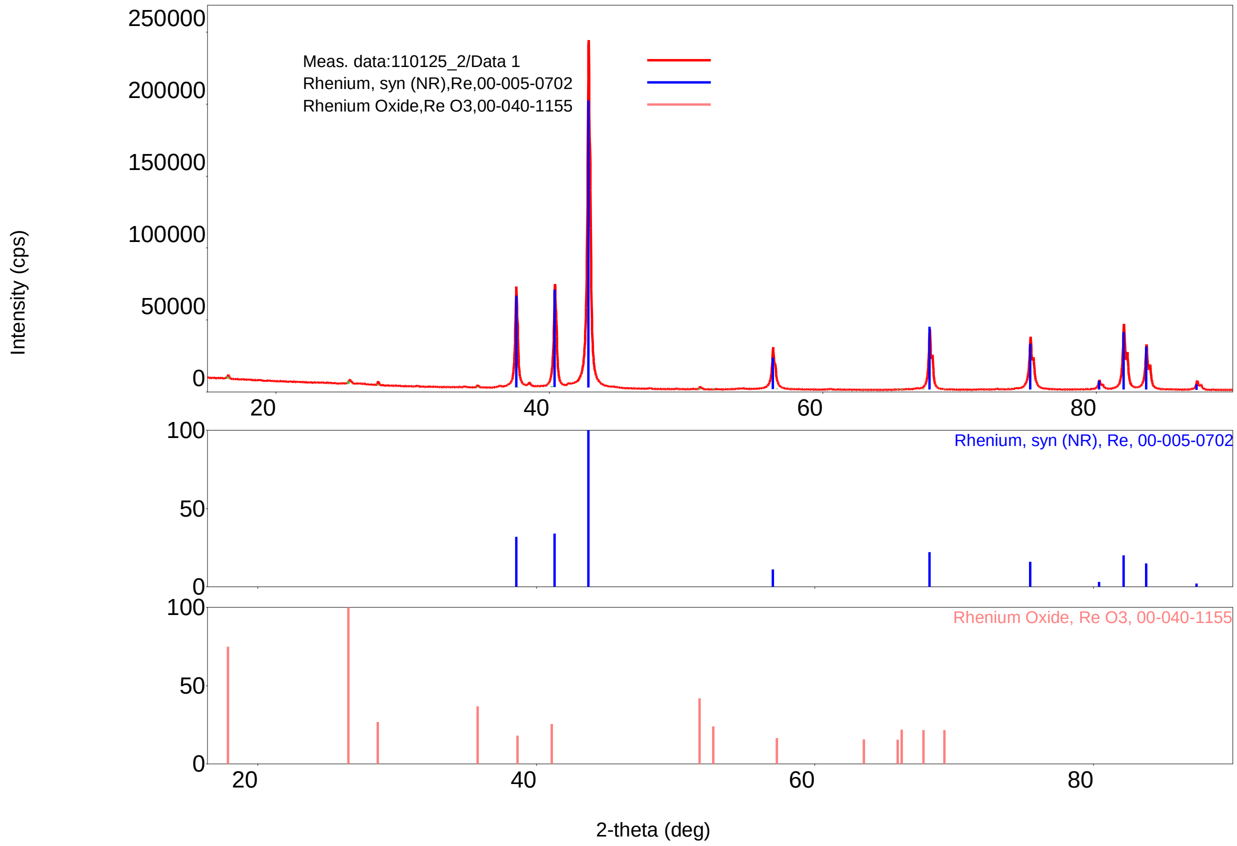

Figure S216. XRD diffraction pattern of initial Re powder.

XRD diffraction pattern of Re powder sample after MW treatment

Qualitative analysis results

Phase name	Formula	Figure of merit	Phase reg. detail
Rhenium, syn (NR)	Re	0.2600243331728576	50702 (ICDD)
Rhenium Oxide	ReO_3	0.5929427543472938	30659764 (ICDD)

Phase data pattern

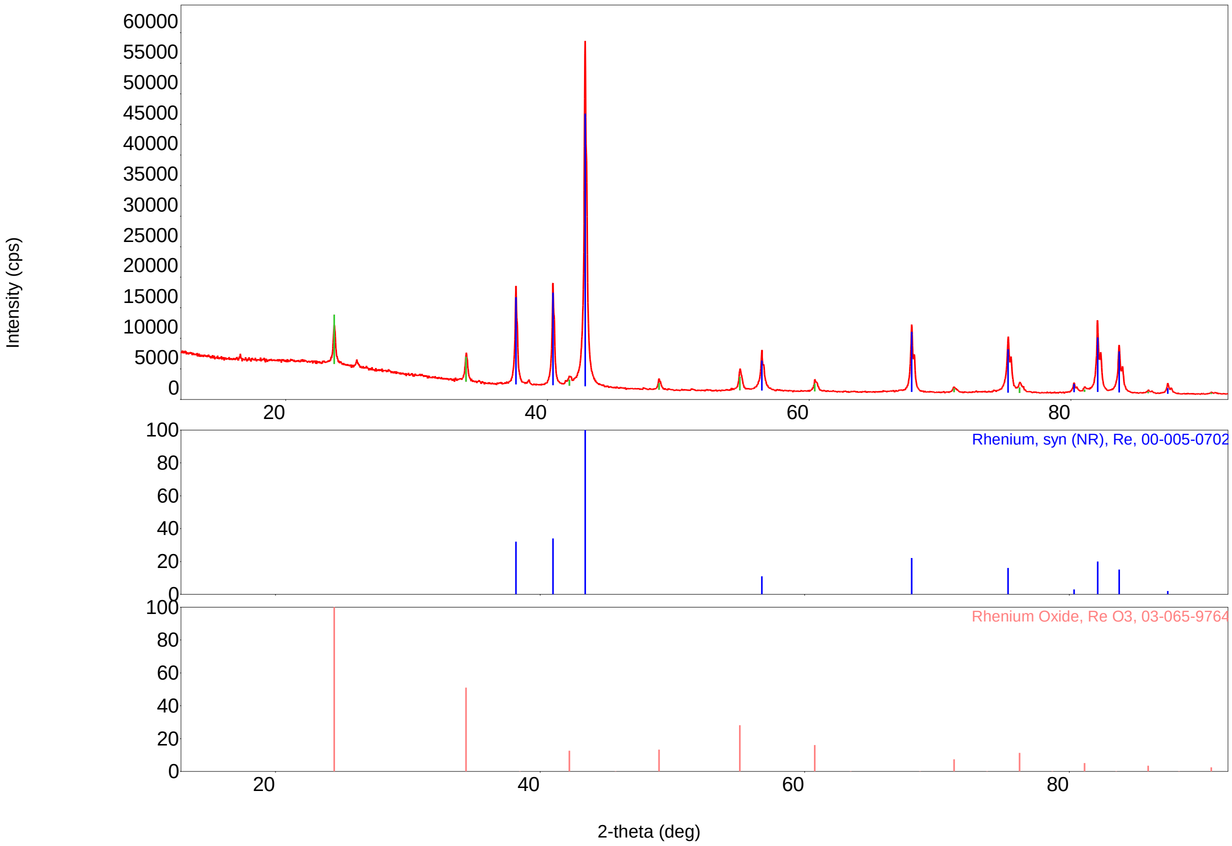

Figure S217. XRD diffraction pattern of Re powder sample after MW treatment.

XRD diffraction pattern of initial WC powder

Qualitative analysis results

Phase name	Formula	Figure of merit	Phase reg. detail
Tungsten Carbide	WC	0.3321868451530957	10892727 (ICDD)

Phase data pattern

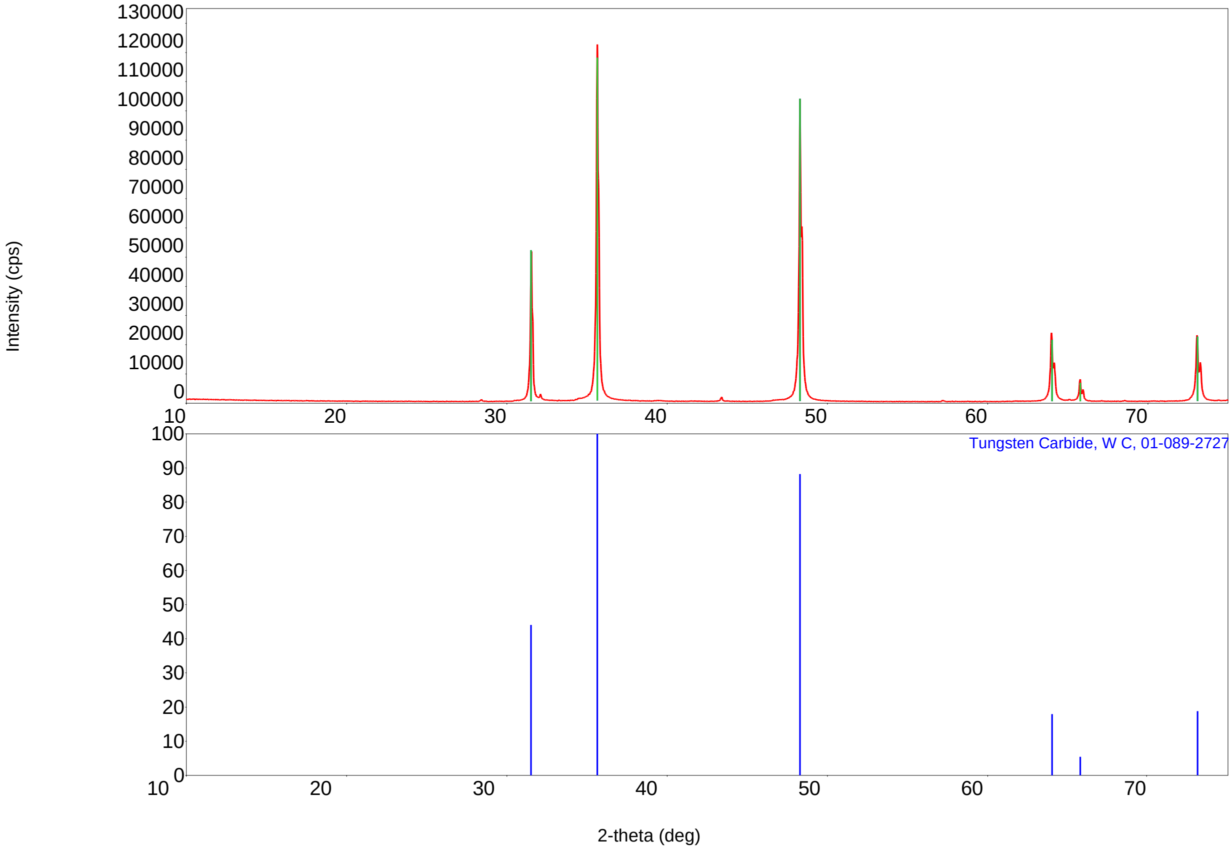

Figure S218. XRD diffraction pattern of initial WC powder.

XRD diffraction pattern of WC powder sample after MW treatment

Qualitative analysis results

Phase name	Formula	Figure of merit	Phase reg. detail
Tungsten Oxide	WO_3	0.3933252532370825	10830950 (ICDD)
Unnamed mineral, syn (NR)	WC	0.7970322278003963	510939 (ICDD)
Wolfram	W	0.9117691763570458	40806 (ICDD)

Phase data pattern

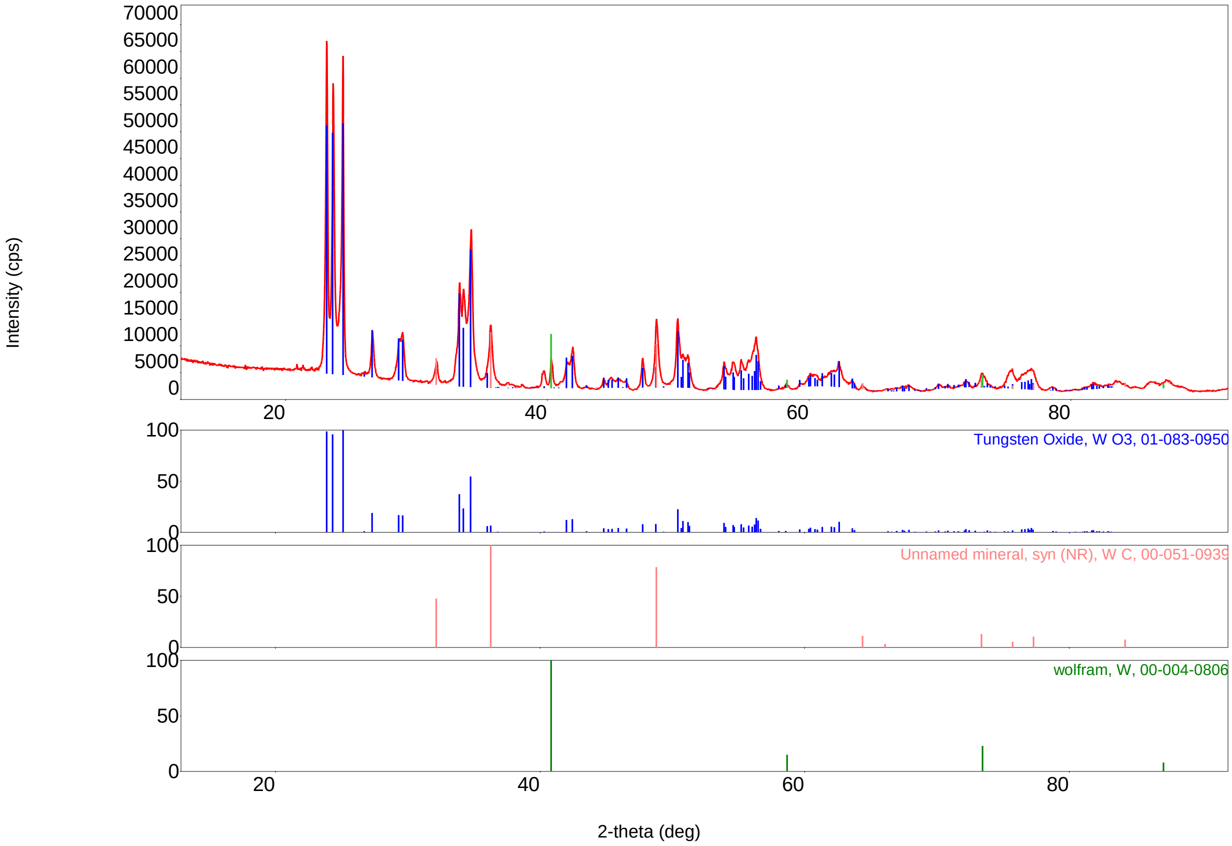

Figure S219. XRD diffraction pattern of WC powder sample after MW treatment with the reference diffraction patterns of WO_3 (PDF 01-083-0950) and other.

XRD diffraction pattern of MoS_2 powder sample after MW treatment

Qualitative analysis results

Phase name	Formula	Figure of merit	Phase reg. detail
Tugarinovite, syn	MoO_2	0.5947432249246876	10781069 (ICDD)
Molybdite, syn	MoO_3	0.763158262495746	10747911 (ICDD)
chi-Mo_4O_{11}	Mo_4O_{11}	1.148356889559762	50337 (ICDD)
Molybdenum	Mo	0.9209986404861873	40809 (ICDD)
Molybdenum Sulfide	Mo_2S_3	1.20307476716514	30656963 (ICDD)

Phase data pattern

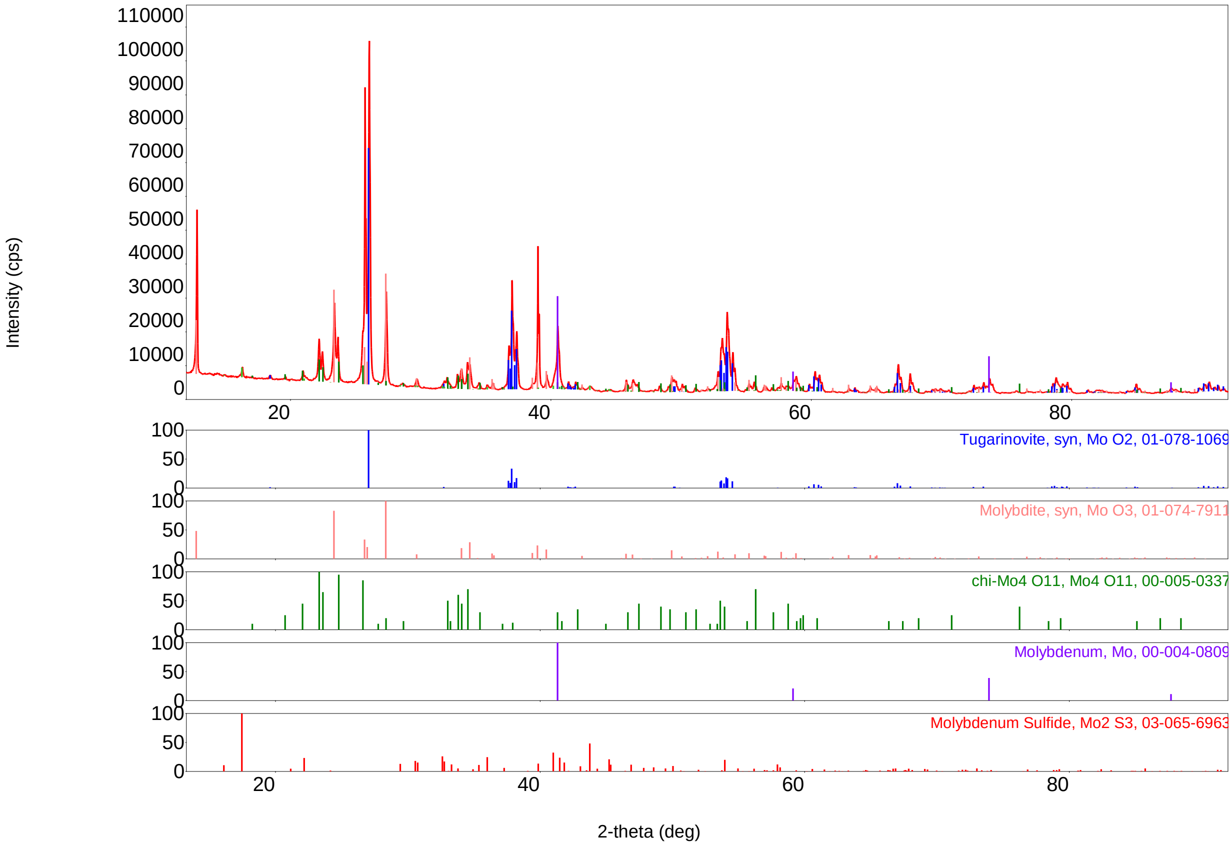

Figure S220. XRD patterns of the of MoS_2 powder sample after MW treatment, with the reference diffraction patterns of MoO_2 (PDF 01-078-1069), MoO_3 (PDF 01-074-7911), Mo_4O_{11} (PDF 00-005-0337) and other.

XRD diffraction pattern of initial W-C powder

Qualitative analysis results

Phase name	Formula	Figure of merit	Phase reg. detail
Tungsten Carbide	WC_{1-x}	0.8545924599599198	201316 (ICDD)
Tungsten Carbide	W_2C	1.388907051635667	30653896 (ICDD)
Unnamed mineral, syn (NR)	WC	1.315216631957023	251047 (ICDD)
Wolfram	W	1.667238816853466	40806 (ICDD)

Phase data pattern

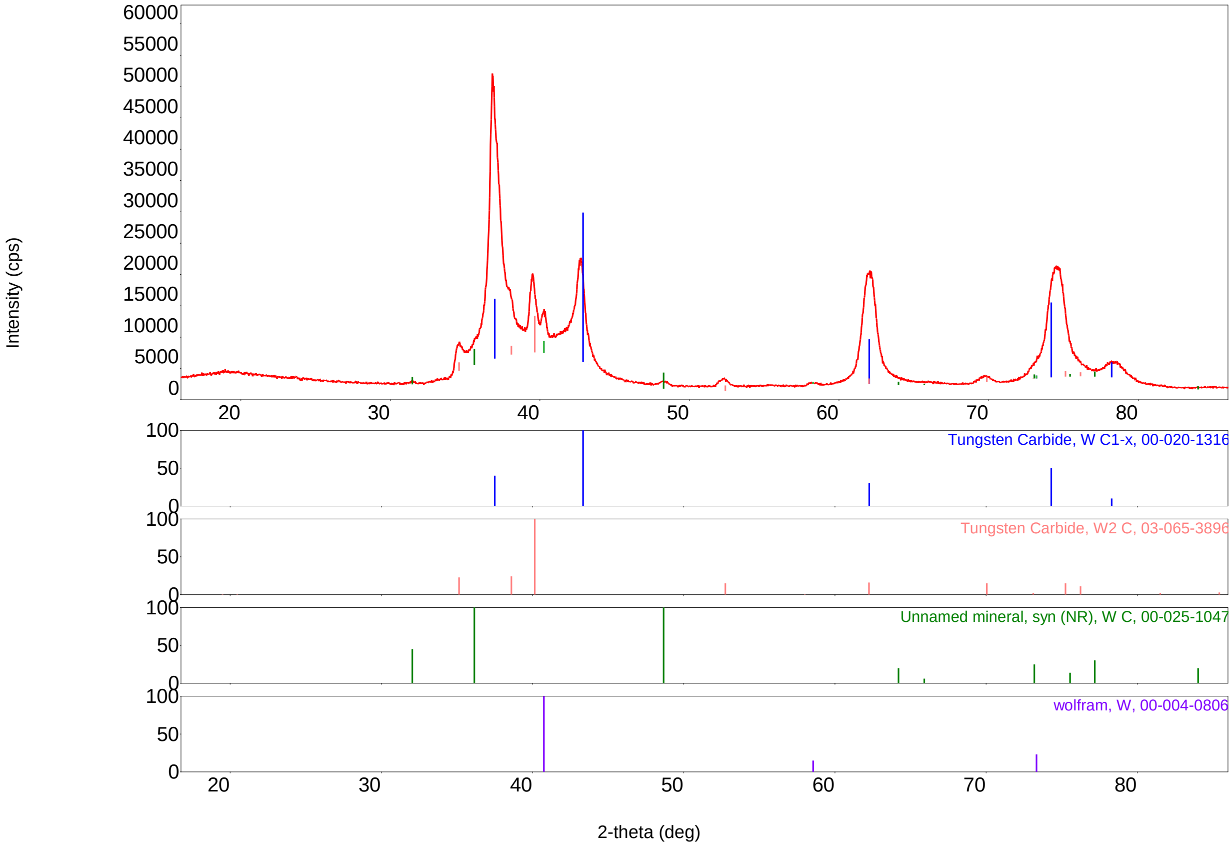

Figure S221. XRD diffraction pattern of initial W-C powder.

XRD diffraction pattern of W-C powder sample after MW treatment

Qualitative analysis results

Phase name	Formula	Figure of merit	Phase reg. detail
Tungsten Oxide	WO_3	0.4869597054349091	10830950 (ICDD)
Tungsten	W	1.047058038640831	10892767 (ICDD)
beta-WO_3	WO_3	1.034093539710211	10894480 (ICDD)

Phase data pattern

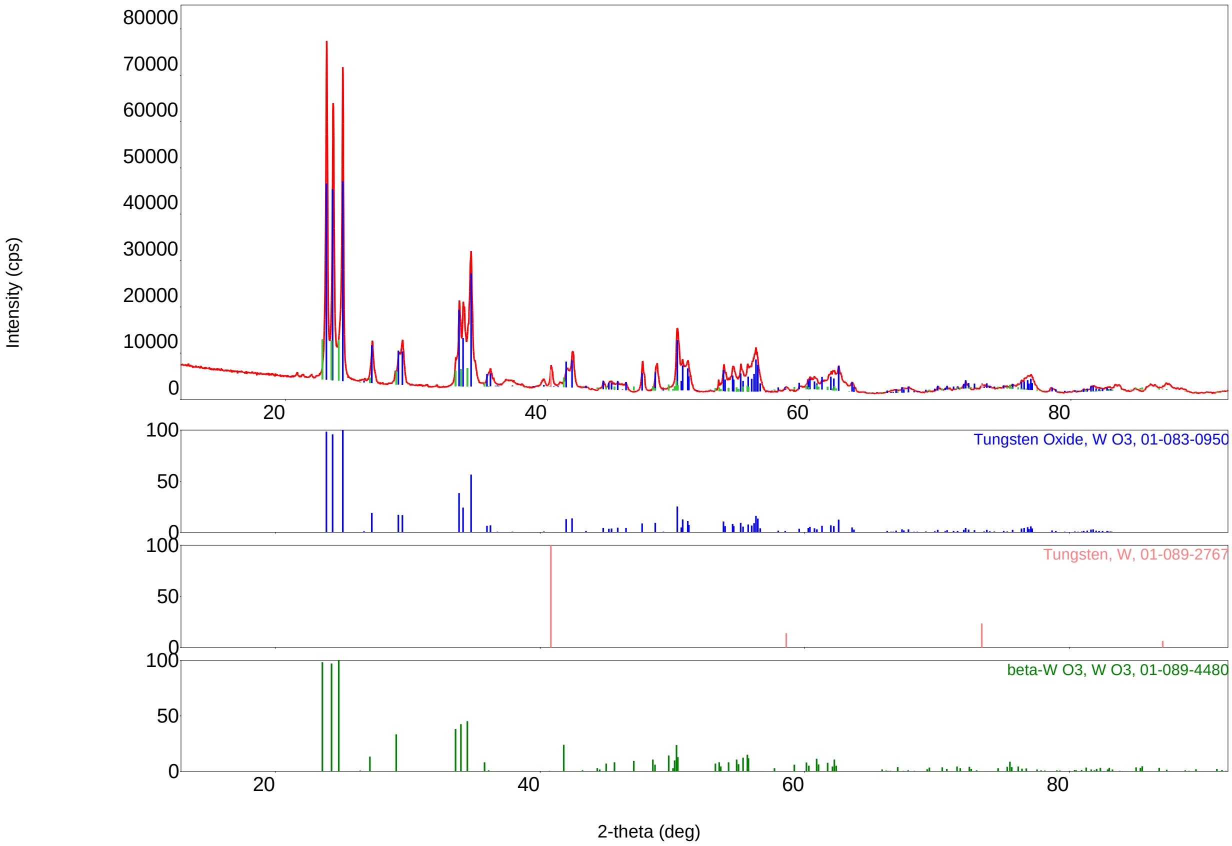

Figure S222. XRD patterns of the of W-C powder sample after MW treatment, with the reference diffraction patterns of WO_3 (PDF 01-083-0950), and other.

XRD diffraction pattern of W-V-C powder sample after MW treatment

Qualitative analysis results

Phase name	Formula	Figure of merit	Phase reg. detail
Tungsten Oxide	WO_3	0.4126909778378652	10830950 (ICDD)
Tungsten Oxide	WO_3	0.7244880639712449	10891287 (ICDD)
Tungsten	W	1.143433753013287	10892767 (ICDD)

Phase data pattern

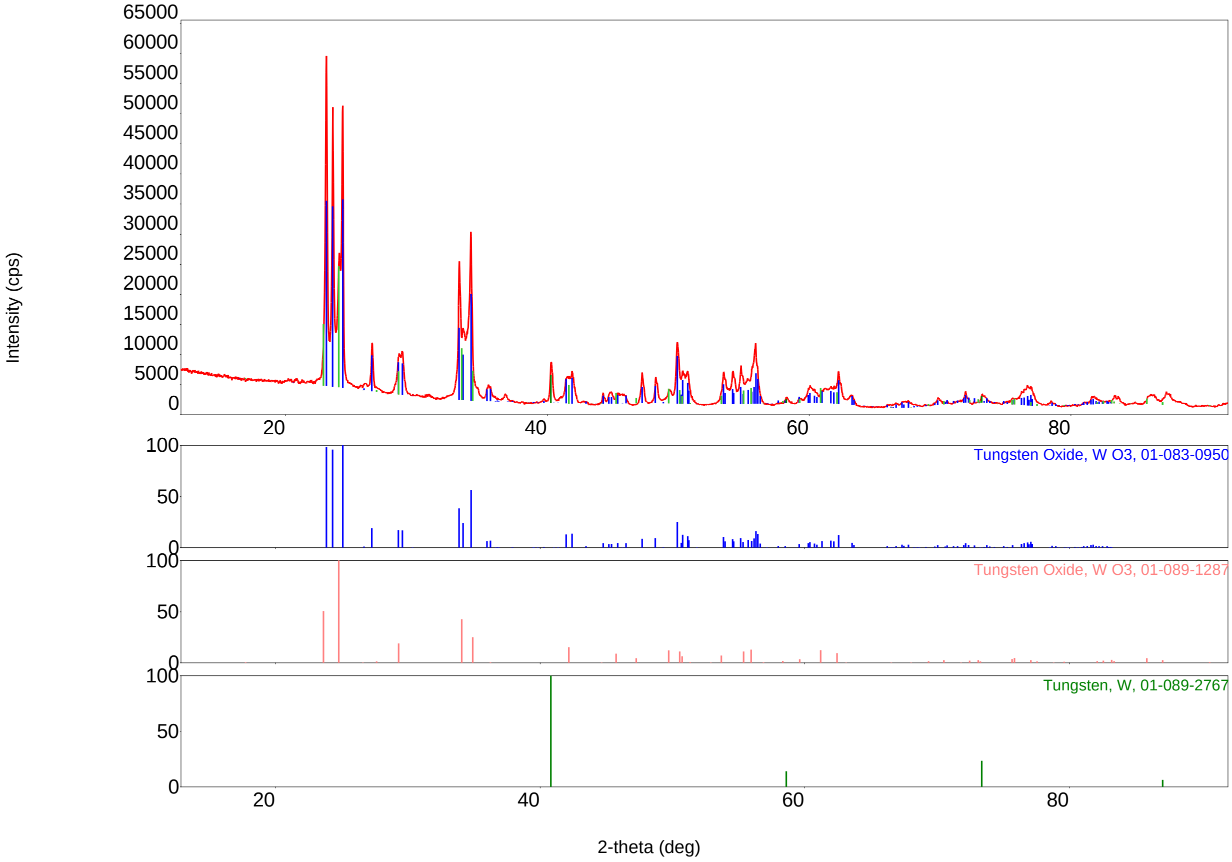

Figure S223. XRD patterns of the of W-V-C powder sample after MW treatment, with the reference diffraction patterns of WO_3 (PDF 01-083-0950), WO_3 (PDF 01-089-1287) and W (PDF 01-089-2767).
